# Identification and Molecular Characterization of a Novel Hordeivirus Associated With Yellow Mosaic Disease of Privet (*Ligustrum vulgare*) in Europe

**DOI:** 10.3389/fmicb.2021.723350

**Published:** 2021-09-27

**Authors:** Jean-Sébastien Reynard, Silvia Turco, Justine Brodard, Isabelle Kellenberger, François Maclot, Olivier Schumpp, Paul Gugerli, Mikhail M. Pooggin

**Affiliations:** ^1^Virology-Phytoplasmology Laboratory, Agroscope, Nyon, Switzerland; ^2^Department of Environmental Sciences, Botany, University of Basel, Basel, Switzerland; ^3^Laboratory, TERRA-Gembloux Agro-Bio Tech, University of Liège, Gembloux, Belgium; ^4^PHIM Plant Health Institute, University of Montpellier, INRAE, CIRAD, IRD, Institute Agro, Montpellier, France

**Keywords:** *Ligustrum*, *Hordeivirus*, distribution, virions, transmission, siRNAs, genome, phylogeny

## Abstract

Wild plants serve as a large reservoir of known and yet-unknown viruses and as a source of viral pathogens of cultivated plants. Yellow mosaic disease of forest shrub *Ligustrum vulgare* (privet) was recurrently observed in Europe for more than 100 years. Using a universal virus identification approach based on deep sequencing and *de novo* assembly of viral small interfering (si)RNAs we identified a causative agent of this disease in Switzerland and reconstructed its complete 3-segmented RNA genome. Notably, a short 3′-terminal common region (CR) attached to each segment via a ∼53–71 nucleotide poly(A) tract, as determined by RT-PCR sequencing, was initially identified as an orphan siRNA contig with conserved tRNA-like secondary structure. Phylogenomic analysis classified this virus as a novel member in the genus *Hordeivirus* of family *Virgaviridae*, which we named ligustrum mosaic virus (LigMV). Similar to other hordeiviruses, LigMV formed rod-shape virions (visualized by electron microscopy), was transmitted through seeds and could also be mechanically transmitted to herbaceous hosts *Chenopodium quinoa* and *Nicotiana benthamiana*. Blot hybridization analysis identified genomic and subgenomic RNAs, sharing the 3′-CR and likely serving as monocistronic mRNAs for seven evolutionarily-conserved viral proteins including two subunits of viral RNA-dependent RNA polymerase, coat protein, triple gene block proteins mediating viral movement and cysteine-rich suppressor of RNA silencing. Analysis of size, polarity, and hotspot profiles of viral siRNAs suggested that they are produced by the plant antiviral Dicer-like (DCL) proteins DCL2 and DCL4 processing double-stranded intermediates of genomic RNA replication. Whole genome sequencing of French and Austrian isolates of LigMV revealed its genetic stability over a wide geographic range (>99% nucleotide identity to Swiss isolates and each other), suggesting its persistence and spread in Europe via seed dispersal.

## Introduction

Wild plants host a large number of known and yet-unknown viruses and therefore serve an important source of newly emerging viral pathogens of cultivated plants, which justifies efforts to identify causative agents of wild plant diseases. Privet (*Ligustrum vulgare* L., family *Oleaceae*) is a wild shrub growing in forest edges and a very common plant in natural ecosystems of Switzerland and other European countries, which is also often used for ornamental and hedgerow purposes. Viral disease symptoms on privet were already reported at the beginning of the last century in Germany (Baur, 1906; Schmelzer, 1963). Erwin Baur (1875–1933), considered as one of the fathers of plant virology, studied in his pioneer works “infektiöse Chlorose” (infectious chlorosis) on different plants, including privet, and showed that viruses are the causal agents (Baur, 1904, 1906, 1908). The last sentence of Baur’s work from 1908 was the following: “Die nächste Aufgabe wird es jetzt sein, den rätselhaften Infektionstoff einigermassen zu isolieren” (The next task will now be to isolate the mysterious infection substance). Since that time, privet and other *Ligustrum* species were shown to be (naturally or experimentally) infected with several viruses including tomato black ring virus (genus *Nepovirus*; Smith, 1957), cucumber mosaic virus (*Cucumovirus*; Schmelzer, 1963), arabis mosaic virus (*Nepovirus*; Dupuis et al., 2008), ligustrum virus A (*Carlavirus*; Igori et al., 2016), ligustrum necrotic ringspot virus (*Carlavirus*; Scott and Zimmerman, 2008), privet ringspot virus (*Ilarvirus*; Aboughanem-Sabanadzovic et al., 2016) and privet leaf blotch-associated virus (*Idaeovirus*; Navarro et al., 2017).

Similar to Erwin Baur’s observations, we found a viral chlorosis affecting privets in Switzerland and neighboring countries. In this work we followed Baur’s investigations using modern techniques including electron microscopy, serology and, finally, deep small RNA (sRNA) sequencing and bioinformatics to identify and characterize the causal agent of infectious chlorosis in privet, which we named here ligustrum mosaic virus (LigMV). Viral small interfering RNAs (siRNAs) are known to be produced by an RNA interference (RNAi)-based antiviral defense mechanism in all land plants and to cover both strands of the entire genomes of RNA and DNA viruses as well as viral satellites and viroids, thus enabling *de novo* reconstruction of viral genomes from siRNA sequencing reads (Pooggin, 2018).

## Materials and Methods

### Plant Samples

In summer 2015, symptomatic and asymptomatic leaves were collected from a privet shrub (*L. vulgare*) in a forest close to Nyon (Vaud, Switzerland) on the edge of Lake Geneva (400 m.a.s.l.). From the leaf sample showing yellow mosaic symptoms, virus particles were purified and visualized by electron microscopy (see below) and found to be similar to those reported by René Bovey (Bovey, 1976). These plant samples were then used for total RNA extraction and Illumina sequencing of sRNAs to reconstruct the virus genome (see below).

To survey the geographic distribution of LigMV, symptomatic privet plants were collected in natural ecosystems in Switzerland from different regions (Geneva, Lausanne, Biel, Argau). Samples of symptomatic plants were also collected from one site in France (Bellegarde, Ain department) and one site in Austria (Innsbruck).

### Electron Microscopy Analysis of Ligustrum Mosaic Virus Virion Morphology

Ten grams of privet leaves were ground in liquid nitrogen and the powder was transferred to 50 ml of an extraction solution (0.18 M phosphate citrate, 0.2% thioglycolic acid, pH 7.0) and stirred for 20 min on ice. The solution was filtered through a double layer of gauze and supplemented with 25 ml ether and 25 ml tetrachloromethane before being stirred at room temperature for 5 min. The solution was clarified by centrifugation for 20 min at 4,000 rpm and the upper phase was taken for particle sedimentation by ultracentrifugation for 1 h at 40,000 rpm. The pellet was resuspended in 1 ml of 0.018 M phosphate citrate pH 7.0. Three microliters of the suspension were mixed with 3 μl of 0.1% bovine serum albumin and 3 μl of 4% phosphotungstic acid at pH 6.0. The solution was sprayed onto a microscopy grid as described by Bovey (1976) and observed using a Tecnai G2 Spirit transmission electron microscope (FEI, Eindhoven).

### Ligustrum Mosaic Virus Diagnostics by Reverse Transcription-PCR

Total RNA was extracted from mature leaves using RNeasy Plant Mini Kit (Qiagen). Based on the LigMV genomic information obtained by Illumina sequencing, a primer pair amplifying a portion of the viral RNA-dependent RNA polymerase (RdRP) was designed (LigMV_1079F and LigMV_2063R, Supplementary Table 1). The amplified fragments were cleaned up using PCR purification kit (Qiagen) and directly sequenced from both ends at Fasteris SA (Switzerland).

### Ligustrum Mosaic Virus Transmission to Herbaceous Hosts

Virus transmission was performed by grinding symptomatic privet leaves in 0.2 M Na_2_HPO_4_ (pH 7.6) and then gently rubbing the resulting extract onto carborundum-dusted leaves of herbaceous hosts *Chenopodium quinoa* and *Nicotiana benthamiana*. Inoculated plants were kept in a greenhouse at 20–22°C under natural light and were checked regularly for symptoms. The presence of virus infection was ascertained by the RT-PCR analysis as described above.

### Ligustrum Mosaic Virus Antiserum Production

LigMV virions were purified from locally infected leaves of *Chenopodium quinoa* as described previously (Gugerli, 1976). 200 μg purified virions were intravenously injected into a locally bred rabbit. Four days later the intravenous injection with 200 μg purified virions was repeated. Eight days later, an intramuscular injection with 200 μg purified virions 1:1 diluted with Freund’s complete adjuvant was done. Five blood samples (20–40 ml each) were taken at weekly intervals beginning at 7 days post-intramuscular injection. Antiserum was evaluated by Ouchterlony double diffusion tests as described by Gugerli (1976) and ELISA as described by Clark and Adams (1977).

### Assay for Ligustrum Mosaic Virus Seed Transmission

Mature fruits were collected on *L. vulgare* plants with yellow mosaic symptoms in the region of Nyon during autumn 2016 and 2018. Seeds were separated from fruit and cleaned by hand. Seeds were conserved for 3 months at 4°C and then were planted to assess seed transmission rate of the virus. Four months after germination, LigMV infection was evaluated by the RT-PCR analysis as described above.

### Total RNA Extraction for Small RNA Sequencing

One gram of privet leaf tissue was ground in liquid nitrogen, 5 ml of GHCL solution (6.5 M guanidine hydrochloride, 100 μM Tris HCl pH 8.0, 100 μM sodium acetate pH 5.5, 100 μM β-mercaptoethanol) was added and the resulting suspension was transferred into a sterile disposable polypropylene tube and mixed vigorously. After incubation at room temperature for 10 min, the suspension was centrifuged at 10,000 g for 10 min at 4°C and the supernatant was then transferred into a new tube. 2.5 ml Trizol reagent (Sigma) and 1 ml chloroform were added to the supernatant, the mixture was vortexed thoroughly and the centrifugation was repeated at the same conditions. The aqueous phase was transferred to a new tube and an equal volume of isopropanol was added, followed by vortexing. The mixture was incubated on ice for 30 min and then centrifuged at 10,000 g for 20 min at 4°C. The pellet was washed in 5 ml of 75% ethanol (pre-chilled on ice) and the precipitation was prolonged for 90 min at −20°C. The RNA was then pelleted at 10,000 g for 10 min at 4°C, vacuum-dried for 10 min and dissolved in DEPC-treated water at 65°C for 10 min. Total RNA concentration was measured using NanoDrop Spectrophotometer (Thermo Fisher Scientific) and Qubit RNA HS Assay Kit (Thermo Fisher Scientific).

### Illumina Sequencing and Bioinformatic Analysis of Small RNAs

Sequencing was performed at Fasteris SA (Switzerland)^1^ : a 19–30 nt fraction of total RNA was taken for cDNA library preparation using TruSeq small RNA kit. Ten libraries including HYT-23 (non-symptomatic *L. vulgare* leaf sample) and HYT-24 (symptomatic *L. vulgare* leaf sample) were multiplexed and sequenced in one lane of HiSeq 2500, yielding 11,185,665 (HYT-23) and 11,332,891 (HYT-24) clusters with Q30 of 94.99 and 95.33%, respectively. After adapter trimming, HYT-23 and HYT-24 contained respectively 8,960,951 and 9,657,337 reads in a size range from 18 to 28 nt.

For *de novo* assembly of sRNAs, Velvet 1.2.10 (Zerbino and Birney, 2008)^2^ was used with k-mer values set to 13, 15, 17, 19, 21, and 23. The graphs created by Velvet were then used by Oases 0.2.09 (Schulz et al., 2012).^3^ The Oases contigs obtained with different k-mers values (13, 15, 17, 19, 21, 23) were combined in one single FASTA file for each sample, the host plant related contigs were filtered out by BWA mapping of the HYT-24 contigs to the HYT-23 contigs and the resulting viral contigs were scaffolded using a Seqman module of Lasergene DNAstar package (SeqMan Pro 12.0.0). The Seqman contigs were analyzed by BLASTn to identify viral contigs and the consensus sequences of three genomic RNA (gRNA) segments of LigMV (see Supplementary Dataset 1) were determined as described below. The Integrative Genomics Viewer (IGV; Thorvaldsdóttir et al., 2013) was used to visualize Oases or Seqman contigs mapped using BWA to the viral reference sequences.

For viral sRNA profiling, the redundant 20–25 nt sRNA reads were mapped using BWA to the consensus sequences of reconstructed LigMV gRNAs with zero mismatches and with up to 2 mismatches. The resulting mapping data were analyzed using in-house scripts to sort and count the viral sRNAs by size (20, 21, 22, 23, 24, 25 nt, total 20–25 nt), polarity (forward, reverse, total) and 5′-terminal nucleotide identity (5′A, 5′C, 5′G, 5′U) and generate count tables (Supplementary Dataset 2). MISIS-2 (Seguin et al., 2016) was used for further analysis of viral sRNAs including visualization of 5′-nucleotide frequencies and generation and visualization of single-nucleotide resolution maps for all size-classes (Supplementary Dataset 3) as well as for determination of single nucleotide polymorphism (SNP) variants in consensus sequences.

### Reverse Transcriptase-PCR and Rapid Amplification of cDNA Ends Validation of 3′-Common Regions and Termini of Ligustrum Mosaic Virus gRNAs

Two antisense oligonucleotides (LigMV_3UTR_79as and LigMV_3UTR_133as; Supplementary Table 1) were designed on a 3′-common region (CR) contig with tRNA-like structure (see below) and used as primers for a reverse transcriptase (RT) reaction as follows. One μg of total RNA was incubated with 1 μl 10 mM dNTP mix, 2 pmol of RT primer, 4 μl of 5x first-strand synthesis buffer [250 mM Tris-HCl (pH 8.3), 375 mM KCl, 15 mM MgCl_2_, 0.1 M DTT], 1 μl 0.1 M DTT, 1 μl (40 U) of RNase inhibitor RNasin (Promega) and 1 μl (200 U) of Superscript III reverse transcriptase (Invitrogen) and incubated at 55°C for 60 min. The reaction was stopped by heating at 70°C for 15 min.

PCR amplification was performed in 25 μl of 1x PCR buffer supplied with 1.5 mM of magnesium chloride, 0.5 μl of 10 mM dNTP mix, 2.5 U Taq polymerase (Sigma), 20 pmol of RT primer (3UTR_79as or 3UTR_133as), 20 pmol of gRNA segment-specific primer (Supplementary Table 1 and Supplementary Figure 1A) and 1 μl of the RT reaction mixture as template for one cycle at 95°C for 10 min, 35 cycles of 95°C for 30 s, 52°C for 30 s, and 72°C for 1 min, and a final cycle at 72°C for 3 min.

The products were analyzed by electrophoresis on a 1.5% agarose gel in TAE buffer (40 mM Tris acetate, 1 mM EDTA, pH 8.0) and stained with ethidium bromide. The PCR products were purified from the gel (Supplementary Figure 1A) with the GenElute^TM^ Gel Extraction Kit (Sigma) and sequenced directly or cloned and sequenced (Fasteris SA, Switzerland). The sequencing reads are shown in Supplementary Figures 1B–D.

For 3′-RACE analysis, RNA was polyuridylated using Poly(U) polymerase (New England Biolabs) and then converted to cDNA using reverse transcriptase Superscript III (Invitrogen) primed with DNA oligonucleotide 5′-AAAAAAAAAAAAAAAAAAAAAATGG, followed by PCR using the latter primer in pair with forward primer 5′-TGCCTGCTATTAAGACGGTG specific to the LigMV 3′-CR contig. For 5′-RACE analysis, GeneRacer kit (Invitrogen) was used following the manufacturer’s protocol. RT-PCR was performed using the GeneRacer 5′-adaptor primer in pair with the LigMV segment-specific reverse primers: LigMValpha251as, LigMVbeta205as, or LigMVgamma276as (Supplementary Table 1). The resulting PCR products (at least two per each gRNA) were cloned in pGM-T (Promega) and sequenced.

### Extraction and Illumina Sequencing of Viral Double-Stranded RNA

dsRNA was extracted from LigMV-infected *L. vulgare* leaves, purified by CF11 cellulose batch chromatography and then sequenced as described previously (Valverde et al., 1986; Candresse et al., 2013). Briefly, purified dsRNA was denatured for 5 min at 99°C and converted to cDNA by reverse transcription with primer PcDNA_12_ (5′-TGTGTTGGGTGTGTTTGGN_1__2_-3′) using the Superscript II RT (Invitrogen) according to the manufacturer’s instructions, followed by a whole-genome amplification procedure. The amplified material was sequenced on HiSeq HO 2 × 125 nt v4 chemistry lane at Fasteris SA (Switzerland). *De novo* assembly of LigMV genome was performed using Geneious.^4^

### Extraction and Illumina Sequencing of Virion-Associated RNA

LigMV particle purification and RNA extraction were performed using the virion-associated nucleic acid (VANA) protocol as described previously (Maclot et al., 2021). cDNA libraries were then prepared according to the protocol of Palanga et al. (2016). Illumina sequencing was done at Fasteris SA (Switzerland) using the NEBNext Ultra II DNA library prep kit (New England BioLabs) and NextSeq platform with 2 × 150 nt sequencing reads. Raw reads were demultiplexed and the adaptors trimmed with Cutadapt (Martin, 2011). The trimmed reads were then assembled in contigs by SPAdes (Bankevich et al., 2012) on the Durandal cluster (ULiège, Belgium).

### RNA Blot Hybridization Analysis

For Northern blotting hybridization analysis of high molecular weight RNA, 10 μg of total RNA were vacuum-dried and resuspended in 5 μl DEPC-treated water and mixed with 5 μl RNA Gel Loading Dye (Thermo Fisher Scientific). The samples were incubated at 70°C for 10 min and loaded on formaldehyde-containing agarose gel [1.2% agarose, 3% formaldehyde, 1x MOPS buffer (0.02 M MOPS pH 7.0, 1 mM EDTA, 5 mM sodium acetate)], followed by electrophoresis for 2.5 h at 100 V. RNA was transferred by capillarity blotting to a Hybond N + membrane (Amersham) for 24 h in transfer buffer (50 mM NaH_2_PO_4_, 5 mM EDTA, pH 6.5) and cross-linked twice with 1,200 μjoules × 100 UV light in a Statalinker 1800 (Stratagene).

For sRNA blot hybridization analysis, 10 μg of total RNA were dried in a SpeedVac, resuspended in 10 μl of RNA Gel Loading Dye (Thermo Fisher Scientific), warmed at 95°C for 3 min and loaded on 15% polyacrylamide-urea gel (19:1 acrylamide:bis-acrylamide and 8 M urea), followed by electrophoresis at 3 V for 4 h. RNA was then transferred to a Hybond N + membrane (Amersham) by electroblotting in 1x TBE buffer at 10 V overnight at 4°C. The RNA was cross-linked to the membrane twice with 1,200 μ joules × 100 UV light in a Stratalinker 1800 (Stratagene).

Blot hybridization was performed as described previously (Akbergenov et al., 2006). Briefly, DNA oligonucleotide probes specific for LigMV 3′-CR, gRNAs and sgRNAs were designed based on the reconstructed consensus viral sequences (see Supplementary Table 1 for probe sequences). The DNA oligonucleotides were end-labeled with P32 γ-ATP (Hartmann Analytic) by T4 polynucleotide kinase (Roche) and purified through MicroSpin G-25 columns (Amersham). The hybridization was carried out overnight at 35°C in UltraHyb-oligo buffer (Ambion) followed by washing 3 times with 2X SCC, 0.5% SDS for 30 min at 35°C. The membranes were exposed for 1–7 days to a phosphor screen and scanned in a GE Typhoon 8600 imager (GE Healthcare Life Sciences). For repeated hybridizations the membrane was stripped with 0.5X SSC, 0.5% SDS for 30 min at 80°C and then with 0.1X SSC, 0.5% SDS for 30 min at 80°C.

### Bioinformatic Tools for Phylogenomic Analysis

For phylogenomic analysis of LigMV we used the NCBI tools BLASTn, ORF finder, Smart Blast and CD (Conserved Domains)-search^5^ as well as Sequence Demarcation Tool (STD) v1.2 with MUSCLE option (Muhire et al., 2014)^6^ and MAFFT v7 (Katoh and Standley, 2013).^7^

## Results

### Virion Morphology and Lab Hosts of Ligustrum Mosaic Virus

**FIGURE 1:**
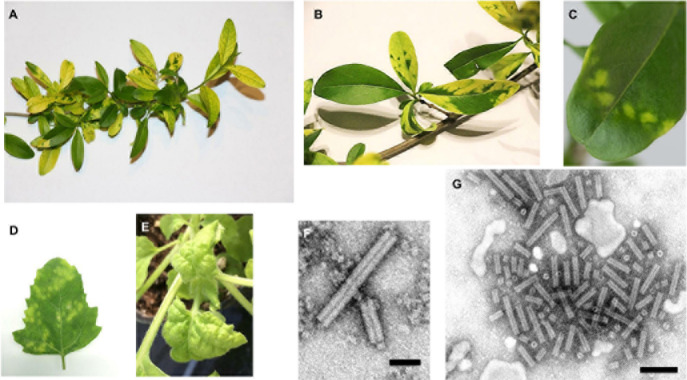
Ligustrum mosaic virus (LigMV) disease symptoms and virion morphology. **(A–C)** *Ligustrum vulgare* plants collected near Nyon (Switzerland) in 2015. **(D)** *Chenopodium quinoa* and **(E)** *Nicotiana benthamiana* infected with LigMV via mechanical inoculation with sap extract from the symptomatic leaves of *L. vulgare*. Photographs were taken 15 **(D)** and 10 **(E)** days post inoculation. **(F,G)** Electron micrographs of purified LigMV particles stained with phosphotungstic acid. The size bars represent 50 nm **(F)** and 100 nm **(G)**.

Since the 1970th, a virus-like disease of privet has been recurrently observed in Switzerland and neighboring countries. The disease symptoms are manifested by yellow mosaic variegation on privet leaves (Figures 1A–C) that usually appeared at the beginning of the growth period and were observable until late autumn. Electron microscopy analysis of partially purified extracts from the diseased (but not healthy) plants has revealed “tobravirus-like” rod-shaped virions of ca. 22 nm in diameter and ca. 110–150 nm in length (Bovey, 1976), although longer and shorter particles were also observed (Figures 1F,G).

The virus isolated from symptomatic privet leaves could be transmitted to *Chenopodium quinoa* and *Nicotiana benthamiana* by mechanical inoculation, causing local chlorotic lesions and systemic disease symptoms (chlorosis and downward leaf curling), respectively (Figures 1D,E). Antibodies raised in rabbit against this virus and shown to be specific to its coat protein did not cross-react to tobacco rattle virus (genus *Tobravirus*) or barley stripe mosaic virus (genus *Hordeivirus*) (data not shown).

### *De novo* Reconstruction of a Complete 3-Segmented RNA Genome of Ligustrum Mosaic Virus by Illumina Small RNA Sequencing

**FIGURE 2:**
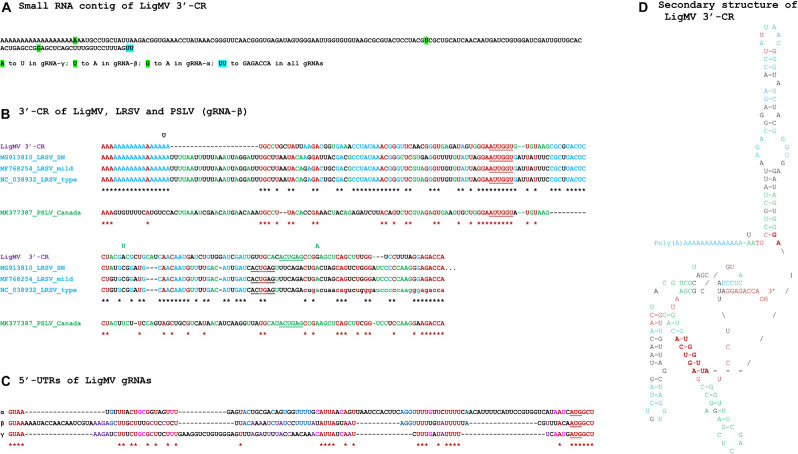
Ligustrum mosaic virus (LigMV) 3′-common region (CR) and 5′-UTR sequences and comparison of the 3′-CRs of LigMV and other hordeiviruses. **(A)** Sequence of the small RNA contig representing the LigMV 3′-CR. The nucleotide positions that distinguish the 3′-CRs of three genomic RNA (gRNA) segments (α, β, γ) are highlighted in green, with the nucleotide substitutions indicated below the contig sequence. The last two nucleotides of the contig (UU, highlighted in cyan) are not confirmed by 3′-RACE analysis and, based on this analysis, are replaced with GAGACCA in all three gRNAs. **(B)** Alignment of the 3′-CRs of LigMV (isolate CH), lychnis ringspot virus (LRSP) isolates SN (NCBI accession: MG913810), mild (MF768254) and type (NC_038932), and poa semilatent virus (PSLV) isolate Canada (MK377387). The lowercase nucleotides in LRSV type strain—missing in the reference sequence NC_038932—are added according to Solovyev et al. (1996). The nucleotides identical in all the viral species and isolates are shown in red and indicated in red asterisks’ consensus. The nucleotides identical between LigMV and all the LRSV isolates are shown in cyan and the LigMV-LRSV consensus sequence is indicated with black asterisks. The nucleotides identical only between LigMV and PSLV or only between PSLV and LRSP are shown in green. The nucleotides involved in formation of ascending and descending arms of the central helix of the tRNA-like structure are underlined. The nucleotide changes distinguishing LigMV gRNAs **(A)** are shown above the alignment at corresponding positions. **(C)** Comparison of the 5′-untranslated regions (UTRs) of LigMV gRNAs α, β, and γ. The identical nucleotides shared by all the three gRNA segments are shown in red and indicated with asterisks. The additional nucleotides identical only between α and β, or α and γ, or β and γ, are shown in blue, pink, and purple, respectively. The AUG start codon of each gRNA is underlined. **(D)** Tentative secondary structure of the LigMV 3′-CR sequence. For its comparison with tentative secondary structures of 3′-CRs of PSLV, LRSV, and BSMV, see Supplementary Figure 2. The structural elements identical to those found either in LRSV or PSLV or in both viruses are highlighted in cyan, green, and red, respectively. Bold red indicates the two primary sequence elements GGAAUUGGU and ACUGA involved in formation of the central helix of the tRNA-like structure and its connection to the hairpin stem, which are also conserved in the predicted tRNA-like structure of BSMV (Supplementary Figure 2B). The nucleotide changes distinguishing LigMV gRNAs **(A)** and shown at the corresponding positions do not destabilize any secondary structure element.

Total RNA extracts from non-symptomatic (HYT-23) and symptomatic (HYT-24) privet leaf samples (collected near Nyon in 2015) were subjected to Illumina sequencing of 18–30 nt sRNAs as described in “Materials and Methods” section. For each sample, redundant reads in the range of 20–25 nt (9,608,673 and 10,745,889 reads, respectively) were assembled using Velvet/Oases (k-mer values set from 13 to 23, with minimum contig length of 50 nts) and the resulting contigs were combined. The contigs from HYT-23 were used to filter out host-related contigs from HYT-24 using a mapping tool BWA and the resulting unmapped contigs were scaffolded using Seqman (for more details see section “Materials and Methods”). BLASTn analysis of the Seqman contigs revealed three large contigs distantly related to three genomic RNA (gRNA) segments (α, β and γ) of lychnis ringspot virus (LRSV) and barley stripe mosaic virus (BSMV), both belonging to the genus *Hordeivirus* of family *Virgaviridae* (Adams et al., 2017; Savenkov, 2021). Notably, the γ-related contig was found to be much larger than expected for hordeiviral γ segments, due to an inverted repeat in the 5′-terminal region. After correction of this apparent artifact of *de novo* assembly, the three contigs were used as reference sequences to map redundant 20–25 nt reads from HYT-24 and determine the consensus sequence. The consensus sRNA contigs corresponded both in size and protein-coding capacity to the respective segments of LRSV and BSMV, except that they all appeared to lack the 3′-untranslated common region (3′-CR) with a tRNA-like structure usually attached via a poly(A) tract to each hordeivirus segment (Agranovsky et al., 1978, 1983; Jackson et al., 2009). Screening of orphan Oases contigs for presence of stem-loop secondary structures using MFold allowed us to identify a 177 nt contig with the primary sequence distantly related to that of LRSV 3′-CR (Figures 2A,B).

RT-PCR analysis with primers designed based on the sRNA contigs’ sequences (Supplementary Figure 1A and Supplementary Table 1), followed by cloning and sequencing (or direct sequencing) of the resulting PCR products from both ends confirmed that the orphan contig with tRNA-like secondary structure is attached via a ∼53–71 nt poly(A) tract to the 3′-terminus of each gRNA. Note that we were not able to determine precisely the number of adenosines in the poly(A) tracts, because of polymerase slippage on these homopolymeric sequences (Supplementary Figures 1B–D). For further analysis, we set the poly(A) tract length to 59 nts in each gRNA segment of LigMV.

To validate the 5′- and 3′-terminal sequences of each gRNA segment we employed 5′- and 3′-RACE (rapid amplification of cDNA ends), respectively, as described in “Materials and Methods” section. The 5′-RACE revealed that the 5′-terminus of gRNA-α is 2 nt shorter than that of the sRNA contig. Interestingly, the first and second nucleotides in the α contig sequence are supported by 4 and 55 sRNA reads of antisense polarity, some of which showing SNPs at the corresponding positions, suggesting that one to two non-template nucleotides may have been added to sRNA precursors by viral (or host) RNA-dependent RNA polymerase. In the case of gRNA-β, the 5′-RACE did not support existence of the first 33 nt of the β contig, while in the case of gRNA-γ the first 17 nts differed at 11 positions from those in the γ contig (Supplementary Dataset 1A). The 3′-RACE was done using an approach similar to that developed earlier for sequencing of hordeiviral tRNA-like 3′-terminal sequences which universally end with CCA_OH_ (Kozlov et al., 1984; Supplementary Figure 2): RNA was polyuridylated using poly(U) polymerase and then converted to cDNA using reverse transcriptase primed with DNA oligonucleotide 5′-AAAAAAAAAAAAAAAAAAAAAATGG-3′, followed by PCR using the latter primer in pair with a forward primer specific to the LigMV 3′-CR contig (Supplementary Table 1). Cloning and sequencing of the resulting PCR product (two independent clones) determined the correct 3′-terminal 6 nucleotides ending with CCA, which were substituted with 2 uridines in the sRNA contig (Figures 2A,B).

Simultaneous mapping of 20–25 nt sRNA reads on the reference sequences of LigMV gRNAs α, β, and γ (corrected based on the RT-PCR and RACE results) confirmed the complete consensus genome sequence of the virus (deposited to NCBI Genbank under the accession numbers MW752157, MW752158 and MW752159). Notably, the 5′-RACE-corrected nucleotides in 5′-UTRs and three SNP positions distinguishing α, β, and γ 3′-CRs were fully supported by sRNA reads (Supplementary Figure 3). The only polymorphic positions identified in the LigMV genome were the 3′-CR terminal cytosine and adenine which were replaced by uridines in a fraction of sRNA reads (Supplementary Figure 3).

### Phylogenomic Analysis of Ligustrum Mosaic Virus

**FIGURE 3:**
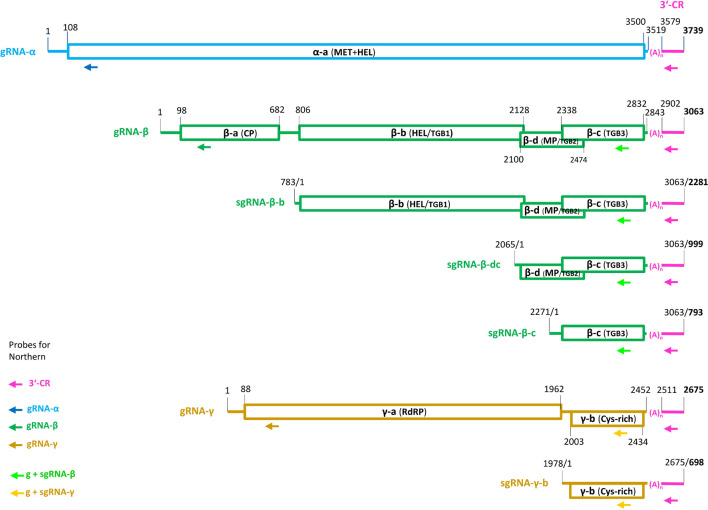
Genome organization of ligustrum mosaic virus (LigMV). The genomic (g) and subgenomic (sg) RNAs are shown schematically with the open rectangles representing the open reading frames (ORFs). Pink color indicates the 3′-terminal common region (CR) with the tRNA like secondary structure, connected to each RNA via a poly(A) tract (*n* = 59 nucleotides). The nucleotide numbering is from the 5′-end of each RNA (for the sgRNAs, their positions on the gRNA are also indicated). The gRNA-α encodes the α-a protein with N-terminal methyl-transferase (MT) and C-terminal helicase (HEL) domains. The gRNA-β encodes four proteins: the coat protein (CP) (β-a) translated from the gRNA-β itself; the triple gene block protein 1 (TGBp1, also named β-b) translated from sgRNA-β-b; and the TGBp2 (β-d) and TGBp3 (β-c) translated from bicistronic sgRNA-β-dc by a leaky scanning. The TGBp3 may also be translated from a putative monocistronic sgRNA-β-c. The gRNA-γ encodes two proteins: the γ-a protein (the polymerase subunit of the replicase RdRP) translated from gRNA-γ itself and the cysteine-rich γ-b pathogenicity protein translated from sgRNA-γ-b. The arrows indicate positions of the DNA oligonucleotide probes used for Northern blot hybridization analysis of LigMV RNAs.

NCBI ORF finder and protein BLAST analyses revealed that LigMV genome encodes seven proteins—one in gRNA-α, four in gRNA-β, and two in gRNA-γ—all homologous to the respective proteins encoded by other hordeiviruses (Figure 3).

**FIGURE 4:**
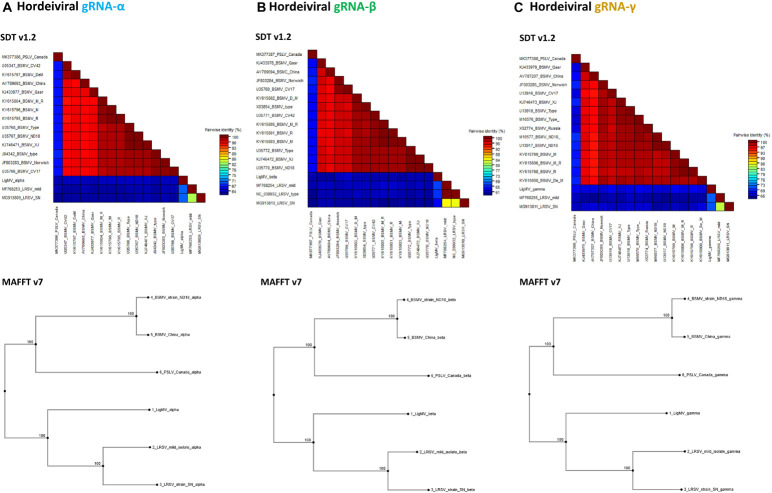
Phylogenetic analysis of ligustrum mosaic virus (LigMV). Percentage of pairwise nucleotide identity (excluding indels) between complete sequences of hordeiviral gRNAs α **(A)**, β **(B)**, and γ **(C)**, was calculated using Sequence Demarcation Tool (SDT v1.2; Muhire et al., 2014) with MUSCLE option and shown as multicolor heat maps. The NCBI GenBank accession numbers of several isolates of BSMV, two isolates of LRSV and a single available isolate of PSLV are indicated. Phylogenetic trees were generated using MAFFT v7.

The 3,739 nt gRNA-α of LigMV is most similar to that of LRSV mild isolate (MF768253), sharing 69% nt identity (Figure 4A) within 95% query coverage. The 1,130 aa residues long α-a protein encoded by this gRNA is most similar to its homolog from LRSV mild isolate (1,141 aa; 99% coverage, 71% identities, 83% positives) and contains viral methyltransferase (pfam01660), viral methyltransferase C-terminal (pfam08456) and viral superfamily 1 RNA helicase (pfam01443) domains, which are implicated in gRNA capping and replication of hordeiviruses (Jackson et al., 2009).

The 3,063 nt gRNA-β of LigMV is most similar to that of LRSV mild isolate (MF768254), sharing 72% identity (Figure 4B) within 50% query coverage. The 194 aa β-a protein (22 kDa) is most similar to its homolog from LRSV type strain (NC_038932) (195 aa; 97% coverage, 61% identities, 76% positives) and contains a TMV coat superfamily domain (pfam00721) identified in coat proteins of + ssRNA tobamoviruses, hordeiviruses, tobraviruses, furoviruses, and potyviruses. The 440 aa β-b protein (49 kDa) is most similar to β-b protein of LRSV isolate SN (MG913810) (441 aa; 89% coverage, 58% identities, 75% positives) and contains a viral superfamily 1 RNA helicase domain (pfam01443). The 124 aa β-d protein (14 kDa) is most similar to its homolog from LRSV type strain (123 aa; 99% coverage, 73% identities, 86% positives) and contains a viral beta C/D like family domain (pfam04530). The 164 aa β-c protein (19 kDa) is most similar to its homolog of LRSV type strain (157 aa; 98% coverage, 44% identities, 59% positives) and contains a plant virus movement protein domain (pfam01307). The overlapping ORFs β-b, β-d, and β-c together constitute a conserved triple gene block (TGB) module encoding, respectively, TGBp1, TGBp2, and TGBp3 proteins, implicated in cell-to-cell and systemic movement of hordeiviruses and some other genera of plant + ssRNA viruses (Jackson et al., 2009).

The 2,675 nt gRNA-γ of LigMV is most similar to that of LRSV mild isolate (MF768255), sharing 74% identity (Figure 4C) within 73% query coverage. The 624 aa γ-a protein (72 kDa) [whose translation is likely initiated at a second of the two adjacent AUGs, located in the most optimal context with A at position −3 and G at position + 4, conserved in all three gRNAs (Figure 2C)] is most similar to its homolog from LRSV mild isolate (627 aa; 99% coverage, 76% identities, 85% positives) and contains an RNA-dependent RNA polymerase RdRP_2 superfamily domain (pfam00978), implicated in gRNA replication and sgRNA transcription of hordeiviruses and some other genera of + ssRNA viruses (Jackson et al., 2009). The 143 aa γ-b protein (16 kDa) [whose translation is likely initiated at the second in-frame AUG of γ-b ORF, based on its stronger context with G at position + 4, a predicted position of sgRNA-γ-b transcription start site, and identification of a 1 nt indel between the two AUGs in French isolate of LigMV (see below; Supplementary Dataset 1A)] is most similar to its homolog from LRSV type strain (NC_038933) (144 aa; 100% coverage, 52% identities, 64% positives) and contains a viral_P18 domain (pfam04521), identified in hordeiviral γ-b and other cysteine-rich proteins of + ssRNA viruses and implicated in zinc and RNA binding and suppression of RNA silencing (reviewed in Jackson et al., 2009).

MFold-assisted manual folding of the 3′-CR sequence of LigMV gRNAs into secondary structure revealed a hairpin-like stem-loop structure immediately downstream of the poly(A) tract followed by a 3′-tRNA-like structure containing a pseudoknot (Figure 2D). This structural configuration is remarkably similar to that predicted in 3′-UTRs of LRSV and poa semilatent virus (PSLV) (Solovyev et al., 1996; see Supplementary Figure 2A), being composed of the structural elements identical (or similar) to those found either in LRSV or PSLV or in both viruses (Figures 2B,D, highlighted in cyan, green and red, respectively). Moreover, the two primary sequence elements GGAAUUGGU and ACUGA involved in formation of a central helix of the tRNA-like structure (underlined nucleotides) and its connection to the hairpin stem-loop (shown in bold red in Figure 2D) are also conserved in the predicted tRNA-like structure of BSMV (Kozlov et al., 1984; Supplementary Figure 2B). Notably, three SNPs that distinguish the 3′-CRs of LigMV gRNA segments (Figure 2A) do not alter the secondary structure (Figure 2D).

Alignment of the 5′-UTRs of LigMV gRNAs revealed, besides the conserved initiation context of the 5′-proximal genes mentioned above, other four common sequence motifs (Figure 2C), which can potentially serve as *cis*-acting elements in translation, packaging and/or replication of gRNAs. Analysis of their secondary structures, however, did not reveal any striking similarities (data not shown). Furthermore, comparison of each of the three 5′-UTRs of LigMV with the corresponding 5′-UTRs of other hordeiviruses did not reveal any striking common features except for short sequence motifs, although pairwise alignments showed relatedness of LigMV to each hordeivirus (Supplementary Dataset 1B).

**FIGURE 5:**
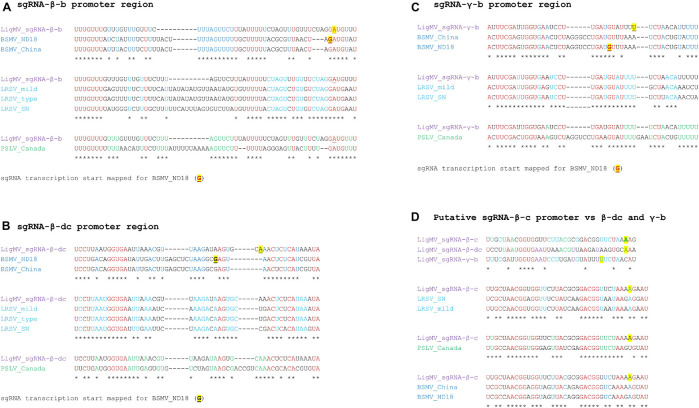
Sequence analysis of ligustrum mosaic virus (LigMV) subgenomic RNA (sgRNA) promoters. The sequences preceding predicted transcription start sites of the sgRNAs β-b **(A)**, β-dc **(B),** and γ-b **(C)** and the putative sgRNA-β-c **(D)** were compared with the respective sequences from barley stripe mosaic virus (BSMV) isolates ND18 (NCBI accession: U35770) and China (AY789694), lychnis ringspot virus (LRSV) isolates SN (MG913810), mild (MF768254) and type (NC_038932), and poa semilatent virus (PSLV) isolate Canada (MK377387). The nucleotides identical in all the viral species and isolates are shown in red. The nucleotides identical between LigMV and both BSMV isolates are shown in blue. The nucleotides identical between LigMV and all the LRSV isolates are shown in cyan. The nucleotides identical between LigMV and PSLV are shown in green. Consensus nucleotides are indicated by asterisks. The sgRNA transcription start sites mapped for the BSMV isolate ND18 (Zhou and Jackson, 1996b) are underlined and highlighted in yellow. The predicted start sites for LigMV sgRNAs are highlighted in yellow.

It has been established that 5′-distal ORFs of hordeiviral gRNAs β and γ are translated from subgenomic (sg)RNAs. The transcription start sites of these sgRNAs were precisely mapped only for BSMV strain ND18 (Gustafson et al., 1987; Zhou and Jackson, 1996b). Our comparative analysis of sgRNA promoter regions revealed conserved elements shared by all the four hordeiviruses and allowed us to predict, taking into account indels in LigMV vs. BSMV, the transcription start sites for LigMV sgRNAs β-b, β-dc, and γ-b (Figures 5A–C; underlined nucleotides). Interestingly, the promoters of LigMV sgRNAs β-dc and γ-b share sequence similarities including a GGUG motif with each other and with a sequence located upstream of β-c ORF (Figure 5D), suggesting that the latter ORF may also be expressed from a separate sgRNA. On the other hand, the sequence between the AUG start codons of β-d and β-c ORFs contains no other AUG (see Supplementary Dataset 1A), which is consistent with a leaky scanning strategy allowing translation of these two ORFs from a bicistronic sgRNA-β-dc as established for BSMV and other hordeiviruses (Jackson et al., 2009).

Phylogenetic analysis using MAFFT either with complete concatenated hordeiviral genomes or with separate gRNA segments revealed that LigMV is more related to LRSV than to PSLV or BSMV (Figure 4).

Taken together our phylogenomic analyses showed that LigMV can be defined as a distinct novel species in the genus *Hordeivirus* sharing with other hordeiviruses (i) the genome organization, (ii) protein functional domains and (iii) *cis*-acting RNA elements required for translation of viral proteins, gRNA replication and sgRNA transcription.

### Northern Blotting Hybridization Analysis of Ligustrum Mosaic Virus Genomic and Subgenomic RNAs

**FIGURE 6:**
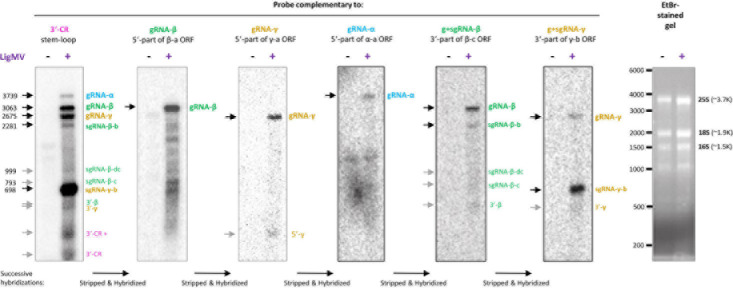
Northern blot hybridization analysis of ligustrum mosaic virus (LigMV) genomic (g) and subgenomic (sg) RNAs. Total RNA from healthy (–) and LigMV-infected (+) *Ligustrum vulgare* leave samples (HYT-23 and HYT-24, respectively) was separated on 1.2% agarose denaturing gel. The gel was stained with ethidium bromide (EtBr) and then RNA was transferred and cross-linked to nylon membrane. The membrane was successively hybridized with DNA oligonucleotide probes complementary to the 3′-common (CR) stem-loop sequence, 5′-part of the β-a open reading frame (ORF), 5′-part of the γ-a ORF, 5′-part of the α-a ORF, 3′-part of the β-c ORF, and 3′-part of the γ-b ORF (the probe sequences are given in Supplementary Table 1, and their positions on the virus genome are indicated in Figure 3). Cropped images of the membrane scans after each hybridization and the EtBr-stained gel are shown (see uncropped images and their alignment in Supplementary Figure 4). The positions of the g and sg RNAs identified with high confidence are indicated with black arrows, those identified with lower confidence with gray arrows and their expected sizes in nucleotides are given on the left. The positions of RNA size markers indicated (see the RNA ladder itself in the uncropped image in Supplementary Figure 4).

To validate the reconstructed genome of LigMV and measure relative abundance of its genomic and subgenomic RNAs, we performed Northern blotting hybridization analysis of the total RNA samples used for Illumina sRNA-seq, as described in section “Materials and Methods”. By successive hybridization of the blot membrane with 31–34 nt DNA oligonucleotide probes complementary to the 3′-CR, 5′-parts of each gRNA and 3′-parts of gRNAs β and γ (Supplementary Table 1), followed by alignment of the scans (Supplementary Figure 4), we identified with high confidence the gRNAs α, β, and γ and sgRNAs β-b and γ-b of expected sizes, all sharing the 3′-CR (Figure 6, bands indicated with black arrows). The sgRNA-γ-b is the most abundant species, followed by gRNA-β, gRNA-γ, sgRNA-β-b, and gRNA-α (in that order of decreasing abundance). We also identified the sgRNA-β-dc and putative sgRNA-β-c of expected sizes, albeit with lower confidence due to their low abundance only slightly exceeding a background smear of degradation products of longer viral RNAs (Figure 6, gray arrows). Additional low abundance RNAs identified with sequence-specific probes are ∼180 and ∼280 nt species containing the 3′-CR sequence, a ∼280 nt species containing the 5′-part of γ-a ORF sequence, a ∼500 nt species containing the 3′-part of β-c ORF and the 3′-CR sequences, and a ∼500 nt species carrying the 3′-part of γ-b ORF and the 3′-CR sequences (Figure 6, gray arrows). Several distinct species which appear over a background smear of β-a ORF-specific hybridization below the gRNA-β band and range in size from 300 to 2500 nt (Figure 6) may represent gRNA-β derivatives produced by plant 3′–5′ exoribonuclease(s) terminating at stable secondary structures.

### Interaction of Ligustrum Mosaic Virus With the Plant RNA Interference Machinery

**FIGURE 7:**
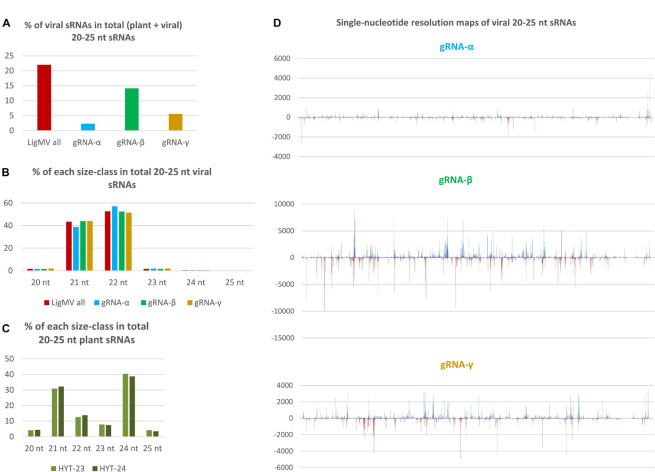
Illumina sequencing analysis of viral and host small RNAs from ligustrum mosaic virus (LigMV)-infected and healthy *Ligustrum vulgare* plants. Small RNA (sRNA) fractions of total RNA extracted from healthy (HYT-23) and LigMV-infected (HYT-24) leaf samples were converted to cDNA and Illumina sequenced. Then, 20–25 nt reads representing the majority in each library (Supplementary Dataset 2) were mapped on the reference sequences of LigMV gRNA segments α, β and γ with zero mismatches and counted. The bar graphs in **(A–C)** represent the percentage of viral genome-derived sRNA reads in total (plant + viral) 20–25 nt reads and their distribution between the three gRNA segments **(A)**, the relative abundance of each sRNA size-class in total 20–25 nt viral reads for the complete virus genome and each gRNA segment **(B)** and the relative abundance of each sRNA size in total 20–25 nt plant (non-viral) reads from the healthy and virus-infected plants **(C)**. **(D)** Shows single-nucleotide resolution maps of 20–25 nt viral sRNAs generated by MISIS-2 (Seguin et al., 2016) for each gRNA segment (see Supplementary Dataset 3 for each size-class). Forward and reverse strand-derived viral sRNAs are represented in blue and red, respectively.

Mapping of sRNA reads from the virus-infected privet leaf sample HYT-24 on the reconstructed LigMV genome revealed that viral sRNAs constitute 22% of total (plant + viral) 20–25 nt reads (Figure 7A). Only negligible amounts of viral sRNA reads were found in the non-symptomatic privet leaf sample HYT-23 (0.002% of total, likely representing cross-contamination during sRNA extraction or Illumina sequencing) (Supplementary Dataset 2).

Viral sRNAs were found to be predominantly 22 and 21 nucleotides in length, while other size-classes accumulate at very low levels (Figure 7B). This finding was validated by sRNA blot hybridization analysis (Supplementary Figure 5). Analysis of single-nucleotide resolution maps of viral sRNAs (Supplementary Dataset 3 and Figure 7D) revealed that the two major size-classes are derived from both strands of the entire virus genome and their hotspots are unequally distributed between the three gRNA segments with gRNA-β being the main source of viral sRNAs (14.1% of total), followed by gRNA-γ (5.6% of total) and gRNA-α (2.3% of total) (Figures 7A,D). Thus, relative abundance of viral sRNAs well correlates with relative abundance of gRNAs (Figure 6). Notably, sgRNA-γ-b being the most abundant viral RNA does not appear to be a source of more abundant viral sRNAs than the portion of gRNA-γ upstream of the sgRNA-γ transcription start site. This suggests that viral sRNAs are produced mainly from genomic and antigenomic RNA intermediates of gRNA replication and that sgRNA-γ-b does not appear to give rise to dsRNA precursors of sRNAs. Note that a prominent hotspot in the 3′-CR of gRNA-α likely represents viral sRNAs derived from the 3′-CRs of gRNAs β and γ that were randomly distributed between identical sequences of the three 3′-CRs by the mapping tool BWA. Taken together, the size, polarity, and hotspot profiles of LigMV-derived 21 and 22 nt sRNAs indicate that these are *bona fide* siRNAs produced by privet orthologs of Dicer-like (DCL) 4 and DCL2, respectively from longer double-stranded RNA intermediates of viral replication as established for other + ssRNA viruses in the model plant *Arabidopsis thaliana* and other plant species (reviewed by Pooggin, 2018). Analysis of 5′-nucleotide identities revealed that both 21 and 22 nt viral siRNAs are depleted in 5′-terminal G (7–12%) and have similar frequencies of 5′U, 5′A, and 5′C (Supplementary Dataset 2C), suggesting their preferential association with privet Argonaute (AGO) 1-, AGO2- and AGO5-like proteins as established for virus-derived siRNAs in Arabidopsis (Pooggin, 2018).

Interestingly, despite massive production of 21–22 nt viral siRNAs, the size profile of plant sRNAs dominated by 21, 22, and 24 nt classes was not affected by LigMV infection (Figure 7C).

### Geographic Distribution and Genetic Stability of Ligustrum Mosaic Virus Isolates

**FIGURE 8:**
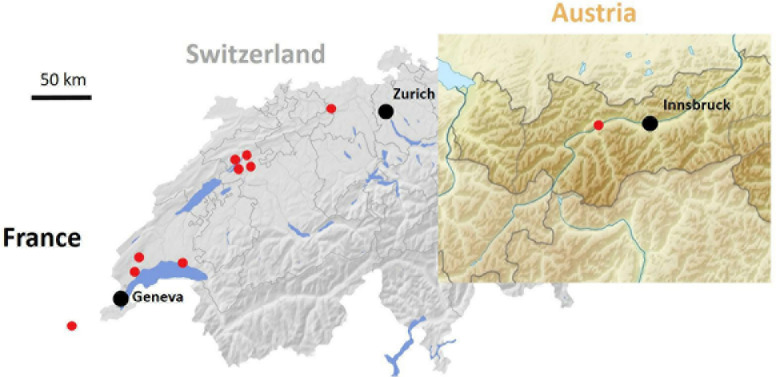
Geographic distribution of ligustrum mosaic virus (LigMV) in Switzerland and its neighboring countries. Red circles represent sites with at least one LigMV-infected *L. vulgare* plant.

Virus monitoring in wild ecosystems (forest edges) of Switzerland and its neighboring countries was undertaken to evaluate geographical distribution of LigMV. The virus was found to be widespread in Switzerland and was also spotted in Austria and France (Figure 8). Using RT-PCR diagnostics (see section “Materials and Methods”) we found a perfect association between yellow mosaic symptoms on the privet leaves and detection of the viral RNA, providing a strong evidence that LigMV is the disease causal agent. Indeed, LigMV was detected in all 18 symptomatic privet leaves, while none of the 25 symptomless privet leaves was RT-PCR positive. This was consistent with our bioinformatic analysis of Illumina sRNA reads that revealed no other virus but LigMV in the symptomatic privet leaves.

To evaluate the genetic stability of LigMV, a 1.9 Kb region of gRNA-γ encoding the viral RdRP was amplified by RT-PCR from 9 Swiss isolates and 1 Austrian isolate (Figure 8) and sequenced by Sanger technology. Only 16 SNPs were identified at the same positions in 2 or more isolates and 17 additional SNPs were identified only in single isolates (Supplementary Dataset 1C). Notably, the consensus sequence of these 10 isolates deviated only at 4 SNP positions from the Swiss isolate sequence reconstructed from sRNAs. Illumina sequencing of dsRNA preparation from one of the Swiss isolates, followed by *de novo* assembly (see section “Materials and Methods”), yielded an incomplete genome, lacking 3′-CRs and poly(A) tracts in all gRNAs and some other sequences (Supplementary Dataset 1A). Several SNPs and indels distinguished the dsRNA contigs from the sRNA consensus sequence: 9 SNPs in gRNA-α, 6 SNPs and 2 short indels (a 3 nt deletion and a 4 nt insertion in the sgRNA-β-b promoter/intergenic region) in gRNA-β, and 9 SNPs and one indel (a 1 nt insertion in the 5′-UTR) in gRNA-γ (Supplementary Dataset 1A). Excluding indels and missing sequences the two Swiss isolates share 99.5% (gRNA-α), 99.4% (gRNA-β), and 99.3% (gRNA-γ) identities.

To further evaluate the genome stability of LigMV over its geographic range we undertook the complete genome sequencing of one French and one Austrian isolate (Figure 8) using a virion-associated nucleic acid (VANA) sequencing approach (see section “Materials and Methods”). Illumina sequencing of VANA preps yielded 150,298 and 111,586 reads for the French and Austrian isolates, respectively. One quarter of those reads could be *de novo* assembled into contigs that represented the near-complete genomes of both isolates, lacking only 3′-CRs, but containing 5′-UTRs and 3′-terminal poly(A) tracts in each gRNA segment. Notably, 5′-UTRs of the French and Austrian isolates were somewhat longer than those confirmed by 5′-RACE in the Swiss isolate (except for the French gRNA-β contig lacking 23 nts at the 5′-terminus) and exhibited some SNP/indel deviations from the 5′-terminal sequences of the Swiss isolate gRNA β and γ (but not α) segments (Supplementary Dataset 1A). Excluding 3′-UTRs, the gRNA-α sequence contains 3 French isolate- and 7 Austrian isolate-specific SNPs and 2 SNPs common to both (French and Austrian) isolates, the gRNA-β sequence contains 5 French isolate- and 6 Austrian isolate-specific SNPs, 4 common SNPs and 2 common short indels (a 4 nt insertion and a 1 nt deletion in the sgRNA-β-b promoter region), and the gRNA-γ sequence contains 5 French isolate- and 2 Austrian isolate-specific SNPs, one French isolate-specific indel (a 1 nt insertion 14 nts downstream of the predicted sgRNA-β-b transcription start and 9 nts upstream of the authentic AUG start codon of γ-b ORF) and 7 common SNPs. Of the SNPs and indels shared by French and Austrian isolates, 9 SNPs and one indel (the 4 nt insertion in sgRNA-β-b promoter) are also shared with the Swiss isolate assembled from dsRNA reads (Supplementary Dataset 1A). Taken together, both French and Austrian isolates shared some sequence features that distinguished them from the Swiss isolates and some features that united them with the Swiss isolates. The pairwise comparison using STD v1.2 with MUSCLE (excluding indels) showed that the French, Austrian and Swiss isolates share more than 99% nucleotide identities, with the highest identity being in gRNA-α of the French and the Swiss sRNA isolate (99.8%), which indicates a genetic stability of LigMV in Switzerland and its neighboring countries.

### Vertical Transmission of Ligustrum Mosaic Virus

To assess seed transmission of LigMV, which may contribute to its genetic stability, mature fruits were collected from infected *L. vulgare* plants in the region of Nyon during autumn 2016 and 2018. In two germination experiments with cleaned seeds, a total of 439 plantlets were obtained and screened by RT-PCR for virus infection (see section “Materials and Methods”). Three plantlets were found to be infected by LigMV, indicating a seed transmission rate of ca. 0.7%.

## Discussion

We report here the identification and in-depth biological and molecular characterization of a new virus infecting non-cultivated *Ligustrum vulgare* in wild ecosystems, named ligustrum mosaic virus (LigMV). Based on the International Committee of Taxonomy of Viruses (ICTV)-species demarcation criterion based on complete genome sequence similarity, this 3-segmented + ssRNA virus should be considered as a first representative of a novel species in the genus *Hordeivirus* of family *Virgaviridae* (Adams et al., 2017; Savenkov, 2021), for which we propose a name *Hordeivirus ligustri* to conform a binomial nomenclature species format recently adopted by the ICTV (Walker et al., 2021).

Erwin Baur, who described for the first time a viral mosaic disease in *Ligustrum*, reported that the virus could not be seed-transmitted (Baur, 1908). However, given the low rate of seed transmission of LigMV in our study (0.7%), it is plausible that Baur did not observe any seed transmission events (he observed only 29 seedlings) even it would have been the same virus. The second author to report a viral mosaic in *Ligustrum* was Schmelzer (1963), who noted that it might be the same as Baur’s viral disease. The disease symptoms such as yellowish/white leaf blotches that he described are similar to those observed here with LigMV (Figures 1A–C). Thus, LigMV might be the virus responsible for viral mosaic described by Baur and Schmelzer in Germany, given the wide geographical distribution observed (i.e., France, Switzerland, Austria) and the symptoms similarities. However, we have no final proof for this hypothesis. A distinct + ssRNA virus of the genus *Idaeovirus* (unassigned to any family), privet leaf blotch-associated virus, was recently identified in southern Italy to be associated with the leaf blotch disease of *Ligustrum japonica*, which resembles infectious chlorosis described by E. Baur: the symptoms of this disease, however, differ from those observed in LigMV-infected privet plants in that they include characteristic “ring spots” (Navarro et al., 2017).

Besides *L. vulgare* that is a dicotyledonous plant in the *Oleaceae* family, other natural host plants of LigMV are not known yet. Phylogenetically, LigMV is most closely related to LRSV (Figure 4) that naturally infects lychnis and other wild dicotyledonous plants within *Caryophyllaceae* and *Labiatae* families, and more distantly related to PSLV and BSMV that infect wild and cultivated monocotyledonous *Poaceae* plants such as poa for PSLV and barley for BSMV. Hordeiviruses are known to be vertically transmitted through seeds or pollen, as well as mechanically by direct leaf contact under field conditions. Given a low seed transmission rate of LigMV in our experiments (0.7%), we cannot exclude a combination of vertical and horizontal transmissions of this virus. Indeed, we found that LigMV can be readily transmitted to the lab hosts *Chenopodium quinoa* and *Nicotiana benthamiana* via mechanical inoculation (Figures 1D,E). Similar to our findings, other hordeiviruses can also elicit local lesions in *Chenopodium* species and establish systemic infections in *N. benthamiana* (see ICTV report 2019, genus *Hordeivirus*).^8^

Based on electron microscopy examination, LigMV virions have a diameter of ∼22 nm and variable lengths with a majority being ∼100–150 nm (Figures 1F,G), which is similar to other hordeiviruses having the rod-shape particles of ∼20 × 100–150 nm in size. The length variation observed for LigMV particles could be due to differences in the lengths of three encapsidated gRNA segments (3.7, 3.1, and 2.7 Kb). Moreover, the shorter sgRNAs (1, 0.8, 0.7, 0.5, 0.3, and 0.2 Kb) detected by Northern (Figure 6) may also be encapsidated as demonstrated for BSMV sgRNA-γ-b (Stanley et al., 1984). Interestingly, virions of other hordeiviruses such as BSMV often form in purified preparations a range of hetero-disperse end-to-end aggregates up to 1,000 nm in length (Atabekov et al., 1968; see also the ICTV report 2019, genus *Hordeivirus*). Such elongated aggregates were also observed in the purified preparations of LigMV by Bovey (1976), being composed of particles of different lengths (Figure 1G, and data not shown).

### Genome Organization and Gene Expression Strategy of Ligustrum Mosaic Virus

Like other hordeiviruses, LigMV was found to be composed of three gRNA segments designated α, β, and γ, whose lengths (3.7, 3.1, and 2.7 Kb) are comparable to those of BSMV-ND18 (3.8, 3.2, and 2.8 Kb), LRSV (3.8, 3.0, and 2.7 Kb), and PSLV (3.8, 3.6, and 3.1 Kb), and to encode 7 evolutionarily-conserved viral proteins (Figure 3). By analogy with other hordeiviruses (Jackson et al., 2009) and based on our analysis, the gRNA-α is a monocistronic mRNA encoding a large protein with methyltransferase and helicase domains involved in viral replication. The gRNA-β encodes 4 proteins and serves as a monocistronic mRNA for the 5′-proximal CP (viral coat protein), while its complementary (minus) strand serves as a template for transcription of sgRNA-β-b (a monocistronic mRNA for TGBp1/helicase), sgRNA-β-dc (a dicistronic mRNA for TGBp2/movement protein and TGBp3), and a putative sgRNA-β-c (a monocistronic mRNA for TGBp3). The gRNA-γ encodes two proteins and serves as a monocistronic RNA for viral RdRP, while its complementary strand serves as a template for transcription of sgRNA-γ-b (a monocistronic mRNA for cysteine-rich protein/silencing suppressor).

In other hordeiviruses, each gRNA has a cap structure (m7GpppG…) at its 5’-end, and a conserved tRNA-like structure at the 3’-end. Our secondary structure modeling revealed that the tRNA-like structure of LigMV 3′-CR is remarkably similar to those predicted for PSLV, LSRV, and BSMV (Figure 2C and Supplementary Figure 2) which all resemble tRNA-Tyr and have a capability to accept tyrosine as demonstrated for BSMV and PSLV (Agranovsky et al., 1981, 1992; Loesch-Fries and Hall, 1982). The tRNA-like structure and preceding hairpin-like stem-loop structure(s) in LigMV and other hordeiviruses (Supplementary Figure 2) are likely involved in gRNA stabilization and replication (antigenomic RNA strand synthesis) as well as in formation of movement complexes and virions and in translation initiation on gRNAs and sgRNAs in coordination with the internal poly(A) tract (Jackson et al., 2009; see below). It is worth mentioning that sgRNA-γ-b from purified virions of BSMV was found to lack the tRNA-like structure and have a long poly(A) tail (up to 150 nts; Stanley et al., 1984). We found that in LigMV infected plants the sgRNA-γ-b containing the 3′-CR with tRNA-like structure is highly abundant (Figure 6). Whether or not this sgRNA is efficiently encapsidated remains to be investigated.

In BSMV, the 5′-UTRs of gRNA segments are necessary for gRNA replication and apparently contain recognition signals for (+)strand RNA synthesis, but they display little similarity in primary sequences apart from their 5′-terminal sequence following the cap (m7GpppGUA; Zhou and Jackson, 1996a), which is conserved in most viruses of the family *Virgaviridae* (Savenkov, 2021). On the other hand, if compared for each gRNA separately the 5′-UTRs of LRSV, PSLV, and BSMV share more similarity. These findings have led to the hypothesis that dissimilar template recognition signals in the 5′-UTRs of gRNA-α, gRNA-β, and gRNA-γ regulate relative accumulation of these gRNA segments (Savenkov et al., 1998). Contrary to the above findings, we found that the 5′-UTRs of LigMV gRNAs share the 5′-terminal GUAA element, 3 conserved motifs in the internal sequence and the identical initiation context of AUG start codons (Figure 2C). Moreover, the 5′-UTRs of LigMV, LRSV, PSLV, and BSMV do not share any striking common features, although the 5′UTRs of LigMV were more similar to those of LRSV (Supplementary Dataset 1B). Thus, it remains to be investigated whether and how the (+)strand synthesis of gRNA segment is regulated and coordinated by the 5′-UTR primary sequence motifs or secondary structure elements.

Regarding regulation of translation initiation on LigMV gRNAs, three features of their 5′-UTRs—(i) absence of internal AUGs, (ii) relatively short length, and (iii) strong initiation context of AUG start codons (adenosine at position −3 and guanosine at position + 4)—are consistent with the 5′-cap-dependent linear ribosome scanning mechanism operating on most eukaryotic mRNAs. Similar to other hordeiviruses, the sgRNA-β-dc of LigMV can potentially serve as a bicistronic mRNA for translation of TGBp2 and TGBp3 via a leaky scanning mechanism as the region between the AUG start codons of the two overlapping ORFs contains no other AUG and the AUG of the first ORF is in a poor initiation context (with cytosines at both −3 and + 4 positions), which would allow for its bypassing by a fraction of scanning ribosomes. At the same time, our Northern blot and sgRNA promoter sequence analyses (Figures 5D, 6) provide preliminary evidence that TGBp3 may also be translated from a putative monocistronic sgRNA-β-c.

Translation of most plant and viral mRNAs possessing long poly(A) tails is facilitated by poly(A)-binding protein (PABP) that interacts with the translation initiation complex bound to the 5′-cap. We found that similar to other (but not all) hordeiviruses (Agranovsky et al., 1978, 1983; Jackson et al., 2009), LigMV genomic and subgenomic RNAs possess an internal poly(A) tract in their 3′-UTRs. Besides hordeiviruses, 3′-UTRs of most bromoviruses and some tobamoviruses possess internal poly(A) tracts. In the tomabovirus hibiscus latent Singapore virus, the internal poly(A) (77–96 nts in length) separating the CP ORF and the structured region with 3′-tRNA-like structure has been implicated in initiation of translation and control of mRNA decay through interaction with PABP: the minimal length of poly(A) required for PABP binding and viral CP expression was 24 nts (Niu et al., 2015). This is consistent with findings in yeasts that PABP binding density is one molecule for every 25 nts of poly(A) (Sachs et al., 1987). Our RT-PCR sequencing data (Supplementary Figure 1) revealed a variable length of the internal poly(A) tracts in LigMV RNAs, confirming previous findings for other hordeiviruses and other genera of + ssRNA viruses. Notably, the poly(A) tracts of LigMV (∼53–71 nts in each gRNA) are longer than those of LRSV (44–48 nts in α, 39–43 nts in β, 28–62 nts in γ) and BSMV (15–50 nts in α, 17–42 nts in β, 15–39 nts in γ), suggesting that LigMV has evolved toward better interaction with PABP than other hordeiviruses. In contrast, PSLV has evolved to replace a long poly(A) tract with a short A-rich sequence and a 4-hairpin domain preceding the conserved structural configuration (Solovyev et al., 1996; Supplementary Figure 2A). Likewise, BSMV has evolved to reduce the length of poly(A) and, in compensation, acquired a 2-hairpin domain (Kozlov et al., 1984; Supplementary Figure 2B). The roles of the hairpin domains fully or partially replacing a long poly(A) tract in PSLV and BSMV and the elongated hairpin present in all hordeiviruses including LigMV (Figure 2C and Supplementary Figure 2) remain to be investigated. In tomaboviruses, a conserved upstream pseudoknot domain together with the 3′-terminal tRNA-like structure have been implicated in both translation and replication of viral gRNA (reviewed in Chujo et al., 2015).

Northern blot hybridization analysis allowed us not only to identify LigMV gRNAs and sgRNAs but also measure their relative abundance. Notably, sgRNA-γ-b accumulated at much higher levels than gRNAs and other sgRNAs taken together (Figure 6), which may reflect an important function of the 16 kDa cysteine-rich protein γ-b at a given stage of infection in the symptomatic privet leaf. Indeed, the hordeiviral γ-b proteins and other small cysteine-rich proteins of + ssRNA viruses have been implicated in RNA binding and suppression of RNA silencing (Yelina et al., 2002; Bragg and Jackson, 2004; Li et al., 2020). In a previous study also using Northern blot analysis with a 3′-CR specific probe, sgRNA-γ was found to be the most abundant viral RNA in LRSV-infected *N. benthamiana* (Jiang et al., 2018). Moreover, the relative abundance of LRSV genomic and subgenomic RNAs appeared to be comparable to that of LigMV in *L. vulgare*, although Jiang and co-authors could only identify three gRNAs and sgRNA-γ but not the other sgRNAs. Furthermore, our analysis showed that sgRNA-β-b (mRNA for TGBp1) and sgRNA-β-dc (mRNA for TGBp2 and TGBp3) accumulate at lower levels than gRNAs, with sgRNA-β-dc being barely detectable (Figure 6). This is consistent with the previous findings in BSMV-infected plants and protoplasts, where sgRNA-β-dc accumulated at very low levels, much lower than sgRNA-β-b (Zhou and Jackson, 1996b and references therein). In protoplasts their expression was found to be temporal, while sgRNA-γ was expressed constitutively at relatively high levels (Zhou and Jackson, 1996b; Johnson et al., 2003). Hence, LigMV and BSMV (and possibly other hordeiviruses) have evolved similar strategies for expression of the triple gene block proteins by regulating relative abundance of the two sgRNAs likely at the transcriptional level. Consistent with the latter hypothesis the sgRNA promoter regions of these two sgRNAs in LigMV, BSMV, LRSV, and PSLV have distinct common elements (Figure 5). It is puzzling, however, that the promoter region of sgRNA-γ-b, the most abundant viral RNA, shares more commonalities with the promoter of the least abundant sgRNA-β-dc than with the promoter of sgRNA-β-b. Thus, mechanisms regulating transcription, stability and translation of hordeiviral sgRNAs at different stages of replication in single cells and systemic infection remain to be further investigated.

Previously it has been noted that sgRNA promoters and other cis-acting elements of BSMV and PSLV are more closely related to each other than to those of LRSV (Jackson et al., 2009). This is consistent with the biological evidence suggesting closer relatedness of BSMV and PSLV to each other than to LRSV (Hunter et al., 1989). Our comparative sequence analysis shows that LigMV is most closely related to LRSV (Figure 4), although some of the elements of its primary sequence and secondary structures are more related to those of PSLV and/or BSMV and some core elements are universally conserved (Figures 2, 5 and Supplementary Dataset 1B). Thus, all four hordeiviruses have evolved from a common ancestor by preserving the core sequence elements and adapting other *cis*-acting elements to co-evolving host plants (monocots for BSMV and PSLV and dicots for LRSV and LigMV).

### Interaction of Ligustrum Mosaic Virus With the Plant RNA Interference

We found that LigMV-infected privet leaf tissues accumulated highly abundant viral siRNAs (22% of total sRNAs), indicating that the virus triggers a strong RNAi response. Based on the sRNA size and hotspot profiling (Figure 7) and the knowledge of viral siRNA biogenesis in land plants (Pooggin, 2018), LigMV appears to be targeted mainly by privet homologs of DCL2 and DCL4 that process double-stranded intermediates of viral gRNA replication into 22 and 21 nt siRNAs, respectively. The 5′-terminal nucleotide identity profile of LigMV siRNAs shows that they are likely sorted and stabilized by privet homologs of AGO1 (5′U), AGO2 (5′A), and AGO5 (5′C) proteins. The fact that the highly abundant sgRNA-γ was not a major source of viral siRNAs indicates that the host RNA-dependent RNA polymerase (RDR) activity may not contribute in a major way to production of double-stranded RNA substrates for Dicers. Since DCL4 is known to be a primary Dicer targeting cytoplasmic RNA viruses, while DCL2 is activated when DCL4 activity is diminished or suppressed (reviewed in Csorba et al., 2015; Pooggin, 2016, 2018), the predominance of 22 nt viral siRNAs, which accumulated at levels exceeding those of 21 nt viral siRNAs (Figure 7B), suggests that DCL4 activity in LigMV-infected privet cells might be suppressed by a viral silencing suppressor protein. As mentioned above, the γ-b protein translated from the most abundant sgRNA is a silencing suppressor (Yelina et al., 2002; Bragg and Jackson, 2004; Li et al., 2020) that can potentially target DCL4 and perhaps other components of the plant RNAi machinery, as demonstrated for other viral suppressor proteins (reviewed in Csorba et al., 2015; Pooggin, 2016). However, viral suppressor activity does not appear to prevent massive production of viral 21–22 nt siRNAs. Interestingly, a previous sRNA sequencing analysis of *Ligustrum japonica* infected by privet leaf blotch-associated virus (+ ssRNA virus of the genus *Idaeovirus*) has revealed predominantly 21 and 22 nt siRNAs derived from both strands of the viral genome, which accumulated at very low levels in the infected leaves (0.6% of total sRNAs) (Navarro et al., 2017).

### Genetic Stability of Ligustrum Mosaic Virus and Other Hordeiviruses

Analysis of the complete or near-complete genome sequences from the Swiss, French and Austrian isolates obtained in our study revealed a genetic stability of LigMV across a wide geographic region (>99% pairwise identities). Such genetic stability of LigMV can be explained by seed (and possibly mechanical) transmission in its natural host, similar to other hordeiviruses that lack a vector and rarely jump host plant species. Smith et al. (2014) have reported identification and reference-based reconstruction of an ancient isolate of BSMV by sequencing sRNAs extracted from a 750 years-old seed of barley, with 99.4% of the contemporary virus reference genome being covered by sRNA contigs. Using this ancient genome sequence, the rate of molecular evolution of BSMV was estimated to be 3.9 × 10^–5^ substitutions per site per year, which is much slower than estimated from the extant strains of BSMV (Smith et al., 2014). Besides being indicative of genetic stability of BSMV in its barley host over a time span of 750 years, the Smith et al. (2014), study confirms that deep sRNA sequencing and bioinformatics is a powerful approach for virus identification and genome reconstruction as established for all land plants (Pooggin, 2018). Our work further showed that a complete 3-segmented hordeivirus genome can be *de novo* assembled from sRNA sequencing reads, even without a filtering step through the host genome (unavailable for *L. vulgare*), which can improve viral genome reconstruction (Seguin et al., 2014). This is likely due to massive production of viral siRNAs and their stabilization by the host AGO proteins.

## Conclusion

In this study, we have used deep small RNA sequencing and bioinformatics to identify a causative agent of yellow mosaic disease of the wild dicotyledonous plant *Ligustrum vulgare*, observed in Europe for more than 100 years, and to reconstruct its complete 3-segmented RNA genome. Our phylogenomic analysis shows that this virus, named ligustrum mosaic virus (LigMV), is a member of a novel species in the genus *Hordeivirus* (family *Virgaviridae*), for which we will propose the name *Hordeivirus ligustri* in an official taxonomic proposal to the ICTV following publication of this paper. LigMV is more related to the hordeivirus LRSV infecting wild dicotyledonous plants than to the hordeiviruses PSLV and BSMV infecting wild and cultivated monocotyledonous plants, respectively. Similar to other hordeiviruses, LigMV forms rod-shape virions and is transmitted through seeds. Genomic and subgenomic RNAs revealed by Northern blotting hybridization can potentially serve as mRNAs for seven evolutionarily conserved viral proteins. Notably, the 3′-common region sequence attached to each genomic and subgenomic RNA via a 57–71 nucleotide poly(A) tract can assume a conserved tRNA-like structure with two stem-loop elements resembling respective elements of LRSV or PSLV, and pseudoknot’s central and 3′-terminal helices near identical to those of all three (LRSV, PSLV, BSMV) hordeiviruses. Analysis of size, polarity, and hotspot profiles of viral small RNAs in comparison with relative abundance of genomic and subgenomic RNAs suggests that double-stranded intermediates of viral genomic RNA replication are targeted by the plant antiviral Dicers DCL2 and DCL4 generating, respectively, 22 and 21 nt viral siRNAs. Whole genome sequencing of Swiss, French and Austrian isolates of LigMV shows its genetic stability over a wide geographic range, suggesting its persistence and spread in Europe via seed dispersal. LigMV is the fourth virus species of genus *Hordeivirus* with completely sequenced genome, which allows for better characterization of the molecular evolution and biology of this genus and other genera of the family *Virgaviridae*.

## Data Availability Statement

The datasets presented in this study can be found in online repositories. The names of the repository/repositories and accession number(s) can be found below: https://www.ncbi.nlm.nih.gov/genbank/, MW752157, MW752158, and MW752159.

## Author Contributions

J-SR, PG, and MP conceived and designed the study. J-SR, ST, JB, IK, and PG performed the experiments. ST, J-SR, FM, and MP analyzed bioinformatically and interpreted the sequencing data. MP and J-SR wrote the draft manuscript and prepared the figures and the Supplementary Files. All the authors corrected and approved the final manuscript for submission.

## Conflict of Interest

The authors declare that the research was conducted in the absence of any commercial or financial relationships that could be construed as a potential conflict of interest.

## Publisher’s Note

All claims expressed in this article are solely those of the authors and do not necessarily represent those of their affiliated organizations, or those of the publisher, the editors and the reviewers. Any product that may be evaluated in this article, or claim that may be made by its manufacturer, is not guaranteed or endorsed by the publisher.

## Footnotes

1
www.fasteris.com

2
https://www.ebi.ac.uk/~zerbino/velvet/

3
https://www.ebi.ac.uk/~zerbino/oases/

4
www.geneious.com

5
https://blast.ncbi.nlm.nih.gov/Blast.cgi

6
http://web.cbio.uct.ac.za/~brejnev/

7
https://mafft.cbrc.jp/alignment/server/

8
https://talk.ictvonline.org/ictv-reports/ictv_online_report/positive-sense-rna-viruses/w/virgaviridae/669/genus-hordeivirus

## Dedication

This article is dedicated to the memory of Dr. René Bovey (1919–2018), a great scientist who started this virological study more than 50 years ago.

## Supplementary Material

Supplementary Figure 1 RT-PCR sequencing of the 3′-terminal regions of LigMV gRNAs containing internal poly(A) tracts. **(A)** PCR design and agarose gel electrophoresis analysis of the PCR products. **(B–D)** Sequences of the PCR products representing 3′-terminal regions of LigMV gRNA-α (S1B), gRNA-β **(C)**, and gRNA-γ **(D)**.

Supplementary Figure 2 Predicted secondary structures in 3′-common regions (CRs) of LigMV, PSLV, LRSV, and BSMV. For description, see the text and the caption of Figure 2.

Supplementary Figure 3 MISIS-generated maps of small RNAs derived from 5′-UTRs and 3′-CRs of LigMV genomic RNAs α, β, and γ. The 20–25 nucleotide (nt) sRNAs from LigMV-infected plant (HYT-24) were mapped to the reconstructed consensus reference sequences of LigMV genomic (g) RNAs alpha (gRNA-α), beta (gRNA-β), and gamma (gRNA-γ) using BWA and the resulting BAM files were analyzed by MISIS-2 (Seguin et al., 2016). The sRNA maps were visualized zooming in the 5′-terminal (left) and 3′-terminal (right) regions of each gRNA: print screen images of the MISIS-2 maps are shown.

Supplementary Figure 4 Northern blotting hybridization analysis of LigMV genomic and subgenomic RNAs: Alignment of the gel and the hybridization scans. For description, see the text and the caption of Figure 6.

Supplementary Figure 5 Blot hybridization analysis of LigMV-derived small interfering RNAs. Total RNA extracted from LigMV-infected (+) and healthy (−) *L. vulgare* leaf tissues was separated on 15% polyacrylamid-urea gel and blotted to a nylon membrane, followed by UV cross-linking. The membrane was successively hybridized with P32-lablelled DNA oligonucleotide probes specific to LigMV 3′-CR and gRNA-beta (Supplementary Table 1). Positions of 21- and 22-nt viral siRNAs are indicated with arrows. EtBr-stained gel is shown as loading control.

Supplementary Table 1 DNA oligonucleotides used as primers for RT-PCR and 5′- and 3′-RACE analyses, and as probes for Northern and sRNA blot hybridization analyses.

Supplementary Dataset 1 Nucleotide sequences and multiple alignments of LigMV isolates and 5′-UTRs of LigMV genomic and subgenomic RNAs in comparison with other hordeiviruses. **(A)** Nucleotide sequences of Swiss, French and Austrian isolates of LigMV and their multiple alignments. **(B)** Comparison of 5′-UTRs of LigMV, LRSV, PSLV, and BSMV. **(C)** Nucleotide sequences of 1.9 Kb gRNA-γ region amplified by RT-PCR from 10 LigMV isolates and their multiple alignment comparison with complete and near complete genome sequences of Swiss (CH), French (FR) and Austrian (AUT) isolates obtained by Illumina sequencing (sRNA-seq, dsRNA-seq and VANA-seq).

Supplementary Dataset 2 Counts of non-viral and virus-derived sRNAs from LigMV-infected (HYT-24) and healthy (HYT-23) *Ligustrum vulgare* plants. The sRNA libraries HYT-23 and HYT-24 were mapped to the LigMV gRNA α, β, and γ reference sequences and counted by size (20, 21, 22, 23, 24, 25 nt, total 20–25 nt), polarity (forward, reverse, total) and 5′-nucleotide identity (5′A, 5′C, 5′G, 5′U). The counts are presented in three tables: two with counts of reads mapped to the viral reference sequences with zero **(A)** or up to 2 **(B)** mismatches, and one table with 5′-nucleotide frequencies of viral sRNAs mapped to the viral references with zero mismatches **(C)**.

Supplementary Dataset 3 MISIS-generated, single-nucleotide resolution maps of 20–25 nt viral sRNAs in ligustrum mosaic virus (LigMV)-infected *Ligustrum vulgare*. The 20–25 nucleotide (nt) sRNAs from LigMV-infected plant (HYT-24) were mapped to the reconstructed consensus reference sequences of LigMV genomic (g) RNAs alpha **(A)**, beta **(B)**, and gamma **(C)** using BWA and the resulting BAM files were analyzed by MISIS-2 (Seguin et al., 2016) to generate count tables of reads mapped to each reference sequence with zero mismatches and sorted by size and polarity. In each count table, the first column gives nucleotide positions of the corresponding genomic RNA sequence. In the next columns, the positions of 5’-terminal nucleotide of sense sRNAs and 3’-terminal nucleotide of antisense siRNAs along the reference sequence are indicated, and the read counts are given for each sRNA of 20-, 21-, 22-, 23-, 24-, and 25-nt classes mapped with zero mismatches to the forward strand (columns 20, 21, 22, 23, 24, 25) and the reverse strand (columns 20_rev, 21_rev, 22_rev, 23_rev, 24_rev, 25_rev), along with the total counts of 20–25 nt sRNAs mapped on the forward (fwd_total) and reverse (rev_total) strands and on both strands (total). Histograms of the maps of total reads and those of each size-class are inserted on the right.
