# Supplementary material for: Identification and Molecular Characterization of a Novel Hordeivirus Associated With Yellow Mosaic Disease of Privet (Ligustrum vulgare) in Europe
Source: Front Microbiol. 2021 Sep 27;12:723350. doi: 10.3389/fmicb.2021.723350 (PMC8503643; doi:10.3389/fmicb.2021.723350)

Figure S1. RT-PCR sequencing of the 3’-terminal regions of LigMV gRNAs containing internal poly(A) tracts. (**S1A**) PCR design and agarose gel electrophoresis analysis of the PCR products; (**S1B-D**) Sequences of the PCR products representing 3’-terminal regions of LigMV gRNA-α (**S1B**), gRNA-β (**S1C**) and gRNA-γ (**S1D**).

Figure S1A. PCR design and agarose gel electrophoresis analysis of the PCR products.

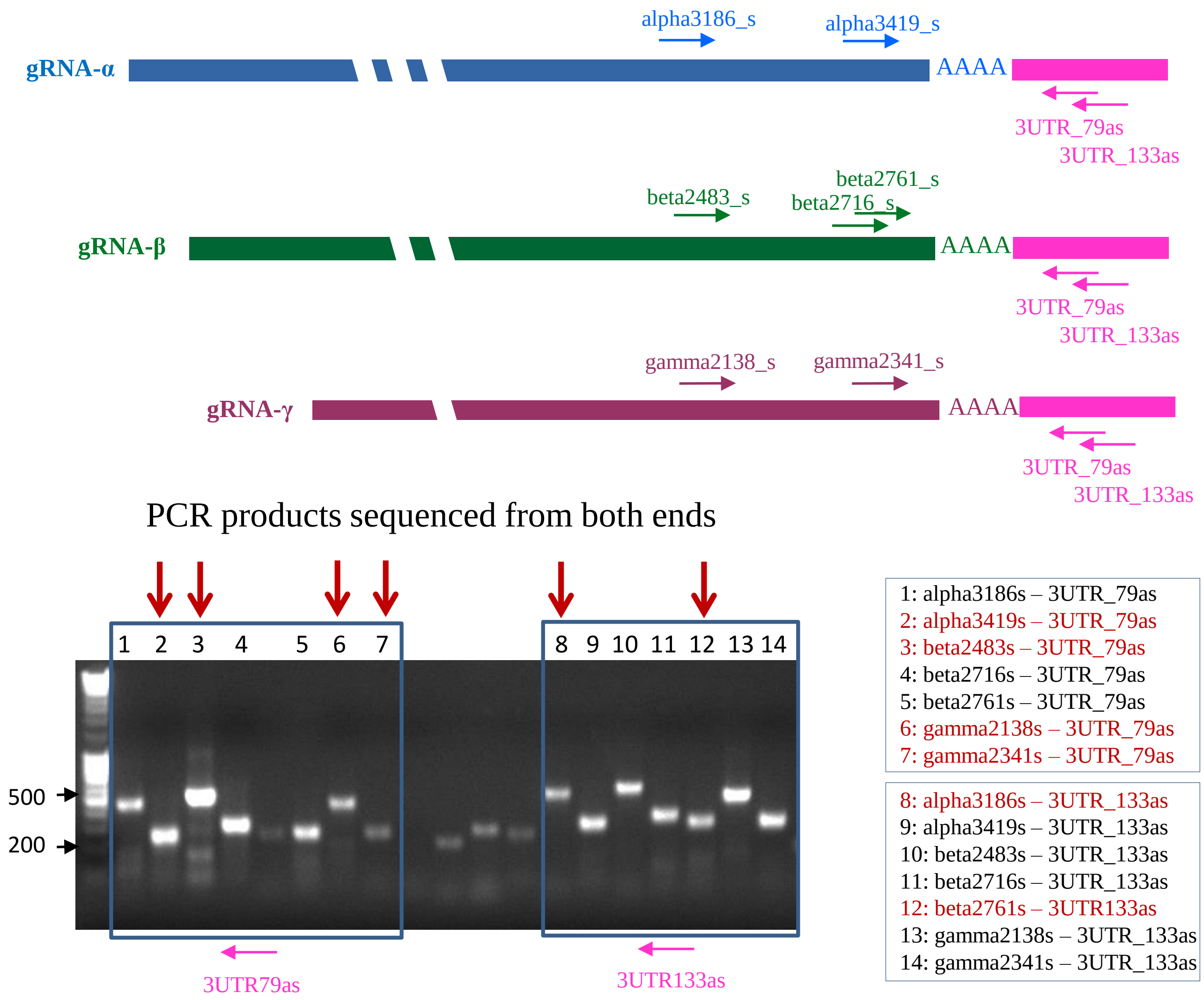

Figure S1B. Sequences of the PCR products representing 3’-terminal region of LigMV gRNA-α.

PCR product N1 (a3419s-3UTR79as)**:** 8T-**58A** (sRNA **contig** ends with ...GTTTT , lacking ... TCTTTTTTTTAAAA...)

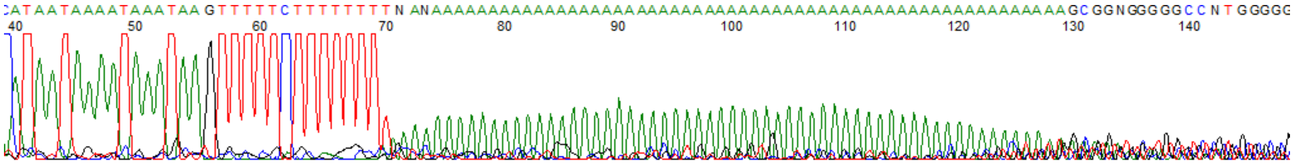

PCR product N1 (a3419s-3UTR79as): **62A** (sRNA **contig** starts with 17A-TGCC...)

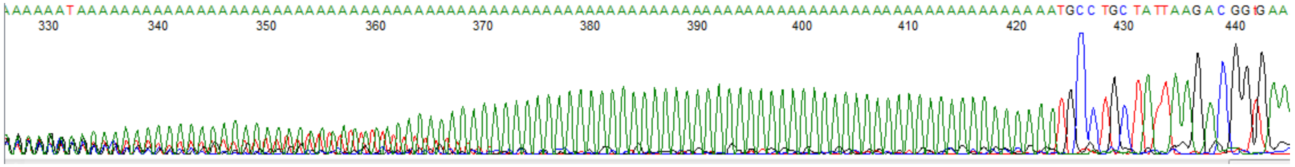

PCR product N2 (a3186s-UTR133as): 7T-**60A** (sRNA **contig** ends at 3511 with ...GTTTT , lacking ... TCTTTTTTTAAAA...)

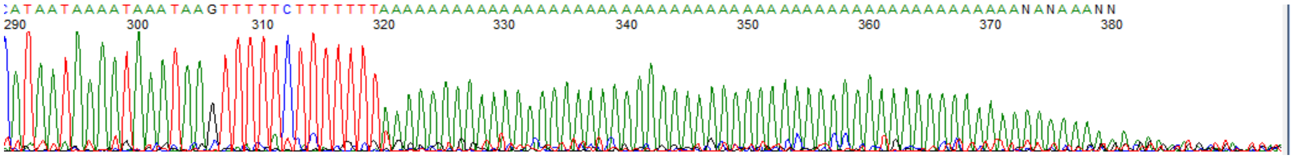

PCR product N2 (a3186s-UTR133as): **58A** (sRNA **contig** starts with 17A-TGCC...)

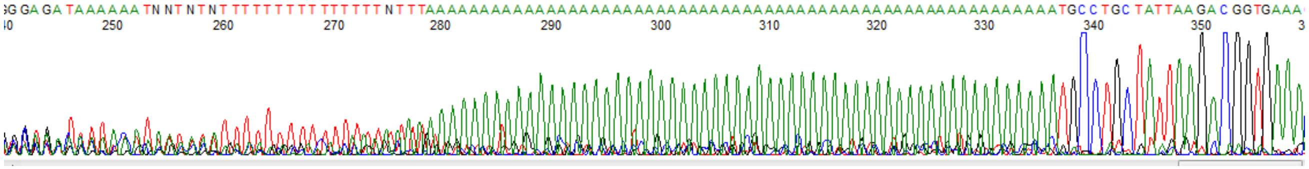

Cloned PCR product: M13 reverse read: **53A** (sRNA **contig** starts with 17A-TGCC...)

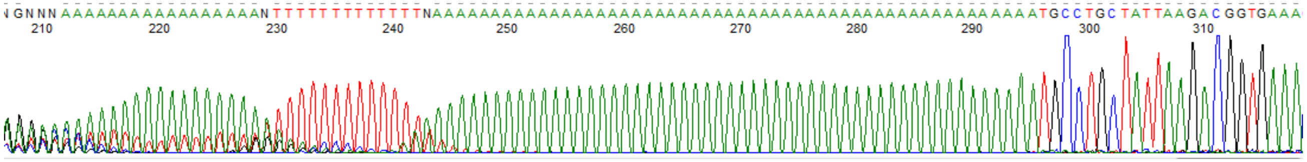

Figure S1C. Sequences of the PCR products representing 3'-terminal region of LigMV gRNA-β.

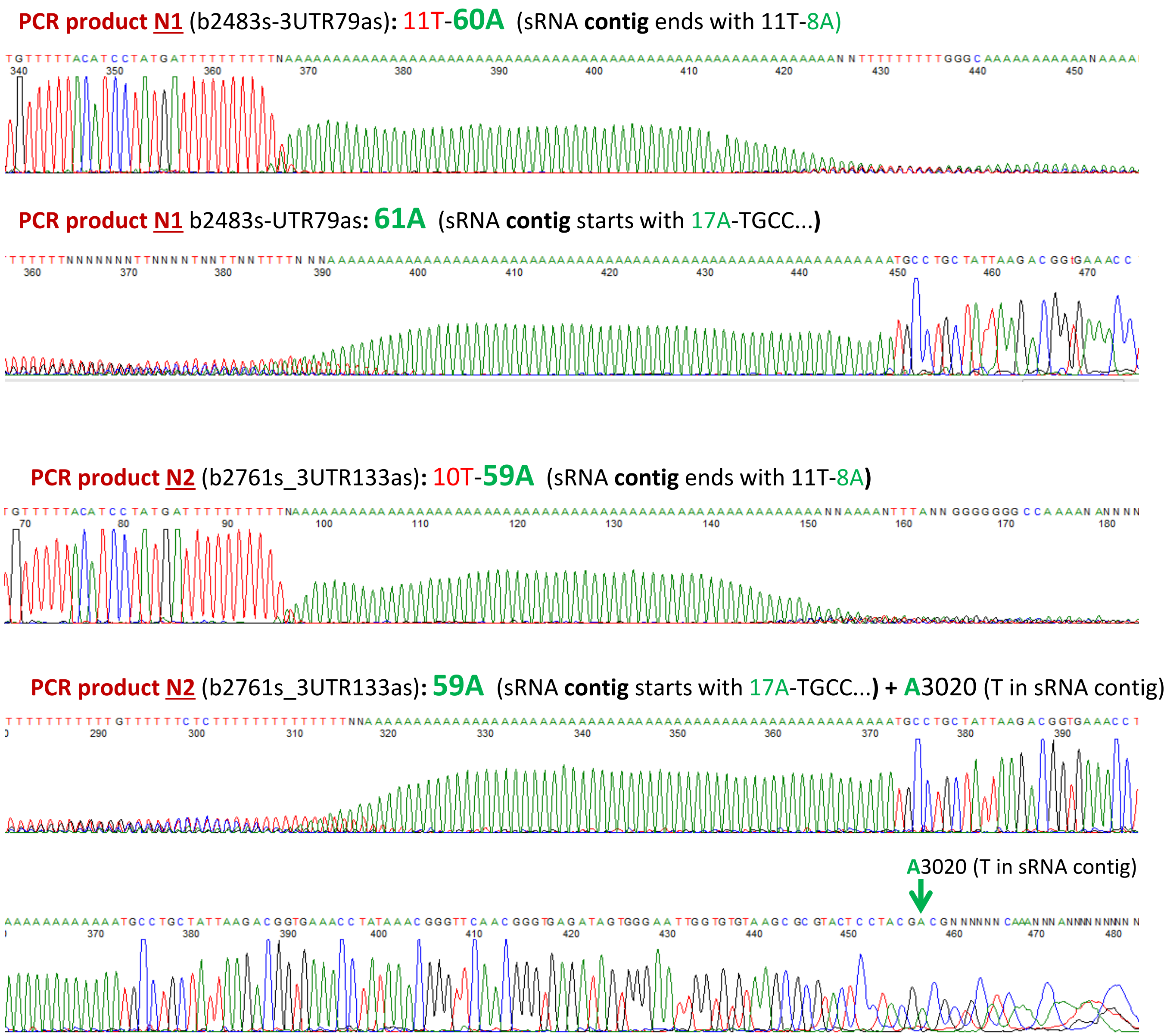

Figure S1C. Sequences of the PCR products representing 3'-terminal region of LigMV gRNA-β. (cont.)

Cloned PCR product N1 ; M13 forward read; 10T-**60A** (sRNA **contig**: ...ATGA-11T-8A)

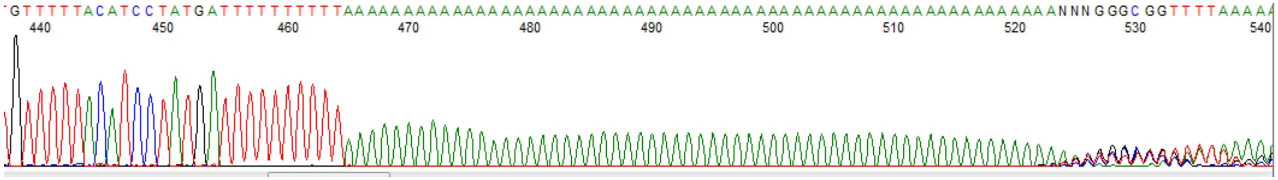

Cloned PCR product N2 ; M13 forward read; 10T-**61A**

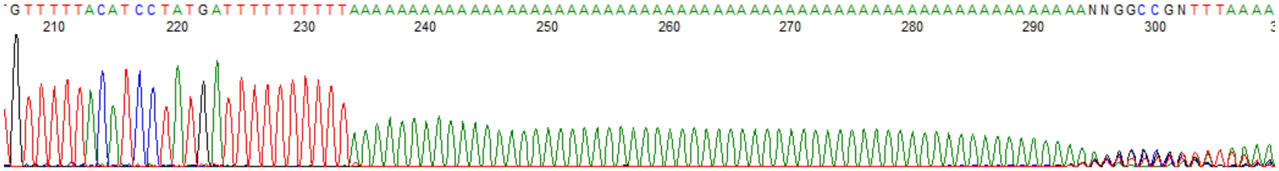

Figure S1D. Sequences of the PCR products representing 3'-terminal region of LigMV gRNA-γ.

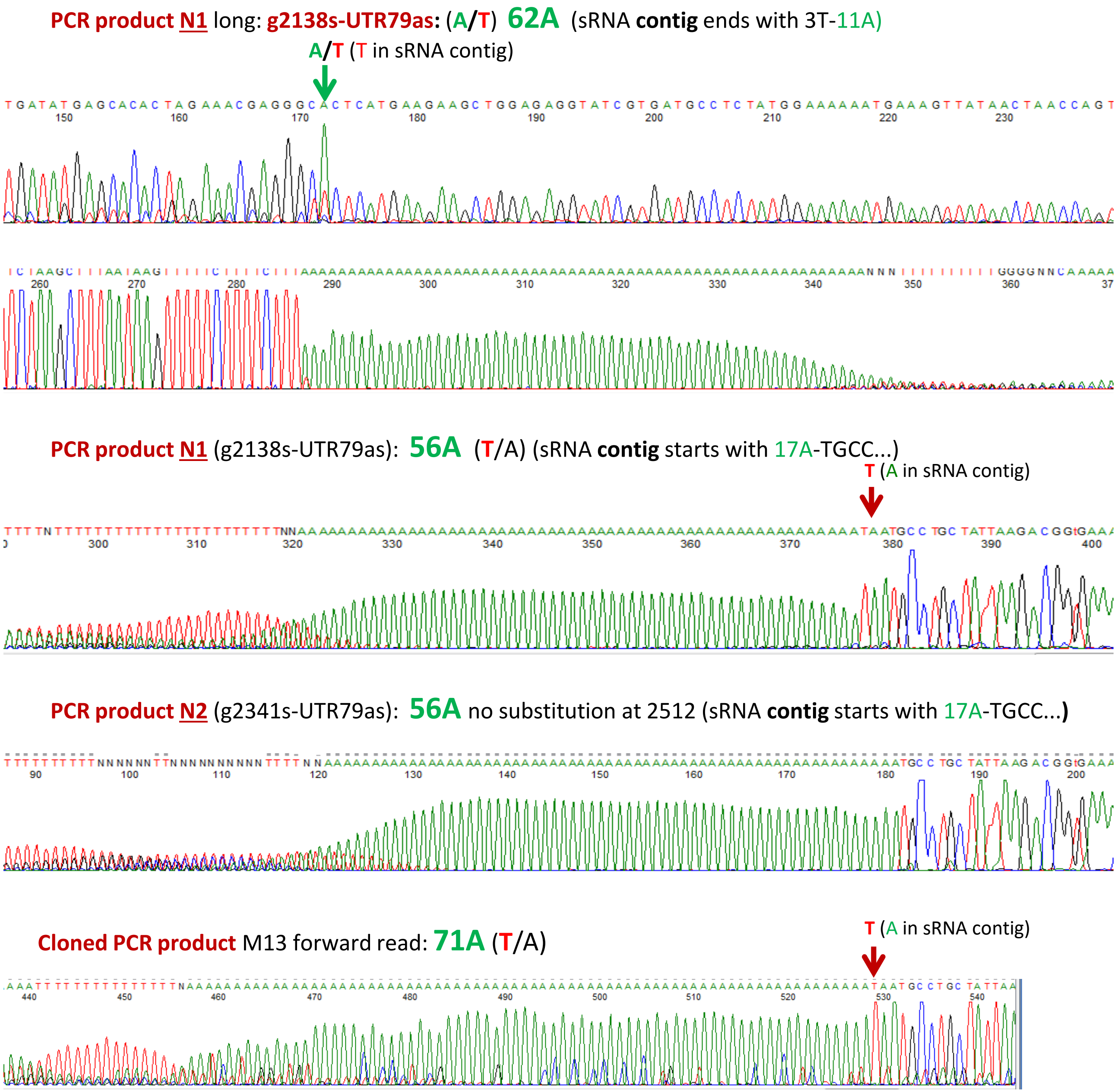

Supplement: Supplementary Figure 1 — RT-PCR sequencing of the 3′-terminal regions of LigMV gRNAs containing internal poly(A) tracts. (A) PCR design and agarose gel electrophoresis analysis of the PCR products. (B–D) Sequences of the PCR products representing 3′-terminal regions of LigMV gRNA-α (S1B), gRNA-β (C), and gRNA-γ (D). [file Image_1.pdf]
