# Supplementary material for: Identification and Molecular Characterization of a Novel Hordeivirus Associated With Yellow Mosaic Disease of Privet (Ligustrum vulgare) in Europe
Source: Front Microbiol. 2021 Sep 27;12:723350. doi: 10.3389/fmicb.2021.723350 (PMC8503643; doi:10.3389/fmicb.2021.723350)

Figure S2. Predicted secondary structures in 3'-common regions (CRs) of LigMV, PSLV, LRSV and BSMV. For description, see the text and the caption of Figure 2.

A PSLV and LRSV (Solovyev et al. 1996) LigMV (this study)

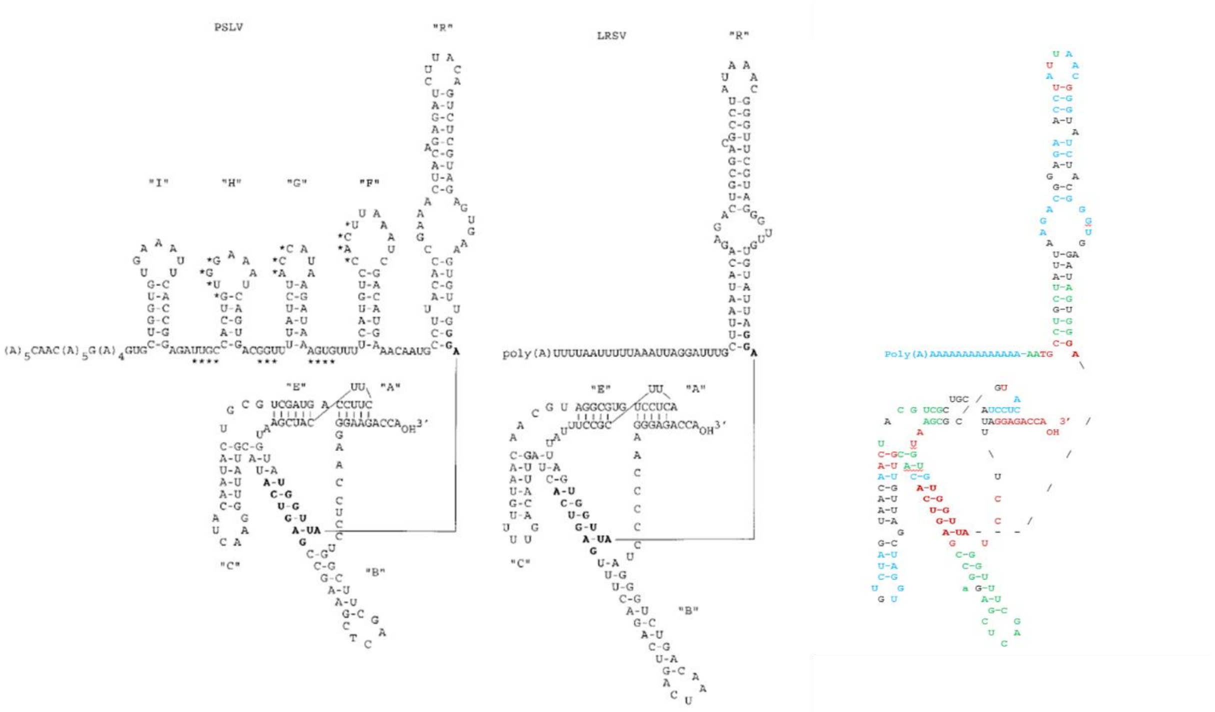

B BSMV (Kozlov et al. 1984)

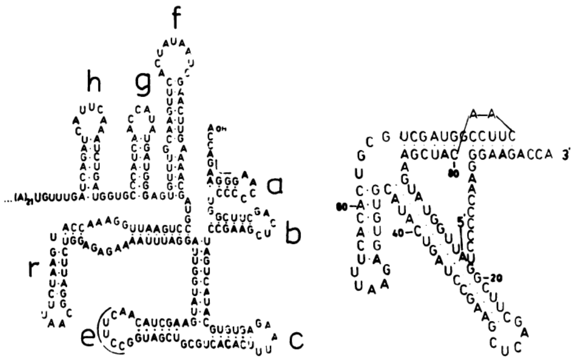

Supplement: Supplementary Figure 2 — Predicted secondary structures in 3′-common regions (CRs) of LigMV, PSLV, LRSV, and BSMV. For description, see the text and the caption of Figure 2. [file Image_2.pdf]
