# Supplementary material for: Identification and Molecular Characterization of a Novel Hordeivirus Associated With Yellow Mosaic Disease of Privet (Ligustrum vulgare) in Europe
Source: Front Microbiol. 2021 Sep 27;12:723350. doi: 10.3389/fmicb.2021.723350 (PMC8503643; doi:10.3389/fmicb.2021.723350)

**Figure S3. MISIS-generated maps of small RNAs derived from 5’-UTRs and 3’-CRs of LigMV genomic RNAs α, β and γ.** The 20-25 nucleotide sRNAs from LigMV-infected plant (HYT-24) were mapped to the reconstructed consensus reference sequences of LigMV genomic (g) RNAs alpha (gRNA-α), beta (gRNA-β) and gamma (gRNA-γ) using BWA and the resulting BAM files were analysed by MISIS-2 (Seguin et al. 2016). The sRNA maps were visualized zooming in the 5´-terminal (left) and 3´-terminal (right) regions of each gRNA: print screen images of the MISIS-2 maps are shown.

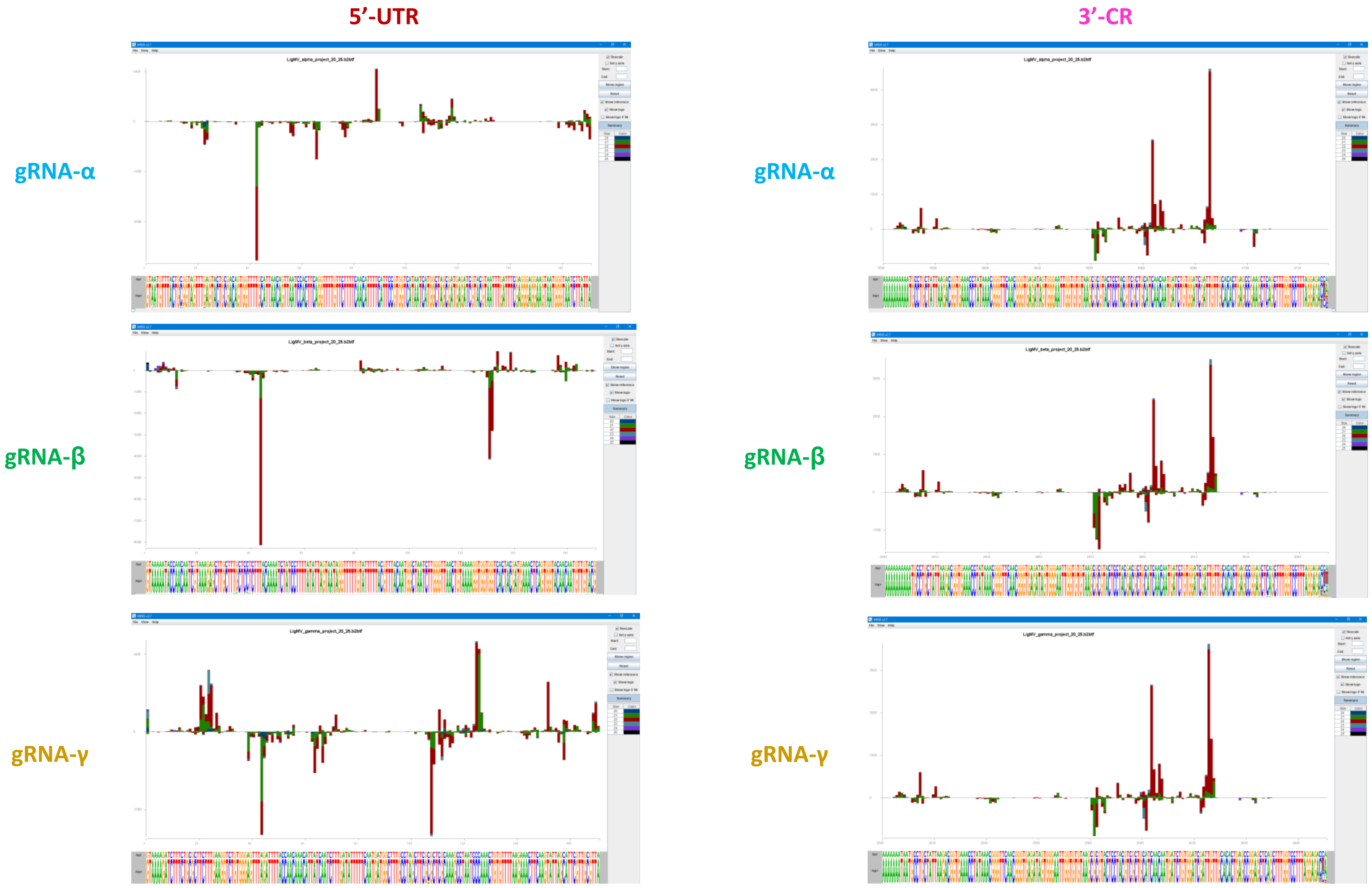

Supplement: Supplementary Figure 3 — MISIS-generated maps of small RNAs derived from 5′-UTRs and 3′-CRs of LigMV genomic RNAs α, β, and γ. The 20–25 nucleotide (nt) sRNAs from LigMV-infected plant (HYT-24) were mapped to the reconstructed consensus reference sequences of LigMV genomic (g) RNAs alpha (gRNA-α), beta (gRNA-β), and gamma (gRNA-γ) using BWA and the resulting BAM files were analyzed by MISIS-2 (Seguin et al., 2016). The sRNA maps were visualized zooming in the 5′-terminal (left) and 3′-terminal (right) regions of each gRNA: print screen images of the MISIS-2 maps are shown. [file Image_3.pdf]
