# Supplementary material for: Identification and Molecular Characterization of a Novel Hordeivirus Associated With Yellow Mosaic Disease of Privet (Ligustrum vulgare) in Europe
Source: Front Microbiol. 2021 Sep 27;12:723350. doi: 10.3389/fmicb.2021.723350 (PMC8503643; doi:10.3389/fmicb.2021.723350)

Figure S4. Northern blot hybridization analysis of LigMV genomic and subgenomic RNAs: Alignment of the gel and the hybridization scans. For description, see the text and the caption of Figure 6.

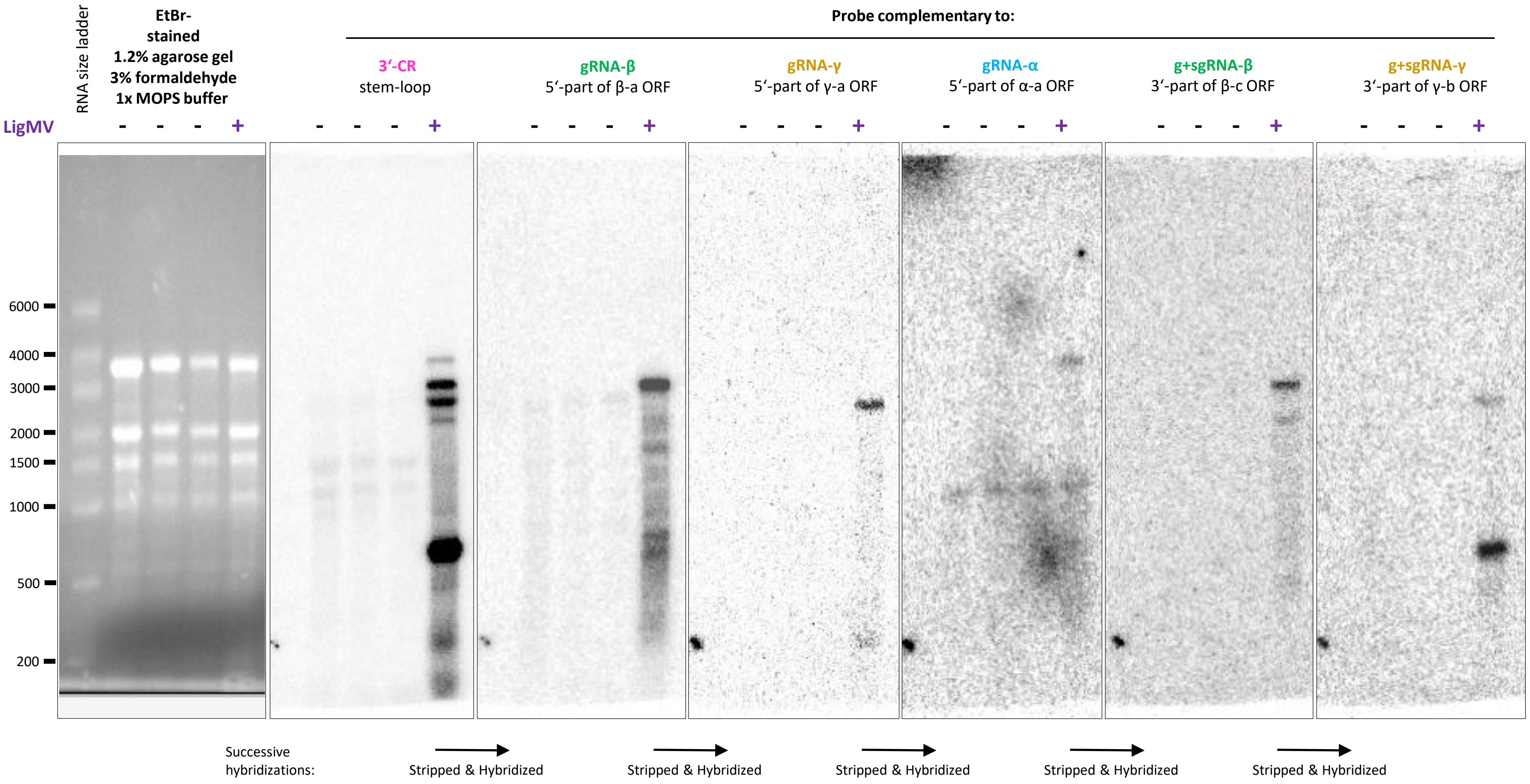

Supplement: Supplementary Figure 4 — Northern blotting hybridization analysis of LigMV genomic and subgenomic RNAs: Alignment of the gel and the hybridization scans. For description, see the text and the caption of Figure 6. [file Image_4.pdf]
