# Supplementary material for: Identification and Molecular Characterization of a Novel Hordeivirus Associated With Yellow Mosaic Disease of Privet (Ligustrum vulgare) in Europe
Source: Front Microbiol. 2021 Sep 27;12:723350. doi: 10.3389/fmicb.2021.723350 (PMC8503643; doi:10.3389/fmicb.2021.723350)

Figure S5. Blot hybridization analysis of LigMV-derived small interfering RNAs. Total RNA extracted from LigMV-infected (+) and healthy (-) *L. vulgare* leaf tissues was separated on 15% polyacrylamid-urea gel and blotted to a nylon membrane, followed by UV cross-linking. The membrane was successively hybridized with P32-lablelled DNA oligonucleotide probes specific to LigMV 3´-CR and gRNA-beta (Table S1). Positions of 21- and 22-nt viral siRNAs are indicated with arrows. EtBr-stained gel is shown as loading control.

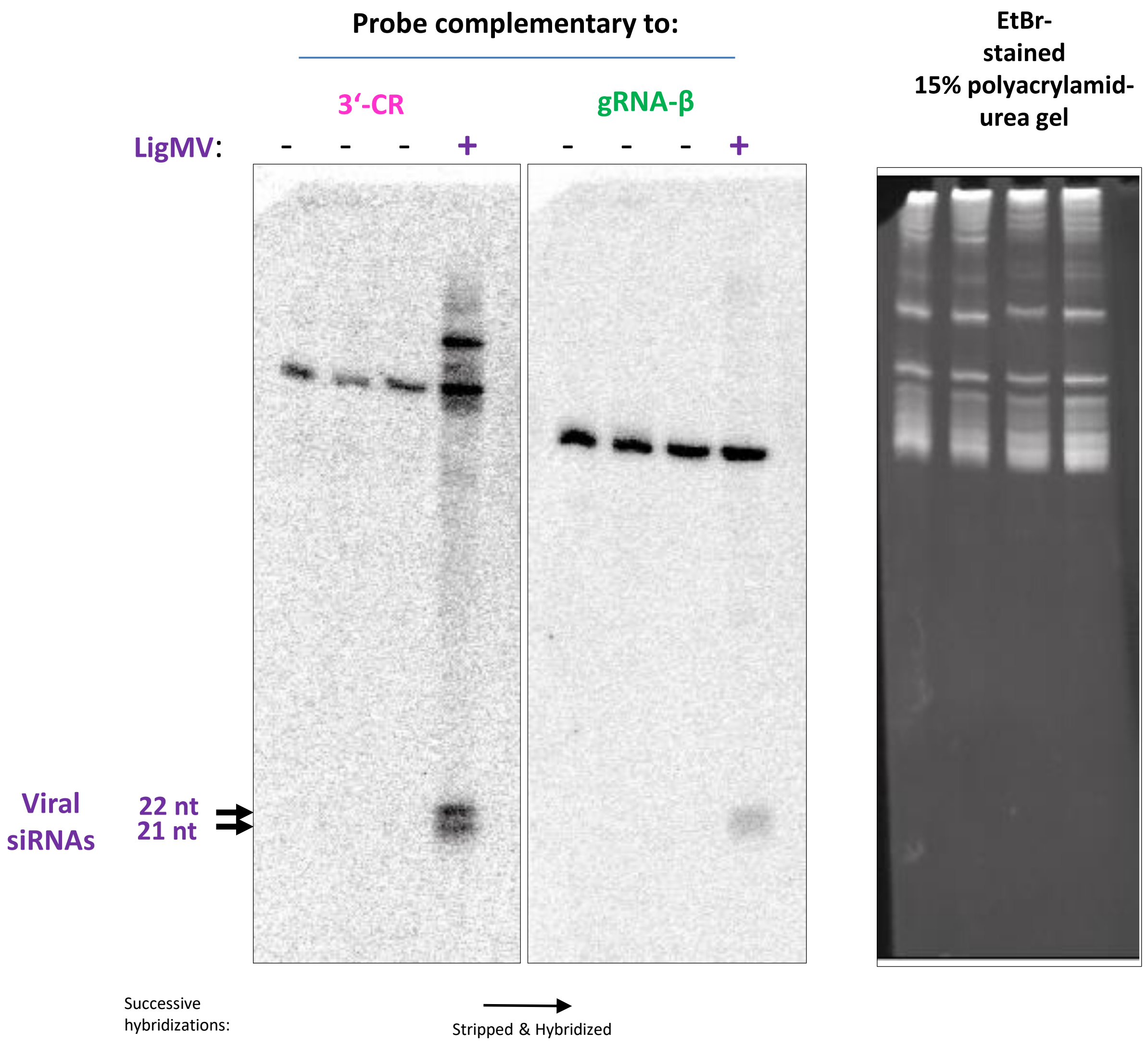

Supplement: Supplementary Figure 5 — Blot hybridization analysis of LigMV-derived small interfering RNAs. Total RNA extracted from LigMV-infected (+) and healthy (−) L. vulgare leaf tissues was separated on 15% polyacrylamid-urea gel and blotted to a nylon membrane, followed by UV cross-linking. The membrane was successively hybridized with P32-lablelled DNA oligonucleotide probes specific to LigMV 3′-CR and gRNA-beta (Supplementary Table 1). Positions of 21- and 22-nt viral siRNAs are indicated with arrows. EtBr-stained gel is shown as loading control. [file Image_5.pdf]
