# Supplementary material for: Identification and Molecular Characterization of a Novel Hordeivirus Associated With Yellow Mosaic Disease of Privet (Ligustrum vulgare) in Europe
Source: Front Microbiol. 2021 Sep 27;12:723350. doi: 10.3389/fmicb.2021.723350 (PMC8503643; doi:10.3389/fmicb.2021.723350)
Supplement: Supplementary Table 1 — DNA oligonucleotides used as primers for RT-PCR and 5′- and 3′-RACE analyses, and as probes for Northern and sRNA blot hybridization analyses. [file Table_1.pdf]

Table S1. DNA oligonucleotides used as primers for RT-PCR and 5´- and 3´-RACE analyses, and as probes for Northern and sRNA blot hybridization analyses.

Name original	Specific to / Detects	Sequence (5' to 3')
RT-PCR primers for sequencing the internal poly(A) tracts attached to LigMV gRNAs		
LigMV_3UTR_133as	3’-CR of all gRNAs /sense	CGGCTCAGTGTGCAACAATC
LigMV_3UTR_79as	3’-CR of all gRNAs /sense	AGTACGCGCTTACACACCAA
LigMV_alpha3186_s	3’-part of gRNA-α /antisense	TCTGCAAGCAGAAAAGGAGACA
LigMV_alpha3419_s	3’-part of gRNA-α /antisense	GATCGTTTGTTACTGGACATAGCAAATGAAGATGG
LigMV_beta2483_s	3’-part of gRNA-β /antisense	CCAGATTGCAAGATCTGTGGG
LigMV_beta2716_s	3’-part of gRNA-β /antisense	TACCCCTATGGGAGTGTTCCTGTGTGGGATCGC
LigMV_beta2761_s	3’-part of gRNA-β /antisense	ATGGATCATGCGACCCAAT
LigMV_gamma2138_s	3’-part of gRNA-γ /antisense	AGCACGGGCTTACAGGAAAA
LigMV_gamma2341_s	3’-part of gRNA-γ /antisense	GAAGAAGCTGGAGAGGTATCGTGATGCCTCTATGG
Primers for 3'-RACE		
LigMV_3UTR_1s	LigMV 3’-CR /antisense	TGCCTGCTATTAAGACGGTG
Hordei_CCApolyU_as	polyuridylated viral RNAs	AAAAAAAAAAAAAAAAAAAAAATGG
Reverse primers for 5'-RACE		
LigMV_alpha251_as	5’-part of gRNA-α /antisense	CGACCAACGTTCTTCTTTTC
LigMV_beta205_as	5’-part of gRNA-β /antisense	AGTCGTGCATAGCACTAGTA
LigMV_gamma276_as	5’-part of gRNA-γ /antisense	AAACAGCCTTACCCGAAAAG
Primers for RT-PCR diagnostics of LigMV		
LigMV_1079F	5’-part of gRNA-γ / sense	TGCAGCTTTGCAAACGGTAG
LigMV_ 2063R	3’-part of gRNA-γ /antisense	CAAACTCTTGGGTTGGCTGC
Probes for Northern blot hybridization analysis of LigMV genomic and subgenomic RNAs		
LigMV_3UTR_as	3’-CR of all gRNAs /sense	CGTAGGAGTACGCGCTTACACACCAATTCCCAC
LigMV_alpha190_as	5’-part of gRNA-α /sense	GCACAGTGTGTCGTGCAAAGGTGATCTCACC
LigMV_beta225_as	5’-part of gRNA-β /sense	GCATGTCTTTCCAGGACTCTTTGGCGACCCACC
LigMV_gamma191_as	5’-part of gRNA-γ /sense	CCCAAACCATCTTTTTAAGACAGAGAGAATAAACG
LigMV_beta2716_as	3’-part of gRNA-β /sense	GCGATCCCACACAGGAACACTCCCATAGGGGTA
LigMV_gamma2341_as	3’-part of gRNA-γ /sense	CCATAGAGGCATCACGATACCTCTCCAGCTTCTTC
Probe for blot hybridization analysis of viral siRNAs:		
LigMV_3UTR_s	3’-CR of gRNAs /antisense	GTGGGAATTGGTGTGTAAGCGCGTACTCCTACG
