# Supplementary material for: Identification and Molecular Characterization of a Novel Hordeivirus Associated With Yellow Mosaic Disease of Privet (Ligustrum vulgare) in Europe
Source: Front Microbiol. 2021 Sep 27;12:723350. doi: 10.3389/fmicb.2021.723350 (PMC8503643; doi:10.3389/fmicb.2021.723350)
Supplement: Supplementary Dataset 1 — Nucleotide sequences and multiple alignments of LigMV isolates and 5′-UTRs of LigMV genomic and subgenomic RNAs in comparison with other hordeiviruses. (A) Nucleotide sequences of Swiss, French and Austrian isolates of LigMV and their multiple alignments. (B) Comparison of 5′-UTRs of LigMV, LRSV, PSLV, and BSMV. (C) Nucleotide sequences of 1.9 Kb gRNA-γ region amplified by RT-PCR from 10 LigMV isolates and their multiple alignment comparison with complete and near complete genome sequences of Swiss (CH), French (FR) and Austrian (AUT) isolates obtained by Illumina sequencing (sRNA-seq, dsRNA-seq and VANA-seq). [file Data_Sheet_1.pdf]

Supplementary Dataset S1. Nucleotide sequences and multiple alignments of LigMV isolates, and 5´-UTRs of LigMV genomic and subgenomic RNAs in comparison with other hordeiviruses.

Dataset S1A. Nucleotide sequences of Swiss, French and Austrian isolates of LigMV and their multiple alignments

Dataset S1B. Comparison of 5’-UTRs of LigMV, LRSV, PSLV and BSMV

Dataset S1C. Nucleotide sequences of 1.9 Kb gRNA-γ region RT-PCR amplified from 10 LigMV isolates and their multiple alignment comparison with complete and near complete genome sequences of Swiss (CH), French (FR) and Austrian (AUT) isolates obtained by Illumina sequencing (sRNA-seq, dsRNA-seq and VANA-seq)

Dataset S1A. Nucleotide sequences of Swiss, French and Austrian isolates of LigMV and their multiple alignments

In the Swiss (CH) sRNA isolate, UNDERLINED NUCLEOTIDES in 5’- and 3’-regions were confirmed by respectively 5’-RACE and RT-PCR with 3-CR and segment-specific primers, followed by 3’-RACE (the lowercase nucleotides are not supported or deviating from sRNA contigs). The **lowercase bold** nucleotides in the poly(A) tract and upstream sequences – are those missing in the sRNA contigs. The three **bold** nucleotides in the 3’-CR are the SNP positions distinguishing each gRNA. In **BOLD RED** are the start and stop codons of viral ORFs. In cyan are positions of SNPs and indels (underlined) in the Austrian isolate, in green those in the French isolate and in yellow those in both French (FR) and Austrian (AUT) isolates. In grey, SNPs and indels in the Swiss isolate assembled from Illumina dsRNA reads (in **yellow bold** – identical SNPs shared with French and Austrian isolates).

>LigMV_alpha_CH_sRNA
acGTAATGTTTACTGCGGTAGTTTGAGTACTGCGACAGTGGTTTTGCATTAACAGTTAATCCACTTCAGGTTTTGTTCTTTTCAACATTTTCATTCCGTGGTCATAATC**ATG**GCTAGCGATGAGATCGTACGTAATTTGATTTCGAGGGAGGAAGTAATG
GGTAATCTTATTAGTACAGCATCGTCATCGGTGAGATCACCTTTGCACGACACACTGTGCTCGCACGTTAATGCCGTTGTGAATTCCTTGGAAAAGAAGAACGTTGGTCGAAGGCAGATTGATGTGAGGAGAAATTTAACATCTGATGAGTTACAGATGT
TAATAAAGGATTATCCTGAATATTCTGTGACATCATCTGCTTGTGAGAGTGGAACTCATAGTATGGCAGCATGCTATAGATTTTTAGAAACTGAATACTTATTAGATTTTGTACCAAAAAAAGAAACGACGATTTGGGATATTGGTGGGAACTGGTTCTC
GCACATGAAATTCAGGAGTGATTTGGATATACATTGTTGCTGTCCCATCCTATCTTTGCGCGATGCAGAACGCGTTGAAACTAGATATATGAGTATGCAGAGATATATGAGAGGTGATAAAGACAAATCTTTGGCTTTATTTTCAAAATATAGCTCTGTA
CTCGTAGATCAAGCGAGGCGCAAGGAGTGCATTGAGTCGGGAACTTTGAATCCTTCACAGTTGGATGGAGAATTATTTTGTGAGAATACGTTTCAGGAGTGTGTTCGAAAAGCACCCGAGGGCTCATTGCCTGTGGCGATTGCAGTTCATAGTGTGTATG
ATATTGAGGTTGAGAGTTTGGTAAGAGCTTTGAAACAAAAGGGGATCACTCAGATGTACGGATGTATGTTGTTTCCACCAAGTATCTTAGTTGGAGAAGTAAAAGGTGATTTGCCAGCGGTAAAAGGAAAGTTTAGTATAGAGAAAGGGAAAATTTCTTT
CTTTTTTCTTAACGATCCAAACGCCGGTTATACTCACGATTTAAAAGACTACCTAAGATATTTTGAAGACACATATATCACTATACAAAACGGTGTGTTCTCGATTGAGCTTCTTCAAATGAGAGGGGATACCATGTTTTTTAAGATTACTGATGTCACC
GTAGCCGCGTACCATTTGCGGTATAGGGGTGTCAAGTATGATGAAACTTTCAAGTGTATTCCTATGGTTGCTCAGACCTCCGTTGTTGTTCCGATTTTTTCTTGGAATGATCGGGGAATAGAGATTGATACAGCTCTAATTCCGAGGAATATTGTTGAAC
AGGGAGCGTCGTATATGATGAAAAATAAGGAGAAAGATCTGACGGTAAGTGTGTTGAAAAACTACTTATCTTCGATTAATAATTCTTATATTTTTAACGGCTCTCAATGTCGACAAGGAGAAAAAATTGGACCAAGTCTTATTTCACAGCTTGCGGTAAC
GTTATACTTGAGGGAAAAGGTTTACAGAAATCGAGAGAACCAAGTGGTAAAACATTTTGAAGACTTGTTTATGGATGACATTTCTTTAAGTGATTTATTTGTTGGAGCTCTTTGGAAAAATAATCGCACCGCTGTTGCTCGCATTTATAGAGTGATGAAG
ATGAGCATGGCCAAGTGGTTTGGTCTGACAGTAGATCCGAGTTCTTTTGACATTTGTGACCCTCCGCTTTTCGTAGAAATACGAGACAGATGGAAGGTTCAAGCCAGAGGTGACATCAAATTAAGCTCGTTGTATGACTTTGAGGATGATGC**G**CGGGAGT
TTGATAAAAGAGTGGAAGAGAAGCGACGTTTGGCTGAAGATATTGTCAATAAAGTTACAGGGGTTGCAAGCCCTGGAAGTCCTACGAATATGGAGAACCTACAGAAGCTAAATTCAATTTCACGCCTGTTTCTAGCTGCTACTGTGGTTAACAAATTCAA
GACTTCGTCTTTCGATAGTTTTTCAGATACGGCTTCCGTCCATTCGGAGTCTTCATTGACTTTACCTTCTTCACCGACATTATCTATCGATGGTCGACTCCCTATGTGGGAAGTAGCTCATGCAGATGTGTTGAATTTTCAACAAATTAATGGCAACATG
TTAAAGAGAACTGGTGTACCAGATAGACCAGTAGAAAGCAAAGATTATTTTGAGAACTCTAGAGCCGAGTTTTTATACTATTTGTTGTCTAGTGTTATAAGTGAAAGAGCCCAAATGATGGACATTGTTCATAGTTACAGGCTCGGTCATTTATTCACTG
ACAAGGTAGTGGCTCCTCTAACATCGTATATCTACTCTTGTGAGAGGGCGGATTGGGTGTTGAGGACTCCTCGAGCGAATGATGTGGGACATAACTTTGCGGTGCATTTCAATTTCAAAGGAGAGAGTAAAGAGACAGATGAGTACCTAGAGTATTGTGA
GATCGTTGATGTGAGATGGGACAAGTCTGGAGGTTTTATGTCAAACATTCCAATCGTACCAAAGTCTACCGGTTTTTACTTGTTTTGTGAGCCAACTAAATTGTGTAATAATTATCTTATTTACAACAATTTAGTAGATCTTTTTTCTTCGTGTAAGAAT
AGAAAGGTCACCTTTGAACTCATAGAGGGTGTTCCGGGTTGCGGAAAATCCACAATGATTTTGGAGACTGCAAACATGAGAAGTGAGGCCGTTGTAGGTGAGGGAAGACAAGCAATGGATGATCTGAGAAATAGATTCGTGAAACAGAAGAAGTGGTCAC
AAAACACAGCTAATAAGAAAGTGAGAACTTTAGATAGCGTTCTTTTGGCTACTCAACCTGACAGAATCCCGAAGGTGAAAGGCTTCCATTTCGATGAGGCATTAAAGGTGCATTTTGGTGCTATACTTTTTTGTGCAGATAAAATGGGAAGCAATTATGT
GATCGGACAAGGTGACAGAGCTCAATTGCCTATGATTAATCGAGTTGAGGGCATATCTCTGAAGTTTAGCACGCCAGATTACTCTCAAGTGAAAGTGACACCGAAATTGTTATCATATAGAATTCCGGGGGATGTTGCATATTACTTGAGTAAGAGGGCC
TGTTATAAGGACAGGGGTATTCCACAGGTTGTAACTACAAAAAATCAGGTCACAAGGTCAATGTTCGCGAGGGGGGCAGAACACAACTGAGAGGTTTGTTAGTCTACAAGACTTACCGATCGTTTTGGGTGCGCAGTATCTTACTTTTCTGCAAGCAGAAA
AGGAGACAATTATTTCGCATTTAAAGGCAAAAGGTGTGAAGGATTTCAATGTGAATACCATTCATGAATCTCAGGGAGGAACATTTAAAAATGTTATTCTTGTTCGTTTGCAAAGAACTGAGAATGAGATTTATCCTGGTGGTGTGAGAAGTTCAAGTTA
CGTTGTTGTTGGAATATCTCGACATACGGAAACTTTCACATACTGTAGTGTCGTTGATGATCGTTTGTTACTGGACATAGCAAATGAAGATGGTTTAAAAAATACTCCGATTCGGACGTTTCAAGAGTACATAATAAAA**TAA**ATAAGTTTT**tcttttttt**
taaaaaaaaaaaaaaaaaaaaaaaaaaaaaaaaaaaaaaaaaAAAAAAAAAAAAAA**A**AATGCCTGCTATTAAGACGGTGAAACCTATAAACGGGTTCAACGGGTGAGATAGTGGGAATTGGTGTGTAAGCGCGTACTCCTACG**T**CGCTGCATCAACAAT
GATCTGTGGATCGATTGTTGCACACTGAGCCG**a**AGCTCAGCTTTGGTCCTTTAGGAGACCA

>LigMV_alpha_FR_VANA
CAGCGCCCCCCTGGTAATGTTTACTGCGGTAGTTTGAGTACTGCGACAGTGGTTTTGCATTAACAGTTAATCCACTTCAGGTTTTGTTCTTTTCAACATTTTCATTCCGTGGTCATAATCATGGCTAGCGATGAGATCGTACGTAATTTGATTTCGAGGG
AGGAAGTAATGGGTAATCTTATTAGTACAGCATCGTCATCGGTGAGATCACCTTTGCACGACACACTGTGCTCGCACGTTAATGCCGTTGTGAATTCCTTGGAAAAGAAGAACGTTGGTCGAAGGCAGATTGATGTGAGGAGAAATTTAACATCTGATGA
GTTACAGATGTTAATAAAGGATTATCCTGAATATTCTGTGACATCATCTGCTTGTGAGAGTGGAACTCATAGTATGGCAGCATGCTATAGATTTTTAGAAACTGAATACTTATTAGATTTTGTACCAAAAAAAGAAACGACGATTTGGGATATTGGTGGG
AACTGGTTCTCGCACATGAAATTCAGGAGTGATTTGGATATACATTGTTGCTGTCCCATCCTATCTTTGCGCGATGCAGAACGCGTTGAAACTAGATATATGAGTATGCAGAGATATATGAGAGGTGATAAAGACAAATCTTTGGCTTTATTTTCAAAAT
ATAGCTCTGTACTCGTAGATCAAGCGAGGCGCAAGGAGTGCATTGAGTCGGGAACTTTGAATCCTTCACAGTTGGATGGAGAATTATTTTGTGAGAATACGTTTCAGGAGTGTGTTCGAAAAGCACCCGAGGGCTCATTGCCTGTGGCGATTGCAGTTCA
TAGTGTGTATGATATTGAGGTTGAGAGTTTGGTAAGAGCTTTGAAACAAAAGGGGATCACTCAGATGTACGGATGTATGTTGTTTCCACCAAGTATCTTAGTTGGAGAAGTAAAAGGTGATTTGCCAGCGGTAAAAGGAAAGTTTAGTATAGAGAAAGGG
AAAATTTCTTTCTTTTTTCTTAACGATCCAAACGCCGGTTATACTCACGATTTAAAAGACTACCTAAGATATTTTGAAGACACATATATCACTATACAAAATGGTGTGTTCTCGATTGAGCTTCTTCAAATGAGAGGGGATACCATGTTTTTTAAGATTA
CTGATGTCACCGTAGCCGCGTACCATTTGCGGTATAGGGGTGTCAAGTATGATGAAACTTTCAAGTGTATTCCTATGGTTGCTCAGACCTCCGTTGTTGTTCCGATTTTTTCTTGGAATGATCGGGGAATAGAGATTGATACAGCTCTAATTCCGAGGAA
TATTGTTGAACAGGGAGCGTCGTATATGATGAAAAATAAGGAGAAAGATCTGACGGTAAGTGTGTTGAAAAACTACTTATCTTCGATTAATAATTCTTATATTTTTAACGGCTCTCAATGTCGACAAGGAGAAAAAATTGGACCAAGTCTTATTTCACAA
CTTGCGGTAACGTTATACTTGAGGGAAAAGGTTTACAGAAATCGAGAGAACCAAGTGGTAAAACATTTTGAAGACTTGTTTATGGATGACATTTCTTTAAGTGATTTATTTGTTGGAGCTCTTTGGAAAAATAATCGCACCGCTGTTGCTCGCATTTATA
GAGTGATGAAGATGAGCATGGCCAAGTGGTTTGGTCTGACAGTAGATCCGAGTTCTTTTGACATTTGTGACCCTCCGCTTTTCGTAGAAATACGAGACAGATGGAAGGTTCAAGCCAGAGGTGACATCAAATTAAGCTCGTTGTATGACTTTGAGGATGA
TGCACGGGAGTTTGATAAAAGAGTGGAAGAGAAGCGACGTTTGGCTGAAGATATTGTCAATAAAGTTACAGGGGTTGCAAGCCCTGGAAGTCCTACGAATATGGAGAACCTACAGAAGCTAAATTCAATTTCACGCCTGTTTCTAGCAGCTACTGTGGTT
AACAAATTCAAGACTTCGTCTTTCGATAGTTTTTCAGATACGGCTTCCGTCCATTCAGAGTCTTCATTGACTTTACCTTCTTCACCGACATTATCTATCGATGGTCGACTCCCTATGTGGGAAGTAGCTCATGCAGATGTGTTGAATTTTCAACAAATTA
ATGGCAACATGTTAAAGAGAACTGGTGTACCAGATAGACCAGTAGAAAGCAAAGATTATTTTGAGAACTCTAGAGCCGAGTTTTTATACTATTTGTTGTCTAGTGTTATAAGTGAAAGAGCCCAAATGATGGACATTGTTCATAGTTACAGGCTCGGTCA
TTTATTCACTGACAAGGTAGTGGCTCCTCTAACATCGTATATCTACTCTTGTGAGAGGGCGGATTGGGTGTTGAGGACTCCTCGAGCGAATGATGTGGGACATAACTTTGCGGTGCATTTCAATTTCAAAGGAGAGAGTAAAGAGACAGATGAGTACCTA
GAGTATTGTGAGATCGTTGATGTGAGATGGGACAAGTCTGGAGGTTTTATGTCAAACATTCCAATCGTACCAAAGTCTACCGGTTTTTACTTGTTTTGTGAGCCAACTAAATTGTGTAATAATTATCTTATTTACAACAATTTAGTAGATCTTTTTTCTT
CGTGTAAGAATAGAAAGGTCACCTTTGAACTCATAGAGGGTGTTCCGGGTTGCGGAAAATCCACAATGATTTTGGAGACTGCAAACATGAGAAGTGAGGCCGTTGTAGGTGAGGGAAGACAAGCAATGGATGATCTGAGAAATAGATTCGTGAAACAGAA
GAAGTGGTCACAAAACACAGCTAATAAGAAAGTGAGAACTTTAGATAGCGTTCTTTTGGCTACTCAACCTGACAGAATCCCGAAGGTGAAAGGCTTCCATTTCGATGAGGCATTAAAGGTGCATTTTGGTGCTATACTTTTTTGTGCAGATAAAATGGGA
AGCAATTATGTGATCGGACAAGGTGACAGAGCTCAATTGCCTATGATTAATCGAGTTGAGGGCATATCTCTGAAGTTTAGCACGCCAGATTACTCTCAAGTGAAAGTGACACCGAAATTGTTATCATATAGAATTCCGGGGGATGTTGCATATTACTTGA
GTAAGAGGGCCTGTTATAAGGACAGGGGTATTCCACAGGTTGTAACTACAAAAAATCAGGTCACAAGGTCAATGTTCGCGAGGGGGGCAGAACACAACTGAGAGGTTTGTTAGTCTACAAGACTTACCGATCGTTTTGGGTGCGCAGTATCTTACTTTTCT
GCAAGCAGAAAAGGAGACAATTATTTCGCATTTAAAGGCAAAAGGTGTGAAGGATTTCAATGTGAATACCATTCATGAATCTCAGGGAGGAACATTTAAAAATGTTATTCTTGTTCGTTTGCAAAGAACTGAGAATGAGATTTATCCTGGTGGTGTGAGA
AGTTCAAGTTACGTTGTTGTTGGAATATCTCGACATACGGAAACTTTCACATACTGTAGTGTCGTTGATGATCGTTTGTTACTGGACATAGCAAATGAAGATGGTTTAAAAAATACTCCGATTCGGACGTTTCAAGAGTACATAATAAAATAAATAAGTT
TTTCTTTTTCCTTTTTTAAAAAAAAAAAAAAAAAAAAAAAAAAA

>LigMV_alpha_AUT_VANA
GAGACTCTGGTAATGTTTACTGCGGTAGTTTGAGTACTGCGACAGTGGTTTTGCATTAACAGTTAATCCACTTCAGGTTTTGTTCTTTTCAACATTTTCATTCCGTGGTCATAATCATGGCTAGCGATGAGATCGTACGTAATTTGATTTCGAGGGAGGA
AGTAATGGGTAATCTTATTAGTACAGCATCGTCATCGGTGAGATCACCTTTGCACGACACACTGTGCTCGCACGTTAATGCCGTTGTGAATTCCTTGGAAAAGAAGAACGTTGGTCGAAGGCAGATTGATGTGAGGAGAAATTTAACATCTGATGAGTTA
CAGATGTTAATAAAGGATTATCCTGAATATTCTGTGACATCATCTGCTTGTGAGAGTGGAACTCATAGTATGGCAGCATGCTATAGATTTTTAGAAACTGAATACTTATTAGATTTTGTACCAAAAAAAGAAACGACGATTTGGGATATTGGTGGGAACT
GGTTCTCGCACATGAAATTCAGGAGTGATTTGGATATACATTGTTGCTGTCCCATTCTATCTTTGCGCGATGCAGAACGCGTTGAAACTAGATATATGAGTATGCAGAGATATATGAGAGGTGATAAAGACAAATCTTTGGCTTTATTTTCAAAATATAG
CTCTGTACTCGTAGATCAAGCGAGGCGCAAGGAGTGCATTGAGTCGGGAACTTTGAATCCTTCACAGTTGGATGGAGAATTATTTTGTGAGAATACGTTTCAGGAGTGTGTTCGAAAAGCACCCGAGGGCTCATTGCCTGTGGCGATTGCAGTTCATAGT
GTGTATGATATTGAGGTTGAGAGTTTGGTAAGAGCTTTGAAACAAAAGGGGATCACTCAGATGTACGGATGTATGTTGTTTCCACCAAGTATCTTAGTTGGAGAAGTAAAAGGTGATTTGCCAGCGGTAAAAGGAAAGTTTAGTATAGAGAAAGGGAAAA
TTTCTTTCTTTTTTCTTAACGATCCGAACGCCGGTTATACTCACGATTTAAAAGACTACCTAAGATATTTTGAAGACACATATATCACTATACAAAATGGTGTGTTCTCGATTGAGCTTCTTCAAATGAGAGGGGATACCATGTTTTTTAAGATTACTGA
TGTCACCGTAGCCGCGTACCATTTGCGGTATAGGGGTGTCAAGTATGATGAAACTTTCAAGTGTATTCCTATGGTTGCTCAGACCTCCGTTGTTGTTCCGATTTTTTCTTGGAATGATCGGGGAATAGAGATTGATACAGCTCTAATTCCGAGGAATATT
GTTGAACAGGGAGCGTCGTATATGATGAAAAATAAGGAGAAAGATCTGACGGTAAGTGTGTTGAAAAACTACTTATCTTCGATTAATAATTCTTATATTTCAACGGCTCTCAATGTCGACAAGGAGAAAAAATTGGACCAAGTCTTATTTCACAGCTTG
CGGTAACGTTATACTTGAGGGAAAAGGTTTACAGAAATCGAGAGAACCAAGTGGTAAAACATTTTGAAGACTTGTTTATGGATGACATTTCTTTAAGTGATTTATTTGTTGGAGCTCTTTGGAAAAATAATCGCACCGCTGTTGCTCGCATTTATAGAGT
GATGAAGATGAGCATGGCCAAGTGGTTTGGTCTGACAGTAGATCCGAGTTCTTTTGACATTTGTGACCCTCCGCTTTTCGTAGAAATACGAGACAGATGGAAGGTTCAAGCCAGAGGTGACATCAAATTAAGCTCGTTGTATGACTTTGAGGATGATGCA
CGGGAGTTTGATAAAAGAGTGGAAGAGAAGCGACGTTTGGCTGAAGATATTGTCAATAAAGTTACAGGGGTTGCAAGCCCTGGAAGTCCTACGAATATGGAGAACCTACAGAAGCTAAATTCAATTTCACGCCTGTTTCTAGCTGCTACTGTGGTTAACA
AATTCAAGACTTCGTCTTTCGATAGTTTTTCAGATACGGCTTCCGTCCATTCGGAGTCTTCATTGACTTTACCTTCTTCACCGACATTATCTATCGATGGTCGACTCCCTATGTGGGAAGTAGCTCATGCAGATGTGTTGAATTTTCAACAAATTAATGG
CAACATGTTAAAGAGAACTGGTGTACCAGATAGACCAGTAGAAAGCAAAGATTATTTTGAGAACTCTAGAGCCGAGTTTTTATACTATTTGTTGTCTAGTGTTATAAGTGAAAGAGCCCAAATGATGGACATTGTTCATAGTTACAGGCTTGGTCATTTA
TTCACTGACAAGGTAGTGGCTCCTCTAACATCGTATATCTACTCTTGTGAGAGGGCGGATTGGGTGTTGAGGACTCCTCGAGCGAATGATGTGGGACATAACTTTGCGGTGCATTTCAATTTCAAAGGAGAGAGTAAAGAGACAGATGAGTACCTAGAGT
ATTGTGAGATCGTTGATGTGAGATGGGACAAGTCTGGAGGTTTTATGTCAAACATTCCAATCGTACCAAAGTCTACCGGTTTTTACTTGTTTTGTGAGCCAACTAAATTGTGTAATAATTATCTTATTTACAACAATTTAGTAGATCTTTTTTCTTCGTG
TAAGAATAGAAAGGTCACCTTTGAACTCATAGAGGGCGTTCCGGGTTGCGGAAAATCCACAATGATTTTGGAGACTGCAAACATGAGAAGTGAGGCCGTTGTAGGTGAGGGAAGACAAGCAATGGATGATCTGAGAAATAGATTCGTGAAACAGAAGAAG
TGGTCACAAAACACAGCTAATAAGAAAGTGAGAACTTTAGATAGCGTTCTTTTGGCTACTCAACCTGACAGAATCCCGAAAGTGAAAGGCTTCCATTTCGATGAGGCATTAAAGGTGCATTTTGGTGCTATACTTTTTTGTGCAGATAAAATGGGAAGCA
ATTATGTGATCGGACAAGGTGACAGAGCTCAATTGCCTATGATTAATCGAGTTGAGGGCATATCTCTGAAGTTTAGCACGCCAGATTACTCTCAAGTGAAAGTGACACCGAAATTGTTATCATATAGAATTCCGGGGGATGTTGCATATTACTTGAGTAA
GAGGGCCTGTTATAAGGACAGGGGTATTCCACAGGTTGTAACTACAAAAAATCAGGTCACAAGGTCAATGTTCGCGAGGGGGGCAGAACACAACTGAGAGGTTTGTTAGTCTACAAGACTTACCGATCGTTTTGGGTGCGCAGTATCTTACTTTTCTGCAA
GCAGAAAAGGAGACAATTATTTCGCATTTAAAGGCAAAAGGTGTGAAGGATTTCAATGTGAATACCATTCATGAATCTCAGGGAGGAACATTTAAAAATGTTATTCTTGTTCGTTTGCAAAGAACTGAGAATGAGATTTATCCTGGTGGTGTGAGAAGTT
CAAGTTACGTTGTTGTTGGAATATCTCGACATACGGAAACTTTCACATACTGTAGTGTCGTTGATGATCGTTTGTTACTGGACATAGCAAATGATGATGGTTTAAAAAATACTCCGATTCGGACGTTTCAAGAGTACATAATAAAATAAATAAGTATTTC
TTATTTTAAAAAAAAAAAAAAAAAAAAAAAAAAAAAAAAAAAAAAACCAATTTCCCTTTTAAAATAAAAAAAAAAAAAAAAAAAAAATTTTTTTTTTTTTAAAAAAAAAAAAAAAAAAAAAAAAAAAAAAAAAAAAAAAAAAAAAAAAAAAAATGCCTGCTATTAAG
ACGGTGAACTGT

>LigMV_alpha_CH_dsRNA
GTAATGTTTACTGCGGTAGTTTGAGTACTGCGACAGTGGTTTTGCATTAACAGTTAATCCACTTCAGGTTTTGTTCTTTTCAACATTTTCATTCCGTGGTCATAATCATGGCTAGCGATGAGATCGTACGTAATTTGATTTCGAGGGAGGAAGTAATGGG
TAATCTTATTAGTACAGCATCGTCATCGGTGAGATCACCTTTGCACGACACACTGTGCTCGCACGTTAATGCCGTTGTGAATTCCTTGGAAAAGAAGAACGTTGGTCGAAGGCAGATTGATGTGAGGAGAAATTTAACATCTGATGAGTTACAGATGTTA
ATAAAGGATTATCCTGAATATTCTGTGACATCATCTGCTTGTGAGAGTGGAACTCATAGTATGGCAGCATGCTATAGATTTTTAGAAACTGAATACTTATTAGATTTTGTACCAAAAAAAGAAACGACGATTTGGGATATTGGTGGGAACTGGTTCTCGC
ACATGAAATTCAGGAGTGATTTGGATATACATTGTTGCTGTCCCATCCTATCTTTGCGCGATGCAGAACGCGTTGAAACTAGATATATGAGTATGCAGAGATATATGAGAGGTGATAAAGACAAATCTTTGGCTTTATTTTCAAAATATAGCTCTGTACT
CGTAGATCAAGCGAGGCGCAAGGAGTGCATTGAGTCGGGAACTTTGAATCCTTCACAGTTGGATGGAGAATTATTTTGTGAGAATACGTTTCAGGAGTGTGTTCGAAAAGCACCCGAGGGCTCATTGCCTGTGGCGATTGCAGTTCATAGTGTGTATGAT
ATTGAGGTTGAGAGTTTGGTAAGGGCTTTGAAACAAAAGGGGATCACTCAGATGTACGGATGTATGTTGTTTCCACCAAGTATCTTAGTTGGAGAAGTGAAAGGTGATTTGCCAGCGGTAAAAGGAAAGTTAAATAGAG
ATGTTCTTTTATGATTACTGATGTCACCGAAGCCGCGTACCATTTGCGGTATAGGGGTGTCAAGTATGATGAAACTTTCAAGTGTATTCCTATGGTTGCTCAGACCTCCGTTGTTGTTCCGATTTTTTCTTGGAATGATCGGGGAATAGAGATTGATACA
GCTCTAATTCCGAGGAATATTGTTGAACAGGGAGCGTCGTATATGATGAAAAATAAGGAGAAAGATCTGACGGTAAGTGTGTTGAAAAACTACTTATCTTCGATTAATAATTCTTATATTTTTAACGGCTCTCAATGTCGACAAGGAGAAAAAATTGGAC
CAAGTCTTATTTCACAGCTTGCGGTAACGTTATACTTGAGGGAAAAGGTTTACAGAAATCGAGAGAACCAAGTGGTAAAACATTTTGAAGACTTGTTTATGGATGACATTTCTTTAAGTGATTTATTTGCTGGAGCTCTTTGGAAAAATAATCGCACCGC
TGTTGCTCGCATTTATAGAGTGATGAAGATGAGCATGGCCAAGTGGTTTGGTCTGACAGTAGATCCGAGTTCTTTTGACATTTGTGACCCTCCGCTTTTCGTAGAAATACGAGATAGATGGAAGGTTCAAGCCAGAGGTGACATCAAATTAAGCTCGTTG
TATGACTTTGAGGATGATGCACGGGAGTTTGATAAAAGAGTGGAAGAGAAGCGACGTTTGGCTGAAGATATTGTCAATAAAGTTACAGGGGTTGCAAGCCCTGGAAGTCCTACGAATATGGAGAACCTACAGAAGCTAAATTCAATTTCACGCCTGTTTC
TAGCTGCTACTGTGGTTAACAAATTCAAGACTTCGTCTTTCGATAGTTTTTCAGATACGGCTTCCGTCCATTCGGAGTCTTCATTGACTTTACCTTCTTCACCGACATTATCTATCGATGGTCGACTCCCTATGTGGGAAGTAGCTCATGCAGATGTGTT
GAATTTTCAACAAATTAATGGCAACATGTTAAAGAGAACTGGTGTACCAGATAGACCAGTAGAAAGCAAAGATTATTTTGAGAACTCTAGAGCCGAGTTTTTATACTATTTGTTGTCTAGTGTTATAAGTGAAAGAGCCCAAATGATGGACATTGTTCAT
AGTTACAGGCTCGGTCATTTATTCACTGACAAGGTAGTGGCTCCTCTAACATCGTATATCTACTCTTGTGAGAGGGCGGATTGGGTGTTGAGGACTCCTCGAGCGAATGATGTGGGACATAACTTTGCGGTGCATTTCAATTTCAAAGGAGAGAGTAAAG
AGACAGATGAGTACCTAGAGTATTGTGAGATCGTTGATGTGAGATGGGACAAGTCTGGAGGTTTTATGTCAAACATTCCAATCGTACCAAAGTCTACCGGTTTTTACTTGTTTTGTGAGCCGACTAAATTGTGTAATAATTATCTTATTTACAACAATTT
AGTAGATCTTTTTTCTTCGTGTAAGAATAGAAAGGTCACCTTTGAACTCATAGAGGGTGTTCCGGGTTGCGGAAAATCCACAATGATTTTGGAGACTGCAAACATGAGAAGTGAGGCCGTTGTAGGTGAGGGAAGACAAGCAATGGATGATCTGAGAAAT
AGATTCGTGAAACAGAAGAAGTGGTCACAAAACACAGCTAATAAGAAAGTGAGAACTTTAGATAGCGTTCTTTTGGCTACTCAACCTGACAGAATCCCGAAGGTGAAAGGCTTCCATTTCGATGAGGCATTAAAGGTGCATTTTGGTGCTATACTTTTTT
GTGCAGATAAAATGGGAAGCAATTATGTGATCGGACAAGGTGACAGAGCTCAATTGCCTATGATTAATCGAGTTGAGGGCATATCTCTGAAGTTTAGCACGCCAGATTACTCTCAAGTGAAAGTGACACCGAAATTGTTATCATATAGAATTCCGGGGGA
TGTTGCATATTACTTGAGTAAGAGGGCCTGTTATAAGGACAGGGGTATTCCACAGGTTGTAACTACAAAAAATCAGGTCACAAGGTCAATGTTCGCGAGGGGGGCAGAACACAGCTGAGAGGTTTGTTAGTCTACAAGACTTACCGATCGTTTTGGGTGCG
CAGTATCTTACTTTTCTGCAAGCAGAAAAGGAGACAATTATTTCGCATTTAAAGGCAAAAGGTGTGAAGGATTTCAATGTGAATACCATTCATGAATCTCAGGGAGGAACATTTAAAAATGTTATTCTTGTTCGTTTGCAAAGAACTGAGAATGAGATTT
ATCCTGGTGGTGTGAGAAGTTCAAGTTACGTTGTTGTTGGAATATCTCGACATACGGAAACTTTCACATACTGTAGTGTCGTTGATGATCGTTTGTTACTGGACATAGCAAATGAAGATGGTTTAAAAAATACTCCGAYTCGGACGTTTCAAGAGTACAT

AATAAAATAAATAAGTTT

>LigMV_beta_CH_sRNA
cagtaaaggtgtacttgtaccaacgcatgattGTAAAAATACCAACAATCGTAAAGAGCTTGCTTTGCTCCTCTTTACAAAATCTATCCTTTTATATTAGTAATAGGTTTTTGTATTTTTACGTTTACAATGGCTAATCTTGGGTTAACTGTAAAAGGTG
GTGGTCACTACGATGAAACTCAGTGGTACAACAATGTTGTACGTACTAGTGCTATGCACGACTGGTGGGTCGCCAAAGAGTCCTGGAAAGACATGCTTGATGCACTTTCTAAGGTTCAATTTGATGTGATCAATTCTCGTAGAGCAATCGCCGAAATCAT
TATTAACCTAAAAAGAGATTTACCTGCTGCCTTAGATCGACGTTTCGCTGGGTCCACTGGGACTTTAGGCGCTGCCAACTACCGTGAAGCTCCTCTCTTTGTGAGGTTAGATGGTGCTGCTCGAGCACACTTAACAGCTCTTGCCAGAATTGGTGACCAG
CCCTCTACATCACGAGACTTGGAACTTTACCGTGGGAAAGCTACCGTCCAGCAACCAGCTTCTGCAAATCCTCCTGCAATTAAAGATATTCAGCCGTTAAGGGACGGTGCATTGCACCTGACCTGGGATTTGAAAGACCTCGTTGTCAGTGATCCGCCGA
TTTATGACAGATTGACGTTCGAAGAACAGTTCCATCTGGTCTGGTACGAAAGAGTACCGGTTGTTCAGCAGTAAATTTTGATTATACATTGTAAACAATAATAATAGACTTTTCTTTGTGTGTTTGTTTGTTTGTTTGTTTCTTTAGTTCTTTATTTTTC
TAGTTTGTTTCTAGGATGTTTTTTGTGTTTGTTAACCATGTCAATCGTTGATCATTGTTGAAAACGTTAAGAAAGTTGTAAGTGAAGAAACTCCCGTAATCGATGATAAGAAACCGACAAAGAAGAAACGAAGGAATGTCAACAAGAAAAAGAAACAGCGT
GAGCCATTGGCTGCTGTGACTGATTCTAAAGAAAATTCTTTTAGGAGGTCGAATAGCCTTGAATCTTTGGGGAAGTATTTAGATACGTGGAAGGATTCTGAAAATTCAAAAAAGAAGAAATCTGTGGAAGAGAAGCACGCTGCAAATGACGCCAGACGTT
TTGGTTTAGCTGAGAAGTTTGCGTTGCAGTTGCATTCTGATTGCGTAGCGGCTGGTTTCAAGGATACTGGTAAGAATTTTCAAATGTTGCCTGCAAATTATCTTGAGAGAAGTAGATTACTCGCTCTCTATGATAAGAATCTCAAGCAGAGTTTAAAAAA
GGCGTGTGAAACTGAACAGGAGAGAATTAGGACTAAAGTGTTCCTTTTAAGGAGCGCAAGACCTAATGTTAATTTCAAAATTGGAATTATCTCCGGTGTTGCTGGTTCTGGAAAATCTACACTCATAAGAAGTTTGTGTTCGAAATCGAATGCACTGTGC
GTACTGGCGAACCCTCGTTTGAAAGAGACAGACTACAAAGGTCAAGACAAGACCTTTACGCTGCAACAAGTTCTTTTGTCTATTGTTCCAATGCGTGCTGATATAATCGTCGTTGATGAATATACTTTGGCTGAAAGCGCAGAAATCCTACTGCTGCAAC
GTCTATTGCAAGCTAGCTTCTTGCTCCTCTTTGGGGACGTTGCCCAAGGCGAATCTGATAATGCTTCGAGCTTAGAGTATCTCCAGTTGCCTGTAGTTTACAGGTCTGTTACATCGCACCGTTTGGGAAACGAGACAGCTGCTTTTTGCAAAAAGCATGG
TTTGGCTTTTGATTCCGATTCTCCAGTGAAAGATGAAATAATTACGAGTGGATATGAAGGAGACACAGAGACAACAGAGAAAAACATTGCCTTTTCCGCAGCGACAGTTGAAGACTTGCATGAGGCTGGTGTTGAGGCGACCTTGGTTTTGGAAACACAA
GGAAAAGAATATGATAGTGTTACTTTGTTTGTAAGAGATAGTGACAGAGAAGCTGCAAGCAATCCGCATGCACGAACTGTTGCTTTTACTCGTCATAAACGTTTGTTAATAATTAGGGCTGAGGAAGAAATTCTTCCTTCTCTCCTTAATGGTGAATTAA
ACGTTAAGATAAGTGCAAACTCTCATAAATACGGTGACATAAAATATTCTCATGCCGACAACAGTAGCTCAGAGACCTAACAAATATTGGCCTATAGTGGTTGGAGTTGGCTTGATTGGTTTGTTTGGTTATTTGATTTTCACTAATCAAAAACACTCTA
CCCAATCTGGCGACAACATTCACAAATTTGCTAACGGTGGTTCTTACGCGGACGGTTCTAAAAGAATAAATTACAATAGAAATAACGATCTTTCTTACGGTTACAAGGGTTTATTTAGTGGTAGTAATAATGCCTCACCTCCTTAACTTGTCTCTGCTCGG
AATGTTCCTTACAGCCATGTGTGCCGGAATTTACGGAGAATACATGCGCAGAAAGGAACAATCAGGATGTATTGTTTGCCCCCCAGATTGCAAGATCTGTGGGTAATGACTACATCTTCGGTGTGGTTTATGGAATTATAGGTGGAATACTATCAGTTTT
AATCCTTATATGGTTGTTTGGTTTAACATCAGATAATGGAAGAAAGCCAGCTGTTTCTTGGTTTAGACAAGACCTTAACGGTTTTGAAATGCAAGTAACGGAAGGTTCCCCAATTGATCCTGAAGTAGTAAAAGCTGTCCATCACTTTCAGAAATACCCC
TATGGGAGTGTTCCTGTGTGGGATCGCTTTGATAACATAATGGATCATGCGACCCAATGGTTAACTTCATTTATTAATTTGATGTTGTTTTTATTCATGTGTGGTGTGGTTTTCTTTTTCGTAGGGTGTTTTTACATCCTATGATTTTTTTTTTAAAAA
AAAaaaaaaaaaaaaaaaaaaaaaaaaaaaaaaaaaAAAAAAAAAAAAAAAAATGCCTGCTATTAAGACGGTGAAACCTATAAACGGGTTCAACGGGTGAGATAGTGGGAATTGGTGTGTAAGCGCGTACTCCTACGaCGCTGCATCAACAATGATCTG
TGGATCGATTGTTGCACACTGAGCCGGAGCTCAGCTTTGGTCCTTTAGGAGACCA

>LigMV_beta_FR_VANA
CGCTACGCAATCAGAATGCAACTGCAACGCAAACTTCTCAGCTAAACCAAAACGTCTGGCGTCATTTGCAGCGTGCTCCTCTTTACAAAATCTATCCTTTTATATTAGTAATAGGTTTTTGTATTTTTACGTTTACAATGGCTAATCTTGGGTTAACTGT
AAAAGGTGGTGGTCACTACGATGAAACTCAGTGGTACAACAATGTTGTACGTACTAGTGCTATGCACGACTGGTGGGTCGCCAAAGAGTCCTGGAAAGACATGCTTGATGCACTTTCTAAGGTTCAATTTGATGTGATCAATTCTCGTAGAGCAATCGCC
GAAATCATTATTAACCTAAAAAGAGATTTACCTGCTGCCTTAGATCGACGTTTCGCTGGGTCCACTGGGACTTTAGGCGCTGCCAACTACCGTGAAGCTCCTCTCTTTGTGAGGTTAGATGGTGCTGCTCGAGCACACTTAACAGCTCTTGCCAGAATTG
GTGACCAGCCCTCTACATCACGAGACTTGGAACTTTACCGTGGGAAAGCTACCGTCCAGCAACCAGCTTCTGCAAATCCTCCTGCAATTAAAGATATTCAGCCGTTAAGGGACGGTGCATTGCACCTGACCTGGGATTTGAAAGACCTCGTTGTCAGTGA
TCCGCCGATTTATGACAGATTGACGTTCGAAGAACAGTTCCATCTGGTCTGGTACGAAAGAGTACCGGTTGTTCAGCAGTAAATTTTGATTATACATTGTAAACAATAATAATAGACTTTTCTTTGTGTGTTTGTTTGTTTGTTTGTTTGTTTCTTTAGT
TCTTTATTTTTCTAGTTTGTTTCTAGGATGTTTTTGTGTTTGTTAACCATGTCAATCGTTGATCAAGTTGAAAACGTTAAGAAAGTTGTAAGTGAAGAAACTCCCGTAATCGATGATAAGAAACCGACAAAGAAGAAACGAAGGAATGTCAACAAGAAAA
AGAAACAGCGTGAGCCATTGGCTGCTGTGACTGATTCTAAAGAAAATTCTTTTAGGAGGTCGAATAGCCTTGAATCTTTGGGGAAGTATTTAGATACGTGGAAGGATTCTGAAAATTCAAAAAAGAAGAAATCTGTGGAAGAGAAGCACGCTGCAAATGA
CGCCAGACGTTTTGGTTTAGCTGAGAAGTTTGCGTTGCAGTTGCATTCTGATTGCGTAGCGGCTGGTTTCAAGGATACTGGTAAGAATTTTCAAATGTTGCCTGCAAATTATCTTGAGAGAAGTAGATTACTCGCTCTCTATGATAAGAATCTCAAGCAG
AGTTTAAAAAAGGCGTGTGAAACTGAACAGGAGAGAATTAGGACTAAAGTGTTCCTTTTAAGGAGCGCAAGACCTAATGTTAATTTCAAAATTGGAATTATCTCCGGTGTTGCTGGTTCTGGAAAATCTACACTCATAAGAAGTTTGTGTTCGAAATCGA
ATGCACTGTGCGTACTGGCGAATCCTCGTTTGAAAGAGACAGACTACAAAGGTCAAGACAAGACCTTTACGCTGCAACAAGTTCTTTTGTCTATTGTTCCAATGCGTGCTGATATAATCGTCGTTGATGAATATACTTTGGCTGAAAGCGCAGAAATCCT
ACTGCTGCAACGTCTATTGCAAGCTAGCTTCTTGCTCCTCTTTGGGGACGTTGCCCAAGGCGAATCTGATAATGCTTCGAGCTTAGAGTATCTCCAGTTGCCTGTAGTTTACAGGTCTGTTACATCGCACCGTTTGGGAAACGAGACAGCTGCTTTTTGC
AAAAAGCATGGTTTGGCTTTTGATTCCGATTCTCCAGTGAAAGATGAAATAATTACGAGTGGATATGAAGGAGACACAGAGACAACAGAGAAAAACATTGCCTTTTCCGCAGCGACAGTTGAAGACTTGCATGAGGCTGGTGTTGAGGCGACCTTGGTTT
TGGAAACACAAGGAAAAGAATATGATAGTGTTACTTTGTTTGTAAGAGATAGTGACAGAGAAGCTGCAAGCAATCCGCATGCACGAACTGTTGCTTTTACTCGTCATAAACGTTTGTTAATAATTAGGGCTGAGGAAGAAATTCTTCCTTCTCTCCTTAA
TGGTGAATTAAACGTTAAGATAAGTGCAAACTCTCATAAATACGGTGACATAAAATATTCTCATGCCGACAACAGTAGCTCAGAGACCTAACAAATATTGGCCTATAGTGGTTGGAGTTGGCTTGATTGGTTTGTTTGGTTATTTGATTTTCACTAATCA
AAAACACTCTACCCAATCTGGCGACAACATCCACAAATTTGCTAACGGTGGTTCTTACGCGGACGGTTCTAAAAGAATAAATTACAATAGAAATAACGATCTTTCTTACGGTTACAAGGGTTTATTTAGTGGTAGTAATAATGCCTCATTCCTTAACTTG
TCTCTGCTCGGAATGTTCCTTACAGCCATGTGTGCCGGAATTTACGGAGAATACATGCGCAGAAAGGAACAATCAGGATGTATTGTTTGCCCCCCAGATTGCAAGATCTGTGGGTAATGACTACATCTTCGGTGTGGTTTATGGAATTATAGGTGGAATA
CTATCAGTTTTAATCCTTATATGGTTGTTTGGTTTAACATCAGATAGTGGAAGAAAGCCAGCTGTTTCTTGGTTTAGACAAGACCTTAACGGTTTTGAAATGCAAGTAACGGAAGGTTCCGCAATTGATCCTGAAGTAGTAAAAGCTGTCCATCACTTTC
AGAAATACCCCTATGGGAGTGTTCCTGTGTGGGATCACTTTGATAACATAATGGATCATACGACCCAATGGTTAACTTCATTTATTAATTTGATGTTGTATTTATTCATGTGTGGTGTGGTTTTCTTTTTCGTAGGGTGTTTTTACATCCTATGATTTTT
TTTATTAAAAAAAAAAAAAAAAAAAAAAAAAAAAAAAAAAAAAAAAAAAAAAAAAAAAAAAAAAAAAAAAAAAAA

>LigMV_beta_AUT_VANA
GCAAAGCGGTTGCTCCTCTTTACAAAATCTATCCTTTTATATTAGTAGTAGGTTTTTGTATTTTTACGTTTACAATGGCTAATCTTGGGTTAACTGTAAAAGGTGGTGGTCACTACGATGAAACTCAGTGGTACAACAATGTTGTACGTACTAGTGCTAT
GCACGACTGGTGGGTCGCCAAAGAGTCCTGGAAAGACATGCTTGATGCACTTTCTAAGGTTCAATTTGATGTGATCAATTCTCGTAGAGCAATCGCCGAAATCATTATTAACCTAAAAAGAGATTTACCTGCTGCCTTAGATCGACGTTTCGCTGGGTCC
ACTGGGACTTTAGGCGCTGCCAACTACCGTGAAGCTCCTCTCTTTGTGAGGTTAGATGGTGCTGCTCGAGCACACTTAACAGCTCTTGCCAGAATTGGTGACCAGCCCTCTACATCACGAGACTTGGAACTTTACCGTGGGAAAGCTACCGTCCAGCAAC
CAGCTTCTACAAATCCTCCTGCAATTAAAGATATTCAGCCGTTAAGGGACGGTGCATTGCACCTGACCTGGGATTTGAAAGACCTCGTTGTCAGTGATCCGCCGATTTATGACAGATTGACGTTCGAAGAACAGTTCCATCTGGTCTGGTACGAAAGAGT
ACCGGTTGTTCAGCAGTAAATTTTGATTATACATTGTAAACAATAATAATAGACTTTTCTTTGTGTGTTTGTTTGTTTGTTTGTTTGTTTCTTTAGTTCTTTATTTTTCTAGTTTGTTTCTAGGATGTTTTTGTGTTTGTTAACCATGTCAATCGTTGAT
CAAGTTGAAAACGTTAAGAAAGTTGTAAGTGAAGAAACTCCCGTAATCGATGATAAGAAACCGACAAAGAAGAAACGAAGGAATGTCAACAAGAAAAAGAAACAGCGTGAGCCATTGGCTGCTGTGACTGATTCTAAAGAAAATTCTTTTAGGAGGTCGA
ATAGCCTTGAATCTTTGGGGAAGTATTTAGATACGTGGAAGGATTCTGAAAATTCAAAAAAGAAGAAATCTGTGGAAGAGAAGCACGCTGCAAATGACGCCAGACGTTTTGGTTTAGCTGAGAAGTTTGCGTTGCAGTTGCATTCTGATTGCGTAGCGGC
TGGTTTCAAGGATACTGGTAAGAATTTTCAAATGTTGCCTGCAAATTATCTTGAGAGAAGTAGATTACTCGCTCTCTATGATAAGAATCTCAAGCAGAGTTTAAAAAAGGCGTGTGAAACTGAACAGGAGAGAATTAGGACTAAAGTGTTCCTTTTAAGG
AGCGCAAGACCTAATGTTAATTTCAAAATTGGAATTATCTCCGGTGTTGCTGGTTCTGGAAAATCTACACTCATAAGAAGTTTGTGTTCGAAATCGAATGCACTGTGCGTACTGGCGAATCCTCGTTTGAAAGAGACAGACTACAAAGGTCAAGACAAGA
CCTTTACGCTGCAACAAGTTCTTTTGTCTATTGTTCCAATGCGTGCTGATATAATCGTCGTTGATGAATATACTCTGGCTGAAAGCGCAGAAATCCTACTGCTGCAACGTCTATTGCAAGCTAGCTTCTTGCTCCTCTTTGGGGACGTTGCCCAAGGCGA
ATCTGACAATGCTTCGAGCTTAGAGTATCTCCAGTTGCCTGTAGTTTACAGGTCTGTTACATCGCACCGTTTGGGAAACGAGACAGCTGCTTTTTGCAAAAAGCATGGTTTGGCTTTTGATTCCAATTCTCCAGTGAAAGATGAAATAATTACGAGTGGA
TATGAAGGAGACACAGAGACAACAGAGAAAAACATTGCCTTTTCCGCAGCGACAGTTGAAGACTTGCATGAGGCTGGTGTTGAGGCGACCTTGGTTTTGGAAACACAAGGAAAAGAATATGATAGTGTTACTTTGTTTGTAAGAGATAGTGACAGAGAAG
CTGCAAGCAATCCGCATGCACGAACTGTTGCTTTTACTCGTCATAAACGTTTGTTAATAATTAGGGCTGAGGAAGAAATTCTTCCTTCTCTCCTTAATGGTGAATTAAACGTTAAGATAAGTGCAAACTCTCATAAATACGGTGACATAAAATATTCTCA
TGCCGACAACAGTAGCTCAGAGACCTAACAAATATTGGCCTATAGTGGTTGGAGTTGGCTTGATTGGTTTGTTTGGTTATTTGATTTTCACTAATCAAAAACACTCTACCCAATCTGGCGACAACATCCACAAATTTGCTAACGGTGGTTCTTACGCGGA
CGGTTCTAAAAGAATAAATTACAATAGAAATAACGATCTTTCTTACGGTTACAAGGGTTTATTTAGTGGTAGTAATAATGCCTCACTCCTTAACTTGTCTCTGCTCGGAATGTTCCTTACAGCCATGTGTGCCGGAATTTACGGAGAATACATGCGCAGA
AAGGAACAATCAGGATGTATTGTTTGCCCCCCAGATTGCAAGATCTGTGGGTAATGACTACATCTTCGGTGTGGTTTATGGAATTATAGGTGGAATACTATCAGTTTTAATCCTTATATGGTTGTTTGGTTTAACATCAGATAATGGAAGGAAGCCAGCT
GTTTCTTGGTTTAGACAAGACCTTAACGGTTTTGAAATGCAAGTAACGGAAGGTTCCCCAATTGATCCTGAAGTAGTAAAAGCTGTCCATCACTTTCAGAAATACCCCTATGGGAGTGTTCCTGTGTGGGATCACTTTGATAACATAATGGATCATGCGA
CCCAATGGTTAACTTCATTTATTAATTTGATGTTGTTTTTATTCATGTGTGGTGTGGTTTTCTTTTTCGTAGGGTGTTTTTACATCCTATGATTTTTTTTAAAAAAAAAAAAAAAAAAAAAAAAAAAAAAAAAAAAAAAAAAAAAAAAAAAAAAAA

>LigMV_beta_CH_dsRNA
TTTTTTTTTTGTATTTTTACGTTTACAATGGCTAATCTTGGGTTAACTGTAAAAGGTGGTGGTCACTACGATGAAACTCAGTGGTACAACAATGTTGTACGTACTAGTGCTATGCACGACTGGTGGGTCGCCAAAGAGTCCTGGAAAGACATGCTTGATG
CACTTTCTAAGGTTCAATTTGATGTGATCAATTCTCGTAGAGCAATCGCCGAAATCATTATTAACCTAAAAAGAGATTTACCTGCTGCCTTAGATCGACGTTTCGCTGGGTCCACTGGGACTTTAGGCGCTGCCAACTACCGTGAAGCTCCTCTCTTTGT
GAGGTTAGATGGTGCTGCTCGAGCACACTTAACAGCTCTTGCCAGAATTGGTGACCAGCCCTCTACATCACGAGACTTGGAACTTTACCGTGGGAAAGCTACCGTCCAGCAACCAGTTTCTGCAAATCCTCCTGCAATTAAAGATATTCAACCGTTAAGG
GACGGTGCATTGCACCTGACCTGGGATTTGAAAGACCTCGTTGTCAGTGATCCGCCGATTTATGACAGATTGACGTTCGAAGAACAGTTCCATCTGGTCTGGTACGAAAGAGTACCGGTTGTTCAGCAGTAAATTTTGATTATACATTGTAAACAATAAT
AGACTTTTCTTTGTGTGTTTGTTTGTTTGTTTGTTTGTTTCTTTAGTTCTTTATTTTTCTAGTTTGTTTCTAGGATGTTTTTTGTGTTTGTTAACCATGTCAATCGTTGATCAAGTTGAAAACGTTAAGAAAGTTGTAAGTGAAGAAACTCCCGTAATCG
ATGATAAGAAACCGACAAAGAAGAAACGAAGGAATGTCAACAAGAAAAAGAAACAGCGTGAGCCATTGGCTGCTGTGACTGATTCTAAAGAAAATTCTTTTAGGAGGTCGAATAGCCTTGAATCTTTGGGGAAGTATTTAGATACGTGGAAGGATTCTGA
AAATTCAAAAAAGAAGAAATCTGTGGAAGAGAAGCACGCTGCAAATGACGCCAGACGTTTTGGTTTAGCTGAGAAGTTTGCGTTGCAGTTGCATTCTGATTGCGTAGCGGCTGGTTTCAAGGATACTGGTAAGAATTTTCAAATGTTGCCTGCAAATTAT
CTTGAGAGAAGTAGATTACTCGCTCTCTATGATAAGAATCTCAAGCAGAGTTTAAAAAAGGCGTGTGAAACTGAACAGGAGAGAATTAGGACTAAAGTGTTCCTTTTAAGGAGCGCAAGACCTAATGTTAATTTCAAAATTGGAATTATCTCCGGTGTTG
CTGGTTCTGGAAAATCTACACTCATAAGAAGTTTGTGTTCGAAATCGAATGCACTGTGCGTACTGGCGAATCCTCGTTTGAAAGAGACAGACTACAAAGGTCAAGACAAGACCTTTACGCTGCAACAAGTTCTTTTGTCTATTGTTCCAATGCGTGCTGA
TATAATCGTCGTTGATGAATATACTTTGGCTGAAAGCGCAGAAATCCTACTGCTGCAACGTCTATTGCAAGCTAGCTTCTTGCTCCTCTTTGGGGACGTTGCCCAAGGCGAATCTGATAATGCTTCGAGCTTAGAGTATCTCCAGTTGCCTGTAGTTTAC
AGGTCTGTTACGTCGCACCGTTTGGGAAATGAGACAGCTGCTTTTTGCAAAAAGCATGGTTTGGCTTTTGATTCCGATTCTCCAGTGAAAGATGAAATAATTACGAGTGGATATGAAGGAGACACAGAGACAACAGAGAAAAACATTGCCTTTTCCGCAG
CGACAGTTGAAGACTTGCATGAGGCTGGTGTTGAGGCGACCTTGGTTTTGGAAACACAAGGAAAAGAATATGATAGTGTTACTTTGTTTGTAAGAGATAGTGACAGAGAAGCTGCAAGCAATCCGCATGCACGAACTGTTGCTTTTACTCGTCATAAACG
TTTGTTAATAATTAGGGCTGAGGAAGAAATTCTTCCTTCTCTCCTTAATGGTGAATTAAACGTTAAGATAAGTGCAAACTCTCATAAATACGGTGACATAAAATATTCTCATGCCGACAACAGTAGCTCAGAGACCTAACAAATATTnnnnTATAGTGGT
TGGAGTTGGCTTGATTGGTTTGTTTGGTTATTTGATTTTCACTAATCAAAAACACTCTACCCAATCTGGCGACAACATCCACAAATTTGCTAACGGTGGTTCTTACGCGGACGGTTCTAAAAGAATAAATTACAATAGAAATAACGATCTTTCTTACGGT
TACAAGGGTTTATTTAGTGGTAGTAATAATGCCTCACTCCTTAACTTGTCTCTGCTCGGAATGTTCCTTACAGCCATGTGTGCCGGAATTTACGGAGAATACATGCGCAGAAAGGAACAATCAGGATGTATTGTTTGCCCCCCAGATTGCAAGATCTGTG
GGTAATGACTACATCTTCGGTGTGGTTTATGGAATTATAGGTGGAATACTATCAGTTTTAATCCTTATATGGTTGTTTGGTTTAACATCAGATAATGGAAGAAAGCCAGCTGTTTCTTGGTTTAGACAAGACCTTAACGGTTTTGAAATGCAAGTAACGG
AAGGTTCCCCAATTGATCCTGAAGTAGTAAAAGCTGTCCATCACTTTCAGAAATACCCCTATGGGAGTGTTCCTGTGTGGGATCACTTTGATAACATAATGGATCATGCGACCCAATGGTTAACTTCATTTATTAATTTGATGTTGTTTTTATTCATGTG
TGGTGTGGTTTTCTTTTTCGTAGGGTGTTTTTACATCCTATGATTTTCTTTTTTTTTTAAAAAAAAAAAA

>LigMV_gamma_CH_5’-RACE product
GTAAAAGATCTTTCTGCGCTTCTTTGAAGGTCTGTGGGAGTTTAGATTTACCAACAAACATTATCAATCTTTGATATTTTTCAATGATGGCTTTGCCTAGCTTCGCGCTCGCAAAGCCTAATCCCAAACTGTGTTTTAAGAAACTTCAAGTATTAGCAT
TCGTTGCGTTACTTGTGAGGTCGAAAAAAACGTTTATTCTCTCTGTCTTAAAAAGATGGTTTGGGATTTTCTTCCTAAGGAAGAATTGGTTCGCATGTTTGAAAGAGAAACTTAT

>LigMV_gamma_CH_sRNA
tccttAGcggaTTCcGaGCTTCTTTGAAGGTCTGTGGGAGTTTAGATTTACCAACAAACATTATCAATCTTTGATATTTTTCAATGATGGCTTTGCCTAGCTTCGCGCTCGCAAAGCCTAATCCCAAACTGTGTTTTAAGAAACTTCAAGTATTAGCAT
TCGTTGCGTTACTTGTGAGGTCGAAAAAAACGTTTATTCTCTCTGTCTTAAAAAGATGGTTTGGGATTTTCTTCCTAAGGAAGAATTGGTTCGCATGTTTGAAAGAGAAACTTATCTTTTCGGGTAAGGCTGTTTACTGCACGGTAACGGCTAGATGTCC
CTTAAAGTTTGACGAGGTTCGAGAAAAGGGTGTTTACAGAGATATCTTTTCGGAAAATGATGTGGTTGATGTGATGGAAATGAAGTTACAAAAACTTTTACATCATGAAGGTGACAAATGGAGGAGAAAAATTGTCTTTGATGTTCCGCGTATGTCTTCT
AAGAGAGTTCCGGTTCCTAGAATTGGTACCGTAAAGTGCATTCAAAGTTGGTTTGATGCTACTTTTCCTGGTAACTCAATCAGGTTCTCAGATTTTGATGGTTACACTGTTGCAATATCGGATCTTGCCGTTGATGTGACAGATTGTAAAATAAAAATTG
GAAAAACTTTTAAGCCGTACGTTTTTAAAAGCGCTCTGCGCCCTGAATTAAGGACATCAATGCCTGAAAAGAGACAAGGTAGTTTAGCCGAAAGTGTGTTGGCTTTGAGAAAAAGAAACTTAGCAGCACCCAGATTGCAAGGGGCTGTTAACGAATGGCA
GGTGATTGAAAATACTATGAAGAGGGCTTTGGATATTTTCTTTATTAAGGATTTGATTGATAGGTCGCACCATGCTTCCTATGAATCAGCCTTAAAATGGTGGGATAAGCAGTCAACTACTGCCCGTGCTCAACTTGCAGCTGAGACGAGACGACTTTGT
GACATCGACTTCTGTACTTACAATTTTATGATTAAAAATGATGTAAAACCGAAGTTGGATCTGACACCCCAAGTTGAGTATGCAGCTTTGCAAACGGTAGTATTTCCTGATAAAATCGTGAATGCTTTTTTCGGCCCTGTTATAAAGGAAATTAATGAGA
GGATCATTAGAGCCTTGAAACCTCATATTGTGTTTAATACACGTATGACGGCCGAAGGTTTAAATGATACTATTTCATTTCTGCCCCCCAAGTCTTACAGGTCACTTGAAATCGATTTCTCAAAATTTGACAAGTCAAAGACTGGTCTGCATATTAAATG
TGTAATAGCTTTATACAAATTGTTTGGACTGGATGGAATCCTAGAGGTGCTCTGGGAGAAATCTCAAGTACAAACATACGTAAAGGACCGAAATTTTGGTCTTGAAGCTTACCTTTTATTTCAGCAGAAATCAGGAAATTGTGACACTTACGGTTCTAAT
ACTTGGTCTGCAGCATTAGCGTTGCTAGATTGTTTGCCTTTGGAGCATTCAGTCTATGCGGTTTTTGGTGGAGACGATTCTTTGATACTATTTGAGGAGGGGTATCTTGTTAATGACCCATGCAAGCGTTTGGCTGGAACGTGGAATTTTGAATGCAAAA
TTTTTGCTTTCAATTTTCCTTCCTTTTGCGGGAAGTTTGTTGTCAGTGTTGACGACAAATATGTTTTTGTACCAGATGCTGCTAAGCTAATCACAAAACTGGGGAGAACCGACGTAAAGGATGTAGAAGCTTTAAGTGAAATTTACATTTCAATTAATGA
CAATTACAGATCCTTTAAGGATTTCCGTGTTTTGGATGAGTTGAGCGATGCTTTGGTTGATAGATATCATGCTCCTTACGACGCTTTATCTGCTTTGGTGTCTCTGTGTTACTATATATTCGATTTCGACAACTTTAAGCAGCTGTTTGGTTGTGATGGC
AATTTTGTGGATAAAATAATTGGAAAGGATTTCGATTGGTGAATCCTTGATGTATTTTTCTAACATTTTTGCTTTTTACCAAATGGCAGCCAACCCAAGAGTTTGTGAATTATGTAACATTGTTTTTGACAAGACTGATACGACTTTCTGTTCTCCTAAA
TGCGAGAAGCGTGCTAGTTTGAAGAAAGAGAGACTTCGTCGTGCTGAGCTGTTTATCAAGCACGGGCTTACAGGAAAACCTTGTGGTTTAAACAAGTATCCCGGTGAATCATGTGGTATGTATGCCTATATTGCAGAAAACCAGTTACCAGATGGCAATA
CTACCCTTACGATTTCCGAATGTTGCGGGCTTAACCACGCTGCGTATTGCTGTACGCTTGATGCTTTTGATATGAGCACACTAGAAACGAGGGCaCTCATGAAGAAGCTGGAGAGGTATCGTGATGCCTCTATGGAAAAAATGAAAGTTATAACTAACCA
GTTAAAAGCGATGAAACTAAATTCTAAGCTTTAATAAGTTTTTCTTTTCTTTAAAAAAAAAAAAAaaaaaaaaaaaaaaaaaaaaaaaaaaaaaaaaAAAAAAAAAAAAAAtAATGCCTGCTATTAAGACGGTGAAACCTATAAACGGGTTCAACGGGTG
AGATAGTGGGAATTGGTGTGTAAGCGCGTACTCCTACGTCGCTGCATCAACAATGATCTGTGGATCGATTGTTGCACACTGAGCCGGAGCTCAGCTTTGGTCCTTTAGGAGACCA

>LigMV_gamma_FR_VANA
AATGAAGGATGTGGTTGATGTGATGTAAAAGTTTTTGTAACTTCATTTCCATCACATCAACCACATCATTTTCCGAAAAGATCTTTCTGCGCTTCTTTGAAGGTCTGTGGGAGTTTAGATTTACCAACAAACATTATCAATCTTTGATATTTTTCAACG
ATGGCTTTGCCTAGCTTCGCGCTCGCAAAGCCTAATCCCAAACTGTGTTTTAAGAAACTTCAAGTATTAGCATTCGTTGCGTTACTTGTGAGGTCGAAAAAAACGTTTATTCTCTCTGTCTTAAAAAGATGGTTTGGGATTTTCTTCCTAAGGAAGAATT
GGTTCGCATGTTTGAAAGAGAAACTTATCTTTTCGGGTAAGGCTGTTTACTGCACGGTAACGGCTAGATGTCCCTTAAAGTTTGACGAGGTTCGAGAAAAAGGTGTTTACAGAGATATCTTTTCGGAAAATGATGTGGTTGATGTGATGGAAATGAAGTT
ACAAAAACTTTTACATCATGAAGGTGACAAATGGAGGAGAAAAATTGTCTTTGATGTTCCGCGTATGTCTTCTAAGAGAGTTCCGGTTCCTAGAATTGGTACCGTAAAGTGCATTCAAAGTTGGTTTGATGCTACTTTTCCTGGTAACTCAATCAGGTTC
TCAGATTTTGATGGTTACACTGTTGCAATATCGGATCTTGCCGTTGATGTGACAGATTGTAAAATAAAAATTGGAAAAACTTTTAAGCCGTACGTTTTTAAAAGCGCTCTGCGTCCTGAATTAAGGACATCAATGCCTGAAAAGAGACAAGGTAGTTTAG
CCGAAAGTGTGTTGGCTTTGAGAAAAAGAAACTTAGCAGCGCCCAGATTGCAAGGGGCTGTTAACGAATGGCAGGTGATTGAAAACACTATGAAGAGGGCTTTGGATATTTTCTTTATTAAGGATTTGATTGATAGGTCGCACCATGCTTCCTATGAATC
AGCCTTAAAATGGTGGGATAAGCAGTCAACTACTGCCCGTGCTCAACTTGCAGCAGAGACGAGACGACTTTGTGACATCGACTTCTGTACTTACAATTTTATGATCAAAAATGATGTAAAACCGAAGTTGGATCTGACACCCCAAGTTGAGTATGCAGCT
TTGCAAACGGTAGTATTTCCTGATAAAATCGTGAATGCTTTTTTCGGCCCTGTTATAAAGGAAATTAATGAGAGGATCATTAGAGCCTTGAAACCTCATATTGTGTTTAATACACGTATGACGGCCGAAGGTTTAAATGATACTATTTCATTTCTGCCCC
CCAAGTCTTACAGGTCACTTGAAATCGATTTCTCAAAATTTGACAAGTCAAAGACTGGTCTGCATATTAAATGTGTAATAGCTTTATACAAATTGTTTGGACTGGATGGAATCCTAGAGGTGCTCTGGGAGAAATCTCAAGTACAAACATACGTAAAGGA
CCGAAATTTTGGTCTTGAAGCTTACCTTTTATTTCAGCAGAAATCAGGAAATTGTGACACTTACGGTTCTAATACTTGGTCTGCAGCATTAGCGTTGCTAGATTGTTTGCCTTTGGAGCATTCAGTCTATGCGGTTTTTGGTGGAGACGATTCTTTGATA
CTATTTGAGGAGGGGTATCTTGTTAATGACCCATGCAAGCGTTTGGCTGGAACGTGGAATTTTGAATGCAAAATTTTTGCTTTCAATTTTCCTTCCTTTTGCGGGAAGTTTGTTGTCAGTGTTGACGACAAATATGTTTTTGTACCAGATGCTGCTAAGC
TAATCACAAAACTGGGGAGAACCGATGTAAAGGATGTAGAAGCTTTAAGTGAAATTTACATTTCAATTAATGACAATTACAGATCCTTTAAGGATTTCCGTGTTTTGGATGAGTTGAGCGATGCTTTGGTTGATAGATATCATGCCCCTTACGACGCTTT
ATCTGCTTTGGTGTCTCTGTGTTACTATATATTCGATTTCGACAACTTTAAGCAGCTGTTTGGTTGTGATGGCAATTTTGTGGATAAAATAATTGGAAAGGATTTCGATTGGTGAATCCTTGATGTATTTTTCTAACACTTTTGCTTTTTACCAAATGG
CAGCCAACCCGAGAGTTTGTGAATTATGTAACATTGTTTTTGACAAGACTGATACGACTTTCTGTTCTCCTAAATGCGAGAAGCGTGCTAGTTTGAAGAAAGAGAGACTTCGTCGTGCTGAGCTGTTTATCAAGCACGGGCTTACAGGAAAACCTTGTGG
TTTAAACAAGTATCCCGGTGAATCATGTGGTATGTATGCCTATATTGCAGAAAACCAGTTACCAGATGGCAATACTACCCTTACGATTTCCGAATGTTGCGGGCTTAACCACGCTGCGTATTGCTGTACGCTTGATGCTTTTGATATGAGCACACTGGAA
ACGAGGGCTCTCATGAAGAAGCTGGAGAGGTATCGTGATGCCTCTATGGAAAAAATGAAAGTTATAACTAACCAGTTAAAAGCGATGAAACTAAATTCTAAGCTTTAATAAGTTTTTCTTTTCTTTTTAAAAAAAAAAAAAAAAAAAAAAAACCCAAACCC

```
CCGCGGTTTTTG

>LigMV_gamma_AUT_VANA
CTACACGACGCTCTTCCGATCTAAGGTAGAAGCGTGGAGACTCTGGTCTGTGGGAGTTTAGATTTTACCAACAAACATTATCAATCTTTGATATTTTTCAATGATGGCTTTGCCTAGCTTCGCGCTCGCAAAGCCTAATCCCAAACTGTGTTTTAAGAAA
CTTCAAGTATTAGCATTCGTTGCGTTACTTGTGAGGTCGAAAAAAACGTTTATTCTCTCTGTCTTAAAAAGATGGTTTGGGATTTTCTTCCTAAGGAAGAATTGGTTCGCATGTTTGAAAGAGAAACTTATCTTTTCGGGTAAGGCTGTTTACTGCACGG
TAACGGCTAGATGTCCCTTAAAGTTTGACGAGGTTCGAGAAAAAGGTGTTTACAGAGATATCTTTTCGGAAAATGATGTGGTTGATGTGATGGAAATGAAGTTACAAAAACTTTTACATCATGAAGGTGACAAATGGAGGAGAAAAATTGTCTTTGATGT
TCCGCGTATGTCTTCTAAGAGAGTTCCGGTTCCTAGAATTGGTACCGTAAAGTGCATTCAAAGTTGGTTTGATGCTACTTTTCCTGGTAACTCAATCAGGTTCTCAGATTTTGATGGTTACACTGTTGCAACATCGGATCTTGCCGTTGATGTGACAGAT
TGTAAAATAAAAATTGGAAAAACTTTTAAGCCGTACGTTTTTAAAAGCGCTCTGCGCCCTGAATTAAGGACATCAATGCCTGAAAAGAGACAAGGTAGTTTAGCCGAAAGTGTGTTGGCTTTGAGAAAAAGAAACTTAGCAGCGCCCAGATTGCAAGGGG
CTGTTAACGAATGGCAGGTGATTGAAAACACTATGAAGAGGGCTTTGGATATTTTCTTTAATAAGGATTTGATTGATAGGTCGCACCATGCTTCCTATGAATCAGCCTTAAAATGGTGGGATAAGCAGTCAACTACTGCCCGTGCTCAACTTGCAGCAGA
GACGAGACGACTTTGTGACATCGACTTCTGTACTTACAATTTTATGATCAAAAATGATGTAAAACCGAAGTTGGATCTGACACCCCAAGTTGAGTATGCAGCTTTGCAAACGGTAGTATTTCCTGATAAAATCGTGAATGCTTTTTTCGGCCCTGTTATA
AAGGAAATTAATGAGAGGATCATTAGAGCCTTGAAACCTCATATTGTGTTTAATACACGTATGACGGCCGAAGGTTTAAATGATACTATTTCATTTCTGCCCCCCAAGTCTTACAGGTCACTTGAAATCGATTTCTCAAAATTTGACAAGTCAAAGACTG
GTCTGCATATTAAATGTGTAATAGCTTTATACAAATTGTTTGGACTGGATGGAATCCTAGAGGTGCTCTGGGAGAAATCTCAAGTACAAACATACGTAAAGGACCGAAATTTTGGTCTTGAAGCTTACCTTTTATTTCAGCAGAAATCAGGAAATTGTGA
CACTTACGGTTCTAATACTTGGTCTGCAGCATTAGCGTTGCTAGATTGTTTGCCTTTGGAGCATTCAGTCTATGCGGTTTTTGGTGGAGACGATTCTTTGATACTATTTGAGGAGGGGTATCTTGTTAATGACCCATGCAAGCGTTTGGCTGGAACGTGG
AATTTTGAATGCAAAATTTTTGCTTTCAATTTTCCTTCCTTTTGCGGGAAGTTTGTTGTCAGTGTTGACGACAAATATGTTTTTGTACCAGATGCTGCTAAGCTAATCACAAAACTGGGGAGAACCGACGTAAAGGATGTAGAAGCTTTAAGTGAAATTT
ACATTTCAATTAATGACAATTACAGATCCTTTAAGGATTTCCGTGTTTTGGATGAGTTGAGCGATGCTTTGGTTGATAGATATCATGCCCCTTACGACGCTTTATCTGCTTTGGTGTCTCTGTGTTACTATATATTCGATTTCGACAACTTTAAGCAGCT
GTTTGGTTGTGATGGCAATTTTGTGGATAAAATAATTGGAAAGGATTTCGATTGGTGAATCCTTGATGTATTTTTCTAACATTTTTGCTTTTTACCAAATGGCAGCCAACCCAAGAGTTTGTGAATTATGTAACATTGTTTTTGACAAGACTGATACGAC
TTTCTGTTCTCCTAAATGCGAGAAGCGTGCTAGTTTGAAGAAAGAGAGACTTCGTCGTGCTGAGCTGTTTATCAAGCACGGGCTTACAGGAAAACCTTGTGGTTTAAACAAGTATCCCGGTGAATCATGTGGTATGTATGCCTATATTGCAGAAAACCAG
TTACCAGATGGCAATACTACCCTTACGATTTCCGAATGTTGCGGGCTTAACCACGCTGCGTATTGCTGTACGCTTGATGCTTTTGATATGAGCACACTGGAAACGAGGGCTCTCATGAAGAAGCTGGAGAGGTATCGTGATGCCTCTATGGAAAAAATGA
AAGTTATAACTAACCAGTTAAAAGCGATGAAACTAAATTCTAAGCTTTAATAAGTTTTTCTTTTCTTTTTAAAAAAAAAAAAAAAAAAAAAAAAAAAAAAAAAAAAAAAAAAAAAAAAAAGATGTTTTAATAAGAGAAAAAAATAAAAAAGATAAAAAAGAA
GAAATAAATTTTAT

>LigMV_gamma_CH_dsRNA
ctggagttcagacgtgtgctcttccgatctatcagagccttCGGATCCTCCTCGCGCTTCTCTGAAGGTCTGTGGGAGTTTAGATTTTACCAACAAACATTATCAATCTTTGATATTTTTCAATGATGGCTTTGCCTAGCTTCGCGCTCGCAAAGCCTAA
TCCCAAACTGTGTTTTAAGAAACTTCAAGTATTAGCATTCGTTGCGTTACTTGTGAGGTCGAAAAAAACGTTTATTCTCTCTGTCTTAAAAAGATGGTTTGGGATTCTCTTCCTAAGGAAGAATTGGTTCGCATGTTTGAAAGAGAAACTTATCTTTTCG
GGTAAGGCTGTTTACTGCACGGTAACGGCTAGATGTCCCTTAAAGTTTGACGAGGTTCGAGAAAAAGGGTGTTTACAGAGATATCTTTTCGGAAAATGATGTGGTTGATGTGATGGAAATGAAGTTACAAAAACTTTTACATCATGAAGGTGACAAATGGA
GGAGAAAAATTGTCTTTGATGTTCCGCGTATGTCTTCTAAGAGAGTTCCGGTTCCTAGAATTGGTACCGTAAAGTGCATTCAAAGTTGGTTTGATGCTACTTTTCCTGGTAACTCAATCAGGTTCTCAGATTTTGATGGTTACACTGTTGCAATATCGGA
TCTTGCCGTTGATGTGACAGATTGTAAAATAAAAATTGGAAAAACTTTTAAGCCGTACGTTTTTAAAAGCGCTCTGCGCCCTGAATTAAGGACATCAATGCCTGAAAAGAGACAAGGTAGTTTAGCCGAAAGTGTGTTGGCTTTGAGAAAAAGAAACTTA
GCAGCGCCCAGATTGCAAGGGGCTGTTAACGAATGGCAGGTGATTGAAAACACTATGAAGAGGGCTTTGGATATTTTCTTTATTAAGGATTTGATTGATAGGTCGCACCATGCTTCCTATGAATCAGCCTTAAAATGGTGGGATAAGCAGTCAACTACTG
CCCGTGCTCAACTTGCAGCAGAGACGAGACGACTTTGTGACATCGACTTCTGTACTTACAATTTTATGATCAAAAATGATGTAAAACCGAAGTTGGATCTGACACCCCAAGTTGAGTATGCAGCTTTGCAAACGGTAGTATTTCCTGATAAAATCGTGAA
TGCTTTTTTCGGCCCTGTTATAAAGGAAATTAATGAGAGGATCATTAGAGCCTTGAAACCTCATATTGTGTTTAATACACGTATGACGGCCGAAGGTTTAAATGATACTATTTCATTTCTGCCCCCCAAGTCTTACAGGTCACTTGAAATCGATTTCTCA
AAATTTGACAAGTCAAAGACTGGTCTGCATATTAAATGTGTAATAGCTTTATACAAATTGTTTGGACTGGATGGAATCCTAGAGGTGCTCTGGGAGAAATCTCAAGTACAAACATACGTAAAGGACCGAAATTTTGGTCTTGAAGCTTACCTTTTATTTC
AGCAGAAATCAGGAAATTGTGACACTTACGGTTCTAATACTTGGTCTGCAGCATTAGCGTTGCTAGATTGTTTGCCTTTGGAGCATTCAGTCTATGCGGTTTTTGGTGGAGACGATTCTTTGATACTATTTGAGGAGGGGTATCTTGTTAATGACCCATG
CAAGCGTTTGGCTGGAACGTGGAATTTTGAATGCAAAATTTTTGCTTTCAATTTTCCTTCCTTTTGCGGGAAGTTTGTTGTCAGTGTTGACGACAAATATGTTTTTGTACCAGATGCTGCTAAGCTAATCACAAAACTGGGGAGAACCGACGTAAAGGAT
GTAGAAGCTTTAAGTGAAATTTACATTTCAATTAATGACAATTACAGATCCTTTAAGGATTTCCGTGTTTTGGATGAGTTGAGCGATGCTTTGGTTGATAGATATCATGCCCCTTACGACGCTTTATCTGCTTTGGTGTCTCTGTGTTACTATATTTTCG
ATTTCGACAACTTTAAGCAGCTGTTTGGTTGTGATGGCAATTTTGTGGATAAAATAATTGGAAAGGATTTCGATTGGTGAATCCTTGATGTATTTTTCTAACATTTTTGCTTTTTACCAAATGGCAGCCAACCCAAGAGTTTGTGAATTATGTAACATTG
TTTTTGACAAGACTGATACGACTTTCTGTTCTCCTAAATGCGAGAAGCGTGCTAGTTTGAAGAAAGAGAGACTTCGTCGTGCTGAGCTGTTTATCAAGCACGGGCTTACAGGAAAACCTTGTGGTTTAAACAAGTATCCCGGTGAATCATGTGGTATGTA
TGCCTTTATTGCAGAAAACCAGTTACCAGATGGCAATACTACCCTTACGATTTCCGAATGTTGCGGGCTTAACCACGCTGCGTATTGCTGTACGCTTGATGCTTTTGATATGAGCACACTGGAAACGAGGGCTCTCATGAAGAAGCTGGAGAGGTATCGT
GATGCCTCTATGGAAAAAATGAAAGTTATAACTAACCAGTTAAAAGCGATGAAAC
```

CLUSTAL multiple sequence alignment by MUSCLE (3.8)

```
LigMV_alpha_CH_sRNA       acGTAATGTTTACTGCGGTAGTTTGAGTACTGCGACAGTGGTTTTGCATTAACAGTTAAT
LigMV_alpha_CH_dsRNA      --GTAATGTTTACTGCGGTAGTTTGAGTACTGCGACAGTGGTTTTGCATTAACAGTTAAT
                            **********************************************************

LigMV_alpha_CH_sRNA       CCACTTCAGGTTTTGTTCTTTTCAACATTTTCATTCCGTGGTCATAATCATGGCTAGCGA
LigMV_alpha_CH_dsRNA      CCACTTCAGGTTTTGTTCTTTTCAACATTTTCATTCCGTGGTCATAATCATGGCTAGCGA
                          ************************************************************

LigMV_alpha_CH_sRNA       TGAGATCGTACGTAATTTGATTTCGAGGGAGGAAGTAATGGGTAATCTTATTAGTACAGC
LigMV_alpha_CH_dsRNA      TGAGATCGTACGTAATTTGATTTCGAGGGAGGAAGTAATGGGTAATCTTATTAGTACAGC
                          ************************************************************

LigMV_alpha_CH_sRNA       ATCGTCATCGGTGAGATCACCTTTGCACGACACACTGTGCTCGCACGTTAATGCCGTTGT
LigMV_alpha_CH_dsRNA      ATCGTCATCGGTGAGATCACCTTTGCACGACACACTGTGCTCGCACGTTAATGCCGTTGT
                          ************************************************************

LigMV_alpha_CH_sRNA       GAATTCCTTGGAAAAGAAGAACGTTGGTCGAAGGCAGATTGATGTGAGGAGAAATTTAAC
LigMV_alpha_CH_dsRNA      GAATTCCTTGGAAAAGAAGAACGTTGGTCGAAGGCAGATTGATGTGAGGAGAAATTTAAC
                          ************************************************************

LigMV_alpha_CH_sRNA       ATCTGATGAGTTACAGATGTTAATAAAGGATTATCCTGAATATTCTGTGACATCATCTGC
LigMV_alpha_CH_dsRNA      ATCTGATGAGTTACAGATGTTAATAAAGGATTATCCTGAATATTCTGTGACATCATCTGC
                          ************************************************************

LigMV_alpha_CH_sRNA       TTGTGAGAGTGGAACTCATAGTATGGCAGCATGCTATAGATTTTTAGAAACTGAATACTT
LigMV_alpha_CH_dsRNA      TTGTGAGAGTGGAACTCATAGTATGGCAGCATGCTATAGATTTTTAGAAACTGAATACTT
                          ************************************************************

LigMV_alpha_CH_sRNA       ATTAGATTTTGTACCAAAAAAAGAAACGACGATTTGGGATATTGGTGGGAACTGGTTCTC
LigMV_alpha_CH_dsRNA      ATTAGATTTTGTACCAAAAAAAGAAACGACGATTTGGGATATTGGTGGGAACTGGTTCTC
                          ************************************************************

LigMV_alpha_CH_sRNA       GCACATGAAATTCAGGAGTGATTTGGATATACATTGTTGCTGTCCCATCCTATCTTTGCG
LigMV_alpha_CH_dsRNA      GCACATGAAATTCAGGAGTGATTTGGATATACATTGTTGCTGTCCCATCCTATCTTTGCG
                          ************************************************************

LigMV_alpha_CH_sRNA       CGATGCAGAACGCGTTGAAACTAGATATATGAGTATGCAGAGATATATGAGAGGTGATAA
LigMV_alpha_CH_dsRNA      CGATGCAGAACGCGTTGAAACTAGATATATGAGTATGCAGAGATATATGAGAGGTGATAA
                          ************************************************************

LigMV_alpha_CH_sRNA       AGACAAATCTTTGGCTTTATTTTCAAAATATAGCTCTGTACTCGTAGATCAAGCGAGGCG
LigMV_alpha_CH_dsRNA      AGACAAATCTTTGGCTTTATTTTCAAAATATAGCTCTGTACTCGTAGATCAAGCGAGGCG
                          ************************************************************

LigMV_alpha_CH_sRNA       CAAGGAGTGCATTGAGTCGGGAACTTTGAATCCTTCACAGTTGGATGGAGAATTATTTTG
LigMV_alpha_CH_dsRNA      CAAGGAGTGCATTGAGTCGGGAACTTTGAATCCTTCACAGTTGGATGGAGAATTATTTTG
                          ************************************************************

LigMV_alpha_CH_sRNA       TGAGAATACGTTTCAGGAGTGTGTTCGAAAAGCACCCGAGGGCTCATTGCCTGTGGCGAT
LigMV_alpha_CH_dsRNA      TGAGAATACGTTTCAGGAGTGTGTTCGAAAAGCACCCGAGGGCTCATTGCCTGTGGCGAT
                          ************************************************************

LigMV_alpha_CH_sRNA       TGCAGTTCATAGTGTGTATGATATTGAGGTTGAGAGTTTGGTAAGAGCTTTGAAACAAAA
LigMV_alpha_CH_dsRNA      TGCAGTTCATAGTGTGTATGATATTGAGGTTGAGAGTTTGGTAAGGGCTTTGAAACAAAA
                          ********************************************* **************

LigMV_alpha_CH_sRNA       GGGGATCACTCAGATGTACGGATGTATGTTGTTTCCACCAAGTATCTTAGTTGGAGAAGT
LigMV_alpha_CH_dsRNA      GGGGATCACTCAGATGTACGGATGTATGTTGTTTCCACCAAGTATCTTAGTTGGAGAAGT
                          ************************************************************

LigMV_alpha_CH_sRNA       AAAAGGTGATTTGCCAGCGGTAAAAGGAAAGTTTAGTATAGAGAAAGGGAAAATTTCTTT
LigMV_alpha_CH_dsRNA      GAAAGGTGATTTGCCAGCGGTAAAAGGAAAGTTAAATAGAGA------------------
                           ******************************** * ** ***

LigMV_alpha_CH_sRNA       CTTTTTTCTTAACGATCCAAACGCCGGTTATACTCACGATTTAAAAGACTACCTAAGATA
LigMV_alpha_CH_dsRNA      ------------------------------------------------------------


LigMV_alpha_CH_sRNA       TTTTGAAGACACATATATCACTATACAAAACGGTGTGTTCTCGATTGAGCTTCTTCAAAT
LigMV_alpha_CH_dsRNA      -----------------------------------TGTTC--------------------
                                                             *****

LigMV_alpha_CH_sRNA       GAGAGGGGATACCATGTTTTTTAAGATTACTGATGTCACCGTAGCCGCGTACCATTTGCG
LigMV_alpha_CH_dsRNA      ------------------TTTTATGATTACTGATGTCACCGAAGCCGCGTACCATTTGCG
                                            ***** ***************** ******************

LigMV_alpha_CH_sRNA       GTATAGGGGTGTCAAGTATGATGAAACTTTCAAGTGTATTCCTATGGTTGCTCAGACCTC
LigMV_alpha_CH_dsRNA      GTATAGGGGTGTCAAGTATGATGAAACTTTCAAGTGTATTCCTATGGTTGCTCAGACCTC
                          ************************************************************

LigMV_alpha_CH_sRNA       CGTTGTTGTTCCGATTTTTTCTTGGAATGATCGGGGAATAGAGATTGATACAGCTCTAAT
LigMV_alpha_CH_dsRNA      CGTTGTTGTTCCGATTTTTTCTTGGAATGATCGGGGAATAGAGATTGATACAGCTCTAAT
                          ************************************************************

LigMV_alpha_CH_sRNA       TCCGAGGAATATTGTTGAACAGGGAGCGTCGTATATGATGAAAAATAAGGAGAAAGATCT
```

```
LigMV_alpha_CH_dsRNA      TCCGAGGAATATTGTTGAACAGGGAGCGTCGTATATGATGAAAAATAAGGAGAAAGATCT
                          ************************************************************

LigMV_alpha_CH_sRNA       GACGGTAAGTGTGTTGAAAAACTACTTATCTTCGATTAATAATTCTTATATTTTTAACGG
LigMV_alpha_CH_dsRNA      GACGGTAAGTGTGTTGAAAAACTACTTATCTTCGATTAATAATTCTTATATTTTTAACGG
                          ************************************************************

LigMV_alpha_CH_sRNA       CTCTCAATGTCGACAAGGAGAAAAAAATTGGACCAAGTCTTATTTCACAGCTTGCGGTAAC
LigMV_alpha_CH_dsRNA      CTCTCAATGTCGACAAGGAGAAAAAAATTGGACCAAGTCTTATTTCACAGCTTGCGGTAAC
                          ************************************************************

LigMV_alpha_CH_sRNA       GTTATACTTGAGGGAAAAGGTTTACAGAAATCGAGAGAACCAAGTGGTAAAACATTTTGA
LigMV_alpha_CH_dsRNA      GTTATACTTGAGGGAAAAGGTTTACAGAAATCGAGAGAACCAAGTGGTAAAACATTTTGA
                          ************************************************************

LigMV_alpha_CH_sRNA       AGACTTGTTTATGGATGACATTTCTTTAAGTGATTTATTTGTTGGAGCTCTTTGGAAAAA
LigMV_alpha_CH_dsRNA      AGACTTGTTTATGGATGACATTTCTTTAAGTGATTTATTTGCTGGAGCTCTTTGGAAAAA
                          ***************************************** ******************

LigMV_alpha_CH_sRNA       TAATCGCACCGCTGTTGCTCGCATTTATAGAGTGATGAAGATGAGCATGGCCAAGTGGTT
LigMV_alpha_CH_dsRNA      TAATCGCACCGCTGTTGCTCGCATTTATAGAGTGATGAAGATGAGCATGGCCAAGTGGTT
                          ************************************************************

LigMV_alpha_CH_sRNA       TGGTCTGACAGTAGATCCGAGTTCTTTTGACATTTGTGACCCTCCGCTTTTCGTAGAAAT
LigMV_alpha_CH_dsRNA      TGGTCTGACAGTAGATCCGAGTTCTTTTGACATTTGTGACCCTCCGCTTTTCGTAGAAAT
                          ************************************************************

LigMV_alpha_CH_sRNA       ACGAGACAGATGGAAGGTTCAAGCCAGAGGTGACATCAAATTAAGCTCGTTGTATGACTT
LigMV_alpha_CH_dsRNA      ACGAGATAGATGGAAGGTTCAAGCCAGAGGTGACATCAAATTAAGCTCGTTGTATGACTT
                          ****** *****************************************************

LigMV_alpha_CH_sRNA       TGAGGATGATGCGCGGGAGTTTGATAAAAGAGTGGAAGAGAAGCGACGTTTGGCTGAAGA
LigMV_alpha_CH_dsRNA      TGAGGATGATGCACGGGAGTTTGATAAAAGAGTGGAAGAGAAGCGACGTTTGGCTGAAGA
                          ************ ***********************************************

LigMV_alpha_CH_sRNA       TATTGTCAATAAAGTTACAGGGGTTGCAAGCCCTGGAAGTCCTACGAATATGGAGAACCT
LigMV_alpha_CH_dsRNA      TATTGTCAATAAAGTTACAGGGGTTGCAAGCCCTGGAAGTCCTACGAATATGGAGAACCT
                          ************************************************************

LigMV_alpha_CH_sRNA       ACAGAAGCTAAATTCAATTTCACGCCTGTTTCTAGCTGCTACTGTGGTTAACAAATTCAA
LigMV_alpha_CH_dsRNA      ACAGAAGCTAAATTCAATTTCACGCCTGTTTCTAGCTGCTACTGTGGTTAACAAATTCAA
                          ************************************************************

LigMV_alpha_CH_sRNA       GACTTCGTCTTTCGATAGTTTTTCAGATACGGCTTCCGTCCATTCGGAGTCTTCATTGAC
LigMV_alpha_CH_dsRNA      GACTTCGTCTTTCGATAGTTTTTCAGATACGGCTTCCGTCCATTCGGAGTCTTCATTGAC
                          ************************************************************

LigMV_alpha_CH_sRNA       TTTACCTTCTTCACCGACATTATCTATCGATGGTCGACTCCCTATGTGGGAAGTAGCTCA
LigMV_alpha_CH_dsRNA      TTTACCTTCTTCACCGACATTATCTATCGATGGTCGACTCCCTATGTGGGAAGTAGCTCA
                          ************************************************************

LigMV_alpha_CH_sRNA       TGCAGATGTGTTGAATTTTCAACAAATTAATGGCAACATGTTAAAGAGAACTGGTGTACC
LigMV_alpha_CH_dsRNA      TGCAGATGTGTTGAATTTTCAACAAATTAATGGCAACATGTTAAAGAGAACTGGTGTACC
                          ************************************************************

LigMV_alpha_CH_sRNA       AGATAGACCAGTAGAAAGCAAAGATTATTTTGAGAACTCTAGAGCCGAGTTTTTATACTA
LigMV_alpha_CH_dsRNA      AGATAGACCAGTAGAAAGCAAAGATTATTTTGAGAACTCTAGAGCCGAGTTTTTATACTA
                          ************************************************************

LigMV_alpha_CH_sRNA       TTTGTTGTCTAGTGTTATAAGTGAAAGAGCCCAAATGATGGACATTGTTCATAGTTACAG
LigMV_alpha_CH_dsRNA      TTTGTTGTCTAGTGTTATAAGTGAAAGAGCCCAAATGATGGACATTGTTCATAGTTACAG
                          ************************************************************

LigMV_alpha_CH_sRNA       GCTCGGTCATTTATTCACTGACAAGGTAGTGGCTCCTCTAACATCGTATATCTACTCTTG
LigMV_alpha_CH_dsRNA      GCTCGGTCATTTATTCACTGACAAGGTAGTGGCTCCTCTAACATCGTATATCTACTCTTG
                          ************************************************************

LigMV_alpha_CH_sRNA       TGAGAGGGCGGATTGGGTGTTGAGGACTCCTCGAGCGAATGATGTGGGACATAACTTTGC
LigMV_alpha_CH_dsRNA      TGAGAGGGCGGATTGGGTGTTGAGGACTCCTCGAGCGAATGATGTGGGACATAACTTTGC
                          ************************************************************

LigMV_alpha_CH_sRNA       GGTGCATTTCAATTTCAAAGGAGAGAGTAAAGAGACAGATGAGTACCTAGAGTATTGTGA
LigMV_alpha_CH_dsRNA      GGTGCATTTCAATTTCAAAGGAGAGAGTAAAGAGACAGATGAGTACCTAGAGTATTGTGA
                          ************************************************************

LigMV_alpha_CH_sRNA       GATCGTTGATGTGAGATGGGACAAGTCTGGAGGTTTTATGTCAAACATTCCAATCGTACC
LigMV_alpha_CH_dsRNA      GATCGTTGATGTGAGATGGGACAAGTCTGGAGGTTTTATGTCAAACATTCCAATCGTACC
                          ************************************************************

LigMV_alpha_CH_sRNA       AAAGTCTACCGGTTTTTACTTGTTTTGTGAGCCAACTAAATTGTGTAATAATTATCTTAT
LigMV_alpha_CH_dsRNA      AAAGTCTACCGGTTTTTACTTGTTTTGTGAGCCGACTAAATTGTGTAATAATTATCTTAT
                          ********************************* **************************

LigMV_alpha_CH_sRNA       TTACAACAATTTAGTAGATCTTTTTTCTTCGTGTAAGAATAGAAAGGTCACCTTTGAACT
LigMV_alpha_CH_dsRNA      TTACAACAATTTAGTAGATCTTTTTTCTTCGTGTAAGAATAGAAAGGTCACCTTTGAACT
                          ************************************************************

LigMV_alpha_CH_sRNA       CATAGAGGGTGTTCCGGGTTGCGGAAAATCCACAATGATTTTGGAGACTGCAAACATGAG
LigMV_alpha_CH_dsRNA      CATAGAGGGTGTTCCGGGTTGCGGAAAATCCACAATGATTTTGGAGACTGCAAACATGAG
                          ************************************************************

LigMV_alpha_CH_sRNA       AAGTGAGGCCGTTGTAGGTGAGGGAAGACAAGCAATGGATGATCTGAGAAATAGATTCGT
LigMV_alpha_CH_dsRNA      AAGTGAGGCCGTTGTAGGTGAGGGAAGACAAGCAATGGATGATCTGAGAAATAGATTCGT
                          ************************************************************

LigMV_alpha_CH_sRNA       GAAACAGAAGAAGTGGTCACAAAACACAGCTAATAAGAAAGTGAGAACTTTAGATAGCGT
LigMV_alpha_CH_dsRNA      GAAACAGAAGAAGTGGTCACAAAACACAGCTAATAAGAAAGTGAGAACTTTAGATAGCGT
                          ************************************************************

LigMV_alpha_CH_sRNA       TCTTTTGGCTACTCAACCTGACAGAATCCCGAAGGTGAAAGGCTTCCATTTCGATGAGGC
LigMV_alpha_CH_dsRNA      TCTTTTGGCTACTCAACCTGACAGAATCCCGAAGGTGAAAGGCTTCCATTTCGATGAGGC
                          ************************************************************

LigMV_alpha_CH_sRNA       ATTAAAGGTGCATTTTGGTGCTATACTTTTTTGTGCAGATAAAATGGGAAGCAATTATGT
LigMV_alpha_CH_dsRNA      ATTAAAGGTGCATTTTGGTGCTATACTTTTTTGTGCAGATAAAATGGGAAGCAATTATGT
                          ************************************************************

LigMV_alpha_CH_sRNA       GATCGGACAAGGTGACAGAGCTCAATTGCCTATGATTAATCGAGTTGAGGGCATATCTCT
LigMV_alpha_CH_dsRNA      GATCGGACAAGGTGACAGAGCTCAATTGCCTATGATTAATCGAGTTGAGGGCATATCTCT
                          ************************************************************

LigMV_alpha_CH_sRNA       GAAGTTTAGCACGCCAGATTACTCTCAAGTGAAAGTGACACCGAAATTGTTATCATATAG
LigMV_alpha_CH_dsRNA      GAAGTTTAGCACGCCAGATTACTCTCAAGTGAAAGTGACACCGAAATTGTTATCATATAG
                          ************************************************************

LigMV_alpha_CH_sRNA       AATTCCGGGGGATGTTGCATATTACTTGAGTAAGAGGGCCTGTTATAAGGACAGGGGTAT
LigMV_alpha_CH_dsRNA      AATTCCGGGGGATGTTGCATATTACTTGAGTAAGAGGGCCTGTTATAAGGACAGGGGTAT
                          ************************************************************

LigMV_alpha_CH_sRNA       TCCACAGGTTGTAACTACAAAAAATCAGGTCACAAGGTCAATGTTCGCGAGGGGGCAGAA
LigMV_alpha_CH_dsRNA      TCCACAGGTTGTAACTACAAAAAATCAGGTCACAAGGTCAATGTTCGCGAGGGGGCAGAA
                          ************************************************************

LigMV_alpha_CH_sRNA       CACAACTGAGAGGTTTGTTAGTCTACAAGACTTACCGATCGTTTTGGGTGCGCAGTATCT
LigMV_alpha_CH_dsRNA      CACAGCTGAGAGGTTTGTTAGTCTACAAGACTTACCGATCGTTTTGGGTGCGCAGTATCT
                          **** *******************************************************

LigMV_alpha_CH_sRNA       TACTTTTCTGCAAGCAGAAAAGGAGACAATTATTTCGCATTTAAAGGCAAAAGGTGTGAA
```

```
LigMV_alpha_CH_dsRNA      TACTTTTCTGCAAGCAGAAAAGGAGACAATTATTTCGCATTTAAAGGCAAAAGGTGTGAA
                          ************************************************************

LigMV_alpha_CH_sRNA       GGATTTCAATGTGAATACCATTCATGAATCTCAGGGAGGAACATTTAAAAATGTTATTCT
LigMV_alpha_CH_dsRNA      GGATTTCAATGTGAATACCATTCATGAATCTCAGGGAGGAACATTTAAAAATGTTATTCT
                          ************************************************************

LigMV_alpha_CH_sRNA       TGTTCGTTTGCAAAGAACTGAGAATGAGATTTATCCTGGTGGTGTGAGAAGTTCAAGTTA
LigMV_alpha_CH_dsRNA      TGTTCGTTTGCAAAGAACTGAGAATGAGATTTATCCTGGTGGTGTGAGAAGTTCAAGTTA
                          ************************************************************

LigMV_alpha_CH_sRNA       CGTTGTTGTTGGAATATCTCGACATACGGAAACTTTCACATACTGTAGTGTCGTTGATGA
LigMV_alpha_CH_dsRNA      CGTTGTTGTTGGAATATCTCGACATACGGAAACTTTCACATACTGTAGTGTCGTTGATGA
                          ************************************************************

LigMV_alpha_CH_sRNA       TCGTTTGTTACTGGACATAGCAAATGAAGATGGTTTAAAAAATACTCCGATTCGGACGTT
LigMV_alpha_CH_dsRNA      TCGTTTGTTACTGGACATAGCAAATGAAGATGGTTTAAAAAATACTCCGAYTCGGACGTT
                          ************************************************** *********

LigMV_alpha_CH_sRNA       TCAAGAGTACATAATAAAATAAATAAGTTTTCTTTTTTTAAAAAAAAAAAAAAAAAAA
LigMV_alpha_CH_dsRNA      TCAAGAGTACATAATAAAATAAATAAGTTT------------------------------
                          ******************************
```

CLUSTAL multiple sequence alignment by MUSCLE (3.8)

```
LigMV_alpha_AUT          ----GAGACTCTGGTAATGTTTACTGCGGTAGTTTGAGTACTGCGACAGTGGTTTTGCAT
LigMV_alpha_CH_sRNA      -----------acGTAATGTTTACTGCGGTAGTTTGAGTACTGCGACAGTGGTTTTGCAT
LigMV_alpha_FR           CAGCGCCCCCCTGGTAATGTTTACTGCGGTAGTTTGAGTACTGCGACAGTGGTTTTGCAT
                                      ***********************************************

LigMV_alpha_AUT          TAACAGTTAATCCACTTCAGGTTTTGTTCTTTTCAACATTTTCATTCCGTGGTCATAATC
LigMV_alpha_CH_sRNA      TAACAGTTAATCCACTTCAGGTTTTGTTCTTTTCAACATTTTCATTCCGTGGTCATAATC
LigMV_alpha_FR           TAACAGTTAATCCACTTCAGGTTTTGTTCTTTTCAACATTTTCATTCCGTGGTCATAATC
                         ************************************************************

LigMV_alpha_AUT          ATGGCTAGCGATGAGATCGTACGTAATTTGATTTCGAGGGAGGAAGTAATGGGTAATCTT
LigMV_alpha_CH_sRNA      ATGGCTAGCGATGAGATCGTACGTAATTTGATTTCGAGGGAGGAAGTAATGGGTAATCTT
LigMV_alpha_FR           ATGGCTAGCGATGAGATCGTACGTAATTTGATTTCGAGGGAGGAAGTAATGGGTAATCTT
                         ************************************************************

LigMV_alpha_AUT          ATTAGTACAGCATCGTCATCGGTGAGATCACCTTTGCACGACACACTGTGCTCGCACGTT
LigMV_alpha_CH_sRNA      ATTAGTACAGCATCGTCATCGGTGAGATCACCTTTGCACGACACACTGTGCTCGCACGTT
LigMV_alpha_FR           ATTAGTACAGCATCGTCATCGGTGAGATCACCTTTGCACGACACACTGTGCTCGCACGTT
                         ************************************************************

LigMV_alpha_AUT          AATGCCGTTGTGAATTCCTTGGAAAAGAAGAACGTTGGTCGAAGGCAGATTGATGTGAGG
LigMV_alpha_CH_sRNA      AATGCCGTTGTGAATTCCTTGGAAAAGAAGAACGTTGGTCGAAGGCAGATTGATGTGAGG
LigMV_alpha_FR           AATGCCGTTGTGAATTCCTTGGAAAAGAAGAACGTTGGTCGAAGGCAGATTGATGTGAGG
                         ************************************************************

LigMV_alpha_AUT          AGAAATTTAACATCTGATGAGTTACAGATGTTAATAAAGGATTATCCTGAATATTCTGTG
LigMV_alpha_CH_sRNA      AGAAATTTAACATCTGATGAGTTACAGATGTTAATAAAGGATTATCCTGAATATTCTGTG
LigMV_alpha_FR           AGAAATTTAACATCTGATGAGTTACAGATGTTAATAAAGGATTATCCTGAATATTCTGTG
                         ************************************************************

LigMV_alpha_AUT          ACATCATCTGCTTGTGAGAGTGGAACTCATAGTATGGCAGCATGCTATAGATTTTTAGAA
LigMV_alpha_CH_sRNA      ACATCATCTGCTTGTGAGAGTGGAACTCATAGTATGGCAGCATGCTATAGATTTTTAGAA
LigMV_alpha_FR           ACATCATCTGCTTGTGAGAGTGGAACTCATAGTATGGCAGCATGCTATAGATTTTTAGAA
                         ************************************************************

LigMV_alpha_AUT          ACTGAATACTTATTAGATTTTGTACCAAAAAAAGAAACGACGATTTGGGATATTGGTGGG
LigMV_alpha_CH_sRNA      ACTGAATACTTATTAGATTTTGTACCAAAAAAAGAAACGACGATTTGGGATATTGGTGGG
LigMV_alpha_FR           ACTGAATACTTATTAGATTTTGTACCAAAAAAAGAAACGACGATTTGGGATATTGGTGGG
                         ************************************************************

LigMV_alpha_AUT          AACTGGTTCTCGCACATGAAATTCAGGAGTGATTTGGATATACATTGTTGCTGTCCCATT
LigMV_alpha_CH_sRNA      AACTGGTTCTCGCACATGAAATTCAGGAGTGATTTGGATATACATTGTTGCTGTCCCATC
LigMV_alpha_FR           AACTGGTTCTCGCACATGAAATTCAGGAGTGATTTGGATATACATTGTTGCTGTCCCATC
                         ***********************************************************

LigMV_alpha_AUT          CTATCTTTGCGCGATGCAGAACGCGTTGAAACTAGATATATGAGTATGCAGAGATATATG
LigMV_alpha_CH_sRNA      CTATCTTTGCGCGATGCAGAACGCGTTGAAACTAGATATATGAGTATGCAGAGATATATG
LigMV_alpha_FR           CTATCTTTGCGCGATGCAGAACGCGTTGAAACTAGATATATGAGTATGCAGAGATATATG
                         ************************************************************

LigMV_alpha_AUT          AGAGGTGATAAAGACAAATCTTTGGCTTTATTTTCAAAATATAGCTCTGTACTCGTAGAT
LigMV_alpha_CH_sRNA      AGAGGTGATAAAGACAAATCTTTGGCTTTATTTTCAAAATATAGCTCTGTACTCGTAGAT
LigMV_alpha_FR           AGAGGTGATAAAGACAAATCTTTGGCTTTATTTTCAAAATATAGCTCTGTACTCGTAGAT
                         ************************************************************

LigMV_alpha_AUT          CAAGCGAGGCGCAAGGAGTGCATTGAGTCGGGAACTTTGAATCCTTCACAGTTGGATGGA
LigMV_alpha_CH_sRNA      CAAGCGAGGCGCAAGGAGTGCATTGAGTCGGGAACTTTGAATCCTTCACAGTTGGATGGA
LigMV_alpha_FR           CAAGCGAGGCGCAAGGAGTGCATTGAGTCGGGAACTTTGAATCCTTCACAGTTGGATGGA
                         ************************************************************

LigMV_alpha_AUT          GAATTATTTTGTGAGAATACGTTTCAGGAGTGTGTTCGAAAAGCACCCGAGGGCTCATTG
LigMV_alpha_CH_sRNA      GAATTATTTTGTGAGAATACGTTTCAGGAGTGTGTTCGAAAAGCACCCGAGGGCTCATTG
LigMV_alpha_FR           GAATTATTTTGTGAGAATACGTTTCAGGAGTGTGTTCGAAAAGCACCCGAGGGCTCATTG
                         ************************************************************

LigMV_alpha_AUT          CCTGTGGCGATTGCAGTTCATAGTGTGTATGATATTGAGGTTGAGAGTTTGGTAAGAGCT
LigMV_alpha_CH_sRNA      CCTGTGGCGATTGCAGTTCATAGTGTGTATGATATTGAGGTTGAGAGTTTGGTAAGAGCT
LigMV_alpha_FR           CCTGTGGCGATTGCAGTTCATAGTGTGTATGATATTGAGGTTGAGAGTTTGGTAAGAGCT
                         ************************************************************

LigMV_alpha_AUT          TTGAAACAAAAGGGGATCACTCAGATGTACGGATGTATGTTGTTTCCACCAAGTATCTTA
LigMV_alpha_CH_sRNA      TTGAAACAAAAGGGGATCACTCAGATGTACGGATGTATGTTGTTTCCACCAAGTATCTTA
LigMV_alpha_FR           TTGAAACAAAAGGGGATCACTCAGATGTACGGATGTATGTTGTTTCCACCAAGTATCTTA
                         ************************************************************

LigMV_alpha_AUT          GTTGGAGAAGTAAAAGGTGATTTGCCAGCGGTAAAAGGAAAGTTTAGTATAGAGAAAGGG
LigMV_alpha_CH_sRNA      GTTGGAGAAGTAAAAGGTGATTTGCCAGCGGTAAAAGGAAAGTTTAGTATAGAGAAAGGG
LigMV_alpha_FR           GTTGGAGAAGTAAAAGGTGATTTGCCAGCGGTAAAAGGAAAGTTTAGTATAGAGAAAGGG
                         ************************************************************

LigMV_alpha_AUT          AAAATTTCTTTCTTTTTTCTTAACGATCCGAACGCCGGTTATACTCACGATTTAAAAGAC
LigMV_alpha_CH_sRNA      AAAATTTCTTTCTTTTTTCTTAACGATCCAAACGCCGGTTATACTCACGATTTAAAAGAC
LigMV_alpha_FR           AAAATTTCTTTCTTTTTTCTTAACGATCCAAACGCCGGTTATACTCACGATTTAAAAGAC
                         ***************************** ******************************

LigMV_alpha_AUT          TACCTAAGATATTTTGAAGACACATATATCACTATACAAAATGGTGTGTTCTCGATTGAG
LigMV_alpha_CH_sRNA      TACCTAAGATATTTTGAAGACACATATATCACTATACAAAACGGTGTGTTCTCGATTGAG
LigMV_alpha_FR           TACCTAAGATATTTTGAAGACACATATATCACTATACAAAATGGTGTGTTCTCGATTGAG
                         ***************************************** ******************

LigMV_alpha_AUT          CTTCTTCAAATGAGAGGGGATACCATGTTTTTTAAGATTACTGATGTCACCGTAGCCGCG
LigMV_alpha_CH_sRNA      CTTCTTCAAATGAGAGGGGATACCATGTTTTTTAAGATTACTGATGTCACCGTAGCCGCG
LigMV_alpha_FR           CTTCTTCAAATGAGAGGGGATACCATGTTTTTTAAGATTACTGATGTCACCGTAGCCGCG
                         ************************************************************

LigMV_alpha_AUT          TACCATTTGCGGTATAGGGGTGTCAAGTATGATGAAACTTTCAAGTGTATTCCTATGGTT
LigMV_alpha_CH_sRNA      TACCATTTGCGGTATAGGGGTGTCAAGTATGATGAAACTTTCAAGTGTATTCCTATGGTT
LigMV_alpha_FR           TACCATTTGCGGTATAGGGGTGTCAAGTATGATGAAACTTTCAAGTGTATTCCTATGGTT
                         ************************************************************

LigMV_alpha_AUT          GCTCAGACCTCCGTTGTTGTTCCGATTTTTTCTTGGAATGATCGGGGAATAGAGATTGAT
LigMV_alpha_CH_sRNA      GCTCAGACCTCCGTTGTTGTTCCGATTTTTTCTTGGAATGATCGGGGAATAGAGATTGAT
```

```
LigMV_alpha_FR           GCTCAGACCTCCGTTGTTGTTCCGATTTTTTCTTGGAATGATCGGGGAATAGAGATTGAT
                         ************************************************************

LigMV_alpha_AUT          ACAGCTCTAATTCCGAGGAATATTGTTGAACAGGGAGCGTCGTATATGATGAAAAATAAG
LigMV_alpha_CH_sRNA      ACAGCTCTAATTCCGAGGAATATTGTTGAACAGGGAGCGTCGTATATGATGAAAAATAAG
LigMV_alpha_FR           ACAGCTCTAATTCCGAGGAATATTGTTGAACAGGGAGCGTCGTATATGATGAAAAATAAG
                         ************************************************************

LigMV_alpha_AUT          GAGAAAGATCTGACGGTAAGTGTGTTGAAAAACTACTTATCTTCGATTAATAATTCTTAT
LigMV_alpha_CH_sRNA      GAGAAAGATCTGACGGTAAGTGTGTTGAAAAACTACTTATCTTCGATTAATAATTCTTAT
LigMV_alpha_FR           GAGAAAGATCTGACGGTAAGTGTGTTGAAAAACTACTTATCTTCGATTAATAATTCTTAT
                         ************************************************************

LigMV_alpha_AUT          ATTTTCAACGGCTCTCAATGTCGACAAGGAGAAAAAATTGGACCAAGTCTTATTTCACAG
LigMV_alpha_CH_sRNA      ATTTTTAACGGCTCTCAATGTCGACAAGGAGAAAAAATTGGACCAAGTCTTATTTCACAG
LigMV_alpha_FR           ATTTTTAACGGCTCTCAATGTCGACAAGGAGAAAAAATTGGACCAAGTCTTATTTCACAA
                         ***** *****************************************************

LigMV_alpha_AUT          CTTGCGGTAACGTTATACTTGAGGGAAAAGGTTTACAGAAATCGAGAGAACCAAGTGGTA
LigMV_alpha_CH_sRNA      CTTGCGGTAACGTTATACTTGAGGGAAAAGGTTTACAGAAATCGAGAGAACCAAGTGGTA
LigMV_alpha_FR           CTTGCGGTAACGTTATACTTGAGGGAAAAGGTTTACAGAAATCGAGAGAACCAAGTGGTA
                         ************************************************************

LigMV_alpha_AUT          AAACATTTTGAAGACTTGTTTATGGATGACATTTCTTTAAGTGATTTATTTGTTGGAGCT
LigMV_alpha_CH_sRNA      AAACATTTTGAAGACTTGTTTATGGATGACATTTCTTTAAGTGATTTATTTGTTGGAGCT
LigMV_alpha_FR           AAACATTTTGAAGACTTGTTTATGGATGACATTTCTTTAAGTGATTTATTTGTTGGAGCT
                         ************************************************************

LigMV_alpha_AUT          CTTTGGAAAAATAATCGCACCGCTGTTGCTCGCATTTATAGAGTGATGAAGATGAGCATG
LigMV_alpha_CH_sRNA      CTTTGGAAAAATAATCGCACCGCTGTTGCTCGCATTTATAGAGTGATGAAGATGAGCATG
LigMV_alpha_FR           CTTTGGAAAAATAATCGCACCGCTGTTGCTCGCATTTATAGAGTGATGAAGATGAGCATG
                         ************************************************************

LigMV_alpha_AUT          GCCAAGTGGTTTGGTCTGACAGTAGATCCGAGTTCTTTTGACATTTGTGACCCTCCGCTT
LigMV_alpha_CH_sRNA      GCCAAGTGGTTTGGTCTGACAGTAGATCCGAGTTCTTTTGACATTTGTGACCCTCCGCTT
LigMV_alpha_FR           GCCAAGTGGTTTGGTCTGACAGTAGATCCGAGTTCTTTTGACATTTGTGACCCTCCGCTT
                         ************************************************************

LigMV_alpha_AUT          TTCGTAGAAATACGAGACAGATGGAAGGTTCAAGCCAGAGGTGACATCAAATTAAGCTCG
LigMV_alpha_CH_sRNA      TTCGTAGAAATACGAGACAGATGGAAGGTTCAAGCCAGAGGTGACATCAAATTAAGCTCG
LigMV_alpha_FR           TTCGTAGAAATACGAGACAGATGGAAGGTTCAAGCCAGAGGTGACATCAAATTAAGCTCG
                         ************************************************************

LigMV_alpha_AUT          TTGTATGACTTTGAGGATGATGCACGGGAGTTTGATAAAAGAGTGGAAGAGAAGCGACGT
LigMV_alpha_CH_sRNA      TTGTATGACTTTGAGGATGATGCGCGGGAGTTTGATAAAAGAGTGGAAGAGAAGCGACGT
LigMV_alpha_FR           TTGTATGACTTTGAGGATGATGCACGGGAGTTTGATAAAAGAGTGGAAGAGAAGCGACGT
                         *********************** ************************************

LigMV_alpha_AUT          TTGGCTGAAGATATTGTCAATAAAGTTACAGGGGTTGCAAGCCCTGGAAGTCCTACGAAT
LigMV_alpha_CH_sRNA      TTGGCTGAAGATATTGTCAATAAAGTTACAGGGGTTGCAAGCCCTGGAAGTCCTACGAAT
LigMV_alpha_FR           TTGGCTGAAGATATTGTCAATAAAGTTACAGGGGTTGCAAGCCCTGGAAGTCCTACGAAT
                         ************************************************************

LigMV_alpha_AUT          ATGGAGAACCTACAGAAGCTAAATTCAATTTCACGCCTGTTTCTAGCTGCTACTGTGGTT
LigMV_alpha_CH_sRNA      ATGGAGAACCTACAGAAGCTAAATTCAATTTCACGCCTGTTTCTAGCTGCTACTGTGGTT
LigMV_alpha_FR           ATGGAGAACCTACAGAAGCTAAATTCAATTTCACGCCTGTTTCTAGCAGCTACTGTGGTT
                         *********************************************** ************

LigMV_alpha_AUT          AACAAATTCAAGACTTCGTCTTTCGATAGTTTTTCAGATACGGCTTCCGTCCATTCGGAG
LigMV_alpha_CH_sRNA      AACAAATTCAAGACTTCGTCTTTCGATAGTTTTTCAGATACGGCTTCCGTCCATTCGGAG
LigMV_alpha_FR           AACAAATTCAAGACTTCGTCTTTCGATAGTTTTTCAGATACGGCTTCCGTCCATTCAGAG
                         ******************************************************** ***

LigMV_alpha_AUT          TCTTCATTGACTTTACCTTCTTCACCGACATTATCTATCGATGGTCGACTCCCTATGTGG
LigMV_alpha_CH_sRNA      TCTTCATTGACTTTACCTTCTTCACCGACATTATCTATCGATGGTCGACTCCCTATGTGG
LigMV_alpha_FR           TCTTCATTGACTTTACCTTCTTCACCGACATTATCTATCGATGGTCGACTCCCTATGTGG
                         ************************************************************

LigMV_alpha_AUT          GAAGTAGCTCATGCAGATGTGTTGAATTTTCAACAAATTAATGGCAACATGTTAAAGAGA
LigMV_alpha_CH_sRNA      GAAGTAGCTCATGCAGATGTGTTGAATTTTCAACAAATTAATGGCAACATGTTAAAGAGA
LigMV_alpha_FR           GAAGTAGCTCATGCAGATGTGTTGAATTTTCAACAAATTAATGGCAACATGTTAAAGAGA
                         ************************************************************

LigMV_alpha_AUT          ACTGGTGTACCAGATAGACCAGTAGAAAGCAAAGATTATTTTGAGAACTCTAGAGCCGAG
LigMV_alpha_CH_sRNA      ACTGGTGTACCAGATAGACCAGTAGAAAGCAAAGATTATTTTGAGAACTCTAGAGCCGAG
LigMV_alpha_FR           ACTGGTGTACCAGATAGACCAGTAGAAAGCAAAGATTATTTTGAGAACTCTAGAGCCGAG
                         ************************************************************

LigMV_alpha_AUT          TTTTTATACTATTTGTTGTCTAGTGTTATAAGTGAAAGAGCCCAAATGATGGACATTGTT
LigMV_alpha_CH_sRNA      TTTTTATACTATTTGTTGTCTAGTGTTATAAGTGAAAGAGCCCAAATGATGGACATTGTT
LigMV_alpha_FR           TTTTTATACTATTTGTTGTCTAGTGTTATAAGTGAAAGAGCCCAAATGATGGACATTGTT
                         ************************************************************

LigMV_alpha_AUT          CATAGTTACAGGCTTGGTCATTTATTCACTGACAAGGTAGTGGCTCCTCTAACATCGTAT
LigMV_alpha_CH_sRNA      CATAGTTACAGGCTCGGTCATTTATTCACTGACAAGGTAGTGGCTCCTCTAACATCGTAT
LigMV_alpha_FR           CATAGTTACAGGCTCGGTCATTTATTCACTGACAAGGTAGTGGCTCCTCTAACATCGTAT
                         ************** *********************************************

LigMV_alpha_AUT          ATCTACTCTTGTGAGAGGGCGGATTGGGTGTTGAGGACTCCTCGAGCGAATGATGTGGGA
LigMV_alpha_CH_sRNA      ATCTACTCTTGTGAGAGGGCGGATTGGGTGTTGAGGACTCCTCGAGCGAATGATGTGGGA
LigMV_alpha_FR           ATCTACTCTTGTGAGAGGGCGGATTGGGTGTTGAGGACTCCTCGAGCGAATGATGTGGGA
                         ************************************************************

LigMV_alpha_AUT          CATAACTTTGCGGTGCATTTCAATTTCAAAGGAGAGAGTAAAGAGACAGATGAGTACCTA
LigMV_alpha_CH_sRNA      CATAACTTTGCGGTGCATTTCAATTTCAAAGGAGAGAGTAAAGAGACAGATGAGTACCTA
LigMV_alpha_FR           CATAACTTTGCGGTGCATTTCAATTTCAAAGGAGAGAGTAAAGAGACAGATGAGTACCTA
                         ************************************************************

LigMV_alpha_AUT          GAGTATTGTGAGATCGTTGATGTGAGATGGGACAAGTCTGGAGGTTTTATGTCAAACATT
LigMV_alpha_CH_sRNA      GAGTATTGTGAGATCGTTGATGTGAGATGGGACAAGTCTGGAGGTTTTATGTCAAACATT
LigMV_alpha_FR           GAGTATTGTGAGATCGTTGATGTGAGATGGGACAAGTCTGGAGGTTTTATGTCAAACATT
                         ************************************************************

LigMV_alpha_AUT          CCAATCGTACCAAAGTCTACCGGTTTTTACTTGTTTTGTGAGCCAACTAAATTGTGTAAT
LigMV_alpha_CH_sRNA      CCAATCGTACCAAAGTCTACCGGTTTTTACTTGTTTTGTGAGCCAACTAAATTGTGTAAT
LigMV_alpha_FR           CCAATCGTACCAAAGTCTACCGGTTTTTACTTGTTTTGTGAGCCAACTAAATTGTGTAAT
                         ************************************************************

LigMV_alpha_AUT          AATTATCTTATTTACAACAATTTAGTAGATCTTTTTTCTTCGTGTAAGAATAGAAAGGTC
LigMV_alpha_CH_sRNA      AATTATCTTATTTACAACAATTTAGTAGATCTTTTTTCTTCGTGTAAGAATAGAAAGGTC
LigMV_alpha_FR           AATTATCTTATTTACAACAATTTAGTAGATCTTTTTTCTTCGTGTAAGAATAGAAAGGTC
                         ************************************************************

LigMV_alpha_AUT          ACCTTTGAACTCATAGAGGGCGTTCCGGGTTGCGGAAAATCCACAATGATTTTGGAGACT
LigMV_alpha_CH_sRNA      ACCTTTGAACTCATAGAGGGTGTTCCGGGTTGCGGAAAATCCACAATGATTTTGGAGACT
LigMV_alpha_FR           ACCTTTGAACTCATAGAGGGTGTTCCGGGTTGCGGAAAATCCACAATGATTTTGGAGACT
                         ******************** ***************************************

LigMV_alpha_AUT          GCAAACATGAGAAGTGAGGCCGTTGTAGGTGAGGGAAGACAAGCAATGGATGATCTGAGA
LigMV_alpha_CH_sRNA      GCAAACATGAGAAGTGAGGCCGTTGTAGGTGAGGGAAGACAAGCAATGGATGATCTGAGA
LigMV_alpha_FR           GCAAACATGAGAAGTGAGGCCGTTGTAGGTGAGGGAAGACAAGCAATGGATGATCTGAGA
                         ************************************************************

LigMV_alpha_AUT          AATAGATTCGTGAAACAGAAGAAGTGGTCACAAAACACAGCTAATAAGAAAGTGAGAACT
LigMV_alpha_CH_sRNA      AATAGATTCGTGAAACAGAAGAAGTGGTCACAAAACACAGCTAATAAGAAAGTGAGAACT
LigMV_alpha_FR           AATAGATTCGTGAAACAGAAGAAGTGGTCACAAAACACAGCTAATAAGAAAGTGAGAACT
                         ************************************************************
```

```
LigMV_alpha_AUT          TTAGATAGCGTTCTTTTGGCTACTCAACCTGACAGAATCCCGAAAGTGAAAGGCTTCCAT
LigMV_alpha_CH_sRNA      TTAGATAGCGTTCTTTTGGCTACTCAACCTGACAGAATCCCGAAGGTGAAAGGCTTCCAT
LigMV_alpha_FR           TTAGATAGCGTTCTTTTGGCTACTCAACCTGACAGAATCCCGAAGGTGAAAGGCTTCCAT
                         ******************************************** ***************

LigMV_alpha_AUT          TTCGATGAGGCATTAAAGGTGCATTTTGGTGCTATACTTTTTTGTGCAGATAAAATGGGA
LigMV_alpha_CH_sRNA      TTCGATGAGGCATTAAAGGTGCATTTTGGTGCTATACTTTTTTGTGCAGATAAAATGGGA
LigMV_alpha_FR           TTCGATGAGGCATTAAAGGTGCATTTTGGTGCTATACTTTTTTGTGCAGATAAAATGGGA
                         ************************************************************

LigMV_alpha_AUT          AGCAATTATGTGATCGGACAAGGTGACAGAGCTCAATTGCCTATGATTAATCGAGTTGAG
LigMV_alpha_CH_sRNA      AGCAATTATGTGATCGGACAAGGTGACAGAGCTCAATTGCCTATGATTAATCGAGTTGAG
LigMV_alpha_FR           AGCAATTATGTGATCGGACAAGGTGACAGAGCTCAATTGCCTATGATTAATCGAGTTGAG
                         ************************************************************

LigMV_alpha_AUT          GGCATATCTCTGAAGTTTAGCACGCCAGATTACTCTCAAGTGAAAGTGACACCGAAATTG
LigMV_alpha_CH_sRNA      GGCATATCTCTGAAGTTTAGCACGCCAGATTACTCTCAAGTGAAAGTGACACCGAAATTG
LigMV_alpha_FR           GGCATATCTCTGAAGTTTAGCACGCCAGATTACTCTCAAGTGAAAGTGACACCGAAATTG
                         ************************************************************

LigMV_alpha_AUT          TTATCATATAGAATTCCGGGGGATGTTGCATATTACTTGAGTAAGAGGGCCTGTTATAAG
LigMV_alpha_CH_sRNA      TTATCATATAGAATTCCGGGGGATGTTGCATATTACTTGAGTAAGAGGGCCTGTTATAAG
LigMV_alpha_FR           TTATCATATAGAATTCCGGGGGATGTTGCATATTACTTGAGTAAGAGGGCCTGTTATAAG
                         ************************************************************

LigMV_alpha_AUT          GACAGGGGTATTCCACAGGTTGTAACTACAAAAAATCAGGTCACAAGGTCAATGTTCGCG
LigMV_alpha_CH_sRNA      GACAGGGGTATTCCACAGGTTGTAACTACAAAAAATCAGGTCACAAGGTCAATGTTCGCG
LigMV_alpha_FR           GACAGGGGTATTCCACAGGTTGTAACTACAAAAAATCAGGTCACAAGGTCAATGTTCGCG
                         ************************************************************

LigMV_alpha_AUT          AGGGGGCAGAACACAACTGAGAGGTTTGTTAGTCTACAAGACTTACCGATCGTTTTGGGT
LigMV_alpha_CH_sRNA      AGGGGGCAGAACACAACTGAGAGGTTTGTTAGTCTACAAGACTTACCGATCGTTTTGGGT
LigMV_alpha_FR           AGGGGGCAGAACACAACTGAGAGGTTTGTTAGTCTACAAGACTTACCGATCGTTTTGGGT
                         ************************************************************

LigMV_alpha_AUT          GCGCAGTATCTTACTTTTCTGCAAGCAGAAAAGGAGACAATTATTTCGCATTTAAAGGCA
LigMV_alpha_CH_sRNA      GCGCAGTATCTTACTTTTCTGCAAGCAGAAAAGGAGACAATTATTTCGCATTTAAAGGCA
LigMV_alpha_FR           GCGCAGTATCTTACTTTTCTGCAAGCAGAAAAGGAGACAATTATTTCGCATTTAAAGGCA
                         ************************************************************

LigMV_alpha_AUT          AAAGGTGTGAAGGATTTCAATGTGAATACCATTCATGAATCTCAGGGAGGAACATTTAAA
LigMV_alpha_CH_sRNA      AAAGGTGTGAAGGATTTCAATGTGAATACCATTCATGAATCTCAGGGAGGAACATTTAAA
LigMV_alpha_FR           AAAGGTGTGAAGGATTTCAATGTGAATACCATTCATGAATCTCAGGGAGGAACATTTAAA
                         ************************************************************

LigMV_alpha_AUT          AATGTTATTCTTGTTCGTTTGCAAAGAACTGAGAATGAGATTTATCCTGGTGGTGTGAGA
LigMV_alpha_CH_sRNA      AATGTTATTCTTGTTCGTTTGCAAAGAACTGAGAATGAGATTTATCCTGGTGGTGTGAGA
LigMV_alpha_FR           AATGTTATTCTTGTTCGTTTGCAAAGAACTGAGAATGAGATTTATCCTGGTGGTGTGAGA
                         ************************************************************

LigMV_alpha_AUT          AGTTCAAGTTACGTTGTTGTTGGAATATCTCGACATACGGAAACTTTCACATACTGTAGT
LigMV_alpha_CH_sRNA      AGTTCAAGTTACGTTGTTGTTGGAATATCTCGACATACGGAAACTTTCACATACTGTAGT
LigMV_alpha_FR           AGTTCAAGTTACGTTGTTGTTGGAATATCTCGACATACGGAAACTTTCACATACTGTAGT
                         ************************************************************

LigMV_alpha_AUT          GTCGTTGATGATCGTTTGTTACTGGACATAGCAAATGATGATGGTTTAAAAAATACTCCG
LigMV_alpha_CH_sRNA      GTCGTTGATGATCGTTTGTTACTGGACATAGCAAATGAAGATGGTTTAAAAAATACTCCG
LigMV_alpha_FR           GTCGTTGATGATCGTTTGTTACTGGACATAGCAAATGAAGATGGTTTAAAAAATACTCCG
                         ************************************** *********************

LigMV_alpha_AUT          ATTCGGACGTTTCAAGAGTACATAATAAAATAAATAAGTATTTCT------TATTTTAAA
LigMV_alpha_CH_sRNA      ATTCGGACGTTTCAAGAGTACATAATAAAATAAATAAGTTTTTCTT-----TTTTTTAAA
LigMV_alpha_FR           ATTCGGACGTTTCAAGAGTACATAATAAAATAAATAAGTTTTTCTTTTTCCTTTTTTAAA
                         *************************************** *****      * *******

LigMV_alpha_AUT          AAAAAAAAAAAAAAAAAAAAAAAAAAAAAAAAAAAACCAATTTCCCTTTTAAAATAAAAA
LigMV_alpha_CH_sRNA      AAAAAAAAAAAAAAAAAAAAAAAAAAAAAAAAAAAA-------------------AAAAA
LigMV_alpha_FR           AAAAAAAAAAAAAAAAAAAAAAAA------------------------------------
                         ************************

LigMV_alpha_AUT          AAAAAAAAAAAAAAATTTTTTTTTTTTAAAAAAAAAAAAAAAAAAAAAAAAAAAAAAAAA
LigMV_alpha_CH_sRNA      AAAAAAAAAAAAAAATGCCTGCTATTAAGACGGTGAAACCTATAAACGGGTTCAACGGGT
LigMV_alpha_FR           ------------------------------------------------------------


LigMV_alpha_AUT          AAAAAAAAAAAAA---------AAATGCCTGCTATTAAG-----------ACGGTGAACT
LigMV_alpha_CH_sRNA      GAGATAGTGGGAATTGGTGTGTAAGCGCGTACTCCTACGTCGCTGCATCAACAATGATCT
LigMV_alpha_FR           ------------------------------------------------------------


LigMV_alpha_AUT          GT------------------------------------------------------
LigMV_alpha_CH_sRNA      GTGGATCGATTGTTGCACACTGAGCCGAAGCTCAGCTTTGGTCCTTTAGGAGACCA
LigMV_alpha_FR           --------------------------------------------------------

----------------------

CLUSTAL multiple sequence alignment by MUSCLE (3.8)


LigMV_beta_CH_sRNA       cagtaaaggtgtacttgtaccaacgcatgattGTAAAAATACCAACAATCGTAAAGAGCT
LigMV_beta_CH_dsRNA      ------------------------------------------------------------


LigMV_beta_CH_sRNA       TGCTTTGCTCCTCTTTACAAAATCTATCCTTTTATATTAGTAATAGGTTTTTGTATTTTT
LigMV_beta_CH_dsRNA      ----------------------------TTTTT--------------TTTTTGTATTTTT
                                                      ****              *************

LigMV_beta_CH_sRNA       ACGTTTACAATGGCTAATCTTGGGTTAACTGTAAAAGGTGGTGGTCACTACGATGAAACT
LigMV_beta_CH_dsRNA      ACGTTTACAATGGCTAATCTTGGGTTAACTGTAAAAGGTGGTGGTCACTACGATGAAACT
                         ************************************************************

LigMV_beta_CH_sRNA       CAGTGGTACAACAATGTTGTACGTACTAGTGCTATGCACGACTGGTGGGTCGCCAAAGAG
LigMV_beta_CH_dsRNA      CAGTGGTACAACAATGTTGTACGTACTAGTGCTATGCACGACTGGTGGGTCGCCAAAGAG
                         ************************************************************

LigMV_beta_CH_sRNA       TCCTGGAAAGACATGCTTGATGCACTTTCTAAGGTTCAATTTGATGTGATCAATTCTCGT
LigMV_beta_CH_dsRNA      TCCTGGAAAGACATGCTTGATGCACTTTCTAAGGTTCAATTTGATGTGATCAATTCTCGT
                         ************************************************************

LigMV_beta_CH_sRNA       AGAGCAATCGCCGAAATCATTATTAACCTAAAAAGAGATTTACCTGCTGCCTTAGATCGA
LigMV_beta_CH_dsRNA      AGAGCAATCGCCGAAATCATTATTAACCTAAAAAGAGATTTACCTGCTGCCTTAGATCGA
                         ************************************************************

LigMV_beta_CH_sRNA       CGTTTCGCTGGGTCCACTGGGACTTTAGGCGCTGCCAACTACCGTGAAGCTCCTCTCTTT
LigMV_beta_CH_dsRNA      CGTTTCGCTGGGTCCACTGGGACTTTAGGCGCTGCCAACTACCGTGAAGCTCCTCTCTTT
                         ************************************************************

LigMV_beta_CH_sRNA       GTGAGGTTAGATGGTGCTGCTCGAGCACACTTAACAGCTCTTGCCAGAATTGGTGACCAG
LigMV_beta_CH_dsRNA      GTGAGGTTAGATGGTGCTGCTCGAGCACACTTAACAGCTCTTGCCAGAATTGGTGACCAG
                         ************************************************************

LigMV_beta_CH_sRNA       CCCTCTACATCACGAGACTTGGAACTTTACCGTGGGAAAGCTACCGTCCAGCAACCAGCT
LigMV_beta_CH_dsRNA      CCCTCTACATCACGAGACTTGGAACTTTACCGTGGGAAAGCTACCGTCCAGCAACCAGTT
                         ********************************************************** *

LigMV_beta_CH_sRNA       TCTGCAAATCCTCCTGCAATTAAAGATATTCAGCCGTTAAGGACGGTGCATTGCACCTG
LigMV_beta_CH_dsRNA      TCTGCAAATCCTCCTGCAATTAAAGATATTCAACCGTTAAGGACGGTGCATTGCACCTG
                         ******************************** ***************************
```

```
LigMV_beta_CH_sRNA       ACCTGGGATTTGAAAGACCTCGTTGTCAGTGATCCGCCGATTTATGACAGATTGACGTTC
LigMV_beta_CH_dsRNA      ACCTGGGATTTGAAAGACCTCGTTGTCAGTGATCCGCCGATTTATGACAGATTGACGTTC
                         ************************************************************

LigMV_beta_CH_sRNA       GAAGAACAGTTCCATCTGGTCTGGTACGAAAGAGTACCGGTTGTTCAGCAGTAAATTTTG
LigMV_beta_CH_dsRNA      GAAGAACAGTTCCATCTGGTCTGGTACGAAAGAGTACCGGTTGTTCAGCAGTAAATTTTG
                         ************************************************************

LigMV_beta_CH_sRNA       ATTATACATTGTAAACAATAATAATAGACTTTTCTTTGTG----TGTTTGTTTGTTTGTT
LigMV_beta_CH_dsRNA      ATTATACATTGTAAAC---AATAATAGACTTTTCTTTGTGTGTTTGTTTGTTTGTTTGTT - shared with FR and AUT
                         ****************   *********************    ****************

LigMV_beta_CH_sRNA       TGTTTCTTTAGTTCTTTATTTTTCTAGTTTGTTTCTAGGATGTTTTTTGTGTTTGTTAAC
LigMV_beta_CH_dsRNA      TGTTTCTTTAGTTCTTTATTTTTCTAGTTTGTTTCTAGGATGTTTTTTGTGTTTGTTAAC
                         ************************************************************

LigMV_beta_CH_sRNA       CATGTCAATCGTTGATCATGTTGAAAACGTTAAGAAAGTTGTAAGTGAAGAAACTCCCGT
LigMV_beta_CH_dsRNA      CATGTCAATCGTTGATCAAGTTGAAAACGTTAAGAAAGTTGTAAGTGAAGAAACTCCCGT
                         ****************** *****************************************

LigMV_beta_CH_sRNA       AATCGATGATAAGAAACCGACAAAGAAGAAACGAAGGAATGTCAACAAGAAAAAGAAACA
LigMV_beta_CH_dsRNA      AATCGATGATAAGAAACCGACAAAGAAGAAACGAAGGAATGTCAACAAGAAAAAGAAACA
                         ************************************************************

LigMV_beta_CH_sRNA       GCGTGAGCCATTGGCTGCTGTGACTGATTCTAAAGAAAATTCTTTTAGGAGGTCGAATAG
LigMV_beta_CH_dsRNA      GCGTGAGCCATTGGCTGCTGTGACTGATTCTAAAGAAAATTCTTTTAGGAGGTCGAATAG
                         ************************************************************

LigMV_beta_CH_sRNA       CCTTGAATCTTTGGGGAAGTATTTAGATACGTGGAAGGATTCTGAAAATTCAAAAAAGAA
LigMV_beta_CH_dsRNA      CCTTGAATCTTTGGGGAAGTATTTAGATACGTGGAAGGATTCTGAAAATTCAAAAAAGAA
                         ************************************************************

LigMV_beta_CH_sRNA       GAAATCTGTGGAAGAGAAGCACGCTGCAAATGACGCCAGACGTTTTGGTTTAGCTGAGAA
LigMV_beta_CH_dsRNA      GAAATCTGTGGAAGAGAAGCACGCTGCAAATGACGCCAGACGTTTTGGTTTAGCTGAGAA
                         ************************************************************

LigMV_beta_CH_sRNA       GTTTGCGTTGCAGTTGCATTCTGATTGCGTAGCGGCTGGTTTCAAGGATACTGGTAAGAA
LigMV_beta_CH_dsRNA      GTTTGCGTTGCAGTTGCATTCTGATTGCGTAGCGGCTGGTTTCAAGGATACTGGTAAGAA
                         ************************************************************

LigMV_beta_CH_sRNA       TTTTCAAATGTTGCCTGCAAATTATCTTGAGAGAAGTAGATTACTCGCTCTCTATGATAA
LigMV_beta_CH_dsRNA      TTTTCAAATGTTGCCTGCAAATTATCTTGAGAGAAGTAGATTACTCGCTCTCTATGATAA
                         ************************************************************

LigMV_beta_CH_sRNA       GAATCTCAAGCAGAGTTTAAAAAAGGCGTGTGAAACTGAACAGGAGAGAATTAGGACTAA
LigMV_beta_CH_dsRNA      GAATCTCAAGCAGAGTTTAAAAAAGGCGTGTGAAACTGAACAGGAGAGAATTAGGACTAA
                         ************************************************************

LigMV_beta_CH_sRNA       AGTGTTCCTTTTAAGGAGCGCAAGACCTAATGTTAATTTCAAAATTGGAATTATCTCCGG
LigMV_beta_CH_dsRNA      AGTGTTCCTTTTAAGGAGCGCAAGACCTAATGTTAATTTCAAAATTGGAATTATCTCCGG
                         ************************************************************

LigMV_beta_CH_sRNA       TGTTGCTGGTTCTGGAAAATCTACACTCATAAGAAGTTTGTGTTCGAAATCGAATGCACT
LigMV_beta_CH_dsRNA      TGTTGCTGGTTCTGGAAAATCTACACTCATAAGAAGTTTGTGTTCGAAATCGAATGCACT
                         ************************************************************

LigMV_beta_CH_sRNA       GTGCGTACTGGCGAACCCTCGTTTGAAAGAGACAGACTACAAAGGTCAAGACAAGACCTT
LigMV_beta_CH_dsRNA      GTGCGTACTGGCGAATCCTCGTTTGAAAGAGACAGACTACAAAGGTCAAGACAAGACCTT
                         *************** ********************************************

LigMV_beta_CH_sRNA       TACGCTGCAACAAGTTCTTTTGTCTATTGTTCCAATGCGTGCTGATATAATCGTCGTTGA
LigMV_beta_CH_dsRNA      TACGCTGCAACAAGTTCTTTTGTCTATTGTTCCAATGCGTGCTGATATAATCGTCGTTGA
                         ************************************************************

LigMV_beta_CH_sRNA       TGAATATACTTTGGCTGAAAGCGCAGAAATCCTACTGCTGCAACGTCTATTGCAAGCTAG
LigMV_beta_CH_dsRNA      TGAATATACTTTGGCTGAAAGCGCAGAAATCCTACTGCTGCAACGTCTATTGCAAGCTAG
                         ************************************************************

LigMV_beta_CH_sRNA       CTTCTTGCTCCTCTTTGGGGACGTTGCCCAAGGCGAATCTGATAATGCTTCGAGCTTAGA
LigMV_beta_CH_dsRNA      CTTCTTGCTCCTCTTTGGGGACGTTGCCCAAGGCGAATCTGATAATGCTTCGAGCTTAGA
                         ************************************************************

LigMV_beta_CH_sRNA       GTATCTCCAGTTGCCTGTAGTTTACAGGTCTGTTACATCGCACCGTTTGGGAAACGAGAC
LigMV_beta_CH_dsRNA      GTATCTCCAGTTGCCTGTAGTTTACAGGTCTGTTACGTCGCACCGTTTGGGAAATGAGAC
                         ************************************ ***************** *****

LigMV_beta_CH_sRNA       AGCTGCTTTTTGCAAAAAGCATGGTTTGGCTTTTGATTCCGATTCTCCAGTGAAAGATGA
LigMV_beta_CH_dsRNA      AGCTGCTTTTTGCAAAAAGCATGGTTTGGCTTTTGATTCCGATTCTCCAGTGAAAGATGA
                         ************************************************************

LigMV_beta_CH_sRNA       AATAATTACGAGTGGATATGAAGGAGACACAGAGACAACAGAGAAAAACATTGCCTTTTC
LigMV_beta_CH_dsRNA      AATAATTACGAGTGGATATGAAGGAGACACAGAGACAACAGAGAAAAACATTGCCTTTTC
                         ************************************************************

LigMV_beta_CH_sRNA       CGCAGCGACAGTTGAAGACTTGCATGAGGCTGGTGTTGAGGCGACCTTGGTTTTGGAAAC
LigMV_beta_CH_dsRNA      CGCAGCGACAGTTGAAGACTTGCATGAGGCTGGTGTTGAGGCGACCTTGGTTTTGGAAAC
                         ************************************************************

LigMV_beta_CH_sRNA       ACAAGGAAAAGAATATGATAGTGTTACTTTGTTTGTAAGAGATAGTGACAGAGAAGCTGC
LigMV_beta_CH_dsRNA      ACAAGGAAAAGAATATGATAGTGTTACTTTGTTTGTAAGAGATAGTGACAGAGAAGCTGC
                         ************************************************************

LigMV_beta_CH_sRNA       AAGCAATCCGCATGCACGAACTGTTGCTTTTACTCGTCATAAACGTTTGTTAATAATTAG
LigMV_beta_CH_dsRNA      AAGCAATCCGCATGCACGAACTGTTGCTTTTACTCGTCATAAACGTTTGTTAATAATTAG
                         ************************************************************

LigMV_beta_CH_sRNA       GGCTGAGGAAGAAATTCTTCCTTCTCTCCTTAATGGTGAATTAAACGTTAAGATAAGTGC
LigMV_beta_CH_dsRNA      GGCTGAGGAAGAAATTCTTCCTTCTCTCCTTAATGGTGAATTAAACGTTAAGATAAGTGC
                         ************************************************************

LigMV_beta_CH_sRNA       AAACTCTCATAAATACGGTGACATAAAATATTCTCATGCCGACAACAGTAGCTCAGAGAC
LigMV_beta_CH_dsRNA      AAACTCTCATAAATACGGTGACATAAAATATTCTCATGCCGACAACAGTAGCTCAGAGAC
                         ************************************************************

LigMV_beta_CH_sRNA       CTAACAAATATTGGCCTATAGTGGTTGGAGTTGGCTTGATTGGTTTGTTTGGTTATTTGA
LigMV_beta_CH_dsRNA      CTAACAAATATTNNNNTATAGTGGTTGGAGTTGGCTTGATTGGTTTGTTTGGTTATTTGA
                         ************    ********************************************

LigMV_beta_CH_sRNA       TTTTCACTAATCAAAAACACTCTACCCAATCTGGCGACAACATTCACAAATTTGCTAACG
LigMV_beta_CH_dsRNA      TTTTCACTAATCAAAAACACTCTACCCAATCTGGCGACAACATCCACAAATTTGCTAACG
                         ******************************************* ****************

LigMV_beta_CH_sRNA       GTGGTTCTTACGCGGACGGTTCTAAAAGAATAAATTACAATAGAAATAACGATCTTTCTT
LigMV_beta_CH_dsRNA      GTGGTTCTTACGCGGACGGTTCTAAAAGAATAAATTACAATAGAAATAACGATCTTTCTT
                         ************************************************************

LigMV_beta_CH_sRNA       ACGGTTACAAGGGTTTATTTAGTGGTAGTAATAATGCCTCACTCCTTAACTTGTCTCTGC
LigMV_beta_CH_dsRNA      ACGGTTACAAGGGTTTATTTAGTGGTAGTAATAATGCCTCACTCCTTAACTTGTCTCTGC
                         ************************************************************

LigMV_beta_CH_sRNA       TCGGAATGTTCCTTACAGCCATGTGTGCCGGAATTTACGGAGAATACATGCGCAGAAAGG
LigMV_beta_CH_dsRNA      TCGGAATGTTCCTTACAGCCATGTGTGCCGGAATTTACGGAGAATACATGCGCAGAAAGG
                         ************************************************************

LigMV_beta_CH_sRNA       AACAATCAGGATGTATTGTTTGCCCCCCAGATTGCAAGATCTGTGGGTAATGACTACATC
LigMV_beta_CH_dsRNA      AACAATCAGGATGTATTGTTTGCCCCCCAGATTGCAAGATCTGTGGGTAATGACTACATC
                         ************************************************************
```

```
LigMV_beta_CH_sRNA       TTCGGTGTGGTTTATGGAATTATAGGTGGAATACTATCAGTTTTAATCCTTATATGGTTG
LigMV_beta_CH_dsRNA      TTCGGTGTGGTTTATGGAATTATAGGTGGAATACTATCAGTTTTAATCCTTATATGGTTG
                         ************************************************************

LigMV_beta_CH_sRNA       TTTGGTTTAACATCAGATAATGGAAGAAAGCCAGCTGTTTCTTGGTTTAGACAAGACCTT
LigMV_beta_CH_dsRNA      TTTGGTTTAACATCAGATAATGGAAGAAAGCCAGCTGTTTCTTGGTTTAGACAAGACCTT
                         ************************************************************

LigMV_beta_CH_sRNA       AACGGTTTTGAAATGCAAGTAACGGAAGGTTCCCCAATTGATCCTGAAGTAGTAAAAGCT
LigMV_beta_CH_dsRNA      AACGGTTTTGAAATGCAAGTAACGGAAGGTTCCCCAATTGATCCTGAAGTAGTAAAAGCT
                         ************************************************************

LigMV_beta_CH_sRNA       GTCCATCACTTTCAGAAATACCCCTATGGGAGTGTTCCTGTGTGGGATCGCTTTGATAAC
LigMV_beta_CH_dsRNA      GTCCATCACTTTCAGAAATACCCCTATGGGAGTGTTCCTGTGTGGGATCACTTTGATAAC
                         ************************************************* **********

LigMV_beta_CH_sRNA       ATAATGGATCATGCGACCCAATGGTTAACTTCATTTATTAATTTGATGTTGTTTTTATTC
LigMV_beta_CH_dsRNA      ATAATGGATCATGCGACCCAATGGTTAACTTCATTTATTAATTTGATGTTGTTTTTATTC
                         ************************************************************

LigMV_beta_CH_sRNA       ATGTGTGGTGTGGTTTTCTTTTTCGTAGGGTGTTTTTACATCCTATGATTTTTTTTTTTA
LigMV_beta_CH_dsRNA      ATGTGTGGTGTGGTTTTCTTTTTCGTAGGGTGTTTTTACATCCTATGATTTTCTTTTTTT
                         **************************************************** ******

LigMV_beta_CH_sRNA       AAAAAAAAAAAAAAAAAAAAAAAAAAAAAAAAAAAAAAAAAAAAAAAAAAAAAAAAAATG
LigMV_beta_CH_dsRNA      TTTAAAAAAAAAAAA---------------------------------------------
                            ************
```

CLUSTAL multiple sequence alignment by MUSCLE (3.8)

```
LigMV_beta_CH_sRNA      -------cagtaaaggtgtacttgtaccaacgcatgattGT---------AAAAATACCA
LigMV_beta_FR           CGCTACGCAATCAGAATGCAACTG---CAACGCAAACTTCTCAGCTAAACCAAAACGTCT
LigMV_beta_AUT          -----------------------G---CAAAGC---------------------------
                                               *   *** **

LigMV_beta_CH_sRNA      ACAATCGTAAAGAGCTTGCTTTGCTCCTCTTTACAAAATCTATCCTTTTATATTAGTAAT
LigMV_beta_FR           GGCGTCATTTGCAGC-----GTGCTCCTCTTTACAAAATCTATCCTTTTATATTAGTAAT
LigMV_beta_AUT          -------------GG-----TTGCTCCTCTTTACAAAATCTATCCTTTTATATTAGTAGT
                                     *       ************************************* *

LigMV_beta_CH_sRNA      AGGTTTTTGTATTTTTACGTTTACAATGGCTAATCTTGGGTTAACTGTAAAAGGTGGTGG
LigMV_beta_FR           AGGTTTTTGTATTTTTACGTTTACAATGGCTAATCTTGGGTTAACTGTAAAAGGTGGTGG
LigMV_beta_AUT          AGGTTTTTGTATTTTTACGTTTACAATGGCTAATCTTGGGTTAACTGTAAAAGGTGGTGG
                        ************************************************************

LigMV_beta_CH_sRNA      TCACTACGATGAAACTCAGTGGTACAACAATGTTGTACGTACTAGTGCTATGCACGACTG
LigMV_beta_FR           TCACTACGATGAAACTCAGTGGTACAACAATGTTGTACGTACTAGTGCTATGCACGACTG
LigMV_beta_AUT          TCACTACGATGAAACTCAGTGGTACAACAATGTTGTACGTACTAGTGCTATGCACGACTG
                        ************************************************************

LigMV_beta_CH_sRNA      GTGGGTCGCCAAAGAGTCCTGGAAAGACATGCTTGATGCACTTTCTAAGGTTCAATTTGA
LigMV_beta_FR           GTGGGTCGCCAAAGAGTCCTGGAAAGACATGCTTGATGCACTTTCTAAGGTTCAATTTGA
LigMV_beta_AUT          GTGGGTCGCCAAAGAGTCCTGGAAAGACATGCTTGATGCACTTTCTAAGGTTCAATTTGA
                        ************************************************************

LigMV_beta_CH_sRNA      TGTGATCAATTCTCGTAGAGCAATCGCCGAAATCATTATTAACCTAAAAAGAGATTTACC
LigMV_beta_FR           TGTGATCAATTCTCGTAGAGCAATCGCCGAAATCATTATTAACCTAAAAAGAGATTTACC
LigMV_beta_AUT          TGTGATCAATTCTCGTAGAGCAATCGCCGAAATCATTATTAACCTAAAAAGAGATTTACC
                        ************************************************************

LigMV_beta_CH_sRNA      TGCTGCCTTAGATCGACGTTTCGCTGGGTCCACTGGGACTTTAGGCGCTGCCAACTACCG
LigMV_beta_FR           TGCTGCCTTAGATCGACGTTTCGCTGGGTCCACTGGGACTTTAGGCGCTGCCAACTACCG
LigMV_beta_AUT          TGCTGCCTTAGATCGACGTTTCGCTGGGTCCACTGGGACTTTAGGCGCTGCCAACTACCG
                        ************************************************************

LigMV_beta_CH_sRNA      TGAAGCTCCTCTCTTTGTGAGGTTAGATGGTGCTGCTCGAGCACACTTAACAGCTCTTGC
LigMV_beta_FR           TGAAGCTCCTCTCTTTGTGAGGTTAGATGGTGCTGCTCGAGCACACTTAACAGCTCTTGC
LigMV_beta_AUT          TGAAGCTCCTCTCTTTGTGAGGTTAGATGGTGCTGCTCGAGCACACTTAACAGCTCTTGC
                        ************************************************************

LigMV_beta_CH_sRNA      CAGAATTGGTGACCAGCCCTCTACATCACGAGACTTGGAACTTTACCGTGGGAAAGCTAC
LigMV_beta_FR           CAGAATTGGTGACCAGCCCTCTACATCACGAGACTTGGAACTTTACCGTGGGAAAGCTAC
LigMV_beta_AUT          CAGAATTGGTGACCAGCCCTCTACATCACGAGACTTGGAACTTTACCGTGGGAAAGCTAC
                        ************************************************************

LigMV_beta_CH_sRNA      CGTCCAGCAACCAGCTTCTGCAAATCCTCCTGCAATTAAAGATATTCAGCCGTTAAGGGA
LigMV_beta_FR           CGTCCAGCAACCAGCTTCTGCAAATCCTCCTGCAATTAAAGATATTCAGCCGTTAAGGGA
LigMV_beta_AUT          CGTCCAGCAACCAGCTTCTACAAATCCTCCTGCAATTAAAGATATTCAGCCGTTAAGGGA
                        ******************* ****************************************

LigMV_beta_CH_sRNA      CGGTGCATTGCACCTGACCTGGGATTTGAAAGACCTCGTTGTCAGTGATCCGCCGATTTA
LigMV_beta_FR           CGGTGCATTGCACCTGACCTGGGATTTGAAAGACCTCGTTGTCAGTGATCCGCCGATTTA
LigMV_beta_AUT          CGGTGCATTGCACCTGACCTGGGATTTGAAAGACCTCGTTGTCAGTGATCCGCCGATTTA
                        ************************************************************

LigMV_beta_CH_sRNA      TGACAGATTGACGTTCGAAGAACAGTTCCATCTGGTCTGGTACGAAAGAGTACCGGTTGT
LigMV_beta_FR           TGACAGATTGACGTTCGAAGAACAGTTCCATCTGGTCTGGTACGAAAGAGTACCGGTTGT
LigMV_beta_AUT          TGACAGATTGACGTTCGAAGAACAGTTCCATCTGGTCTGGTACGAAAGAGTACCGGTTGT
                        ************************************************************

LigMV_beta_CH_sRNA      TCAGCAGTAAATTTTGATTATACATTGTAAACAATAATAATAGACTTTTCTTTGTG----
LigMV_beta_FR           TCAGCAGTAAATTTTGATTATACATTGTAAACAATAATAATAGACTTTTCTTTGTGTGTT - shared with CH_dsRNA
LigMV_beta_AUT          TCAGCAGTAAATTTTGATTATACATTGTAAACAATAATAATAGACTTTTCTTTGTGTGTT
                        ********************************************************

LigMV_beta_CH_sRNA      TGTTTGTTTGTTTGTTTGTTTCTTTAGTTCTTTATTTTTCTAGTTTGTTTCTAGGATGTT
LigMV_beta_FR           TGTTTGTTTGTTTGTTTGTTTCTTTAGTTCTTTATTTTTCTAGTTTGTTTCTAGGATG-T
LigMV_beta_AUT          TGTTTGTTTGTTTGTTTGTTTCTTTAGTTCTTTATTTTTCTAGTTTGTTTCTAGGATG-T
                        ********************************************************** *

LigMV_beta_CH_sRNA      TTTTGTGTTTGTTAACCATGTCAATCGTTGATCATGTTGAAAACGTTAAGAAAGTTGTAA
LigMV_beta_FR           TTTTGTGTTTGTTAACCATGTCAATCGTTGATCAAGTTGAAAACGTTAAGAAAGTTGTAA
LigMV_beta_AUT          TTTTGTGTTTGTTAACCATGTCAATCGTTGATCAAGTTGAAAACGTTAAGAAAGTTGTAA
                        ********************************** *************************

LigMV_beta_CH_sRNA      GTGAAGAAACTCCCGTAATCGATGATAAGAAACCGACAAAGAAGAAACGAAGGAATGTCA
LigMV_beta_FR           GTGAAGAAACTCCCGTAATCGATGATAAGAAACCGACAAAGAAGAAACGAAGGAATGTCA
LigMV_beta_AUT          GTGAAGAAACTCCCGTAATCGATGATAAGAAACCGACAAAGAAGAAACGAAGGAATGTCA
                        ************************************************************

LigMV_beta_CH_sRNA      ACAAGAAAAAGAAACAGCGTGAGCCATTGGCTGCTGTGACTGATTCTAAAGAAAATTCTT
LigMV_beta_FR           ACAAGAAAAAGAAACAGCGTGAGCCATTGGCTGCTGTGACTGATTCTAAAGAAAATTCTT
LigMV_beta_AUT          ACAAGAAAAAGAAACAGCGTGAGCCATTGGCTGCTGTGACTGATTCTAAAGAAAATTCTT
                        ************************************************************

LigMV_beta_CH_sRNA      TTAGGAGGTCGAATAGCCTTGAATCTTTGGGGAAGTATTTAGATACGTGGAAGGATTCTG
LigMV_beta_FR           TTAGGAGGTCGAATAGCCTTGAATCTTTGGGGAAGTATTTAGATACGTGGAAGGATTCTG
LigMV_beta_AUT          TTAGGAGGTCGAATAGCCTTGAATCTTTGGGGAAGTATTTAGATACGTGGAAGGATTCTG
                        ************************************************************

LigMV_beta_CH_sRNA      AAAATTCAAAAAAGAAGAAATCTGTGGAAGAGAAGCACGCTGCAAATGACGCCAGACGTT
LigMV_beta_FR           AAAATTCAAAAAAGAAGAAATCTGTGGAAGAGAAGCACGCTGCAAATGACGCCAGACGTT
LigMV_beta_AUT          AAAATTCAAAAAAGAAGAAATCTGTGGAAGAGAAGCACGCTGCAAATGACGCCAGACGTT
                        ************************************************************

LigMV_beta_CH_sRNA      TTGGTTTAGCTGAGAAGTTTGCGTTGCAGTTGCATTCTGATTGCGTAGCGGCTGGTTTCA
```

```
LigMV_beta_FR           TTGGTTTAGCTGAGAAGTTTGCGTTGCAGTTGCATTCTGATTGCGTAGCGGCTGGTTTCA
LigMV_beta_AUT          TTGGTTTAGCTGAGAAGTTTGCGTTGCAGTTGCATTCTGATTGCGTAGCGGCTGGTTTCA
                        ************************************************************

LigMV_beta_CH_sRNA      AGGATACTGGTAAGAATTTTCAAATGTTGCCTGCAAATTATCTTGAGAGAAGTAGATTAC
LigMV_beta_FR           AGGATACTGGTAAGAATTTTCAAATGTTGCCTGCAAATTATCTTGAGAGAAGTAGATTAC
LigMV_beta_AUT          AGGATACTGGTAAGAATTTTCAAATGTTGCCTGCAAATTATCTTGAGAGAAGTAGATTAC
                        ************************************************************

LigMV_beta_CH_sRNA      TCGCTCTCTATGATAAGAATCTCAAGCAGAGTTTAAAAAAGGCGTGTGAAACTGAACAGG
LigMV_beta_FR           TCGCTCTCTATGATAAGAATCTCAAGCAGAGTTTAAAAAAGGCGTGTGAAACTGAACAGG
LigMV_beta_AUT          TCGCTCTCTATGATAAGAATCTCAAGCAGAGTTTAAAAAAGGCGTGTGAAACTGAACAGG
                        ************************************************************

LigMV_beta_CH_sRNA      AGAGAATTAGGACTAAAGTGTTCCTTTTAAGGAGCGCAAGACCTAATGTTAATTTCAAAA
LigMV_beta_FR           AGAGAATTAGGACTAAAGTGTTCCTTTTAAGGAGCGCAAGACCTAATGTTAATTTCAAAA
LigMV_beta_AUT          AGAGAATTAGGACTAAAGTGTTCCTTTTAAGGAGCGCAAGACCTAATGTTAATTTCAAAA
                        ************************************************************

LigMV_beta_CH_sRNA      TTGGAATTATCTCCGGTGTTGCTGGTTCTGGAAAATCTACACTCATAAGAAGTTTGTGTT
LigMV_beta_FR           TTGGAATTATCTCCGGTGTTGCTGGTTCTGGAAAATCTACACTCATAAGAAGTTTGTGTT
LigMV_beta_AUT          TTGGAATTATCTCCGGTGTTGCTGGTTCTGGAAAATCTACACTCATAAGAAGTTTGTGTT
                        ************************************************************

LigMV_beta_CH_sRNA      CGAAATCGAATGCACTGTGCGTACTGGCGAACCCTCGTTTGAAAGAGACAGACTACAAAG
LigMV_beta_FR           CGAAATCGAATGCACTGTGCGTACTGGCGAATCCTCGTTTGAAAGAGACAGACTACAAAG
LigMV_beta_AUT          CGAAATCGAATGCACTGTGCGTACTGGCGAATCCTCGTTTGAAAGAGACAGACTACAAAG
                        ******************************* ****************************

LigMV_beta_CH_sRNA      GTCAAGACAAGACCTTTACGCTGCAACAAGTTCTTTTGTCTATTGTTCCAATGCGTGCTG
LigMV_beta_FR           GTCAAGACAAGACCTTTACGCTGCAACAAGTTCTTTTGTCTATTGTTCCAATGCGTGCTG
LigMV_beta_AUT          GTCAAGACAAGACCTTTACGCTGCAACAAGTTCTTTTGTCTATTGTTCCAATGCGTGCTG
                        ************************************************************

LigMV_beta_CH_sRNA      ATATAATCGTCGTTGATGAATATACTTTGGCTGAAAGCGCAGAAATCCTACTGCTGCAAC
LigMV_beta_FR           ATATAATCGTCGTTGATGAATATACTTTGGCTGAAAGCGCAGAAATCCTACTGCTGCAAC
LigMV_beta_AUT          ATATAATCGTCGTTGATGAATATACTCTGGCTGAAAGCGCAGAAATCCTACTGCTGCAAC
                        ************************** *********************************

LigMV_beta_CH_sRNA      GTCTATTGCAAGCTAGCTTCTTGCTCCTCTTTGGGGACGTTGCCCAAGGCGAATCTGATA
LigMV_beta_FR           GTCTATTGCAAGCTAGCTTCTTGCTCCTCTTTGGGGACGTTGCCCAAGGCGAATCTGATA
LigMV_beta_AUT          GTCTATTGCAAGCTAGCTTCTTGCTCCTCTTTGGGGACGTTGCCCAAGGCGAATCTGACA
                        ********************************************************** *

LigMV_beta_CH_sRNA      ATGCTTCGAGCTTAGAGTATCTCCAGTTGCCTGTAGTTTACAGGTCTGTTACATCGCACC
LigMV_beta_FR           ATGCTTCGAGCTTAGAGTATCTCCAGTTGCCTGTAGTTTACAGGTCTGTTACATCGCACC
LigMV_beta_AUT          ATGCTTCGAGCTTAGAGTATCTCCAGTTGCCTGTAGTTTACAGGTCTGTTACATCGCACC
                        ************************************************************

LigMV_beta_CH_sRNA      GTTTGGGAAACGAGACAGCTGCTTTTTGCAAAAAGCATGGTTTGGCTTTTGATTCCGATT
LigMV_beta_FR           GTTTGGGAAACGAGACAGCTGCTTTTTGCAAAAAGCATGGTTTGGCTTTTGATTCCGATT
LigMV_beta_AUT          GTTTGGGAAACGAGACAGCTGCTTTTTGCAAAAAGCATGGTTTGGCTTTTGATTCCAATT
                        ******************************************************** ***

LigMV_beta_CH_sRNA      CTCCAGTGAAAGATGAAATAATTACGAGTGGATATGAAGGAGACACAGAGACAACAGAGA
LigMV_beta_FR           CTCCAGTGAAAGATGAAATAATTACGAGTGGATATGAAGGAGACACAGAGACAACAGAGA
LigMV_beta_AUT          CTCCAGTGAAAGATGAAATAATTACGAGTGGATATGAAGGAGACACAGAGACAACAGAGA
                        ************************************************************

LigMV_beta_CH_sRNA      AAAACATTGCCTTTTCCGCAGCGACAGTTGAAGACTTGCATGAGGCTGGTGTTGAGGCGA
LigMV_beta_FR           AAAACATTGCCTTTTCCGCAGCGACAGTTGAAGACTTGCATGAGGCTGGTGTTGAGGCGA
LigMV_beta_AUT          AAAACATTGCCTTTTCCGCAGCGACAGTTGAAGACTTGCATGAGGCTGGTGTTGAGGCGA
                        ************************************************************

LigMV_beta_CH_sRNA      CCTTGGTTTTGGAAACACAAGGAAAAGAATATGATAGTGTTACTTTGTTTGTAAGAGATA
LigMV_beta_FR           CCTTGGTTTTGGAAACACAAGGAAAAGAATATGATAGTGTTACTTTGTTTGTAAGAGATA
LigMV_beta_AUT          CCTTGGTTTTGGAAACACAAGGAAAAGAATATGATAGTGTTACTTTGTTTGTAAGAGATA
                        ************************************************************

LigMV_beta_CH_sRNA      GTGACAGAGAAGCTGCAAGCAATCCGCATGCACGAACTGTTGCTTTTACTCGTCATAAAC
LigMV_beta_FR           GTGACAGAGAAGCTGCAAGCAATCCGCATGCACGAACTGTTGCTTTTACTCGTCATAAAC
LigMV_beta_AUT          GTGACAGAGAAGCTGCAAGCAATCCGCATGCACGAACTGTTGCTTTTACTCGTCATAAAC
                        ************************************************************

LigMV_beta_CH_sRNA      GTTTGTTAATAATTAGGGCTGAGGAAGAAATTCTTCCTTCTCTCCTTAATGGTGAATTAA
LigMV_beta_FR           GTTTGTTAATAATTAGGGCTGAGGAAGAAATTCTTCCTTCTCTCCTTAATGGTGAATTAA
LigMV_beta_AUT          GTTTGTTAATAATTAGGGCTGAGGAAGAAATTCTTCCTTCTCTCCTTAATGGTGAATTAA
                        ************************************************************

LigMV_beta_CH_sRNA      ACGTTAAGATAAGTGCAAACTCTCATAAATACGGTGACATAAAATATTCTCATGCCGACA
LigMV_beta_FR           ACGTTAAGATAAGTGCAAACTCTCATAAATACGGTGACATAAAATATTCTCATGCCGACA
LigMV_beta_AUT          ACGTTAAGATAAGTGCAAACTCTCATAAATACGGTGACATAAAATATTCTCATGCCGACA
                        ************************************************************

LigMV_beta_CH_sRNA      ACAGTAGCTCAGAGACCTAACAAATATTGGCCTATAGTGGTTGGAGTTGGCTTGATTGGT
LigMV_beta_FR           ACAGTAGCTCAGAGACCTAACAAATATTGGCCTATAGTGGTTGGAGTTGGCTTGATTGGT
LigMV_beta_AUT          ACAGTAGCTCAGAGACCTAACAAATATTGGCCTATAGTGGTTGGAGTTGGCTTGATTGGT
                        ************************************************************

LigMV_beta_CH_sRNA      TTGTTTGGTTATTTGATTTTCACTAATCAAAAACACTCTACCCAATCTGGCGACAACATT
LigMV_beta_FR           TTGTTTGGTTATTTGATTTTCACTAATCAAAAACACTCTACCCAATCTGGCGACAACATC
LigMV_beta_AUT          TTGTTTGGTTATTTGATTTTCACTAATCAAAAACACTCTACCCAATCTGGCGACAACATC
                        ***********************************************************

LigMV_beta_CH_sRNA      CACAAATTTGCTAACGGTGGTTCTTACGCGGACGGTTCTAAAAGAATAAATTACAATAGA
LigMV_beta_FR           CACAAATTTGCTAACGGTGGTTCTTACGCGGACGGTTCTAAAAGAATAAATTACAATAGA
LigMV_beta_AUT          CACAAATTTGCTAACGGTGGTTCTTACGCGGACGGTTCTAAAAGAATAAATTACAATAGA
                        ************************************************************

LigMV_beta_CH_sRNA      AATAACGATCTTTCTTACGGTTACAAGGGTTTATTTAGTGGTAGTAATAATGCCTCACTC
LigMV_beta_FR           AATAACGATCTTTCTTACGGTTACAAGGGTTTATTTAGTGGTAGTAATAATGCCTCATTC
LigMV_beta_AUT          AATAACGATCTTTCTTACGGTTACAAGGGTTTATTTAGTGGTAGTAATAATGCCTCACTC
                        ********************************************************* **

LigMV_beta_CH_sRNA      CTTAACTTGTCTCTGCTCGGAATGTTCCTTACAGCCATGTGTGCCGGAATTTACGGAGAA
LigMV_beta_FR           CTTAACTTGTCTCTGCTCGGAATGTTCCTTACAGCCATGTGTGCCGGAATTTACGGAGAA
LigMV_beta_AUT          CTTAACTTGTCTCTGCTCGGAATGTTCCTTACAGCCATGTGTGCCGGAATTTACGGAGAA
                        ************************************************************

LigMV_beta_CH_sRNA      TACATGCGCAGAAAGGAACAATCAGGATGTATTGTTTGCCCCCCAGATTGCAAGATCTGT
LigMV_beta_FR           TACATGCGCAGAAAGGAACAATCAGGATGTATTGTTTGCCCCCCAGATTGCAAGATCTGT
LigMV_beta_AUT          TACATGCGCAGAAAGGAACAATCAGGATGTATTGTTTGCCCCCCAGATTGCAAGATCTGT
                        ************************************************************

LigMV_beta_CH_sRNA      GGGTAATGACTACATCTTCGGTGTGGTTTATGGAATTATAGGTGGAATACTATCAGTTTT
LigMV_beta_FR           GGGTAATGACTACATCTTCGGTGTGGTTTATGGAATTATAGGTGGAATACTATCAGTTTT
LigMV_beta_AUT          GGGTAATGACTACATCTTCGGTGTGGTTTATGGAATTATAGGTGGAATACTATCAGTTTT
                        ************************************************************

LigMV_beta_CH_sRNA      AATCCTTATATGGTTGTTTGGTTTAACATCAGATAATGGAAGAAAGCCAGCTGTTTCTTG
LigMV_beta_FR           AATCCTTATATGGTTGTTTGGTTTAACATCAGATAGTGGAAGAAAGCCAGCTGTTTCTTG
LigMV_beta_AUT          AATCCTTATATGGTTGTTTGGTTTAACATCAGATAATGGAAGGAAGCCAGCTGTTTCTTG
                        *********************************** ****** *****************

LigMV_beta_CH_sRNA      GTTTAGACAAGACCTTAACGGTTTTGAAATGCAAGTAACGGAAGGTTCCCCAATTGATCC
LigMV_beta_FR           GTTTAGACAAGACCTTAACGGTTTTGAAATGCAAGTAACGGAAGGTTCCGCAATTGATCC
LigMV_beta_AUT          GTTTAGACAAGACCTTAACGGTTTTGAAATGCAAGTAACGGAAGGTTCCCCAATTGATCC
                        ************************************************* **********
```

```
LigMV_beta_CH_sRNA      TGAAGTAGTAAAAGCTGTCCATCACTTTCAGAAATACCCCTATGGGAGTGTTCCTGTGTG
LigMV_beta_FR           TGAAGTAGTAAAAGCTGTCCATCACTTTCAGAAATACCCCTATGGGAGTGTTCCTGTGTG
LigMV_beta_AUT          TGAAGTAGTAAAAGCTGTCCATCACTTTCAGAAATACCCCTATGGGAGTGTTCCTGTGTG
                        ************************************************************

LigMV_beta_CH_sRNA      GGATCGCTTTGATAACATAATGGATCATGCGACCCAATGGTTAACTTCATTTATTAATTT
LigMV_beta_FR           GGATCACTTTGATAACATAATGGATCATACGACCCAATGGTTAACTTCATTTATTAATTT
LigMV_beta_AUT          GGATCACTTTGATAACATAATGGATCATGCGACCCAATGGTTAACTTCATTTATTAATTT
                        ***** ********************** *******************************

LigMV_beta_CH_sRNA      GATGTTGTTTTTATTCATGTGTGGTGTGGTTTTCTTTTTCGTAGGGTGTTTTTACATCCT
LigMV_beta_FR           GATGTTGTATTTATTCATGTGTGGTGTGGTTTTCTTTTTCGTAGGGTGTTTTTACATCCT
LigMV_beta_AUT          GATGTTGTTTTTATTCATGTGTGGTGTGGTTTTCTTTTTCGTAGGGTGTTTTTACATCCT
                        ******** ***************************************************

LigMV_beta_CH_sRNA      ATGATTTTTTTTTTTAAAAAAAAAAAAAAAAAAAAAAAAAAAAAAAAAAAAAAAAAAAAA
LigMV_beta_FR           ATGATTTTTTTTATTAAAAAAAAAAAAAAAAAAAAAAAAAAAAAAAAAAAAAAAAAAAAA
LigMV_beta_AUT          ATGATTTTTTTTAAAAAAAAAAAAAAAAAAAAAAAAAAAAAAAAAAAAAAAAAAAAAAAA
                        ************   *********************************************

LigMV_beta_CH_sRNA      AAAAAAAAAAAAAATGCCTGCTATTAAGACGGTGAAACCTATAAACGGGTTCAACGGGTG
LigMV_beta_FR           AAAAAAAAAAAAAA-----------AAAAAAAAAAAAA----------------------
LigMV_beta_AUT          AAAAAAA-----------------------------------------------------
                        *******

LigMV_beta_CH_sRNA      AGATAGTGGGAATTGGTGTGTAAGCGCGTACTCCTACGACGCTGCATCAACAATGATCTG
LigMV_beta_FR           ------------------------------------------------------------
LigMV_beta_AUT          ------------------------------------------------------------


LigMV_beta_CH_sRNA      TGGATCGATTGTTGCACACTGAGCCGGAGCTCAGCTTTGGTCCTTTAGGAGACCA
LigMV_beta_FR           -------------------------------------------------------
LigMV_beta_AUT          -------------------------------------------------------

-------------------
```

CLUSTAL multiple sequence alignment by MUSCLE (3.8)

```
LigMV_beta_CH_RACE        GTAAA-------------------------------------AGATCTTTCT-GCGCTTC
LigMV_gamma_CH_dsRNA      CTGGAGTTCAGACGTGTGCTCTTCCGATCTATCAGAGCCTTCGGATCCTCCTCGCGCTTC
                           *  *                                      **** * ** *******

LigMV_beta_CH_RACE        TTTGAAGGTCTGTGGGAGTTTAGATTTTACCAACAAACATTATCAATCTTTGATATTTTT
LigMV_gamma_CH_dsRNA      TCTGAAGGTCTGTGGGAGTTTAGATTTTACCAACAAACATTATCAATCTTTGATATTTTT
                          * **********************************************************

---------

LigMV_gamma_CH_sRNA       -------------------tccttAG---------------cggaTTC----cGaGCTTC
LigMV_gamma_CH_dsRNA      CTGGAGTTCAGACGTGTGCTCTTCCGATCTATCAGAGCCTTCGGATCCTCCTCGCGCTTC
                                             ** *  *               ***** *    ** *****

LigMV_gamma_CH_sRNA       TTTGAAGGTCTGTGGGAGTTTAGATTTTACCAACAAACATTATCAATCTTTGATATTTTT
LigMV_gamma_CH_dsRNA      TCTGAAGGTCTGTGGGAGTTTAGATTTTACCAACAAACATTATCAATCTTTGATATTTTT
                          * **********************************************************

LigMV_gamma_CH_sRNA       CAATGATGGCTTTGCCTAGCTTCGCGCTCGCAAAGCCTAATCCCAAACTGTGTTTTAAGA
LigMV_gamma_CH_dsRNA      CAATGATGGCTTTGCCTAGCTTCGCGCTCGCAAAGCCTAATCCCAAACTGTGTTTTAAGA
                          ************************************************************

LigMV_gamma_CH_sRNA       AACTTCAAGTATTAGCATTCGTTGCGTTACTTGTGAGGTCGAAAAAAACGTTTATTCTCT
LigMV_gamma_CH_dsRNA      AACTTCAAGTATTAGCATTCGTTGCGTTACTTGTGAGGTCGAAAAAAACGTTTATTCTCT
                          ************************************************************

LigMV_gamma_CH_sRNA       CTGTCTTAAAAAGATGGTTTGGATTTTCTTCCTAAGGAAGAATTGGTTCGCATGTTTGA
LigMV_gamma_CH_dsRNA      CTGTCTTAAAAAGATGGTTTGGATTCTCTTCCTAAGGAAGAATTGGTTCGCATGTTTGA
                          ************************* **********************************

LigMV_gamma_CH_sRNA       AAGAGAAACTTATCTTTTCGGGTAAGGCTGTTTACTGCACGGTAACGGCTAGATGTCCCT
LigMV_gamma_CH_dsRNA      AAGAGAAACTTATCTTTTCGGGTAAGGCTGTTTACTGCACGGTAACGGCTAGATGTCCCT
                          ************************************************************

LigMV_gamma_CH_sRNA       TAAAGTTTGACGAGGTTCGAGAAAAGGGTGTTTACAGAGATATCTTTTCGGAAAATGATG
LigMV_gamma_CH_dsRNA      TAAAGTTTGACGAGGTTCGAGAAAAGGGTGTTTACAGAGATATCTTTTCGGAAAATGATG
                          ************************************************************

LigMV_gamma_CH_sRNA       TGGTTGATGTGATGGAAATGAAGTTACAAAAACTTTTACATCATGAAGGTGACAAATGGA
LigMV_gamma_CH_dsRNA      TGGTTGATGTGATGGAAATGAAGTTACAAAAACTTTTACATCATGAAGGTGACAAATGGA
                          ************************************************************

LigMV_gamma_CH_sRNA       GGAGAAAAATTGTCTTTGATGTTCCGCGTATGTCTTCTAAGAGAGTTCCGGTTCCTAGAA
LigMV_gamma_CH_dsRNA      GGAGAAAAATTGTCTTTGATGTTCCGCGTATGTCTTCTAAGAGAGTTCCGGTTCCTAGAA
                          ************************************************************

LigMV_gamma_CH_sRNA       TTGGTACCGTAAAGTGCATTCAAAGTTGGTTTGATGCTACTTTTCCTGGTAACTCAATCA
LigMV_gamma_CH_dsRNA      TTGGTACCGTAAAGTGCATTCAAAGTTGGTTTGATGCTACTTTTCCTGGTAACTCAATCA
                          ************************************************************

LigMV_gamma_CH_sRNA       GGTTCTCAGATTTTGATGGTTACACTGTTGCAATATCGGATCTTGCCGTTGATGTGACAG
LigMV_gamma_CH_dsRNA      GGTTCTCAGATTTTGATGGTTACACTGTTGCAATATCGGATCTTGCCGTTGATGTGACAG
                          ************************************************************

LigMV_gamma_CH_sRNA       ATTGTAAAATAAAAATTGGAAAAACTTTTAAGCCGTACGTTTTTAAAAGCGCTCTGCGCC
LigMV_gamma_CH_dsRNA      ATTGTAAAATAAAAATTGGAAAAACTTTTAAGCCGTACGTTTTTAAAAGCGCTCTGCGCC
                          ************************************************************

LigMV_gamma_CH_sRNA       CTGAATTAAGGACATCAATGCCTGAAAAGAGACAAGGTAGTTTAGCCGAAAGTGTGTTGG
LigMV_gamma_CH_dsRNA      CTGAATTAAGGACATCAATGCCTGAAAAGAGACAAGGTAGTTTAGCCGAAAGTGTGTTGG
                          ************************************************************

LigMV_gamma_CH_sRNA       CTTTGAGAAAAAGAAACTTAGCAGCACCCAGATTGCAAGGGGCTGTTAACGAATGGCAGG
LigMV_gamma_CH_dsRNA      CTTTGAGAAAAAGAAACTTAGCAGCGCCCAGATTGCAAGGGGCTGTTAACGAATGGCAGG
                          ************************* **********************************

LigMV_gamma_CH_sRNA       TGATTGAAAATACTATGAAGAGGGCTTTGGATATTTTCTTTATTAAGGATTTGATTGATA
LigMV_gamma_CH_dsRNA      TGATTGAAAACACTATGAAGAGGGCTTTGGATATTTTCTTTATTAAGGATTTGATTGATA
                          ********** *************************************************

LigMV_gamma_CH_sRNA       GGTCGCACCATGCTTCCTATGAATCAGCCTTAAAATGGTGGGATAAGCAGTCAACTACTG
LigMV_gamma_CH_dsRNA      GGTCGCACCATGCTTCCTATGAATCAGCCTTAAAATGGTGGGATAAGCAGTCAACTACTG
                          ************************************************************

LigMV_gamma_CH_sRNA       CCCGTGCTCAACTTGCAGCTGAGACGAGACGACTTTGTGACATCGACTTCTGTACTTACA
LigMV_gamma_CH_dsRNA      CCCGTGCTCAACTTGCAGCAGAGACGAGACGACTTTGTGACATCGACTTCTGTACTTACA
                          ******************* ****************************************

LigMV_gamma_CH_sRNA       ATTTTATGATTAAAAATGATGTAAAACCGAAGTTGGATCTGACACCCCAAGTTGAGTATG
LigMV_gamma_CH_dsRNA      ATTTTATGATCAAAAATGATGTAAAACCGAAGTTGGATCTGACACCCCAAGTTGAGTATG
                          ********** *************************************************

LigMV_gamma_CH_sRNA       CAGCTTTGCAAACGGTAGTATTTCCTGATAAAATCGTGAATGCTTTTTTCGGCCCTGTTA
LigMV_gamma_CH_dsRNA      CAGCTTTGCAAACGGTAGTATTTCCTGATAAAATCGTGAATGCTTTTTTCGGCCCTGTTA
                          ************************************************************

LigMV_gamma_CH_sRNA       TAAAGGAAATTAATGAGAGGATCATTAGAGCCTTGAAACCTCATATTGTGTTTAATACAC
LigMV_gamma_CH_dsRNA      TAAAGGAAATTAATGAGAGGATCATTAGAGCCTTGAAACCTCATATTGTGTTTAATACAC
                          ************************************************************
```

```
LigMV_gamma_CH_sRNA       GTATGACGGCCGAAGGTTTAAATGATACTATTTCATTTCTGCCCCCCAAGTCTTACAGGT
LigMV_gamma_CH_dsRNA      GTATGACGGCCGAAGGTTTAAATGATACTATTTCATTTCTGCCCCCCAAGTCTTACAGGT
                          ************************************************************

LigMV_gamma_CH_sRNA       CACTTGAAATCGATTTCTCAAAATTTGACAAGTCAAAGACTGGTCTGCATATTAAATGTG
LigMV_gamma_CH_dsRNA      CACTTGAAATCGATTTCTCAAAATTTGACAAGTCAAAGACTGGTCTGCATATTAAATGTG
                          ************************************************************

LigMV_gamma_CH_sRNA       TAATAGCTTTATACAAATTGTTTGGACTGGATGGAATCCTAGAGGTGCTCTGGGAGAAAT
LigMV_gamma_CH_dsRNA      TAATAGCTTTATACAAATTGTTTGGACTGGATGGAATCCTAGAGGTGCTCTGGGAGAAAT
                          ************************************************************

LigMV_gamma_CH_sRNA       CTCAAGTACAAACATACGTAAAGGACCGAAATTTTGGTCTTGAAGCTTACCTTTTATTTC
LigMV_gamma_CH_dsRNA      CTCAAGTACAAACATACGTAAAGGACCGAAATTTTGGTCTTGAAGCTTACCTTTTATTTC
                          ************************************************************

LigMV_gamma_CH_sRNA       AGCAGAAATCAGGAAATTGTGACACTTACGGTTCTAATACTTGGTCTGCAGCATTAGCGT
LigMV_gamma_CH_dsRNA      AGCAGAAATCAGGAAATTGTGACACTTACGGTTCTAATACTTGGTCTGCAGCATTAGCGT
                          ************************************************************

LigMV_gamma_CH_sRNA       TGCTAGATTGTTTGCCTTTGGAGCATTCAGTCTATGCGGTTTTTGGTGGAGACGATTCTT
LigMV_gamma_CH_dsRNA      TGCTAGATTGTTTGCCTTTGGAGCATTCAGTCTATGCGGTTTTTGGTGGAGACGATTCTT
                          ************************************************************

LigMV_gamma_CH_sRNA       TGATACTATTTGAGGAGGGGTATCTTGTTAATGACCCATGCAAGCGTTTGGCTGGAACGT
LigMV_gamma_CH_dsRNA      TGATACTATTTGAGGAGGGGTATCTTGTTAATGACCCATGCAAGCGTTTGGCTGGAACGT
                          ************************************************************

LigMV_gamma_CH_sRNA       GGAATTTTGAATGCAAAATTTTTGCTTTCAATTTTCCTTCCTTTTGCGGGAAGTTTGTTG
LigMV_gamma_CH_dsRNA      GGAATTTTGAATGCAAAATTTTTGCTTTCAATTTTCCTTCCTTTTGCGGGAAGTTTGTTG
                          ************************************************************

LigMV_gamma_CH_sRNA       TCAGTGTTGACGACAAATATGTTTTTGTACCAGATGCTGCTAAGCTAATCACAAAACTGG
LigMV_gamma_CH_dsRNA      TCAGTGTTGACGACAAATATGTTTTTGTACCAGATGCTGCTAAGCTAATCACAAAACTGG
                          ************************************************************

LigMV_gamma_CH_sRNA       GGAGAACCGACGTAAAGGATGTAGAAGCTTTAAGTGAAATTTACATTTCAATTAATGACA
LigMV_gamma_CH_dsRNA      GGAGAACCGACGTAAAGGATGTAGAAGCTTTAAGTGAAATTTACATTTCAATTAATGACA
                          ************************************************************

LigMV_gamma_CH_sRNA       ATTACAGATCCTTTAAGGATTTCCGTGTTTTGGATGAGTTGAGCGATGCTTTGGTTGATA
LigMV_gamma_CH_dsRNA      ATTACAGATCCTTTAAGGATTTCCGTGTTTTGGATGAGTTGAGCGATGCTTTGGTTGATA
                          ************************************************************

LigMV_gamma_CH_sRNA       GATATCATGCTCCTTACGACGCTTTATCTGCTTTGGTGTCTCTGTGTTACTATATATTCG
LigMV_gamma_CH_dsRNA      GATATCATGCCCCTTACGACGCTTTATCTGCTTTGGTGTCTCTGTGTTACTATATTTTCG
                          ********** ******************************************** ****

LigMV_gamma_CH_sRNA       ATTTCGACAACTTTAAGCAGCTGTTTGGTTGTGATGGCAATTTTGTGGATAAAATAATTG
LigMV_gamma_CH_dsRNA      ATTTCGACAACTTTAAGCAGCTGTTTGGTTGTGATGGCAATTTTGTGGATAAAATAATTG
                          ************************************************************

LigMV_gamma_CH_sRNA       GAAAGGATTTCGATTGGTGAATCCTTGATGTATTTTTCTAACATTTTTGCTTTTTACCAA
LigMV_gamma_CH_dsRNA      GAAAGGATTTCGATTGGTGAATCCTTGATGTATTTTTCTAACATTTTTGCTTTTTACCAA
                          ************************************************************

LigMV_gamma_CH_sRNA       ATGGCAGCCAACCCAAGAGTTTGTGAATTATGTAACATTGTTTTTGACAAGACTGATACG
LigMV_gamma_CH_dsRNA      ATGGCAGCCAACCCAAGAGTTTGTGAATTATGTAACATTGTTTTTGACAAGACTGATACG
                          ************************************************************

LigMV_gamma_CH_sRNA       ACTTTCTGTTCTCCTAAATGCGAGAAGCGTGCTAGTTTGAAGAAAGAGAGACTTCGTCGT
LigMV_gamma_CH_dsRNA      ACTTTCTGTTCTCCTAAATGCGAGAAGCGTGCTAGTTTGAAGAAAGAGAGACTTCGTCGT
                          ************************************************************

LigMV_gamma_CH_sRNA       GCTGAGCTGTTTATCAAGCACGGGCTTACAGGAAAACCTTGTGGTTTAAACAAGTATCCC
LigMV_gamma_CH_dsRNA      GCTGAGCTGTTTATCAAGCACGGGCTTACAGGAAAACCTTGTGGTTTAAACAAGTATCCC
                          ************************************************************

LigMV_gamma_CH_sRNA       GGTGAATCATGTGGTATGTATGCCTATATTGCAGAAAACCAGTTACCAGATGGCAATACT
LigMV_gamma_CH_dsRNA      GGTGAATCATGTGGTATGTATGCCTTTATTGCAGAAAACCAGTTACCAGATGGCAATACT
                          ************************* **********************************

LigMV_gamma_CH_sRNA       ACCCTTACGATTTCCGAATGTTGCGGGCTTAACCACGCTGCGTATTGCTGTACGCTTGAT
LigMV_gamma_CH_dsRNA      ACCCTTACGATTTCCGAATGTTGCGGGCTTAACCACGCTGCGTATTGCTGTACGCTTGAT
                          ************************************************************

LigMV_gamma_CH_sRNA       GCTTTTGATATGAGCACACTAGAAACGAGGGCACTCATGAAGAAGCTGGAGAGGTATCGT
LigMV_gamma_CH_dsRNA      GCTTTTGATATGAGCACACTGGAAACGAGGGCTCTCATGAAGAAGCTGGAGAGGTATCGT
                          ******************** *********** ***************************

LigMV_gamma_CH_sRNA       GATGCCTCTATGGAAAAAAATGAAAGTTATAACTAACCAGTTAAAAGCGATGAAACTAAAT
LigMV_gamma_CH_dsRNA      GATGCCTCTATGGAAAAAAATGAAAGTTATAACTAACCAGTTAAAAGCGATGAAAC-----
                          *******************************************************

LigMV_gamma_CH_sRNA       TCTAAGCTTTAATAAGTTTTTCTTTTCTTTAAAAAAAAAAAAAAAAAAAAAAAAAAAAAA
LigMV_gamma_CH_dsRNA      ------------------------------------------------------------
```

CLUSTAL multiple sequence alignment by MUSCLE (3.8)

```
LigMV_gamma_AUT          -------------CTACACGACGCTCTTCCGATCTAAGGTAGAAGCGTGGAGACTCTGGT
LigMV_gamma_FR        ...CCACATCATTTTCCGAAAAGATCTTTCTGCGCTT-----------CTTTGAA-----GGT
LigMV_gamma_CH_RACE      -------------GTAAAAGATCTTTCTGCGCTT-----------CTTTGAA-----GGT
                                        * * **   *  * ** *            * * **      ***

--

LigMV_gamma_AUT          ----------------------------------------------CTACACGACGCTCT
LigMV_gamma_FR           AATGAAGGATGTGGTTGATGTGATGTAAAAGTTTTTGTAACTTCATTTCCATCACATCAA
LigMV_gamma_CH_sRNA      ------------------------------------------------------------


LigMV_gamma_AUT          TCCGATCTAAGGTAGAAG------CGTGGAGACTCT-----GGTCTGTGGGAGTTTAGAT
LigMV_gamma_FR           CCACATCATTTTCCGAAAAGATCTTTCTGCGCTTCTTTGAAGGTCTGTGGGAGTTTAGAT
LigMV_gamma_CH_sRNA      -----tccttAGcgga--------TTCcGaGCTTCTTTGAAGGTCTGTGGGAGTTTAGAT
                              **       **            * *  ***     *******************


LigMV_gamma_AUT          TTTACCAACAAACATTATCAATCTTTGATATTTTTCAATGATGGCTTTGCCTAGCTTCGC
LigMV_gamma_FR           TTTACCAACAAACATTATCAATCTTTGATATTTTTCAACGATGGCTTTGCCTAGCTTCGC
LigMV_gamma_CH_sRNA      TTTACCAACAAACATTATCAATCTTTGATATTTTTCAATGATGGCTTTGCCTAGCTTCGC
                         ************************************** *********************

LigMV_gamma_AUT          GCTCGCAAAGCCTAATCCCAAACTGTGTTTTAAGAAACTTCAAGTATTAGCATTCGTTGC
LigMV_gamma_FR           GCTCGCAAAGCCTAATCCCAAACTGTGTTTTAAGAAACTTCAAGTATTAGCATTCGTTGC
LigMV_gamma_CH_sRNA      GCTCGCAAAGCCTAATCCCAAACTGTGTTTTAAGAAACTTCAAGTATTAGCATTCGTTGC
                         ************************************************************

LigMV_gamma_AUT          GTTACTTGTGAGGTCGAAAAAAACGTTTATTCTCTCTGTCTTAAAAAGATGGTTTGGGAT
LigMV_gamma_FR           GTTACTTGTGAGGTCGAAAAAAACGTTTATTCTCTCTGTCTTAAAAAGATGGTTTGGGAT
LigMV_gamma_CH_sRNA      GTTACTTGTGAGGTCGAAAAAAACGTTTATTCTCTCTGTCTTAAAAAGATGGTTTGGGAT
                         ************************************************************

LigMV_gamma_AUT          TTTCTTCCTAAGGAAGAATTGGTTCGCATGTTTGAAAGAGAAACTTATCTTTTCGGGTAA
LigMV_gamma_FR           TTTCTTCCTAAGGAAGAATTGGTTCGCATGTTTGAAAGAGAAACTTATCTTTTCGGGTAA
LigMV_gamma_CH_sRNA      TTTCTTCCTAAGGAAGAATTGGTTCGCATGTTTGAAAGAGAAACTTATCTTTTCGGGTAA
                         ************************************************************
```

```
LigMV_gamma_AUT          GGCTGTTTACTGCACGGTAACGGCTAGATGTCCCTTAAAGTTTGACGAGGTTCGAGAAAA
LigMV_gamma_FR           GGCTGTTTACTGCACGGTAACGGCTAGATGTCCCTTAAAGTTTGACGAGGTTCGAGAAAA
LigMV_gamma_CH_sRNA      GGCTGTTTACTGCACGGTAACGGCTAGATGTCCCTTAAAGTTTGACGAGGTTCGAGAAAA
                         ************************************************************

LigMV_gamma_AUT          AGGTGTTTACAGAGATATCTTTTCGGAAAATGATGTGGTTGATGTGATGGAAATGAAGTT
LigMV_gamma_FR           AGGTGTTTACAGAGATATCTTTTCGGAAAATGATGTGGTTGATGTGATGGAAATGAAGTT
LigMV_gamma_CH_sRNA      GGGTGTTTACAGAGATATCTTTTCGGAAAATGATGTGGTTGATGTGATGGAAATGAAGTT
                          ***********************************************************

LigMV_gamma_AUT          ACAAAAACTTTTACATCATGAAGGTGACAAATGGAGGAGAAAAATTGTCTTTGATGTTCC
LigMV_gamma_FR           ACAAAAACTTTTACATCATGAAGGTGACAAATGGAGGAGAAAAATTGTCTTTGATGTTCC
LigMV_gamma_CH_sRNA      ACAAAAACTTTTACATCATGAAGGTGACAAATGGAGGAGAAAAATTGTCTTTGATGTTCC
                         ************************************************************

LigMV_gamma_AUT          GCGTATGTCTTCTAAGAGAGTTCCGGTTCCTAGAATTGGTACCGTAAAGTGCATTCAAAG
LigMV_gamma_FR           GCGTATGTCTTCTAAGAGAGTTCCGGTTCCTAGAATTGGTACCGTAAAGTGCATTCAAAG
LigMV_gamma_CH_sRNA      GCGTATGTCTTCTAAGAGAGTTCCGGTTCCTAGAATTGGTACCGTAAAGTGCATTCAAAG
                         ************************************************************

LigMV_gamma_AUT          TTGGTTTGATGCTACTTTTCCTGGTAACTCAATCAGGTTCTCAGATTTTGATGGTTACAC
LigMV_gamma_FR           TTGGTTTGATGCTACTTTTCCTGGTAACTCAATCAGGTTCTCAGATTTTGATGGTTACAC
LigMV_gamma_CH_sRNA      TTGGTTTGATGCTACTTTTCCTGGTAACTCAATCAGGTTCTCAGATTTTGATGGTTACAC
                         ************************************************************

LigMV_gamma_AUT          TGTTGCAACATCGGATCTTGCCGTTGATGTGACAGATTGTAAAATAAAAATTGGAAAAAC
LigMV_gamma_FR           TGTTGCAATATCGGATCTTGCCGTTGATGTGACAGATTGTAAAATAAAAATTGGAAAAAC
LigMV_gamma_CH_sRNA      TGTTGCAATATCGGATCTTGCCGTTGATGTGACAGATTGTAAAATAAAAATTGGAAAAAC
                         ******** ***************************************************

LigMV_gamma_AUT          TTTTAAGCCGTACGTTTTTAAAAGCGCTCTGCGCCCTGAATTAAGGACATCAATGCCTGA
LigMV_gamma_FR           TTTTAAGCCGTACGTTTTTAAAAGCGCTCTGCGTCCTGAATTAAGGACATCAATGCCTGA
LigMV_gamma_CH_sRNA      TTTTAAGCCGTACGTTTTTAAAAGCGCTCTGCGCCCTGAATTAAGGACATCAATGCCTGA
                         ********************************* **************************

LigMV_gamma_AUT          AAAGAGACAAGGTAGTTTAGCCGAAAGTGTGTTGGCTTTGAGAAAAAGAAACTTAGCAGC
LigMV_gamma_FR           AAAGAGACAAGGTAGTTTAGCCGAAAGTGTGTTGGCTTTGAGAAAAAGAAACTTAGCAGC
LigMV_gamma_CH_sRNA      AAAGAGACAAGGTAGTTTAGCCGAAAGTGTGTTGGCTTTGAGAAAAAGAAACTTAGCAGC
                         ************************************************************

LigMV_gamma_AUT          GCCCAGATTGCAAGGGGCTGTTAACGAATGGCAGGTGATTGAAAACACTATGAAGAGGGC
LigMV_gamma_FR           GCCCAGATTGCAAGGGGCTGTTAACGAATGGCAGGTGATTGAAAACACTATGAAGAGGGC
LigMV_gamma_CH_sRNA      ACCCAGATTGCAAGGGGCTGTTAACGAATGGCAGGTGATTGAAAATACTATGAAGAGGGC
                          ******************************************** **************

LigMV_gamma_AUT          TTTGGATATTTTCTTTAATAAGGATTTGATTGATAGGTCGCACCATGCTTCCTATGAATC
LigMV_gamma_FR           TTTGGATATTTTCTTTATTAAGGATTTGATTGATAGGTCGCACCATGCTTCCTATGAATC
LigMV_gamma_CH_sRNA      TTTGGATATTTTCTTTATTAAGGATTTGATTGATAGGTCGCACCATGCTTCCTATGAATC
                         ***************** ******************************************

LigMV_gamma_AUT          AGCCTTAAAATGGTGGGATAAGCAGTCAACTACTGCCCGTGCTCAACTTGCAGCAGAGAC
LigMV_gamma_FR           AGCCTTAAAATGGTGGGATAAGCAGTCAACTACTGCCCGTGCTCAACTTGCAGCAGAGAC
LigMV_gamma_CH_sRNA      AGCCTTAAAATGGTGGGATAAGCAGTCAACTACTGCCCGTGCTCAACTTGCAGCTGAGAC
                         ****************************************************** *****

LigMV_gamma_AUT          GAGACGACTTTGTGACATCGACTTCTGTACTTACAATTTTATGATCAAAAATGATGTAAA
LigMV_gamma_FR           GAGACGACTTTGTGACATCGACTTCTGTACTTACAATTTTATGATCAAAAATGATGTAAA
LigMV_gamma_CH_sRNA      GAGACGACTTTGTGACATCGACTTCTGTACTTACAATTTTATGATTAAAAATGATGTAAA
                         ********************************************* **************

LigMV_gamma_AUT          ACCGAAGTTGGATCTGACACCCCAAGTTGAGTATGCAGCTTTGCAAACGGTAGTATTTCC
LigMV_gamma_FR           ACCGAAGTTGGATCTGACACCCCAAGTTGAGTATGCAGCTTTGCAAACGGTAGTATTTCC
LigMV_gamma_CH_sRNA      ACCGAAGTTGGATCTGACACCCCAAGTTGAGTATGCAGCTTTGCAAACGGTAGTATTTCC
                         ************************************************************

LigMV_gamma_AUT          TGATAAAATCGTGAATGCTTTTTTCGGCCCTGTTATAAAGGAAATTAATGAGAGGATCAT
LigMV_gamma_FR           TGATAAAATCGTGAATGCTTTTTTCGGCCCTGTTATAAAGGAAATTAATGAGAGGATCAT
LigMV_gamma_CH_sRNA      TGATAAAATCGTGAATGCTTTTTTCGGCCCTGTTATAAAGGAAATTAATGAGAGGATCAT
                         ************************************************************

LigMV_gamma_AUT          TAGAGCCTTGAAACCTCATATTGTGTTTAATACACGTATGACGGCCGAAGGTTTAAATGA
LigMV_gamma_FR           TAGAGCCTTGAAACCTCATATTGTGTTTAATACACGTATGACGGCCGAAGGTTTAAATGA
LigMV_gamma_CH_sRNA      TAGAGCCTTGAAACCTCATATTGTGTTTAATACACGTATGACGGCCGAAGGTTTAAATGA
                         ************************************************************

LigMV_gamma_AUT          TACTATTTCATTTCTGCCCCCCAAGTCTTACAGGTCACTTGAAATCGATTTCTCAAAATT
LigMV_gamma_FR           TACTATTTCATTTCTGCCCCCCAAGTCTTACAGGTCACTTGAAATCGATTTCTCAAAATT
LigMV_gamma_CH_sRNA      TACTATTTCATTTCTGCCCCCCAAGTCTTACAGGTCACTTGAAATCGATTTCTCAAAATT
                         ************************************************************

LigMV_gamma_AUT          TGACAAGTCAAAGACTGGTCTGCATATTAAATGTGTAATAGCTTTATACAAATTGTTTGG
LigMV_gamma_FR           TGACAAGTCAAAGACTGGTCTGCATATTAAATGTGTAATAGCTTTATACAAATTGTTTGG
LigMV_gamma_CH_sRNA      TGACAAGTCAAAGACTGGTCTGCATATTAAATGTGTAATAGCTTTATACAAATTGTTTGG
                         ************************************************************

LigMV_gamma_AUT          ACTGGATGGAATCCTAGAGGTGCTCTGGGAGAAATCTCAAGTACAAACATACGTAAAGGA
LigMV_gamma_FR           ACTGGATGGAATCCTAGAGGTGCTCTGGGAGAAATCTCAAGTACAAACATACGTAAAGGA
LigMV_gamma_CH_sRNA      ACTGGATGGAATCCTAGAGGTGCTCTGGGAGAAATCTCAAGTACAAACATACGTAAAGGA
                         ************************************************************

LigMV_gamma_AUT          CCGAAATTTTGGTCTTGAAGCTTACCTTTTATTTCAGCAGAAATCAGGAAATTGTGACAC
LigMV_gamma_FR           CCGAAATTTTGGTCTTGAAGCTTACCTTTTATTTCAGCAGAAATCAGGAAATTGTGACAC
LigMV_gamma_CH_sRNA      CCGAAATTTTGGTCTTGAAGCTTACCTTTTATTTCAGCAGAAATCAGGAAATTGTGACAC
                         ************************************************************

LigMV_gamma_AUT          TTACGGTTCTAATACTTGGTCTGCAGCATTAGCGTTGCTAGATTGTTTGCCTTTGGAGCA
LigMV_gamma_FR           TTACGGTTCTAATACTTGGTCTGCAGCATTAGCGTTGCTAGATTGTTTGCCTTTGGAGCA
LigMV_gamma_CH_sRNA      TTACGGTTCTAATACTTGGTCTGCAGCATTAGCGTTGCTAGATTGTTTGCCTTTGGAGCA
                         ************************************************************

LigMV_gamma_AUT          TTCAGTCTATGCGGTTTTTGGTGGAGACGATTCTTTGATACTATTTGAGGAGGGGTATCT
LigMV_gamma_FR           TTCAGTCTATGCGGTTTTTGGTGGAGACGATTCTTTGATACTATTTGAGGAGGGGTATCT
LigMV_gamma_CH_sRNA      TTCAGTCTATGCGGTTTTTGGTGGAGACGATTCTTTGATACTATTTGAGGAGGGGTATCT
                         ************************************************************

LigMV_gamma_AUT          TGTTAATGACCCATGCAAGCGTTTGGCTGGAACGTGGAATTTTGAATGCAAAATTTTTGC
LigMV_gamma_FR           TGTTAATGACCCATGCAAGCGTTTGGCTGGAACGTGGAATTTTGAATGCAAAATTTTTGC
LigMV_gamma_CH_sRNA      TGTTAATGACCCATGCAAGCGTTTGGCTGGAACGTGGAATTTTGAATGCAAAATTTTTGC
                         ************************************************************

LigMV_gamma_AUT          TTTCAATTTTCCTTCCTTTTGCGGGAAGTTTGTTGTCAGTGTTGACGACAAATATGTTTT
LigMV_gamma_FR           TTTCAATTTTCCTTCCTTTTGCGGGAAGTTTGTTGTCAGTGTTGACGACAAATATGTTTT
LigMV_gamma_CH_sRNA      TTTCAATTTTCCTTCCTTTTGCGGGAAGTTTGTTGTCAGTGTTGACGACAAATATGTTTT
                         ************************************************************

LigMV_gamma_AUT          TGTACCAGATGCTGCTAAGCTAATCACAAAACTGGGGAGAACCGACGTAAAGGATGTAGA
LigMV_gamma_FR           TGTACCAGATGCTGCTAAGCTAATCACAAAACTGGGGAGAACCGATGTAAAGGATGTAGA
LigMV_gamma_CH_sRNA      TGTACCAGATGCTGCTAAGCTAATCACAAAACTGGGGAGAACCGACGTAAAGGATGTAGA
                         ********************************************* **************

LigMV_gamma_AUT          AGCTTTAAGTGAAATTTACATTTCAATTAATGACAATTACAGATCCTTTAAGGATTTCCG
LigMV_gamma_FR           AGCTTTAAGTGAAATTTACATTTCAATTAATGACAATTACAGATCCTTTAAGGATTTCCG
LigMV_gamma_CH_sRNA      AGCTTTAAGTGAAATTTACATTTCAATTAATGACAATTACAGATCCTTTAAGGATTTCCG
                         ************************************************************

LigMV_gamma_AUT          TGTTTTGGATGAGTTGAGCGATGCTTTGGTTGATAGATATCATGCCCCTTACGACGCTTT
LigMV_gamma_FR           TGTTTTGGATGAGTTGAGCGATGCTTTGGTTGATAGATATCATGCCCCTTACGACGCTTT
LigMV_gamma_CH_sRNA      TGTTTTGGATGAGTTGAGCGATGCTTTGGTTGATAGATATCATGCTCCTTACGACGCTTT
```

```
                         ********************************************* **************

LigMV_gamma_AUT          ATCTGCTTTGGTGTCTCTGTGTTACTATATATTCGATTTCGACAACTTTAAGCAGCTGTT
LigMV_gamma_FR           ATCTGCTTTGGTGTCTCTGTGTTACTATATATTCGATTTCGACAACTTTAAGCAGCTGTT
LigMV_gamma_CH_sRNA      ATCTGCTTTGGTGTCTCTGTGTTACTATATATTCGATTTCGACAACTTTAAGCAGCTGTT
                         ************************************************************

LigMV_gamma_AUT          TGGTTGTGATGGCAATTTTGTGGATAAAATAATTGGAAAGGATTTCGATTGGTGAATCCT
LigMV_gamma_FR           TGGTTGTGATGGCAATTTTGTGGATAAAATAATTGGAAAGGATTTCGATTGGTGAATCCT
LigMV_gamma_CH_sRNA      TGGTTGTGATGGCAATTTTGTGGATAAAATAATTGGAAAGGATTTCGATTGGTGAATCCT
                         ************************************************************

LigMV_gamma_AUT          TGATGTATTTTTCTAACATTTTTGC-TTTTTACCAAATGGCAGCCAACCCAAGAGTTTGT
LigMV_gamma_FR           TGATGTATTTTTCTAACACTTTTGCTTTTTTACCAAATGGCAGCCAACCCGAGAGTTTGT
LigMV_gamma_CH_sRNA      TGATGTATTTTTCTAACATTTTTGC-TTTTTACCAAATGGCAGCCAACCCAAGAGTTTGT
                         ****************** ****** ************************ *********

LigMV_gamma_AUT          GAATTATGTAACATTGTTTTTGACAAGACTGATACGACTTTCTGTTCTCCTAAATGCGAG
LigMV_gamma_FR           GAATTATGTAACATTGTTTTTGACAAGACTGATACGACTTTCTGTTCTCCTAAATGCGAG
LigMV_gamma_CH_sRNA      GAATTATGTAACATTGTTTTTGACAAGACTGATACGACTTTCTGTTCTCCTAAATGCGAG
                         ************************************************************

LigMV_gamma_AUT          AAGCGTGCTAGTTTGAAGAAAGAGAGACTTCGTCGTGCTGAGCTGTTTATCAAGCACGGG
LigMV_gamma_FR           AAGCGTGCTAGTTTGAAGAAAGAGAGACTTCGTCGTGCTGAGCTGTTTATCAAGCACGGG
LigMV_gamma_CH_sRNA      AAGCGTGCTAGTTTGAAGAAAGAGAGACTTCGTCGTGCTGAGCTGTTTATCAAGCACGGG
                         ************************************************************

LigMV_gamma_AUT          CTTACAGGAAAACCTTGTGGTTTAAACAAGTATCCCGGTGAATCATGTGGTATGTATGCC
LigMV_gamma_FR           CTTACAGGAAAACCTTGTGGTTTAAACAAGTATCCCGGTGAATCATGTGGTATGTATGCC
LigMV_gamma_CH_sRNA      CTTACAGGAAAACCTTGTGGTTTAAACAAGTATCCCGGTGAATCATGTGGTATGTATGCC
                         ************************************************************

LigMV_gamma_AUT          TATATTGCAGAAAACCAGTTACCAGATGGCAATACTACCCTTACGATTTCCGAATGTTGC
LigMV_gamma_FR           TATATTGCAGAAAACCAGTTACCAGATGGCAATACTACCCTTACGATTTCCGAATGTTGC
LigMV_gamma_CH_sRNA      TATATTGCAGAAAACCAGTTACCAGATGGCAATACTACCCTTACGATTTCCGAATGTTGC
                         ************************************************************

LigMV_gamma_AUT          GGGCTTAACCACGCTGCGTATTGCTGTACGCTTGATGCTTTTGATATGAGCACACTGGAA
LigMV_gamma_FR           GGGCTTAACCACGCTGCGTATTGCTGTACGCTTGATGCTTTTGATATGAGCACACTGGAA
LigMV_gamma_CH_sRNA      GGGCTTAACCACGCTGCGTATTGCTGTACGCTTGATGCTTTTGATATGAGCACACTAGAA
                         ******************************************************** ***

LigMV_gamma_AUT          ACGAGGGCTCTCATGAAGAAGCTGGAGAGGTATCGTGATGCCTCTATGGAAAAAATGAAA
LigMV_gamma_FR           ACGAGGGCTCTCATGAAGAAGCTGGAGAGGTATCGTGATGCCTCTATGGAAAAAATGAAA
LigMV_gamma_CH_sRNA      ACGAGGGCTCTCATGAAGAAGCTGGAGAGGTATCGTGATGCCTCTATGGAAAAAATGAAA
                         ************************************************************

LigMV_gamma_AUT          GTTATAACTAACCAGTTAAAAGCGATGAAACTAAATTCTAAGCTTTAATAAGTTTTTCTT
LigMV_gamma_FR           GTTATAACTAACCAGTTAAAAGCGATGAAACTAAATTCTAAGCTTTAATAAGTTTTTCTT
LigMV_gamma_CH_sRNA      GTTATAACTAACCAGTTAAAAGCGATGAAACTAAATTCTAAGCTTTAATAAGTTTTTCTT
                         ************************************************************

LigMV_gamma_AUT          TTCTTTTTAAAAAAAAAAAAAAAAAAAAAAAAAAAAAAAAAAAAAAAAAAAAAAAAGATG
LigMV_gamma_FR           TTCTTTTTAAAAAAAAAAAAAAAAAAAAA-------------------------------
LigMV_gamma_CH_sRNA      TTCTTTAAAAAAAAAAAAAAAAAAAAAAAAAAAAAAAAAAAAAAAAAAAAAAAAAAAAAA
                         ******  *********************

LigMV_gamma_AUT          ---------------TTTTAATAAGAGAAAAAAATAAAAAAGATAAAAAAGAAGAAATAA
LigMV_gamma_FR           ------------------------------------------------------------
LigMV_gamma_CH_sRNA      AAAAATAATGCCTGCTATTAAGACGGTGAAACCTATAAACGGGTTCAACGGGTGAGATAG


LigMV_gamma_AUT          ------------------------------------------------------------
LigMV_gamma_FR           ------------------CCCAAACCCCCGCG----------------------------
LigMV_gamma_CH_sRNA      TGGGAATTGGTGTGTAAGCGCGTACTCCTACGTCGCTGCATCAACAATGATCTGTGGATC


LigMV_gamma_AUT          ---------------------------ATTTTAT---------------
LigMV_gamma_FR           ---------------------------GTTTTG----------------
LigMV_gamma_CH_sRNA      GATTGTTGCACACTGAGCCGGAGCTCAGCTTTGGTCCTTTAGGAGACCA
                                                       ***
```

Dataset S1B. Comparison of 5′-UTRs of LigMV, PSLV, LRSV and BSMV

```
>LigMV_alpha
GTAATGTTTACTGCGGTAGTTTGAGTACTGCGACAGTGGTTTTGCATTAACAGTTAATCCACTTCAGGTTTTGTTCTTTTCAACATTTTCATTCCGTGGTCATAATCATGGCT
>MF768253_LRSV_mild Lychnis ringspot virus isolate mild segment RNA alpha, complete sequence
GGTATTGTTTACTGCGGTAGTTTACTGCGTCAGTGGTTTTGCAATAACAGTTTAATCGACTTTAAGATTTCTCTTCTTTCAACAACTTCTTTCCACAGCCGAAATGGCG
>MG913809_LRSV_SN Lychnis ringspot virus strain SN segment RNA alpha, complete sequence
GGTATTGTTTACTGCGGTAGTTTACTGCGTCAGTGGTTTTGCAATAACAGTTTAATCAACTAAAAGATTATTTTTCTTTCAACAATTAAATTCCGCAGTCGAAATGGCG
>AY789693_BSMV_China Barley stripe mosaic virus segment RNA1, complete sequence
GTATGTAAGTTGCCTTTGGGTGTAAAATTTCTTGCATGCACATAATCGTAATCGATTCTTCTTGATCTCTAAACAACACTTTCCCGTTAGCATGGCT
>U35767_BSMV_ND18 Barley stripe mosaic virus ND18 RNA alpha, alpha protein gene, complete cds
GTATGTAAGTTGCCTTTGGGTGTAAAATTTCTTGCATGCACATAATCGTAATCGATTCTTCTTGATCTCTAAACAACACTTTCCCGTTAGCATGGCT
>MK377386_PSLV_Canada Poa semilatent virus isolate Canada segment alpha, complete sequence
GGTATTCGAGAGAGAGTTCGCGGGCTCATTGAAATCTCGGGTACTCATAATCGTGAAATCGATTCGGTTCCTTTTAACTTCACAACAAACTTTTCCAGCTTGTCATCTCCGAGGCGATGGCA
```

```
LigMV_alpha           -GTAATGTTTACTGCGGTAGTTTGAGTACTGCGACAGTGGTTTTGCATTAACAG-TTAATCCACTTC-AGGTTTTGTTCTTTTCAACATTTTCATTCCGTGGTCATAATCATGGCT
MF768253_LRSV_mild    GGTATTGTTTACTGCGGTAGTT----TACTGCGTCAGTGGTTTTGCAATAACAGTTTAATCGACTTTAAGATTTCTCTTCTTTCAACAACTTCTTTCCACAGCCGAA---ATGGCG
MG913809_LRSV_SN      GGTATTGTTTACTGCGGTAGTT----TACTGCGTCAGTGGTTTTGCAATAACAGTTTAATCAACTAAAAGATTATTTTTCTTTCAACAATTAAATTCCGCAGTCGAA---ATGGCG
                       *** *****************    ******* ************* ****** ****** ***   ** **    *  ********  *   ****   * *  *   *****

LigMV_alpha           -GTAATGTTTACTGCGGTAGTTTGAG----TACTGCGACAGTGGTTTTGCATTAACAGTTAATCCACTTCAGGTTTTGTT-------------CTTTTCAACATTTTCAT-TCCGTGGTCATAATCATGGCT
MK377386_PSLV_Canada  GGTA----TTCGAGAGAGAGTTCGCGGGCTCATTGAAATCTCGGGTACTCA-TAATCGTGAAATCGATTCGGTTCCTTTTAACTTCACAACAAACTTTTCCAGCTTGTCATCTCCGAGG------CGATGGCA
                       ***    **   * *  **** * *     * **  *    ** *   ** ***  ** **  *  *** * *  * **               ****** *  ** **** **** **         *****

LigMV_alpha            GTAATGTTTACTGCGGTAGTTTGAGTACTGCGACAGTGGTTTTGCATTAACAGTTAATCCACTTCAGGTTTTGTTCTTTT-----CAACATTTTCATTCCGTGGTCATAATCATGGCT
AY789693_BSMV_China    GT-ATGTAAGTTGC----CTTTGGG---TGTAAAATT--TCTTGCATGCACA--TAATCGTAATCGATTCTTCTTGATCTCTAAACAACACTTTC---------CCGTTAGCATGGCT
U35767_BSMV_ND18       GT-ATGTAAGTTGC----CTTTGGG---TGTAAAATT--TCTTGCATGCACA--TAATCGTAATCGATTCTTCTTGATCTCTAAACAACACTTTC---------CCGTTAGCATGGCT
                       ** ****    ***     **** *   **  * * *  * ******  ***  *****    **   * ** **  * *     ***** ****          * * * *******


LigMV_alpha             -GTAA------TGTTTACTGCGGTAG--TTTGAGTACTGCGACAGTG--GTTTTGCA-----TTAACAGTTAATCCACTTCAGGTTTTGTT--CTTTTCAACATTTTCATTC-------CGTGGTCATAATCATGGCT
MF768253_LRSV_mild      GGTAT------TGTTTACTGCGGTAG--TT----TACTGCGTCAGTG--GTTTTGCA-----TTAACAGTTAATCCACTTCAGGTTTTGTT--CTTTTCAACATTTTCATTC-------CACAGCCGAA---ATGGCG
MG913809_LRSV_SN        GGTAT------TGTTTACTGCGGTAG--TT----TACTGCGTCAGTG--GTTTTGCA-----ATAACAGTTTAATCAACTAAAAGATTATTTTTCTTTCAACAATTAAATTC-------CGCAGTCGAA---ATGGCG
AY789693_BSMV_China     -GTATGTAAGTTGCCTTTGGGTGTAAAATT-------------------TCTTGCATGCACATAATCGT--AATCGATTC-----TTCTTGATCTCTAAACAACACTTTCC-------------CGTTAGCATGGCT
U35767_BSMV_ND18        -GTATGTAAGTTGCCTTTGGGTGTAAAATT-------------------TCTTGCATGCACATAATCGT--AATCGATTC-----TTCTTGATCTCTAAACAACACTTTCC-------------CGTTAGCATGGCT
MK377386_PSLV_Canada    GGTAT-----------TCGAGAGAGAGTT----CGCGGGCTCATTGAAATCTCGGGTACTCATAATCGTGAAATCGATTCGGTTCCTTTTAACTTCACAACAAACTTTTCCAGCTTGTCATCTCCGAGGCGATGGCA
                         ***               *    *   **                     * * *        ***  **  *  *   *        *  *    *   ****       * *               *      *****
```

```
>LigMV_beta
GTAAAAATACCAACAATCGTAAAGAGCTTGCTTTGCTCCTCTTTACAAAATCTATCCTTTTATATTAGTAATAGGTTTTTGTATTTTTACGTTTACAATGGCT
>MF768254_LRSV_mild Lychnis ringspot virus isolate mild segment RNA beta, complete sequence
GGTATTGTTTACTGCGGCAGTTTACTGCGTCAGTGGTTTTGCAATAACAGTTTAATCGACTTTAAGATGTAAAACATACCAGCAATCGAAGAGGACTGGCTGAAAAGCACTTCTCTTCACCTATCTTATATTAGTATTAGGTTTTTCTGTTTTTGTTTTTCCTTACATTACCTGTCAT
GGCT
>NC_038932_LRSV_type Lychnis ringspot virus RNA for beta-A, beta-B, beta-C, beta-D proteins
TTTACTGCGTCAGTGGTTTTGCAATAACAGTTTAATCGACTTTAAGATGTAAAACATACCAGCAATCGAAGAGGACTGGCTGAAAAGCACTTCTCTTCACCTATCTTATATTAGTATTAGGTTTTTCTGTTTTTGTTTTTCCTTACATTACCTGTCATGGCT
>MG913810_LRSV_SN Lychnis ringspot virus strain SN segment RNA beta, complete sequence
GGTAAAAATTACCAGCAATCGAAGAGAACTGGCTGAAAAGCCCTTCTCTTCACCTATCCTTTATTAGTATTAGGTTTATAGTAGTTTTTATCTTCTTCCTTTTCTGTCATGGCT
>AY789694_BSMC_China Barley stripe mosaic virus segment RNA2, complete sequence
GTAAAAGAAAAGGAACAACCCTGTTGTTGTTCGACACTAAACTAAATATATATTATCTTATTAGTGCATTTCTTTTACCGCTTCACAGTATGCCG
>U35770_BSMV_ND18 Barley stripe mosaic virus ND18 RNA beta, coat protein, beta-b, beta-c, and beta-d protein genes, complete cds
GTAAAAGAAAAGGAACAACCCTGTTGTTGTTCGACGCTATACTAAATATATATTATCTTATTAGTGCATTTCTTTTACCACTTCACAGTATGCCG
>MK377387_PSLV_Canada Poa semilatent virus isolate Canada segment beta, complete sequence
GGAAAAACAAACAGCGACCTGGTCGTTGTGCGAAACTCTACCAGTTTCGCGTTCTTTTAGCTAGTGTATATATTAGGATTAGAATTTGTTTTTTCAACCCGTGCATTTCATTATGCCG
```

```
LigMV_beta               -GTAAAA-ATACCAACAATCGTAAAGAGCTTGCT-----TTGCTCCTCTTTACAAAATCTATCCTTTTATATTAGTAATAGGTTTTTGT--ATTTTTACGTT------------TACAATGGCT
MF768254_LRSV_mild  ..AGATGTAAAACATACCAGCAATCGAAGAGGACTGGCTGAAAAGCACTTCTCTTCAC-----CTATC---TTATATTAGTATTAGGTTTTTCT--GTTTTTGTTTTTCCTTACATTACCTGTCATGGCT
NC_038932_LRSV_type ..AGATGTAAAACATACCAGCAATCGAAGAGGACTGGCTGAAAAGCACTTCTCTTCAC-----CTATC---TTATATTAGTATTAGGTTTTTCT--GTTTTTGTTTTTCCTTACATTACCTGTCATGGCT
```

```
MG913810_LRSV_SN         GGTAAAAATTACCAGCAATCGAAGAGAACTGGCTGAAAAGCCCTTCTCTTCAC-----CTATC---CTTTATTAGTATTAGGTTTATAGTAGTTTTTATCTT---CTTCCTTTTCTGTCATGGCT
                          ******  ***** ****** * **  ** ***        ** ***** **     *****    * ******** ******* *     *****   **               *   ******

LigMV_beta                GTAAAAATA---------CCAACAATCGTAAAGAGCTT-----GCTTTGCTCCTCTTTACAAAATCTATCCTTTTATATTAGTAATAGGTTTTTGTATTTTTACGTTT-ACAATGGCT
MK377387_PSLV_Canada      GGAAAAACAAACAGCGACCTGGTCGTTGTGCGAAACTCTACCAGTTTCGCGTTCTTTTAGCTAGTGTATATATTAGGATTAGAATTTGTTTTTTCAACCCGTGCATTTCATTATGCCG
                          * ***** *         *      * **    * **      * ** **     ****   * * ***   **   ***** * * * *****  *    * * *** *  *** *

LigMV_beta               GTAAA--AATACCAACAATCGTAAAGAGCTTGCTTTGCTCCTCTTTACAAAATCTATCCTTTTATATTAGTAATAGGTTTTTGTATTTTTACGTTTACA--ATGGCT
AY789694_BSMC_China      GTAAAAGAAAAGGAACAACCCTG------TTGTTGTTCGACACTAAACTAAATATATATTATCTTATTAGT-----GCATTTCTTTTACCGC-TTCACAGTATGCCG
U35770_BSMV_ND18         GTAAAAGAAAAGGAACAACCCTG------TTGTTGTTCGACGCTATACTAAATATATATTATCTTATTAGT-----GCATTTCTTTTACCAC-TTCACAGTATGCCG
                         *****  ** *  ***** * *       *** * * *  * **  ** **** ***  * *  *******     *  *** * **    * ** ***  *** *
```

```
MK377387_PSLV_Canada      -GGAAAA-ACAAACAGCGAC---------CTGGTCGTTGTGCGAAACTCTACCAGTTTCGCG---TTCTTTTAGCTAGTGTATATATTAGGATTAGAATTTGTTTTTTCAACCCGTGCATTT--CATTATGCCG
AY789694_BSMC_China       -GTAAAAGAAAAGGAACAAC---------CCTGTTGTTGTTCGACACTAAACTAAATATATA---TTATCTTAT----------TAGTGCATTTCT-------TTTACCGC---------TTCACAGTATGCCG
U35770_BSMV_ND18          -GTAAAAGAAAAGGAACAAC---------CCTGTTGTTGTTCGACGCTATACTAAATATATA---TTATCTTAT----------TAGTGCATTTCT-------TTTACCAC---------TTCACAGTATGCCG
LigMV_beta                -GTAAAA--ATACCAACAATCGTAAAGAGCTTGCTT---TGCTCCTCTTTACAAAATCTATCCTTTTATATTAG----------TAATAGGTTTTT--GTATTTTTACGTT------------TACAATGGCT
MF768254_LRSV_mild      ..AGATGTAAAA-CATACCAGCAATCGAAGAGGACTGGCTGAAAAGCACTTCTCTTC---ACCTATC---TTATATTAG----------TATTAGGTTTTT--CTGTTTTTGTTTTTCCTTACATTACCTGTCATGGCT
NC_038932_LRSV_type     ..AGATGTAAAA-CATACCAGCAATCGAAGAGGACTGGCTGAAAAGCACTTCTCTTC---ACCTATC---TTATATTAG----------TATTAGGTTTTT--CTGTTTTTGTTTTTCCTTACATTACCTGTCATGGCT
MG913810_LRSV_SN          GGTAAAA-ATTACCAGCAATCGAAGAGAACTGGCTGAAAAGCCCTTCTCTTC---ACCTATC---CTTTATTAG----------TATTAGGTTTATAGTAGTTTTTATCTT---CTTCCTTTTCTGTCATGGCT
                           * ****    *  * * *          *  *        *    **   *              * * ***            ** *    **          ***                     *** *
```

>LigMV_gamma
GTAAAAGATCTTTCTGCGCTTCTTTGAAGGTCTGTGGGAGTTTAGATTTACCAACAAACATTATCAATCTTTGATATTTTTCAATG**ATG**GCT
>MF768255_LRSV_mild Lychnis ringspot virus isolate mild segment RNA gamma, complete sequence
GGTATTTACCTTTCCGCGCTTCTGTTGAAGGTCTGTGGGAGTTTAGGTCATACCAACAACCGACGATTTTTTCGTTTTTTCGCG**ATG**ACC
>MG913811_LRSV_SN Lychnis ringspot virus strain SN segment RNA gamma, complete sequence
GGTATTTACCTTTCTGCGCTTCTGTTAGAAGGTCTGTGGGAGTTTAGGTCATACCAACACACAACGTTTTCACGTTTTTTCACG**ATG**ACC
>U13917_BSMV_ND18 Barley stripe mosaic virus ND18 RNA gamma segment, complete sequence
GTATAGCTTGAGCATTACCGTCGTGTAATTGCAACACTTGGCTTGCCAAATAACGCTAAAGCGTTCACGAAACAAACAACACTTCGGC**ATG**GAT
>AY787207_BSMV_China Barley stripe mosaic virus segment RNA3, complete sequence
GTATAGCTTGAGCATTACCGTCGTGTAATTGCAACACTTGGCTTACCAAATAACGCTAAAGCGTTCGCGAAACAAATCAAAACCTTGGC**ATG**GAT
>MK377388_PSLV_Canada Poa semilatent virus isolate Canada segment gamma, complete sequence
GGTATTGCTTTTGCGGCAACGCGTCCAGCAAAGTACAGCATACCAACCCCCAAAACATTAACGCGTGTAGCGTTCTTTACCCCAACAACCG**ATG**GCT

```
LigMV_gamma            GTAAAAGATCTTTCTGCGCTTCTTT--GAAGGTCTGTGGGAGTTTAGATTTACCAACAAACATTATCAATCTTTGATATTTTTCAATGATGGCT
MF768255_LRSV_mild     GGTATTTACCTTTCCGCGCTTCTGTT-GAAGGTCTGTGGGAGTTTAGGTCATACCAACAACCGACGA----TTTTTTCGTTTTTTCGCGATGACC
MG913811_LRSV_SN       GGTATTTACCTTTCTGCGCTTCTGTTAGAAGGTCTGTGGGAGTTTAGGTCATACCAACACACAACG-----TTTTCACGTTTTTTCACGATGACC
                       *  *   * ***** ******** *  ****************** *  ******** *          ***    *****    **** *

LigMV_gamma            GTAAAAGATCTTTCTGCGCTTCTTTGAAGGTCTGTGGGAGTTTAGATTTACCAAC-----AAACATTATCAATCTTTGATATTTTTC---------AATGATGGCT
MK377388_PSLV_Canada   GGTATTGCTTTTGCGGC---------AACGCGTCCAGCAAAGTACAGCATACCAACCCCCAAAACATTAAC-GCGTGTAGCGTTCTTTACCCCAACAACCGATGGCT
                       *  *  * * ** * **         ** *  *   * *   ** *  *******     ******** *    * *    ** **          *  *******

LigMV_gamma            GTAAA-----AGATCTTTCTGCGCTTCTTTGAAGGTCTGTGGGAGTTTAGATTTACCAACAAACATTATCAATCTTT---GATATTTTTCAATG-------ATGGCT
U13917_BSMV_ND18       GTATAGCTTGAGCATTACCGTCGTGTAATTGCAACACT-TGG----------CTTGCCAAATAACGCTA--AAGCGTTCACGAAACAAA-CAACACTTCGGCATGGAT
AY787207_BSMV_China    GTATAGCTTGAGCATTACCGTCGTGTAATTGCAACACT-TGG----------CTTACCAAATAACGCTA--AAGCGTTCGCGAAACAAATCAAAACCTTGGCATGGAT
                       *** *     **   *  *  **  *  *** *   ** ***            ** ****  ***  **  ** * **   ** *     ***          **** *

U13917_BSMV_ND18       -GTATA-----------GCTTGAGCATTACCGTCGTGTAATTGCAACACTTGGCTTGCCAAA-----TAACGCTAAA--------GCGTTCACGAAACAAA-CAACACTTCGGC----------ATGGAT
AY787207_BSMV_China    -GTATA-----------GCTTGAGCATTACCGTCGTGTAATTGCAACACTTGGCTTACCAAA-----TAACGCTAAA--------GCGTTCGCGAAACAAATCAAAACCTTGGC----------ATGGAT
MK377388_PSLV_Canada   GGTATT-----------GCTTTTGCGGCAACGCG-TCCA---GCAAAGTACAGCATACCAACCCCCAAAACATTAAC-GCGTGTAGCGTT-----------------CTTTACCCCAACAACCGATGGCT
LigMV_gamma            GTAAAAGATCTTTCTGCGCTTCTTT--GAAGGTC-TGTG---GGAGTTTAGATTTACCAAC-----AAACATTATCAATCTTTGATATT-----------------TTTCAAT---------GATGGCT
MF768255_LRSV_mild     GGTATTTACCTTTCCGCGCTTCTGT-TGAAGGTC-TGTG---GGAGTTTAGGTCATACCAAC-----AACCGACGAT----TTTTTCGTT-----------------TTTTCGC---------GATGACC
MG913811_LRSV_SN       GGTATTTACCTTTCTGCGCTTCTGTTAGAAGGTC-TGTG---GGAGTTTAGGTCATACCAAC--ACACAACGTTTTC--------ACGTT-----------------TTTTCAC---------GATGACC
                          *              ****       *  *   *       * *           * ****       * *                   **                    *
```

LigMV sgRNA leaders

Beta-b GATGTTTTTTGTGTTTGTTAACC**ATG**TCA

Beta-dc AAACTCTCATAAATACGGTGACATAAAATATTCTC**ATG**CCG

Beta-c AGAATAAATTACAATAGAAATAACGATCTTTCTTACGGTTACAAGGGTTTATTTAGTGGTAGTAATA**ATG**CCT

LigMV sgRNA-β-c putative promoter region

>LigMV
UUGCUAACGGUGGUUCUUACGCGGACGGUUCUAAAAGAAUAAAUUACAAUAGAAAUAACGAUCUUUCUUACGGUUACAAGGGUUUAUUUAGUGGUAGUAAUA**AUG**CCU
>LRSV_mild
UUGCCAACGGUGGUUCUUAUCAAGACGGUAAUAAAAGAAUAAAUUAUAACAAAAAUAAUAAUUUAGCUUACGGUUAUAAAGGAUUAUCUA**AUG**CCU
>LRSV_SN
UCGCUAACGGAGGUUUCUAUCAAGACGGUAAUAAGAGGAUAAAUUAUAAUAAAAAUAAUAAUUUAGCCUACGGUUAUAGAGGUUUAUCUA**AUG**CCU
>PSLV_Canada
UUGCCAACGGUGGGAGUUAUCGAGACGGUUCUAAGUGUAUUUCUUACAAUCGAAACAACCCUUUCGCCU**AUG**GCA
>BSMV_China
UCGCUAACGGAGGUAGUUACAGAGACGGGUCAAAAAGUAUAAGUUAUAAUCGCAAUCAUCCUUUUGCCU**AUG**GCA
>BSMV_ND18
UUCGCCAACGGAGGUAGUUACAGGGACGGGUCAAAGAGUAUAAGUUAUAAUCGUAAUCAUCCUUUUGCCU**AUG**GCA

Supplementary Dataset S1C. Nucleotide sequences of 1.9 Kb gRNA-γ region RT-PCR amplified from 10 LigMV isolates and their multiple alignment comparison with complete and near complete genome sequences of Swiss (CH), French (FR) and Austrian (AUT) isolates obtained by Illumina sequencing (sRNA-seq, dsRNA-seq and VANA-seq)

In the CH sRNA isolate, UNDERLINED NUCLEOTIDES in 5'- and 3'-regions were confirmed by respectively 5'-RACE and RT-PCR with 3-CR and segment-specific primers, followed by 3'-RACE (the lowercase nucleotides are not supported or deviating from sRNA contigs). The **lowercase bold** nucleotides in the poly(A) tract and upstream sequences - are those missing in the sRNA contigs. The three **bold** nucleotides in the 3'-CR are the SNP positions distinguishing each gRNA. In **BOLD RED** are the start and stop codons of viral ORFs and transcription start site of sgRNA. In cyan are positions of SNPs and indels (underlined) in the Austrian isolate, in green those in the French isolate and in yellow those in both French and Austrian isolates. In grey, SNPs and indels in the Swiss isolate assembled from Illumina dsRNA reads (**in yellow bold** - identical SNPs shared with French and Austrian isolates).

>LigMV_gamma_CH_sRNA
tccttAGcggaTTCcGaGCTTCTTTGAAGGTCTGTGGGAGTTTAGATTTACCAACAAACATTATCAATCTTTGATATTTTTCAATG**ATG**GCTTTGCCTAGCTTCGCGCTCGCAAAGCCTAATCCCAAACTGTGTTTTAAGAAACTTCAAGTATTAGCAT
TCGTTGCGTTACTTGTGAGGTCGAAAAAAACGTTTATTCTCTCTGTCTTAAAAAGATGGTTTGGGATTTTCTTCCTAAGGAAGAATTGGTTCGCATGTTTGAAAGAGAAACTTATCTTTTCGGGTAAGGCTGTTTACTGCACGGTAACGGCTAGATGTCC
CTTAAAGTTTGACGAGGTTCGAGAAAAGGGTGTTTACAGAGATATCTTTTCGGAAAATGATGTGGTTGATGTGATGGAAATGAAGTTACAAAAACTTTTACATCATGAAGGTGACAAATGGAGGAGAAAAATTGTCTTTGATGTTCCGCGTATGTCTTCT
AAGAGAGTTCCGGTTCCTAGAATTGGTACCGTAAAGTGCATTCAAAGTTGGTTTGATGCTACTTTTCCTGGTAACTCAATCAGGTTCTCAGATTTTGATGGTTACACTGTTGCAATATCGGATCTTGCCGTTGATGTGACAGATTGTAAAATAAAAATTG
GAAAAACTTTTAAGCCGTACGTTTTTAAAAGCGCTCTGCGCCCTGAATTAAGGACATCAATGCCTGAAAAGAGACAAGGTAGTTTAGCCGAAAGTGTGTTGGCTTTGAGAAAAAGAAACTTAGCAGC**A**CCCAGATTGCAAGGGGCTGTTAACGAATGGCA
GGTGATTGAAAA**T**ACTATGAAGAGGGCTTTGGATATTTTCTTTATTAAGGATTTGATTGATAGGTCGCACCATGCTTCCTATGAATCAGCCTTAAAATGGTGGGATAAGCAGTCAACTACTGCCCGTGCTCAACTTGCAGC**T**GAGACGAGACGACTTTGT
GACATCGACTTCTGTACTTACAATTTTATGAT**T**AAAAATGATGTAAAACCGAAGTTGGATCTGACACCCCAAGTTGAGTATGCAGCTTTGCAAACGGTAGTATTTCCTGATAAAATCGTGAATGCTTTTTTCGGCCCTGTTATAAAGGAAATTAATGAGA
GGATCATTAGAGCCTTGAAACCTCATATTGTGTTTAATACACGTATGACGGCCGAAGGTTTAAATGATACTATTTCATTTCTGCCCCCCAAGTCTTACAGGTCACTTGAAATCGATTTCTCAAAATTTGACAAGTCAAAGACTGGTCTGCATATTAAATG
TGTAATAGCTTTATACAAATTGTTTGGACTGGATGGAATCCTAGAGGTGCTCTGGGAGAAATCTCAAGTACAAACATACGTAAAGGACCGAAATTTTGGTCTTGAAGCTTACCTTTTATTTCAGCAGAAATCAGGAAATTGTGACACTTACGGTTCTAAT
ACTTGGTCTGCAGCATTAGCGTTGCTAGATTGTTTGCCTTTGGAGCATTCAGTCTATGCGGTTTTTGGTGGAGACGATTCTTTGATACTATTTGAGGAGGGGTATCTTGTTAATGACCCATGCAAGCGTTTGGCTGGAACGTGGAATTTTGAATGCAAAA
TTTTTGCTTTCAATTTTCCTTCCTTTTGCGGGAAGTTTGTTGTCAGTGTTGACGACAAATATGTTTTTGTACCAGATGCTGCTAAGCTAATCACAAAACTGGGGAGAACCGACGTAAAGGATGTAGAAGCTTTAAGTGAAATTTACATTTCAATTAATGA
CAATTACAGATCCTTTAAGGATTTCCGTGTTTTGGATGAGTTGAGCGATGCTTTGGTTGATAGATATCATGC**T**CCTTACGACGCTTTATCTGCTTTGGTGTCTCTGTGTTACTATATATTCGATTTCGACAACTTTAAGCAGCTGTTTGGTTGTGATGGC
AATTTTGTGGATAAAATAATTGGAAAGGATTTCGATTGG**TGA**ATCCTTGATGTATTT**T**TCTAACATTTTTGCTTTTTACCAA**ATG**GCAGCCAACCCAAGAGTTTGTGAATTATGTAACATTGTTTTTGACAAGACTGATACGACTTTCTGTTCTCCTAAA
TGCGAGAAGCGTGCTAGTTTGAAGAAAGAGAGACTTCGTCGTGCTGAGCTGTTTATCAAGCACGGGCTTACAGGAAAACCTTGTGGTTTAAACAAGTATCCCGGTGAATCATGTGGTATGTATGCCTATATTGCAGAAAACCAGTTACCAGATGGCAATA
CTACCCTTACGATTTCCGAATGTTGCGGGCTTAACCACGCTGCGTATTGCTGTACGCTTGATGCTTTTGATATGAGCACACT**A**GAAACGAGGGC**a**CTCATGAAGAAGCTGGAGAGGTATCGTGATGCCTCTATGGAAAAAATGAAAGTTATAACTAACCA
GTTAAAAGCGATGAAACTAAATTCTAAGCTT**TAA**TAAGTTTTTCTTTTCTTTAAAAAAAAAAAAA**aaaaaaaaaaaaaaaaaaaaaaaaaaaaaaaa**AAAAAAAAAAAAAA**t**AATGCCTGCTATTAAGACGGTGAAACCTATAAACGGGTTCAACGGGTG
AGATAGTGGGAATTGGTGTGTAAGCGCGTACTCCTACG**T**CGCTGCATCAACAATGATCTGTGGATCGATTGTTGCACACTGAGCCG**G**AGCTCAGCTTTGGTCCTTTAGGAGACCA

>LigMV_FR_VANA
AATGAAGGATGTGGTTGATGTGATGTAAAAGTTTTTGTAACTTCATTTCCATCACATCAACCACATCATTTTCCGAAAAGATCTTTCTGCGCTTCTTTGAAGGTCTGTGGGAGTTTAGATTTACCAACAAACATTATCAATCTTTGATATTTTTCAACG
ATGGCTTTGCCTAGCTTCGCGCTCGCAAAGCCTAATCCCAAACTGTGTTTTAAGAAACTTCAAGTATTAGCATTCGTTGCGTTACTTGTGAGGTCGAAAAAAACGTTTATTCTCTCTGTCTTAAAAAGATGGTTTGGGATTTTCTTCCTAAGGAAGAATT
GGTTCGCATGTTTGAAAGAGAAACTTATCTTTTCGGGTAAGGCTGTTTACTGCACGGTAACGGCTAGATGTCCCTTAAAGTTTGACGAGGTTCGAGAAAAAGGTGTTTACAGAGATATCTTTTCGGAAAATGATGTGGTTGATGTGATGGAAATGAAGTT
ACAAAAACTTTTACATCATGAAGGTGACAAATGGAGGAGAAAAATTGTCTTTGATGTTCCGCGTATGTCTTCTAAGAGAGTTCCGGTTCCTAGAATTGGTACCGTAAAGTGCATTCAAAGTTGGTTTGATGCTACTTTTCCTGGTAACTCAATCAGGTTC
TCAGATTTTGATGGTTACACTGTTGCAATATCGGATCTTGCCGTTGATGTGACAGATTGTAAAATAAAAATTGGAAAAACTTTTAAGCCGTACGTTTTTAAAAGCGCTCTGCGTCCTGAATTAAGGACATCAATGCCTGAAAAGAGACAAGGTAGTTTAG
CCGAAAGTGTGTTGGCTTTGAGAAAAAGAAACTTAGCAGCGCCCAGATTGCAAGGGGCTGTTAACGAATGGCAGGTGATTGAAAACACTATGAAGAGGGCTTTGGATATTTTCTTTATTAAGGATTTGATTGATAGGTCGCACCATGCTTCCTATGAATC
AGCCTTAAAATGGTGGGATAAGCAGTCAACTACTGCCCGTGCTCAACTTGCAGCAGAGACGAGACGACTTTGTGACATCGACTTCTGTACTTACAATTTTATGATCAAAAATGATGTAAAACCGAAGTTGGATCTGACACCCCAAGTTGAGTATGCAGCT
TTGCAAACGGTAGTATTTCCTGATAAAATCGTGAATGCTTTTTTCGGCCCTGTTATAAAGGAAATTAATGAGAGGATCATTAGAGCCTTGAAACCTCATATTGTGTTTAATACACGTATGACGGCCGAAGGTTTAAATGATACTATTTCATTTCTGCCCC
CCAAGTCTTACAGGTCACTTGAAATCGATTTCTCAAAATTTGACAAGTCAAAGACTGGTCTGCATATTAAATGTGTAATAGCTTTATACAAATTGTTTGGACTGGATGGAATCCTAGAGGTGCTCTGGGAGAAATCTCAAGTACAAACATACGTAAAGGA
CCGAAATTTTGGTCTTGAAGCTTACCTTTTATTTCAGCAGAAATCAGGAAATTGTGACACTTACGGTTCTAATACTTGGTCTGCAGCATTAGCGTTGCTAGATTGTTTGCCTTTGGAGCATTCAGTCTATGCGGTTTTTGGTGGAGACGATTCTTTGATA

CTATTTGAGGAGGGGTATCTTGTTAATGACCCATGCAAGCGTTTGGCTGGAACGTGGAATTTTGAATGCAAAATTTTTGCTTTCAATTTTCCTTCCTTTTGCGGGAAGTTTGTTGTCAGTGTTGACGACAAATATGTTTTTGTACCAGATGCTGCTAAGC
TAATCACAAAACTGGGGAGAACCGATGTAAAGGATGTAGAAGCTTTAAGTGAAATTTACATTTCAATTAATGACAATTACAGATCCTTTAAGGATTTCCGTGTTTTGGATGAGTTGAGCGATGCTTTGGTTGATAGATATCATGCCCCTTACGACGCTTT
ATCTGCTTTGGTGTCTCTGTGTTACTATATATTCGATTTCGACAACTTTAAGCAGCTGTTTGGTTGTGATGGCAATTTTGTGGATAAAATAATTGGAAAGGATTTCGATTGGTGAATCCTTGATGTATTTTTCTAACACTTTTGCTTTTTTACCAAATGG
CAGCCAACCCGAGAGTTTGTGAATTATGTAACATTGTTTTTGACAAGACTGATACGACTTTCTGTTCTCCTAAATGCGAGAAGCGTGCTAGTTTGAAGAAAGAGAGACTTCGTCGTGCTGAGCTGTTTATCAAGCACGGGCTTACAGGAAAACCTTGTGG
TTTAAACAAGTATCCCGGTGAATCATGTGGTATGTATGCCTATATTGCAGAAAACCAGTTACCAGATGGCAATACTACCCTTACGATTTCCGAATGTTGCGGGCTTAACCACGCTGCGTATTGCTGTACGCTTGATGCTTTTGATATGAGCACACTGGAA
ACGAGGGCTCTCATGAAGAAGCTGGAGAGGTATCGTGATGCCTCTATGGAAAAAATGAAAGTTATAACTAACCAGTTAAAAGCGATGAAACTAAATTCTAAGCTTTAATAAGTTTTTCTTTTCTTTTTAAAAAAAAAAAAAAAAAAAAAAAACCCAAACCC
CCGCGGTTTTTG

>LigMV_AUT_VANA
CTACACGACGCTCTTCCGATCTAAGGTAGAAGCGTGGAGACTCTGGTCTGTGGGAGTTTAGATTTTACCAACAAACATTATCAATCTTTGATATTTTTCAATGATGGCTTTGCCTAGCTTCGCGCTCGCAAAGCCTAATCCCAAACTGTGTTTTAAGAAA
CTTCAAGTATTAGCATTCGTTGCGTTACTTGTGAGGTCGAAAAAAACGTTTATTCTCTCTGTCTTAAAAAGATGGTTTGGGATTTTCTTCCTAAGGAAGAATTGGTTCGCATGTTTGAAAGAGAAACTTATCTTTTCGGGTAAGGCTGTTTACTGCACGG
TAACGGCTAGATGTCCCTTAAAGTTTGACGAGGTTCGAGAAAAAGGTGTTTACAGAGATATCTTTTCGGAAAATGATGTGGTTGATGTGATGGAAATGAAGTTACAAAAACTTTTACATCATGAAGGTGACAAATGGAGGAGAAAAATTGTCTTTGATGT
TCCGCGTATGTCTTCTAAGAGAGTTCCGGTTCCTAGAATTGGTACCGTAAAGTGCATTCAAAGTTGGTTTGATGCTACTTTTCCTGGTAACTCAATCAGGTTCTCAGATTTTGATGGTTACACTGTTGCAACATCGGATCTTGCCGTTGATGTGACAGAT
TGTAAAATAAAAATTGGAAAAACTTTTAAGCCGTACGTTTTTAAAAGCGCTCTGCGCCCTGAATTAAGGACATCAATGCCTGAAAAGAGACAAGGTAGTTTAGCCGAAAGTGTGTTGGCTTTGAGAAAAAGAAACTTAGCAGCGCCCAGATTGCAAGGGG
CTGTTAACGAATGGCAGGTGATTGAAAACACTATGAAGAGGGCTTTGGATATTTTCTTTAATAAGGATTTGATTGATAGGTCGCACCATGCTTCCTATGAATCAGCCTTAAAATGGTGGGATAAGCAGTCAACTACTGCCCGTGCTCAACTTGCAGCAGA
GACGAGACGACTTTGTGACATCGACTTCTGTACTTACAATTTTATGATCAAAAATGATGTAAAACCGAAGTTGGATCTGACACCCCAAGTTGAGTATGCAGCTTTGCAAACGGTAGTATTTCCTGATAAAATCGTGAATGCTTTTTTCGGCCCTGTTATA
AAGGAAATTAATGAGAGGATCATTAGAGCCTTGAAACCTCATATTGTGTTTAATACACGTATGACGGCCGAAGGTTTAAATGATACTATTTCATTTCTGCCCCCCAAGTCTTACAGGTCACTTGAAATCGATTTCTCAAAATTTGACAAGTCAAAGACTG
GTCTGCATATTAAATGTGTAATAGCTTTATACAAATTGTTTGGACTGGATGGAATCCTAGAGGTGCTCTGGGAGAAATCTCAAGTACAAACATACGTAAAGGACCGAAATTTTGGTCTTGAAGCTTACCTTTTATTTCAGCAGAAATCAGGAAATTGTGA
CACTTACGGTTCTAATACTTGGTCTGCAGCATTAGCGTTGCTAGATTGTTTGCCTTTGGAGCATTCAGTCTATGCGGTTTTTGGTGGAGACGATTCTTTGATACTATTTGAGGAGGGGTATCTTGTTAATGACCCATGCAAGCGTTTGGCTGGAACGTGG
AATTTTGAATGCAAAATTTTTGCTTTCAATTTTCCTTCCTTTTGCGGGAAGTTTGTTGTCAGTGTTGACGACAAATATGTTTTTGTACCAGATGCTGCTAAGCTAATCACAAAACTGGGGAGAACCGACGTAAAGGATGTAGAAGCTTTAAGTGAAATTT
ACATTTCAATTAATGACAATTACAGATCCTTTAAGGATTTCCGTGTTTTGGATGAGTTGAGCGATGCTTTGGTTGATAGATATCATGCCCCTTACGACGCTTTATCTGCTTTGGTGTCTCTGTGTTACTATATATTCGATTTCGACAACTTTAAGCAGCT
GTTTGGTTGTGATGGCAATTTTGTGGATAAAATAATTGGAAAGGATTTCGATTGGTGAATCCTTGATGTATTTTTCTAACATTTTTGCTTTTTACCAAATGGCAGCCAACCCAAGAGTTTGTGAATTATGTAACATTGTTTTTGACAAGACTGATACGAC
TTTCTGTTCTCCTAAATGCGAGAAGCGTGCTAGTTTGAAGAAAGAGAGACTTCGTCGTGCTGAGCTGTTTATCAAGCACGGGCTTACAGGAAAACCTTGTGGTTTAAACAAGTATCCCGGTGAATCATGTGGTATGTATGCCTATATTGCAGAAAACCAG
TTACCAGATGGCAATACTACCCTTACGATTTCCGAATGTTGCGGGCTTAACCACGCTGCGTATTGCTGTACGCTTGATGCTTTTGATATGAGCACACTGGAAACGAGGGCTCTCATGAAGAAGCTGGAGAGGTATCGTGATGCCTCTATGGAAAAAATGA
AAGTTATAACTAACCAGTTAAAAGCGATGAAACTAAATTCTAAGCTTTAATAAGTTTTTCTTTTCTTTTTAAAAAAAAAAAAAAAAAAAAAAAAAAAAAAAAAAAAAAAAAAAAAAAAGATGTTTTAATAAGAGAAAAAAATAAAAAAGATAAAAAAGAA
GAAATAAATTTTAT

>LigMV_CH_dsRNA
ctggagttcagacgtgtgctcttccgatctatcagagccttCGGATCCTCCTCGCGCTTCTCTGAAGGTCTGTGGGAGTTTAGATTTTACCAACAAACATTATCAATCTTTGATATTTTTCAATGATGGCTTTGCCTAGCTTCGCGCTCGCAAAGCCTAA
TCCCAAACTGTGTTTTAAGAAACTTCAAGTATTAGCATTCGTTGCGTTACTTGTGAGGTCGAAAAAAACGTTTATTCTCTCTGTCTTAAAAAGATGGTTTGGGATTCTCTTCCTAAGGAAGAATTGGTTCGCATGTTTGAAAGAGAAACTTATCTTTTCG
GGTAAGGCTGTTTACTGCACGGTAACGGCTAGATGTCCCTTAAAGTTTGACGAGGTTCGAGAAAAGGGTGTTTACAGAGATATCTTTTCGGAAAATGATGTGGTTGATGTGATGGAAATGAAGTTACAAAAACTTTTACATCATGAAGGTGACAAATGGA
GGAGAAAAATTGTCTTTGATGTTCCGCGTATGTCTTCTAAGAGAGTTCCGGTTCCTAGAATTGGTACCGTAAAGTGCATTCAAAGTTGGTTTGATGCTACTTTTCCTGGTAACTCAATCAGGTTCTCAGATTTTGATGGTTACACTGTTGCAATATCGGA
TCTTGCCGTTGATGTGACAGATTGTAAAATAAAAATTGGAAAAACTTTTAAGCCGTACGTTTTTAAAAGCGCTCTGCGCCCTGAATTAAGGACATCAATGCCTGAAAAGAGACAAGGTAGTTTAGCCGAAAGTGTGTTGGCTTTGAGAAAAAGAAACTTA
GCAGCGCCCAGATTGCAAGGGGCTGTTAACGAATGGCAGGTGATTGAAAACACTATGAAGAGGGCTTTGGATATTTTCTTTATTAAGGATTTGATTGATAGGTCGCACCATGCTTCCTATGAATCAGCCTTAAAATGGTGGGATAAGCAGTCAACTACTG
CCCGTGCTCAACTTGCAGCAGAGACGAGACGACTTTGTGACATCGACTTCTGTACTTACAATTTTATGATCAAAAATGATGTAAAACCGAAGTTGGATCTGACACCCCAAGTTGAGTATGCAGCTTTGCAAACGGTAGTATTTCCTGATAAAATCGTGAA
TGCTTTTTTCGGCCCTGTTATAAAGGAAATTAATGAGAGGATCATTAGAGCCTTGAAACCTCATATTGTGTTTAATACACGTATGACGGCCGAAGGTTTAAATGATACTATTTCATTTCTGCCCCCCAAGTCTTACAGGTCACTTGAAATCGATTTCTCA
AAATTTGACAAGTCAAAGACTGGTCTGCATATTAAATGTGTAATAGCTTTATACAAATTGTTTGGACTGGATGGAATCCTAGAGGTGCTCTGGGAGAAATCTCAAGTACAAACATACGTAAAGGACCGAAATTTTGGTCTTGAAGCTTACCTTTTATTTC
AGCAGAAATCAGGAAATTGTGACACTTACGGTTCTAATACTTGGTCTGCAGCATTAGCGTTGCTAGATTGTTTGCCTTTGGAGCATTCAGTCTATGCGGTTTTTGGTGGAGACGATTCTTTGATACTATTTGAGGAGGGGTATCTTGTTAATGACCCATG
CAAGCGTTTGGCTGGAACGTGGAATTTTGAATGCAAAATTTTTGCTTTCAATTTTCCTTCCTTTTGCGGGAAGTTTGTTGTCAGTGTTGACGACAAATATGTTTTTGTACCAGATGCTGCTAAGCTAATCACAAAACTGGGGAGAACCGACGTAAAGGAT
GTAGAAGCTTTAAGTGAAATTTACATTTCAATTAATGACAATTACAGATCCTTTAAGGATTTCCGTGTTTTGGATGAGTTGAGCGATGCTTTGGTTGATAGATATCATGCCCCTTACGACGCTTTATCTGCTTTGGTGTCTCTGTGTTACTATATTTTCG
ATTTCGACAACTTTAAGCAGCTGTTTGGTTGTGATGGCAATTTTGTGGATAAAATAATTGGAAAGGATTTCGATTGGTGAATCCTTGATGTATTTTTCTAACATTTTTGCTTTTTACCAAATGGCAGCCAACCCAAGAGTTTGTGAATTATGTAACATTG
TTTTTGACAAGACTGATACGACTTTCTGTTCTCCTAAATGCGAGAAGCGTGCTAGTTTGAAGAAAGAGAGACTTCGTCGTGCTGAGCTGTTTATCAAGCACGGGCTTACAGGAAAACCTTGTGGTTTAAACAAGTATCCCGGTGAATCATGTGGTATGTA
TGCCTTTATTGCAGAAAACCAGTTACCAGATGGCAATACTACCCTTACGATTTCCGAATGTTGCGGGCTTAACCACGCTGCGTATTGCTGTACGCTTGATGCTTTTGATATGAGCACACTGGAAACGAGGGCTCTCATGAAGAAGCTGGAGAGGTATCGT
GATGCCTCTATGGAAAAAATGAAAGTTATAACTAACCAGTTAAAAGCGATGAAACnnnnn

>L.vulgare_iso-2_CH_Nyon_Gen
TTTGATATTTTTCAATGATGGCTTTGCCTAGCTTCGCGCTCGCAAAGCCTAATCCCAAACTGTGTTTTAAGAAACTTCAAGTATTAGCATTCGTTGCGTTACTTGTGAGGTCGAAAAAAACGTTTATTCTCTCTGTCTTAAAAAGATGGTTTGGGATTTT
CTTCCTAAGGAAGAATTGGTTCGCATGTTTGAAAGAGAAACTTATCTTTTCGGGTAAGGCTGTTTACTGCACGGTAACGGCTAGATGTCCCTTAAAGTTTGACGAGGTTCGAGAAAAAGGTGTTTACAGAGATATCTTTTCGGAAAATGATGTGGTTGAT
GTGATGGAAATGAAGTTACAAAAACTTTTACATCATGAAGGTGACAAATGGAGGAGAAAAATTGTCTTTGATGTTCCGCGTATGTCTTCTAAGAGAGTTCCGGTTCCTAGAATTGGTACCGTAAAGTGCATTCAAAGTTGGTTTGATGCTACTTTTCCTG
GTAACTCAATCAGGTTCTCAGATTTTGATGGTTACACTGTTGCAATATCGGATCTTGCCGTTGATGTGACAGATTGTAAAATAAAATTTGGAAAAACTTTTAAGCCGTACGTTTTTAAAAGCGCTCTGCGCCCTGAATTAAGGACATCAATGCCTGAAAA
GAGACAAGGTAGTTTAGCCGAAAGTGTGTTGGCTTTGAGAAAAAGAAACTTAGCAGCGCCCAGATTGCAAGGGGCTGTTAACGAATGGCAGGTGATTGAAAACACTATGAAGAGGGCTTTGGATATTTTCTTTATTAAGGATTTGATTGATAGGTCGCAC
CATGCTTCCTATGAATCAGCCTTAAAATGGTGGGATAAGCAGTCAACTACTGCCCGTGCTCAACTTGCAGCAGAGACGAGACGACTTTGTGACATCGACTTCTGTACTTACAATTTTATGATCAAAAATGATGTAAAACCGAAGTTGGATCTGACACCCC
AAGTTGAGTATGCAGCTTTGCAAACCGTAGTATTTCCTGATAAAATCGTGAATGCTTTTTTCGGCCCTGTTATAAAGGAAATTAATGAGAGGATCATTAGAGCCTTGAAACCTCATATTGTGTTTAATACACGTATGACGGYCGAAGGTTTAAATGATAC
TATTTCATTTCTGCCCCCCAAGTCTTACAGGTCACTTGAAATCGATTTCTCAAAATTTGACAAGTCAAAGACTGGTCTGCATATTAAATGTGTAATAGCTTTATACAAATTGTTTGGACTGGATGGAATCCTAGAGGTGCTCTGGGAGAAATCTCAAGTA
CAAACATACGTAAAGGACCGAAATTTTGGTCTTGAAGCTTACCTTTTATTTCAGCAGAAATCAGGAAATTGTGACACTTACGGTTCTAATACTTGGTCTGCAGCATTAGCGTTGCTAGATTGTTTGCCTTTGGAGCATTCAGTCTATGCGGTTTTTGGTG
GAGACGATTCTTTGATACTATTTGAGGAGGGGTATCTTGTTAATGACCCATGCAAGCGTTTGGCTGGAACGTGGAATTTTGAATGCAAAATTTTTGCTTTCAATTTTCCTTCCTTTTGCGGGAAGTTTGTTGTCAGTGTTGACGACAAATATGTTTTTGT
ACCAGATGCTGCTAAGCTAATCACAAAACTGGGGAGAACCGACGTAAAGGATGTAGAAGCTTTAAGTGAAATTTACATTTCAATTAATGACAAYTACAGATCCTTTAAGGATTTCCGTGTTTTGGATGAGTTGAGCGATGCTTTGGTTGATAGATATCAT
GCYCCTTACGACGCTTTATCTGCTTTGGTGTCTCTGTGTTACTATATATTCGATTTCGACAACTTTAAGCAGCTGTTTGGTTGTGATGGCAATTTTGTGGATAAAATAATTGGAAAGGATTTCGATTGGTGAATCCTTGATGTATTTTTCTAACATTTTT
GCTTTTTACCAAATGGCAGCNNCCNNAAANTTT

>C.quinoa_iso-1288_CH_2007
TTTGATATTTTTCNTGATGGCTTTGCCTAGCTTCGCGCTCGCAAAGCCTAATCCCAAACTGTGTTTTAAGAAACTTCAAGTATTAGCATTCGTTGCGTTACTTGTGAGGTCGAAAAAAACGTTTATTCTCTCTGTCTTAAAAAGATGGTTTGGGATTCTC
TTCCTAAGGAAGAATTGGTTCGCATGTTTGAAAGAGAAACTTATCTTTTCGGGTAAGGCTGTTTACTGCACGGTAACGGCTAGATGTCCCTTAAAGTTTGACGAGGTTCGAGAAAAGGGTGTTTACAGAGATATCTTTTCGGAAAATGATGTGGTTGATG
TGATGGAAATGAAGTTACAAAAACTTTTACATCATGAAGGTGACAAATGGAGGAGAAAAATTGTCTTTGATGTTCCGCGTATGTCTTCTAAGAGAGTTCCGGTTCCTAGAATTGGTACCGTAAAGTGCATTCAAAGTTGGTTTGATGCTACTTTTCCTGG
TAACTCAATCAGGTTCTCAGATTTTGATGGTTACACTGTTGCAATATCGGATCTTGCCGTTGATGTGACAGATTGTAAAATAAAAATTGGAAAAACTTTTAAGCCGTACGTTTTTAAAAGCGCTCTGCGCCCTGAATTAAGGACATCAATGCCTGAGAAG
AGACAAGGTAGTTTAGCCGAAAGTGTGTTGGCTTTGAGAAAAAGAAACTTAGCAGCGCCCAGATTGCAAGGGGCTGTTAACGAATGGCAGGTGATTGAAAACACTATGAAGAGGGCTTTGGATATTTTCTTTATTAAGGATTTGATTGATAGGTCGCACC
ATGCTTCCTATGAATCAGCCTTAAAATGGTGGGATAAGCAGTCAACTACTGCCCGTGCTCAACTTGCAGCAGAGACGAGACGACTTTGTGACATCGACTTCTGTACTTACAATTTTATGATCAAAAATGATGTAAAACCGAAGTTGGATCTGACACCCCA
AGTTGAGTATGCAGCTTTGCAAACGGTAGTATTTCCTGATAAAATCGTGAATGCTTTTTTCGGCCCTGTTATAAAGGAAATTAATGAGAGGATCATTAGAGCCTTGAAACCTCATATTGTGTTTAATACACGTATGACGGCCGAAGGTTTAAATGATACT
ATTTCATTTCTGCCCCCCAAGTCTTACAGGTCACTTGAAATCGATTTCTCAAAATTTGACAAGTCAAAGACTGGTCTGCATATTAAATGTGTAATAGCTTTATACAAATTGTTTGGACTGGATGGAATCCTAGAGGTGCTCTGGGAGAAATCTCAAGTAC
AAACATACGTAAAGGACCGAAATTTTGGTCTTGAAGCTTACCTTTTATTTCAGCAGAAATCAGGAAATTGTGACACTTACGGTTCTAATACTTGGTCTGCAGCATTAGCGTTGCTAGATTGTTTGCCTTTGGAGCATTCAGTCTATGCGGTTTTTGGTGG
AGACGATTCTTTGATACTATTTGAGGAGGGGTATCTTGTTAATGACCCATGCAAGCGTTTGGCTGGAACGTGGAATTTTGAATGCAAAATTTTTGCTTTCAATTTTCCTTCCTTTTGCGGGAAGTTTGTTGTCAGTGTTGACGACAAATATGTTTTTGTA
CCAGATGCTGCTAAGCTAATCACAAAACTGGGGAGAACCGACGTAAAGGATGTAGAAGCTTTAAGTGAAATTTACATTTCAATTAATGACAATTACAGATCCTTTAAGGATTTCCGTGTTTTGGATGAGTTGAGCGATGCTTTGGTTGATAGATATCATG
CCCCTTACGACGCTTTATCTGCTTTGGTGTCTCTGTGTTACTATATTTTCGATTTCGACAACTTTAAGCAGCTGTTTGGTTGTGATGGCAATTTTGTGGATAAAATAATTGGAAAGGATTTCGATTGGTGAATCCTTGATGTATTTTTCTAACATTTTTG
CTTTTTACCAAATGGCAGCCAACCC

>L.vulgare_iso-1_CH_Nyon_Gla
TTTGATATTTTTCATGATGGCTTTGCCTAGCTTCGCGCTCGCAAAGCCTAATCCCAAACTGTGTTTTAAGAAACTTCAAGTATTAGCATTCGTTGCGTTACTTGTGAGGTCGAAAAAAACGTTTATTCTCTCTGTCTTAAAAAGATGGTTTGGGATTTTC
TTCCTAAGGAAGAATTGGTTCGCATGTTTGAAAGAGAAACTTATCTTTTCGGGTAAGGCTGTTTACTGCACGGTAACGGCTAGATGTCCCTTAAAGTTTGACGAGGTTCGAGAAAAGGGTGTTTACAGAGATATCTTTTCGGAAAATGATGTGGTTGATG
TGATGGAAATGAAGTTACAAAAACTTTTACATCATGAAGGTGACAAATGGAGGAGAAAAATTGTCTTTGATGTTCCGCGTATGTCTTCTAAGAGAGTTCCGGTTCCTAGAATTGGTACCGTAAAGTGCATTCAAAGTTGGTTTGATGCTACTTTTCCTGG
TAACTCAATCAGGTTCTCAGATTTTGATGGTTACACTGTTGCAATATCGGATCTTGCCGTTGATGTGACAGATTGTAAAATAAAAATTGGAAAAACTTTTAAGCCGTACGTTTTTAAAAGCGCTCTGCGCCCTGAATTAAGGACATCAATGCCTGAAAAG
AGACAAGGTAGTTTAGCCGAAAGTGTGTTGGCTTTGAGAAAAAGAAACTTAGCAGCACCCAGATTGCAAGGGGCTGTTRACGAATGGCAGGTGATTGAAAATACTATGAAGAGGGCTTTGGATATTTTCTTTATTAAGGATTTGATTGATAGGTCGCACC
ATGCTTCCTATGAATCAGCCTTAAAATGGTGGGATAAGCAGTCAACTACTGCCCGTGCTCAACTTGCARCTGAGACGAGACGACTTTGTGACATCGACTTCTGTACTTACAATTTTATGATTAAAAATGATGTAAAACCGAAGTTGGATCTGACACCCCA
AGTTGAGTATGCAGCTTTGCAAACGGTAGTATTTCCTGATAAAATCGTGAATGCTTTTTTCGGCCCTGTTATAAAGGAAATTAATGAGAGGATCATTAGAGCCTTGAAACCTCATATTGTGTTTAATACACGTATGACGGCCGAAGGTTTAAATGATACT
ATTTCATTTCTGCCCCCCAAGTCTTACAGGTCACTTGAAATCGATTTCTCAAAATTTGACAAGTCAAAGACTGGTCTGCATATTAAATGTGTAATAGCTTTATACAAATTGTTTGGACTGGATGGAATCCTAGAGGTGCTCTGGGAGAAATCTCAAGTAC
AAACATACGTAAAGGACCGAAATTTTGGTCTTGAAGCTTACCTTTTATTTCAGCAGAAATCAGGAAATTGTGACACTTACGGTTCTAATACTTGGTCTGCAGCATTAGCGTTGCTAGATTGTTTGCCTTTGGAGCATTCAGTCTATGCGGTTTTTGGTGG
AGACGATTCTTTGATACTATTTGAGGAGGGGTATCTTGTTAATGACCCATGCAAGCGTTTGGCTGGAACGTGGAATTTTGAATGCAAAATTTTTGCTTTCAATTTTCCTTCCTTTTGCGGGAAGTTTGTTGTCAGTGTTGACGACAAATATGTTTTTGTA
CCAGATGCTGCTAAGCTAATCACAAAACTGGGGAGAACCGACGTAAAGGATGTAGAAGCTTTAAGTGAAATTTACATTTCAATTAATGACAATTACAGATCCTTTAAGGATTTCCGTGTTTTGGATGAGTTGAGCGATGCTTTGGTTGATAGATATCATG
CTCCTTACGACGCTTTATCTGCTTTGGTGTCTCTGTGTTACTATATATTCGATTTCGACAACTTTAAGCAGCTGTTTGGTTGTGATGGCAATTTTGTGGATAAAATAATTGGAAAGGATTTCGATTGGTGAATCCTTGATGTATTTTTCTAACATTTTTG
CTTTTTACCAAATGGCAGCNNCCNNAAANTTT

>C.quinoa_iso-1340_CH_2007
CTTTGATATTTTTCNTGATGGCTTTGCCTAGCTTCGCGCTCGCAAAGCCTAATCCCAAACTGTGTTTTAAGAAACTTCAAGTATTAGCATTCGTTGCGTTACTTGTGAGGTCGAAAAAAACGTTTATTCTCTCTGTCTTAAAAAGATGGTTTGGGATTCT
CTTCCTAAGGAAGAATTGGTTCGCATGTTTGAAAGAGAAACTTATCTTTTCGGGTAAGGCTGTTTACTGCACGGTAACGGCTAGATGTCCCTTAAAGTTTGACGAGGTTCGAGAAAAGGGTGTTTACAGAGATATCTTTTCGGAAAATGATGTGGTTGAT
GTGATGGAAATGAAGTTACAAAAACTTTTACATCATGAAGGTGACAAATGGAGGAGAAAAATTGTCTTTGATGTTCCGCGTATGTCTTCTAAGAGAGTTCCGGTTCCTAGAATTGGTACCGTAAAGTGCATTCAAAGTTGGTTTGATGCTACTTTTCCTG
GTAACTCAATCAGGTTCTCAGATTTTGATGGTTACACTGTTGCAATATCGGATCTTGCCGTTGATGTGACAGATTGTAAAATAAAAATTGGAAAAACTTTTAAGCCGTACGTTTTTAAAAGCGCTCTGCGCCCTGAATTAAGGACATCAATGCCTGAAAA
GAGACAAGGTAGTTTAGCCGAAAGTGTGTTGGCTTTGAGAAAAAGAAACTTAGCAGCGCCCAGATTGCAAGGGGCTGTTAACGAATGGCAGGTGATTGAAAACACTATGAAGAGGGCTTTGGATATTTTCTTTATTAAGGATTTGATTGATAGGTCGCAC
CATGCTTCCTATGAATCAGCCTTAAAATGGTGGGATAAGCAGTCAACTACTGCCCGTGCTCAACTTGCAGCAGAGACGAGACGACTTTGTGACATCGACTTCTGTACTTACAATTTTATGATCAAAAATGATGTAAAACCGAAGTTGGATCTGACACCCC
AAGTTGAGTATGCAGCTTTGCAAACGGTAGTATTTCCTGATAAAATCGTGAATGCTTTTTTCGGCCCTGTTATAAAGGAAATTAATGAGAGGATCATTAGAGCCTTGAAACCTCATATTGTGTTTAATACACGTATGACGGCCGAAGGTTTAAATGATAC
TATTTCATTTCTGCCCCCCAAGTCTTACAGGTCACTTGAAATCGATTTCTCAAAATTTGACAAGTCAAAGACTGGTCTGCATATTAAATGTGTAATAGCTTTATACAAATTGTTTGGACTGGATGGAATCCTAGAGGTGCTCTGGGAGAAATCTCAAGTA
CAAACATACGTAAAGGACCGAAATTTTGGTCTTGAAGCTTACCTTTTATTTCAGCAGAAATCAGGAAATTGTGACACTTACGGTTCTAATACTTGGTCTGCAGCATTAGCGTTGCTAGATTGTTTGCCTTTGGAGCATTCAGTCTATGCGGTTTTTGGTG
GAGACGATTCTTTGATACTATTTGAGGAGGGGTATCTTGTTAATGACCCATGCAAGCGTTTGGCTGGAACGTGGAATTTTGAATGCAAAATTTTTGCTTTCAATTTTCCTTCCTTTTGCGGGAAGTTTGTTGTCAGTGTTGACGACAAATATGTTTTTGT
ACCAGATGCTGCTAAGCTAATCACAAAACTGGGGAGAACCGACGTAAAGGATGTAGAAGCTTTAAGTGAAATTTACATTTCAATTAATGACAATTACAGATCCTTTAAGGATTTCCGTGTTTTGGATGAGTTGAGCGATGCTTTGGTTGATAGATATCAT
GCCCCTTACGACGCTTTATCTGCTTTGGTGTCTCTGTGTTACTATATTTTCGATTTCGACAACTTTAAGCAGCTGTTTGGTTGTGATGGCAATTTTGTGGATAAAATAATTGGAAAGGATTTCGATTGGTGAATCCTTGATGTATTTTTCTAACA

>L.vulgare_iso-3_CH_Biel
TTGATATTTTTCAATGATGGCTTTGCCTAGCTTCGCGCTCGCAAAGCCTAATCCCAAACTGTGTTTTAAGAAACTTCAAGTATTAGCATTCGTTGCGTTACTTGTGAGGTCGAAAAAAACGTTTATTCTCTCTGTCTTAAAAAGATGGTTTGGGATTTTC
TTCCTAAGGAAGAATTGGTTCGCATGTTTGAAAGAGAAACTTATCTTTTCGGGTAAGGCTGTTTACTGCACGGTAACGGCTAGATGTCCCTTAAAGTTTGACGAGGTTCGAGAAAAGGGGGTTTACAGAGATATCTTTTCGGAAAATGATGTGGTTGATG
TGATGGAAATGAAGTTACAAAAACTTTTACATCATGAAGGTGACAAATGGAGGAGAAAAATTGTCTTTGATGTTCCGCGTATGTCTTCTAAGAGAGTTCCGGTTCCTAGAATTGGTACCGTAAAGTGCATTCAAAGTTGGTTTGATGCTACTTTTCCTGG
TAACTCAATCAGGTTCTCAGATTTTGATGGTTACACTGTTGCAATATCGGATCTTGCCGTTGATGTGACAGATTGTAAAATAAAATTTGGAAAAACTTTTAAGCCGTACGTTTTTAAAAGCGCTTTGCGCCCTGAATTAAGGACATCAATGCCTGAAAAG
AGACAAGGTAGTTTAGCCGAAAGTGTGTTGGCTTTGAGAAAAAGAAACTTAGCAGCGCCCAGATTGCAAGGGGCTGTTAACGAATGGCAGGTGATTGAAAACACTATGAAGAGGGCTTTGGATATTTTCTTTAATAAGGATTTGATTGATAGGTCGCACC
ATGCTTCCTATGAATCAGCCTTAAAGTGGTGGGATAAGCAGTCAACTACTGCCCGTGCTCAACTTGCAGCAGAGACGAGACGACTTTGTGACATCGACTTCTGTACTTACAATTTTATGATCAAAAATGATGTAAAACCGAAGTTGGATCTGACACCCCA
AGTTGAGTATGCAGCTCTGCAAACGGTAGTATTTCCTGATAAAATCGTGAATGCTTTTTTCGGCCCTGTTATAAAGGAAATTAATGAGAGGATCATTAGAGCTTTGAAACCTCATATTGTGTTTAATACACGTATGACGGCCGAAGGTTTAAATGATACT
ATTTCATTTCTGCCCCCCAAGTCTTACAGGTCACTTGAAATCGATTTCTCAAAATTTGACAAGTCAAAGACTGGTCTGCATATTAAATGTGTAATAGCTTTATACAAATTGTTTGGACTGGATGGAATCCTAGAGGTGCTCTGGGAGAAATCTCAAATAC
AAACATACGTAAAGGACCGAAATTTTGGTCTTGAAGCTTACCTTTTATTTCAGCAGAAATCAGGAAATTGTGATACTTACGGTTCTAATACTTGGTCTGCAGCATTAGCGTTGCTAGATTGTTTGCCTTTGGAGCATTCAGTCTATGCGGTTTTTGGTGG
AGACGATTCTTTGATACTATTTGAGGAGGGGTATCTTGTTAATGACCCATGCAAGCGTTTGGCTGGAACGTGGAATTTTGAATGCAAAATTTTTGCTTTCAATTTTCCTTCCTTTTGCGGGAAGTTTGTTGTCAGTGTTGATGACAAATATGTTTTTGTA
CCAGATGCTGCTAAGCTAATCACAAAACTGGGGAGAACCGACGTAAAGGATGTAGAAGCTTTAAGTGAAATTTACATTTCAATTAATGACAATTACAGATCCTTTAAGGATTTCCGTGTTTTGGATGAGTTGAGCGATGCTTTGGTTGATAGATATCATG
CCCCTTACGACGCTTTATCTGCTTTGGTGTCTCTGTGTTACTATATTTTCGATTTCGACAACTTTAAGCAGCTGTTTGGTTGTGATGGCAATTTTGTGGATAAAATAATTGGAAAGGATTTCGATTGGTGAATCCTTGATGTATTTTTCCTAACATTNNN
GCTTTTTACCAAATGAGCANCCAACCNNAGAGTTTGNA

>L.vulgare_iso-4_CH_Biel
TTTGATATTTTTCAATGATGGCTTTGCCTAGCTTCGCGCTCGCAAAGCCTAATCCCAAACTGTGTTTTAAGAAACTTCAAGTATTAGCATTCGTTGCGTTACTTGTGAGGTCGAAAAAAACGTTTATTCTCTCTGTCTTAAAAAGATGGTTTGGGATTTT
CTTCCTAAGGAAGAATTGGTTCGCATGTTTGAAAGAGAAACTTATCTTTTCGGGTAAGGCTGTTTACTGCACGGTAACGGCTAGATGTCCCTTAAAGTTTGACGAGGTTCGAGAAAAAGGGGTTTACAGAGATATCTTTTCGGAAAATGATGTGGTTGAT
GTGATGGAAATGAAGTTACAAAAACTTTTACATCATGAAGGTGACAAATGGAGGAGAAAAATTGTCTTTGATGTTCCGCGTATGTCTTCTAAGAGAGTTCCGGTTCCTAGAATTGGTACCGTAAAGTGCATTCAAAGTTGGTTTGATGCTACTTTTCCTG
GTAACTCAATCAGGTTCTCAGATTTTGATGGTTACACTGTTGCAATATCGGATCTTGCCGTTGATGTGACAGATTGTAAAATAAAATTTGGAAAAACTTTTAAGCCGTACGTTTTCAAAAGCGCTTTGCGCCCTGAATTAAGGACATCAATGCCTGAAAA
GAGACAAGGTAGTTTAGCCGAAAGTGTGTTGGCTTTGAGAAAAAGAAACTTAGCAGCGCCCAGATTGCAAGGGGCTGTTAACGAATGGCAGGTGATTGAAAACACTATGAAGAGGGCTTTGGATATTTTCTTTAATAAGGATTTGATTGATAGGTCGCAC
CATGCTTCCTATGAATCAGCCTTAAAGTGGTGGGATAAGCAGTCAACTACTGCCCGTGCTCAACTTGCAGCAGAGACGAGACGACTTTGTGACATCGACTTCTGTACTTACAATTTTATGATCAAAAATGATGTAAAACCGAAGTTGGATCTGACACCCC
AAGTTGAGTATGCAGCTTTGCAAACGGTAGTATTTCCTGATAAAATCGTGAATGCTTTTTTCGGCCCTGTTATAAAGGAAATTAATGAGAGGATCATTAGAGCTTTGAAACCTCATATTGTGTTTAATACACGTATGACGGCCGAAGGTTTAAATGATAC
TATTTCATTTCTGCCCCCCAAGTCTTACAGGTCACTTGAAATCGATTTCTCAAAATTTGACAAGTCAAAGACTGGTCTGCATATTAAATGTGTAATAGCTTTATACAAATTGTTTGGACTGGATGGAATCTTAGAGGTGCTCTGGGAGAAATCTCAAGTA

```
CAAACATACGTAAAGGACCGAAATTTTGGTCTTGAAGCTTACCTTTTATTTCAGCAGAAATCAGGAAATTGTGATACTTACGGTTCTAATACTTGGTCTGCAGCATTAGCGTTGCTAGATTGTTTGCCTTTGGAGCATTCAGTCTATGCGGTTTTTGGTG
GAGACGATTCTTTGATACTATTTGAGGAGGGGTATCTTGTTAATGACCCATGCAAGCGTTTGGCTGGAACGTGGAATTTTGAATGCAAAATTTTTGCTTTCAATTTTCCTTCCTTTTGCGGGAAGTTTGTTGTCAGTGTTGATGACAAATATGTTTTTGT
ACCAGATGCTGCTAAGCTAATCACAAAACTGGGGAGAACCGACGTAAAGGATGTAGAAGCTTTAAGTGAAATTTACATTTCAATTAATGACAATTACAGATCCTTTAAGGATTTCCGTGTTTTGGATGAGTTGAGCGATGCTTTGGTTGATAGATATCAT
GCCCCTTACGACGCTTTATCTGCTTTGGTGTCTCTGTGTTACTATATTTTCGATTTCGACAACTTTAAGCAGCTGTTTGGTTGTGATGGCAATTTTGTGGATAAAATAATTGGAAAGGATTTCGATTGGTGAATCCTTGATGTATTTTTCC

>L.vulgare_iso-5_CH_Veve
CTTTGATATTTTTCAATGATGGCTTTGCCTAGCTTCGCGCTCGCAAAGCCTAATCCCAAACTGTGTTTTAAGAAACTTCAAGTATTAGCATTCGTTGCGTTACTTGTGAGGTCGAAAAAAACGTTTATTCTCTCTGTCTTAAAAAGATGGTTTGGGATTT
TCTTCCTAAGGAAGAATTGGTTCGCATGTTTGAAAGAGAAACTTATCTTTTCGGGTAAGGCTGTTTACTGCACGGTAACGGCTAGATGTCCCTTAAAGTTTGACGAGGTTCGAGAAAAAGGTGTTTACAGAGATATCTTTTCGGAAAATGATGTGGTTGA
TGTGATGGAAATGAAGTTACAAAAACTTTTACATCATGAAGGTGACAAATGGAGGAGAAAAATTGTCTTTGATGTTCCGCGTATGTCTTCTAAGAGAGTTCCGGTTCCTAGAATTGGTACCGTAAAGTGCATTCAAAGTTGGTTTGATGCTACTTTTCCT
GGTAACTCAATCAGGTTCTCAGATTTTGATGGTTACACTGTTGCAATATCGGATCTTGCCGTTGATGTGACAGATTGTAAAATAAAAATTGGAAAAACTTTTAAGCCGTACGTTTTTAAAAGCGCTCTGCGCCCTGAATTAAGGACATCAATGCCTGAAA
AGAGACAAGGTAGTTTAGCCGAAAGTGTGTTGGCTTTGAGAAAAAGAAACTTAGCAGCGCCCAGATTGCAAGGGGCTGTTAACGAATGGCAGGTGATTGAAAACACTATGAAGAGGGCTTTGGATATTTTCTTTATTAAGGATTTGATTGATAGGTCGCA
CCATGCTTCCTATGAATCAGCCTTAAAATGGTGGGATAAGCAGTCAACTACTGCCCGTGCTCAACTTGCAGCAGAGACGAGACGACTTTGTGACATCGACTTCTGTACTTACAATTTTATGATCAAAAATGATGTAAAACCGAAGTTGGATCTGACACCC
CAAGTTGAGTATGCAGCTTTGCAAACGGTAGTATTTCCTGATAAAATCGTGAATGCTTTTTTCGGCCCTGTTATAAAGGAAATTAATGAGAGGATCATTAGAGCCTTGAAACCTCATATTGTGTTTAATACACGTATGACGGCCGAAGGTTTAAATGATA
CTATTTCATTTCTGCCCCCCAAGTCTTACAGGTCACTTGAAATCGATTTCTCAAAATTTGACAAGTCAAAGACTGGTCTGCATATTAAATGTGTAATAGCTTTATACAAATTGTTTGGACTGGATGGAATCCTAGAGGTGCTCTGGGAGAAATCTCAAGT
ACAAACATACGTAAAGGACCGAAATTTTGGTCTTGAAGCTTACCTTTTATTTCAGCAGAAATCAGGAAATTGTGACACTTACGGTTCTAATACTTGGTCTGCAGCATTAGCGTTGCTAGATTGTTTGCCTTTGGAGCATTCAGTCTATGCGGTTTTTGGT
GGAGACGATTCTTTGATACTATTTGAGGAGGGGTATCTTGTTAATGACCCATGCAAGCGTTTGGCTGGAACGTGGAATTTTGAATGCAAAATTTTTGCTTTCAATTTTCCTTCCTTTTGCGGGAAGTTTGTTGTCAGTGTTGACGACAAATATGTTTTTG
TACCAGATGCTGCTAAGCTAATCACAAAACTGGGGAGAACCGACGTAAAGGATGTAGAAGCTTTAAGTGAAATTTACATTTCAATTAATGACAATTACAGATCCTTTAAGGATTTCCGTGTTTTGGATGAGTTAAGCGATGCTTTGGTTGATAGATATCA
TGCCCCTTACGACGCTTTATCTGCTTTGGTGTCTCTGTGTTACTATATATTCGATTTCGACAACTTTAAGCAGCTGTTTGGTTGTGATGGCAATTTTGTGGATAAAATAATTGGAAAGGATTTCGATTGGTGAATCCTTGATGTA

>L.vulgare_iso-6_AUT_Inns
CTTTGATATTTTTCNTGATGGCTTTGCCTAGCTTCGCGCTCGCAAAGCCTAATCCCAAACTGTGTTTTAAGAAACTTCAAGTATTAGCATTCGTTGCGTTACTTGTGAGGTCGAAAAAAACGTTTATTCTCTCTGTCTTAAAAAGATGGTTTGGGATTTT
CTTCCTAAGGAAGAATTGGTTCGCATGTTTGAAAGAGAAACTTATCTTTTCGGGTAAGGCTGTTTACTGCACGGTAACGGCTAGATGTCCCTTAAAGTTTGACGAGGTTCGAGAAAAAGGTGTTTACAGAGATATCTTTTCGGAAAATGATGTGGTTGAT
GTGATGGAAATGAAGTTACAAAAACTTTTACATCATGAAGGTGACAAATGGAGGAGAAAAATTGTCTTTGATGTTCCGCGTATGTCTTCTAAGAGAGTTCCGGTTCCTAGAATTGGTACCGTAAAGTGCATTCAAAGTTGGTTTGATGCTACTTTTCCTG
GTAACTCAATCAGGTTCTCAGATTTTGATGGTTACACTGTTGCAATATCGGATCTTGCCGTTGATGTGACAGATTGTAAAATAAAAATTGGAAAAACTTTTAAGCCGTACGTTTTTAAAAGCGCTCTGCGCCCTGAATTAAGGACATCAATGCCTGAAAA
GAGACAAGGTAGTTTAGCCGAAAGTGTGTTGGCTTTGAGAAAAAGAAACTTAGCAGCGCCCAGATTGCAAGGGGCTGTTAACGAATGGCAGGTGATTGAAAACACTATGAAGAGGGCTTTGGATATTTTCTTTAATAAGGATCTGATTGATAGGTCGCAC
CATGCTTCCTATGAATCAGCCTTAAAATGGTGGGATAAGCAGTCAACTACTGCCCGTGCTCAACTTGCAGCAGAGACGAGACGACTTTGTGACATCGACTTCTGTACTTACAATTTTATGATCAAAAATGATGTAAAACCGAAGTTGGATCTGACACCCC
AAGTTGAGTATGCAGCTTTGCAAACGGTAGTATTTCCTGATAAAATCGTGAATGCTTTTTTCGGCCCTGTTATAAAGGAAATTAATGAGAGGATCATTAGAGCCTTGAAACCTCATATTGTGTTTAATACACGTATGACGGCCGAAGGTTTAAATGATAC
TATTTCATTTCTGCCCCCCAAGTCTTACAGGTCACTTGAAATCGATTTCTCAAAATTTGACAAGTCAAAGACTGGTCTGCATATTAAATGTGTAATAGCTTTATACAAACTGTTTGGACTGGATGGAATCCTAGAGGTGCTCTGGGAGAAATCTCAAGTA
CAAACATACGTAAAGGACCGAAATTTTGGTCTTGAAGCTTACCTTTTATTTCAGCAGAAATCAGGAAATTGTGACACTTACGGTTCTAATACTTGGTCTGCAGCATTAGCGTTGCTAGATTGTTTGCCTTTGGAGCATTCAGTCTATGCGGTTTTTGGTG
GAGACGATTCTTTGATACTATTTGAGGAGGGGTATCTTGTTAATGACCCATGCAAGCGTTTGGCTGGAACGTGGAATTTTGAATGCAAAATTTTTGCTTTCAATTTTCCTTCCTTTTGCGGGAAGTTTGTTGTCAGTGTTGACGACAAATATGTTTTTGT
ACCAGATGCTGCTAAGCTAATCACAAAACTGGGGAGAACCGACGTAAAGGATGTAGAAGCTTTAAGTGAAATTTACATTTCAATTAATGACAATTACAGATCCTTTAAGGATTTCCGTGTTTTGGATGAGTTGAGCGATGCTTTGGTTGATAGATATCAT
GCCCCTTACGACGCTTTATCTGCTTTGGTGTCTCTGTGTTACTATATATTCGATTTCGACAACTTTAAGCAGCTGTTTGGTTGTGATGGCAATTTTGTGGATAAAATAATTGGAAAGGATTTCGATTGGTGAATCCTTGATGTATTTTTCTAACATTTTT
GCTTTTTACCAAATGGCAGCCAAC

>L.vulgare_iso-7_CH_Nyon_Gen
TTTGATATTTTTCAATGATGGCTTTGCCTAGCTTCGCGCTCGCAAAGCCTAATCCCAAACTGTGTTTTAAGAAACTTCAAGTATTAGCATTCGTTGCGTTACTTGTGAGGTCGAAAAAAACGTTTATTCTCTCTGTCTTAAAAAGATGGTTTGGGATTCT
CTTCCTAAGGAAGAATTGGTTCGCATGTTTGAAAGAGAAACTTATCTTTTCGGGTAAGGCTGTTTACTGCACGGTAACGGCTAGATGTCCCTTAAAGTTTGACGAGGTTCGAGAAAAGGGTGTTTACAGAGATATCTTTTCGGAAAATGATGTGGTTGAT
GTGATGGAAATGAAGTTACAAAAACTTTTACATCATGAAGGTGACAAATGGAGGAGAAAAATTGTCTTTGATGTTCCGCGTATGTCTTCTAAGAGAGTTCCGGTTCCTAGAATTGGTACCGTAAAGTGCATTCAAAGTTGGTTTGATGCTACTTTTCCTG
GTAACTCAATCAGGTTCTCAGATTTTGATGGTTACACTGTTGCAATATCGGATCTTGCCGTTGATGTGACAGATTGTAAAATAAAAATTGGAAAAACTTTTAAGCCGTACGTTTTTAAAAGCGCTCTGCGCCCTGAATTGAGGACATCAATGCCTGAGAA
GAGACAAGGTAGTTTAGCCGAAAGTGTGTTGGCTTTGAGAAAAAGAAACTTAGCAGCGCCCAGATTGCAAGGGGCTGTTAACGAATGGCAGGTGATTGAAAACACTATGAAGAGGGCTTTGGATATTTTCTTTATTAAGGATTTGATTGATAGGTCGCAC
CATGCTTCCTATGAATCAGCCTTAAAATGGTGGGATAAGCAGTCAACTACTGCCCGTGCTCAACTTGCAGCAGAGACGAGACGACTTTGTGACATCGACTTCTGTACTTACAATTTTATGATCAAAAATGATGTAAAACCGAAGTTGGATCTGACACCCC
AAGTTGAGTATGCAGCTTTGCAAACGGTAGTATTTCCTGATAAAATCGTGAATGCTTTTTTCGGCCCTGTTATAAAGGAAATTAATGAGAGGATCATTAGAGCCTTGAAACCTCATATTGTGTTTAATACACGTATGACGGCCGAAGGTTTAAATGATAC
TATTTCATTTCTGCCCCCCAAGTCTTACAGGTCACTTGAAATCGATTTCTCAAAATTTGACAAGTCAAAGACTGGTCTGCATATTAAATGTGTAATAGCTTTATACAAATTGTTTGGACTGGATGGAATCCTAGAGGTGCTCTGGGAGAAATCTCAAGTA
CAAACATACGTAAAGGACCGAAATTTTGGTCTTGAAGCTTACCTTTTATTTCAGCAGAAATCAGGAAATTGTGACACTTACGGTTCTAATACTTGGTCTGCAGCATTAGCGTTGCTAGATTGTTTGCCTTTGGAGCATTCAGTCTATGCGGTTTTTGGTG
GAGACGATTCTTTGATACTATTTGAGGAGGGGTATCTTGTTAATGACCCATGCAAGCGTTTGGCTGGAACGTGGAATTTTGAATGCAAAATTTTTGCTTTCAATTTTCCTTCCTTTTGCGGGAAGTTTGTTGTCAGTGTTGACGACAAATATGTTTTTGT
ACCAGATGCTGCTAAGCTAATCACAAAACTGGGGAGAACCGACGTAAAGGATGTAGAAGCTTTAAGTGAAATTTACATTTCAATTAATGACAATTACAGATCCTTTAAGGATTTCCGTGTTTTGGATGAGTTGAGCGATGCTTTGGTTGATAGATATCAT
GCCCCTTACGACGCTTTATCTGCTTTGGTGTCTCTGTGTTACTATATTTTCGATTTCGACAACTTTAAGCAGCTGTTTGGTTGTGATGGCAATTTTGTGGATAAAATAATTGGAAAGGATTTCGATTGNNAATCCTTGATGTATT

>L.vulgare_iso-8_CH_Nyon_Pra
TTTGNTATTTTTCAATGATGGCTTTGCCTAGCTTCGCGCTCGCAAGGCCTAATCCCAAACTGTGTTTTAAGAAACTTCAAGTATTAGCATTCGTTGCGTTACTTGTGAGGTCGAAAAAAACGTTTATTCTCTCTGTCTTAAAAAGATGGTTTGGGATTTT
CTTCCTAAGGAAGAATTGGTTCGCATGTTTGAAAGAGAAACTTATCTTTTCGGGTAAGGCTGTTTACTGCACGGTAACGGCTAGATGTCCCTTAAAGTTTGACGAGGTTCGAGAAAAAGGTGTTTACAGAGATATCTTTTCGGAAAATGATGTGGTTGAT
GTGATGGAAATGAAGTTACAAAAACTTTTACATCATGAAGGTGACAAATGGAGGAGAAAAATTGTCTTTGATGTTCCGCGTATGTCTTCTAAGAGAGTTCCGGTTCCTAGAATTGGTACCGTAAAGTGCATTCAAAGTTGGTTTGATGCTACTTTTCCTG
GTAACTCAATCAGGTTCTCAGATTTTGATGGTTACACTGTTGCAACATCGGATCTTGCCGTTGATGTGACAGATTGTAAAATAAAAATTGGAAAAACTTTTAAGCCGTACGTTTTTAAAAGCGCTCTGCGCCCTGAATTAAGGACATCAATGCCTGAAAA
GAGACAAGGTAGTTTAGCCGAAAGTGTGTTGGCTTTGAGAAAAAGAAACTTAGCAGCGCCCAGATTGCAAGGGGCTGTTAACGAATGGCAGGTGATTGAAAAAACTATGAAGAGGGCTTTGGATATTTTCTTTATTAAGGATTTGATTGATAGGTCGCAC
CATGCTTCCTATGAATCAGCCTTAAAATGGTGGGATAAGCAGTCAACTACTGCCCGTGCTCAACTTGCAGCAGAGACGAGACGACTTTGTGACATCGACTTCTGTACTTACAATTTTATGATCAAAAATGATGTAAAACCGAAGTTGGATCTGACACCCC
AAGTTGAGTATGCAGCTTTGCAAACGGTAGTATTTCCTGATAAAATCGTGAATGCTTTTTTCGGCCCTGTTATAAAGGAAATTAATGAGAGGATCATTAGAGCCTTGAAACCTCATATTGTGTTTAATACACGTATGACGGCCGAAGGTTTAAATGATAC
TATTTCATTTCTGCCCCCCAAGTCTTACAGGTCACTTGAAATCGATTTCTCAAAATTTGACAAGTCAAAGACTGGTCTGCATATTAAATGTGTAATAGCTTTATACAAATTGTTTGGACTGGATGGAATCCTAGAGGTGCTCTGGGAGAAATCTCAAGTA
CAAACATACGTAAAGGACCGAAATTTTGGTCTTGAAGCTTACCTTTTATTTCAGCAGAAATCAGGAAATTGTGACACTTACGGTTCTAATACTTGGTCTGCAGCATTAGCGTTGCTAGATTGTTTGCCTTTGGAGCATTCAGTCTATGCGGTTTTTGGTG
GAGACGATTCTTTGATACTATTTGAGGAGGGGTATCTTGTTAATGACCCATGCAAGCGTTTGGCTGGAACGTGGAATTTTGAATGCAAAATTTTTGCTTTCAATTTTCCTTCCTTTTGCGGGAAGTTTGTTGTCAGTGTTGACGACAAATATGTTTTTGT
ACCAGATGCTGCTAAGCTAATCACAAAACTGGGGAGAACCGACGTAAAGGATGTAGAAGCTTTAAGTGAAATTTACATTTCAATTAATGACAATTACAGATCCTTTAAGGATTTCCGTGTTTTGGATGAGTTGAGCGATGCTTTGGTTGATAGATATCAT
GCCCCTTACGATGCTTTATCTGCTTTGGTGTCTCTGTGTTACTATATATTCGATTTCGACAACTTTAAGCAGCTGTTTGGTTGTGATGGCAATTTTGTGGATAAAATAATTGGAAAGGAT
```

CLUSTAL multiple sequence alignment by MUSCLE (3.8)

```
L.vulgare_iso-3_CH_Biel           ------------------------TTGATATTTTTCAATGATGGCTTTGCCTAGCTTCGC
L.vulgare_iso-4_CH_Biel           -----------------------TTTGATATTTTTCAATGATGGCTTTGCCTAGCTTCGC
L.vulgare_iso-1_CH_Nyon_Gla       -----------------------TTTGATATTTTTC-ATGATGGCTTTGCCTAGCTTCGC
LigMV_gamma_CH_sRNA               TTTACCAACAAACATTATCAATCTTTGATATTTTTCAATGATGGCTTTGCCTAGCTTCGC
L.vulgare_iso-2_CH_Nyon_Gen       -----------------------TTTGATATTTTTCAATGATGGCTTTGCCTAGCTTCGC
L.vulgare_iso-8_CH_Nyon_Pra       -----------------------TTTGNTATTTTTCAATGATGGCTTTGCCTAGCTTCGC
LigMV_FR                          TTTACCAACAAACATTATCAATCTTTGATATTTTTCAACGATGGCTTTGCCTAGCTTCGC
L.vulgare_iso-6_AUT_Inns          ----------------------CTTTGATATTTTTC-NTGATGGCTTTGCCTAGCTTCGC
LigMV_AUT                         TTTACCAACAAACATTATCAATCTTTGATATTTTTCAATGATGGCTTTGCCTAGCTTCGC
L.vulgare_iso-5_CH_Veve           ----------------------CTTTGATATTTTTCAATGATGGCTTTGCCTAGCTTCGC
L.vulgare_iso-7_CH_Nyon_Gen       -----------------------TTTGATATTTTTCAATGATGGCTTTGCCTAGCTTCGC
C.quinoa_iso-1288_CH_2007         -----------------------TTTGATATTTTTC-NTGATGGCTTTGCCTAGCTTCGC
LigMV_CH_dsRNA                    TTTACCAACAAACATTATCAATCTTTGATATTTTTCAATGATGGCTTTGCCTAGCTTCGC
C.quinoa_iso-1340_CH_2007         ----------------------CTTTGATATTTTTC-NTGATGGCTTTGCCTAGCTTCGC
                                                          *** ********   *********************

L.vulgare_iso-3_CH_Biel           GCTCGCAAAGCCTAATCCCAAACTGTGTTTTAAGAAACTTCAAGTATTAGCATTCGTTGC
L.vulgare_iso-4_CH_Biel           GCTCGCAAAGCCTAATCCCAAACTGTGTTTTAAGAAACTTCAAGTATTAGCATTCGTTGC
L.vulgare_iso-1_CH_Nyon_Gla       GCTCGCAAAGCCTAATCCCAAACTGTGTTTTAAGAAACTTCAAGTATTAGCATTCGTTGC
LigMV_gamma_CH_sRNA               GCTCGCAAAGCCTAATCCCAAACTGTGTTTTAAGAAACTTCAAGTATTAGCATTCGTTGC
L.vulgare_iso-2_CH_Nyon_Gen       GCTCGCAAAGCCTAATCCCAAACTGTGTTTTAAGAAACTTCAAGTATTAGCATTCGTTGC
L.vulgare_iso-8_CH_Nyon_Pra       GCTCGCAAGGCCTAATCCCAAACTGTGTTTTAAGAAACTTCAAGTATTAGCATTCGTTGC
LigMV_FR                          GCTCGCAAAGCCTAATCCCAAACTGTGTTTTAAGAAACTTCAAGTATTAGCATTCGTTGC
L.vulgare_iso-6_AUT_Inns          GCTCGCAAAGCCTAATCCCAAACTGTGTTTTAAGAAACTTCAAGTATTAGCATTCGTTGC
LigMV_AUT                         GCTCGCAAAGCCTAATCCCAAACTGTGTTTTAAGAAACTTCAAGTATTAGCATTCGTTGC
L.vulgare_iso-5_CH_Veve           GCTCGCAAAGCCTAATCCCAAACTGTGTTTTAAGAAACTTCAAGTATTAGCATTCGTTGC
L.vulgare_iso-7_CH_Nyon_Gen       GCTCGCAAAGCCTAATCCCAAACTGTGTTTTAAGAAACTTCAAGTATTAGCATTCGTTGC
C.quinoa_iso-1288_CH_2007         GCTCGCAAAGCCTAATCCCAAACTGTGTTTTAAGAAACTTCAAGTATTAGCATTCGTTGC
LigMV_CH_dsRNA                    GCTCGCAAAGCCTAATCCCAAACTGTGTTTTAAGAAACTTCAAGTATTAGCATTCGTTGC
C.quinoa_iso-1340_CH_2007         GCTCGCAAAGCCTAATCCCAAACTGTGTTTTAAGAAACTTCAAGTATTAGCATTCGTTGC
                                  ******** ***************************************************

L.vulgare_iso-3_CH_Biel           GTTACTTGTGAGGTCGAAAAAAACGTTTATTCTCTCTGTCTTAAAAAGATGGTTTGGGAT
L.vulgare_iso-4_CH_Biel           GTTACTTGTGAGGTCGAAAAAAACGTTTATTCTCTCTGTCTTAAAAAGATGGTTTGGGAT
L.vulgare_iso-1_CH_Nyon_Gla       GTTACTTGTGAGGTCGAAAAAAACGTTTATTCTCTCTGTCTTAAAAAGATGGTTTGGGAT
LigMV_gamma_CH_sRNA               GTTACTTGTGAGGTCGAAAAAAACGTTTATTCTCTCTGTCTTAAAAAGATGGTTTGGGAT
L.vulgare_iso-2_CH_Nyon_Gen       GTTACTTGTGAGGTCGAAAAAAACGTTTATTCTCTCTGTCTTAAAAAGATGGTTTGGGAT
L.vulgare_iso-8_CH_Nyon_Pra       GTTACTTGTGAGGTCGAAAAAAACGTTTATTCTCTCTGTCTTAAAAAGATGGTTTGGGAT
LigMV_FR                          GTTACTTGTGAGGTCGAAAAAAACGTTTATTCTCTCTGTCTTAAAAAGATGGTTTGGGAT
L.vulgare_iso-6_AUT_Inns          GTTACTTGTGAGGTCGAAAAAAACGTTTATTCTCTCTGTCTTAAAAAGATGGTTTGGGAT
LigMV_AUT                         GTTACTTGTGAGGTCGAAAAAAACGTTTATTCTCTCTGTCTTAAAAAGATGGTTTGGGAT
L.vulgare_iso-5_CH_Veve           GTTACTTGTGAGGTCGAAAAAAACGTTTATTCTCTCTGTCTTAAAAAGATGGTTTGGGAT
L.vulgare_iso-7_CH_Nyon_Gen       GTTACTTGTGAGGTCGAAAAAAACGTTTATTCTCTCTGTCTTAAAAAGATGGTTTGGGAT
C.quinoa_iso-1288_CH_2007         GTTACTTGTGAGGTCGAAAAAAACGTTTATTCTCTCTGTCTTAAAAAGATGGTTTGGGAT
LigMV_CH_dsRNA                    GTTACTTGTGAGGTCGAAAAAAACGTTTATTCTCTCTGTCTTAAAAAGATGGTTTGGGAT
C.quinoa_iso-1340_CH_2007         GTTACTTGTGAGGTCGAAAAAAACGTTTATTCTCTCTGTCTTAAAAAGATGGTTTGGGAT
                                  ************************************************************

L.vulgare_iso-3_CH_Biel           TTTCTTCCTAAGGAAGAATTGGTTCGCATGTTTGAAAGAGAAACTTATCTTTTCGGGTAA
L.vulgare_iso-4_CH_Biel           TTTCTTCCTAAGGAAGAATTGGTTCGCATGTTTGAAAGAGAAACTTATCTTTTCGGGTAA
L.vulgare_iso-1_CH_Nyon_Gla       TTTCTTCCTAAGGAAGAATTGGTTCGCATGTTTGAAAGAGAAACTTATCTTTTCGGGTAA
LigMV_gamma_CH_sRNA               TTTCTTCCTAAGGAAGAATTGGTTCGCATGTTTGAAAGAGAAACTTATCTTTTCGGGTAA
L.vulgare_iso-2_CH_Nyon_Gen       TTTCTTCCTAAGGAAGAATTGGTTCGCATGTTTGAAAGAGAAACTTATCTTTTCGGGTAA
L.vulgare_iso-8_CH_Nyon_Pra       TTTCTTCCTAAGGAAGAATTGGTTCGCATGTTTGAAAGAGAAACTTATCTTTTCGGGTAA
LigMV_FR                          TTTCTTCCTAAGGAAGAATTGGTTCGCATGTTTGAAAGAGAAACTTATCTTTTCGGGTAA
L.vulgare_iso-6_AUT_Inns          TTTCTTCCTAAGGAAGAATTGGTTCGCATGTTTGAAAGAGAAACTTATCTTTTCGGGTAA
LigMV_AUT                         TTTCTTCCTAAGGAAGAATTGGTTCGCATGTTTGAAAGAGAAACTTATCTTTTCGGGTAA
L.vulgare_iso-5_CH_Veve           TTTCTTCCTAAGGAAGAATTGGTTCGCATGTTTGAAAGAGAAACTTATCTTTTCGGGTAA
L.vulgare_iso-7_CH_Nyon_Gen       TCTCTTCCTAAGGAAGAATTGGTTCGCATGTTTGAAAGAGAAACTTATCTTTTCGGGTAA
C.quinoa_iso-1288_CH_2007         TCTCTTCCTAAGGAAGAATTGGTTCGCATGTTTGAAAGAGAAACTTATCTTTTCGGGTAA
LigMV_CH_dsRNA                    TCTCTTCCTAAGGAAGAATTGGTTCGCATGTTTGAAAGAGAAACTTATCTTTTCGGGTAA
C.quinoa_iso-1340_CH_2007         TCTCTTCCTAAGGAAGAATTGGTTCGCATGTTTGAAAGAGAAACTTATCTTTTCGGGTAA
```

```
                                  * **********************************************************

L.vulgare_iso-3_CH_Biel           GGCTGTTTACTGCACGGTAACGGCTAGATGTCCCTTAAAGTTTGACGAGGTTCGAGAAAA
L.vulgare_iso-4_CH_Biel           GGCTGTTTACTGCACGGTAACGGCTAGATGTCCCTTAAAGTTTGACGAGGTTCGAGAAAA
L.vulgare_iso-1_CH_Nyon_Gla       GGCTGTTTACTGCACGGTAACGGCTAGATGTCCCTTAAAGTTTGACGAGGTTCGAGAAAA
LigMV_gamma_CH_sRNA               GGCTGTTTACTGCACGGTAACGGCTAGATGTCCCTTAAAGTTTGACGAGGTTCGAGAAAA
L.vulgare_iso-2_CH_Nyon_Gen       GGCTGTTTACTGCACGGTAACGGCTAGATGTCCCTTAAAGTTTGACGAGGTTCGAGAAAA
L.vulgare_iso-8_CH_Nyon_Pra       GGCTGTTTACTGCACGGTAACGGCTAGATGTCCCTTAAAGTTTGACGAGGTTCGAGAAAA
LigMV_FR                          GGCTGTTTACTGCACGGTAACGGCTAGATGTCCCTTAAAGTTTGACGAGGTTCGAGAAAA
L.vulgare_iso-6_AUT_Inns          GGCTGTTTACTGCACGGTAACGGCTAGATGTCCCTTAAAGTTTGACGAGGTTCGAGAAAA
LigMV_AUT                         GGCTGTTTACTGCACGGTAACGGCTAGATGTCCCTTAAAGTTTGACGAGGTTCGAGAAAA
L.vulgare_iso-5_CH_Veve           GGCTGTTTACTGCACGGTAACGGCTAGATGTCCCTTAAAGTTTGACGAGGTTCGAGAAAA
L.vulgare_iso-7_CH_Nyon_Gen       GGCTGTTTACTGCACGGTAACGGCTAGATGTCCCTTAAAGTTTGACGAGGTTCGAGAAAA
C.quinoa_iso-1288_CH_2007         GGCTGTTTACTGCACGGTAACGGCTAGATGTCCCTTAAAGTTTGACGAGGTTCGAGAAAA
LigMV_CH_dsRNA                    GGCTGTTTACTGCACGGTAACGGCTAGATGTCCCTTAAAGTTTGACGAGGTTCGAGAAAA
C.quinoa_iso-1340_CH_2007         GGCTGTTTACTGCACGGTAACGGCTAGATGTCCCTTAAAGTTTGACGAGGTTCGAGAAAA
                                  ************************************************************

L.vulgare_iso-3_CH_Biel           GGGGGTTTACAGAGATATCTTTTCGGAAAATGATGTGGTTGATGTGATGGAAATGAAGTT
L.vulgare_iso-4_CH_Biel           AGGGGTTTACAGAGATATCTTTTCGGAAAATGATGTGGTTGATGTGATGGAAATGAAGTT
L.vulgare_iso-1_CH_Nyon_Gla       GGGTGTTTACAGAGATATCTTTTCGGAAAATGATGTGGTTGATGTGATGGAAATGAAGTT
LigMV_gamma_CH_sRNA               GGGTGTTTACAGAGATATCTTTTCGGAAAATGATGTGGTTGATGTGATGGAAATGAAGTT
L.vulgare_iso-2_CH_Nyon_Gen       AGGTGTTTACAGAGATATCTTTTCGGAAAATGATGTGGTTGATGTGATGGAAATGAAGTT
L.vulgare_iso-8_CH_Nyon_Pra       AGGTGTTTACAGAGATATCTTTTCGGAAAATGATGTGGTTGATGTGATGGAAATGAAGTT
LigMV_FR                          AGGTGTTTACAGAGATATCTTTTCGGAAAATGATGTGGTTGATGTGATGGAAATGAAGTT
L.vulgare_iso-6_AUT_Inns          AGGTGTTTACAGAGATATCTTTTCGGAAAATGATGTGGTTGATGTGATGGAAATGAAGTT
LigMV_AUT                         AGGTGTTTACAGAGATATCTTTTCGGAAAATGATGTGGTTGATGTGATGGAAATGAAGTT
L.vulgare_iso-5_CH_Veve           AGGTGTTTACAGAGATATCTTTTCGGAAAATGATGTGGTTGATGTGATGGAAATGAAGTT
L.vulgare_iso-7_CH_Nyon_Gen       GGGTGTTTACAGAGATATCTTTTCGGAAAATGATGTGGTTGATGTGATGGAAATGAAGTT
C.quinoa_iso-1288_CH_2007         GGGTGTTTACAGAGATATCTTTTCGGAAAATGATGTGGTTGATGTGATGGAAATGAAGTT
LigMV_CH_dsRNA                    GGGTGTTTACAGAGATATCTTTTCGGAAAATGATGTGGTTGATGTGATGGAAATGAAGTT
C.quinoa_iso-1340_CH_2007         GGGTGTTTACAGAGATATCTTTTCGGAAAATGATGTGGTTGATGTGATGGAAATGAAGTT
                                   ** ********************************************************

L.vulgare_iso-3_CH_Biel           ACAAAAACTTTTACATCATGAAGGTGACAAATGGAGGAGAAAAATTGTCTTTGATGTTCC
L.vulgare_iso-4_CH_Biel           ACAAAAACTTTTACATCATGAAGGTGACAAATGGAGGAGAAAAATTGTCTTTGATGTTCC
L.vulgare_iso-1_CH_Nyon_Gla       ACAAAAACTTTTACATCATGAAGGTGACAAATGGAGGAGAAAAATTGTCTTTGATGTTCC
LigMV_gamma_CH_sRNA               ACAAAAACTTTTACATCATGAAGGTGACAAATGGAGGAGAAAAATTGTCTTTGATGTTCC
L.vulgare_iso-2_CH_Nyon_Gen       ACAAAAACTTTTACATCATGAAGGTGACAAATGGAGGAGAAAAATTGTCTTTGATGTTCC
L.vulgare_iso-8_CH_Nyon_Pra       ACAAAAACTTTTACATCATGAAGGTGACAAATGGAGGAGAAAAATTGTCTTTGATGTTCC
LigMV_FR                          ACAAAAACTTTTACATCATGAAGGTGACAAATGGAGGAGAAAAATTGTCTTTGATGTTCC
L.vulgare_iso-6_AUT_Inns          ACAAAAACTTTTACATCATGAAGGTGACAAATGGAGGAGAAAAATTGTCTTTGATGTTCC
LigMV_AUT                         ACAAAAACTTTTACATCATGAAGGTGACAAATGGAGGAGAAAAATTGTCTTTGATGTTCC
L.vulgare_iso-5_CH_Veve           ACAAAAACTTTTACATCATGAAGGTGACAAATGGAGGAGAAAAATTGTCTTTGATGTTCC
L.vulgare_iso-7_CH_Nyon_Gen       ACAAAAACTTTTACATCATGAAGGTGACAAATGGAGGAGAAAAATTGTCTTTGATGTTCC
C.quinoa_iso-1288_CH_2007         ACAAAAACTTTTACATCATGAAGGTGACAAATGGAGGAGAAAAATTGTCTTTGATGTTCC
LigMV_CH_dsRNA                    ACAAAAACTTTTACATCATGAAGGTGACAAATGGAGGAGAAAAATTGTCTTTGATGTTCC
C.quinoa_iso-1340_CH_2007         ACAAAAACTTTTACATCATGAAGGTGACAAATGGAGGAGAAAAATTGTCTTTGATGTTCC
                                  ************************************************************

L.vulgare_iso-3_CH_Biel           GCGTATGTCTTCTAAGAGAGTTCCGGTTCCTAGAATTGGTACCGTAAAGTGCATTCAAAG
L.vulgare_iso-4_CH_Biel           GCGTATGTCTTCTAAGAGAGTTCCGGTTCCTAGAATTGGTACCGTAAAGTGCATTCAAAG
L.vulgare_iso-1_CH_Nyon_Gla       GCGTATGTCTTCTAAGAGAGTTCCGGTTCCTAGAATTGGTACCGTAAAGTGCATTCAAAG
LigMV_gamma_CH_sRNA               GCGTATGTCTTCTAAGAGAGTTCCGGTTCCTAGAATTGGTACCGTAAAGTGCATTCAAAG
L.vulgare_iso-2_CH_Nyon_Gen       GCGTATGTCTTCTAAGAGAGTTCCGGTTCCTAGAATTGGTACCGTAAAGTGCATTCAAAG
L.vulgare_iso-8_CH_Nyon_Pra       GCGTATGTCTTCTAAGAGAGTTCCGGTTCCTAGAATTGGTACCGTAAAGTGCATTCAAAG
LigMV_FR                          GCGTATGTCTTCTAAGAGAGTTCCGGTTCCTAGAATTGGTACCGTAAAGTGCATTCAAAG
L.vulgare_iso-6_AUT_Inns          GCGTATGTCTTCTAAGAGAGTTCCGGTTCCTAGAATTGGTACCGTAAAGTGCATTCAAAG
LigMV_AUT                         GCGTATGTCTTCTAAGAGAGTTCCGGTTCCTAGAATTGGTACCGTAAAGTGCATTCAAAG
L.vulgare_iso-5_CH_Veve           GCGTATGTCTTCTAAGAGAGTTCCGGTTCCTAGAATTGGTACCGTAAAGTGCATTCAAAG
L.vulgare_iso-7_CH_Nyon_Gen       GCGTATGTCTTCTAAGAGAGTTCCGGTTCCTAGAATTGGTACCGTAAAGTGCATTCAAAG
C.quinoa_iso-1288_CH_2007         GCGTATGTCTTCTAAGAGAGTTCCGGTTCCTAGAATTGGTACCGTAAAGTGCATTCAAAG
LigMV_CH_dsRNA                    GCGTATGTCTTCTAAGAGAGTTCCGGTTCCTAGAATTGGTACCGTAAAGTGCATTCAAAG
C.quinoa_iso-1340_CH_2007         GCGTATGTCTTCTAAGAGAGTTCCGGTTCCTAGAATTGGTACCGTAAAGTGCATTCAAAG
                                  ************************************************************

L.vulgare_iso-3_CH_Biel           TTGGTTTGATGCTACTTTTCCTGGTAACTCAATCAGGTTCTCAGATTTTGATGGTTACAC
L.vulgare_iso-4_CH_Biel           TTGGTTTGATGCTACTTTTCCTGGTAACTCAATCAGGTTCTCAGATTTTGATGGTTACAC
L.vulgare_iso-1_CH_Nyon_Gla       TTGGTTTGATGCTACTTTTCCTGGTAACTCAATCAGGTTCTCAGATTTTGATGGTTACAC
LigMV_gamma_CH_sRNA               TTGGTTTGATGCTACTTTTCCTGGTAACTCAATCAGGTTCTCAGATTTTGATGGTTACAC
L.vulgare_iso-2_CH_Nyon_Gen       TTGGTTTGATGCTACTTTTCCTGGTAACTCAATCAGGTTCTCAGATTTTGATGGTTACAC
L.vulgare_iso-8_CH_Nyon_Pra       TTGGTTTGATGCTACTTTTCCTGGTAACTCAATCAGGTTCTCAGATTTTGATGGTTACAC
LigMV_FR                          TTGGTTTGATGCTACTTTTCCTGGTAACTCAATCAGGTTCTCAGATTTTGATGGTTACAC
L.vulgare_iso-6_AUT_Inns          TTGGTTTGATGCTACTTTTCCTGGTAACTCAATCAGGTTCTCAGATTTTGATGGTTACAC
LigMV_AUT                         TTGGTTTGATGCTACTTTTCCTGGTAACTCAATCAGGTTCTCAGATTTTGATGGTTACAC
L.vulgare_iso-5_CH_Veve           TTGGTTTGATGCTACTTTTCCTGGTAACTCAATCAGGTTCTCAGATTTTGATGGTTACAC
L.vulgare_iso-7_CH_Nyon_Gen       TTGGTTTGATGCTACTTTTCCTGGTAACTCAATCAGGTTCTCAGATTTTGATGGTTACAC
C.quinoa_iso-1288_CH_2007         TTGGTTTGATGCTACTTTTCCTGGTAACTCAATCAGGTTCTCAGATTTTGATGGTTACAC
LigMV_CH_dsRNA                    TTGGTTTGATGCTACTTTTCCTGGTAACTCAATCAGGTTCTCAGATTTTGATGGTTACAC
C.quinoa_iso-1340_CH_2007         TTGGTTTGATGCTACTTTTCCTGGTAACTCAATCAGGTTCTCAGATTTTGATGGTTACAC
                                  ************************************************************

L.vulgare_iso-3_CH_Biel           TGTTGCAATATCGGATCTTGCCGTTGATGTGACAGATTGTAAAATAAAATTTGGAAAAAC
L.vulgare_iso-4_CH_Biel           TGTTGCAATATCGGATCTTGCCGTTGATGTGACAGATTGTAAAATAAAATTTGGAAAAAC
L.vulgare_iso-1_CH_Nyon_Gla       TGTTGCAATATCGGATCTTGCCGTTGATGTGACAGATTGTAAAATAAAAATTGGAAAAAC
LigMV_gamma_CH_sRNA               TGTTGCAATATCGGATCTTGCCGTTGATGTGACAGATTGTAAAATAAAAATTGGAAAAAC
L.vulgare_iso-2_CH_Nyon_Gen       TGTTGCAATATCGGATCTTGCCGTTGATGTGACAGATTGTAAAATAAAATTTGGAAAAAC
L.vulgare_iso-8_CH_Nyon_Pra       TGTTGCAACATCGGATCTTGCCGTTGATGTGACAGATTGTAAAATAAAAATTGGAAAAAC
LigMV_FR                          TGTTGCAATATCGGATCTTGCCGTTGATGTGACAGATTGTAAAATAAAAATTGGAAAAAC
L.vulgare_iso-6_AUT_Inns          TGTTGCAATATCGGATCTTGCCGTTGATGTGACAGATTGTAAAATAAAAATTGGAAAAAC
LigMV_AUT                         TGTTGCAACATCGGATCTTGCCGTTGATGTGACAGATTGTAAAATAAAAATTGGAAAAAC
L.vulgare_iso-5_CH_Veve           TGTTGCAATATCGGATCTTGCCGTTGATGTGACAGATTGTAAAATAAAAATTGGAAAAAC
L.vulgare_iso-7_CH_Nyon_Gen       TGTTGCAATATCGGATCTTGCCGTTGATGTGACAGATTGTAAAATAAAAATTGGAAAAAC
C.quinoa_iso-1288_CH_2007         TGTTGCAATATCGGATCTTGCCGTTGATGTGACAGATTGTAAAATAAAAATTGGAAAAAC
LigMV_CH_dsRNA                    TGTTGCAATATCGGATCTTGCCGTTGATGTGACAGATTGTAAAATAAAAATTGGAAAAAC
C.quinoa_iso-1340_CH_2007         TGTTGCAATATCGGATCTTGCCGTTGATGTGACAGATTGTAAAATAAAAATTGGAAAAAC
                                  ******** **************************************** **********

L.vulgare_iso-3_CH_Biel           TTTTAAGCCGTACGTTTTTAAAAGCGCTTTGCGCCCTGAATTAAGGACATCAATGCCTGA
L.vulgare_iso-4_CH_Biel           TTTTAAGCCGTACGTTTTCAAAAGCGCTTTGCGCCCTGAATTAAGGACATCAATGCCTGA
L.vulgare_iso-1_CH_Nyon_Gla       TTTTAAGCCGTACGTTTTTAAAAGCGCTCTGCGCCCTGAATTAAGGACATCAATGCCTGA
LigMV_gamma_CH_sRNA               TTTTAAGCCGTACGTTTTTAAAAGCGCTCTGCGCCCTGAATTAAGGACATCAATGCCTGA
L.vulgare_iso-2_CH_Nyon_Gen       TTTTAAGCCGTACGTTTTTAAAAGCGCTCTGCGCCCTGAATTAAGGACATCAATGCCTGA
L.vulgare_iso-8_CH_Nyon_Pra       TTTTAAGCCGTACGTTTTTAAAAGCGCTCTGCGCCCTGAATTAAGGACATCAATGCCTGA
LigMV_FR                          TTTTAAGCCGTACGTTTTTAAAAGCGCTCTGCGTCCTGAATTAAGGACATCAATGCCTGA
L.vulgare_iso-6_AUT_Inns          TTTTAAGCCGTACGTTTTTAAAAGCGCTCTGCGCCCTGAATTAAGGACATCAATGCCTGA
LigMV_AUT                         TTTTAAGCCGTACGTTTTTAAAAGCGCTCTGCGCCCTGAATTAAGGACATCAATGCCTGA
L.vulgare_iso-5_CH_Veve           TTTTAAGCCGTACGTTTTTAAAAGCGCTCTGCGCCCTGAATTAAGGACATCAATGCCTGA
L.vulgare_iso-7_CH_Nyon_Gen       TTTTAAGCCGTACGTTTTTAAAAGCGCTCTGCGCCCTGAATTGAGGACATCAATGCCTGA
C.quinoa_iso-1288_CH_2007         TTTTAAGCCGTACGTTTTTAAAAGCGCTCTGCGCCCTGAATTAAGGACATCAATGCCTGA
LigMV_CH_dsRNA                    TTTTAAGCCGTACGTTTTTAAAAGCGCTCTGCGCCCTGAATTAAGGACATCAATGCCTGA
C.quinoa_iso-1340_CH_2007         TTTTAAGCCGTACGTTTTTAAAAGCGCTCTGCGCCCTGAATTAAGGACATCAATGCCTGA
                                  ****************** ********* **** ******** *****************

L.vulgare_iso-3_CH_Biel           AAAGAGACAAGGTAGTTTAGCCGAAAGTGTGTTGGCTTTGAGAAAAAGAAACTTAGCAGC
L.vulgare_iso-4_CH_Biel           AAAGAGACAAGGTAGTTTAGCCGAAAGTGTGTTGGCTTTGAGAAAAAGAAACTTAGCAGC
L.vulgare_iso-1_CH_Nyon_Gla       AAAGAGACAAGGTAGTTTAGCCGAAAGTGTGTTGGCTTTGAGAAAAAGAAACTTAGCAGC
LigMV_gamma_CH_sRNA               AAAGAGACAAGGTAGTTTAGCCGAAAGTGTGTTGGCTTTGAGAAAAAGAAACTTAGCAGC
L.vulgare_iso-2_CH_Nyon_Gen       AAAGAGACAAGGTAGTTTAGCCGAAAGTGTGTTGGCTTTGAGAAAAAGAAACTTAGCAGC
L.vulgare_iso-8_CH_Nyon_Pra       AAAGAGACAAGGTAGTTTAGCCGAAAGTGTGTTGGCTTTGAGAAAAAGAAACTTAGCAGC
LigMV_FR                          AAAGAGACAAGGTAGTTTAGCCGAAAGTGTGTTGGCTTTGAGAAAAAGAAACTTAGCAGC
L.vulgare_iso-6_AUT_Inns          AAAGAGACAAGGTAGTTTAGCCGAAAGTGTGTTGGCTTTGAGAAAAAGAAACTTAGCAGC
LigMV_AUT                         AAAGAGACAAGGTAGTTTAGCCGAAAGTGTGTTGGCTTTGAGAAAAAGAAACTTAGCAGC
L.vulgare_iso-5_CH_Veve           AAAGAGACAAGGTAGTTTAGCCGAAAGTGTGTTGGCTTTGAGAAAAAGAAACTTAGCAGC
L.vulgare_iso-7_CH_Nyon_Gen       GAAGAGACAAGGTAGTTTAGCCGAAAGTGTGTTGGCTTTGAGAAAAAGAAACTTAGCAGC
C.quinoa_iso-1288_CH_2007         GAAGAGACAAGGTAGTTTAGCCGAAAGTGTGTTGGCTTTGAGAAAAAGAAACTTAGCAGC
LigMV_CH_dsRNA                    AAAGAGACAAGGTAGTTTAGCCGAAAGTGTGTTGGCTTTGAGAAAAAGAAACTTAGCAGC
C.quinoa_iso-1340_CH_2007         AAAGAGACAAGGTAGTTTAGCCGAAAGTGTGTTGGCTTTGAGAAAAAGAAACTTAGCAGC
```

```
 ***********************************************************

L.vulgare_iso-3_CH_Biel          GCCCAGATTGCAAGGGGCTGTTAACGAATGGCAGGTGATTGAAAACACTATGAAGAGGGC
L.vulgare_iso-4_CH_Biel          GCCCAGATTGCAAGGGGCTGTTAACGAATGGCAGGTGATTGAAAACACTATGAAGAGGGC
L.vulgare_iso-1_CH_Nyon_Gla      ACCCAGATTGCAAGGGGCTGTTRACGAATGGCAGGTGATTGAAAATACTATGAAGAGGGC
LigMV_gamma_CH_sRNA              ACCCAGATTGCAAGGGGCTGTTAACGAATGGCAGGTGATTGAAAATACTATGAAGAGGGC
L.vulgare_iso-2_CH_Nyon_Gen      GCCCAGATTGCAAGGGGCTGTTAACGAATGGCAGGTGATTGAAAACACTATGAAGAGGGC
L.vulgare_iso-8_CH_Nyon_Pra      GCCCAGATTGCAAGGGGCTGTTAACGAATGGCAGGTGATTGAAAAAACTATGAAGAGGGC
LigMV_FR                         GCCCAGATTGCAAGGGGCTGTTAACGAATGGCAGGTGATTGAAAACACTATGAAGAGGGC
L.vulgare_iso-6_AUT_Inns         GCCCAGATTGCAAGGGGCTGTTAACGAATGGCAGGTGATTGAAAACACTATGAAGAGGGC
LigMV_AUT                        GCCCAGATTGCAAGGGGCTGTTAACGAATGGCAGGTGATTGAAAACACTATGAAGAGGGC
L.vulgare_iso-5_CH_Veve          GCCCAGATTGCAAGGGGCTGTTAACGAATGGCAGGTGATTGAAAACACTATGAAGAGGGC
L.vulgare_iso-7_CH_Nyon_Gen      GCCCAGATTGCAAGGGGCTGTTAACGAATGGCAGGTGATTGAAAACACTATGAAGAGGGC
C.quinoa_iso-1288_CH_2007        GCCCAGATTGCAAGGGGCTGTTAACGAATGGCAGGTGATTGAAAACACTATGAAGAGGGC
LigMV_CH_dsRNA                   GCCCAGATTGCAAGGGGCTGTTAACGAATGGCAGGTGATTGAAAACACTATGAAGAGGGC
C.quinoa_iso-1340_CH_2007        GCCCAGATTGCAAGGGGCTGTTAACGAATGGCAGGTGATTGAAAACACTATGAAGAGGGC
                                  ********************* ********************** **************

L.vulgare_iso-3_CH_Biel          TTTGGATATTTTCTTTAATAAGGATTTGATTGATAGGTCGCACCATGCTTCCTATGAATC
L.vulgare_iso-4_CH_Biel          TTTGGATATTTTCTTTAATAAGGATTTGATTGATAGGTCGCACCATGCTTCCTATGAATC
L.vulgare_iso-1_CH_Nyon_Gla      TTTGGATATTTTCTTTATTAAGGATTTGATTGATAGGTCGCACCATGCTTCCTATGAATC
LigMV_gamma_CH_sRNA              TTTGGATATTTTCTTTATTAAGGATTTGATTGATAGGTCGCACCATGCTTCCTATGAATC
L.vulgare_iso-2_CH_Nyon_Gen      TTTGGATATTTTCTTTATTAAGGATTTGATTGATAGGTCGCACCATGCTTCCTATGAATC
L.vulgare_iso-8_CH_Nyon_Pra      TTTGGATATTTTCTTTATTAAGGATTTGATTGATAGGTCGCACCATGCTTCCTATGAATC
LigMV_FR                         TTTGGATATTTTCTTTATTAAGGATTTGATTGATAGGTCGCACCATGCTTCCTATGAATC
L.vulgare_iso-6_AUT_Inns         TTTGGATATTTTCTTTAATAAGGATCTGATTGATAGGTCGCACCATGCTTCCTATGAATC
LigMV_AUT                        TTTGGATATTTTCTTTAATAAGGATTTGATTGATAGGTCGCACCATGCTTCCTATGAATC
L.vulgare_iso-5_CH_Veve          TTTGGATATTTTCTTTATTAAGGATTTGATTGATAGGTCGCACCATGCTTCCTATGAATC
L.vulgare_iso-7_CH_Nyon_Gen      TTTGGATATTTTCTTTATTAAGGATTTGATTGATAGGTCGCACCATGCTTCCTATGAATC
C.quinoa_iso-1288_CH_2007        TTTGGATATTTTCTTTATTAAGGATTTGATTGATAGGTCGCACCATGCTTCCTATGAATC
LigMV_CH_dsRNA                   TTTGGATATTTTCTTTATTAAGGATTTGATTGATAGGTCGCACCATGCTTCCTATGAATC
C.quinoa_iso-1340_CH_2007        TTTGGATATTTTCTTTATTAAGGATTTGATTGATAGGTCGCACCATGCTTCCTATGAATC
                                 ***************** ******* **********************************

L.vulgare_iso-3_CH_Biel          AGCCTTAAAGTGGTGGGATAAGCAGTCAACTACTGCCCGTGCTCAACTTGCAGCAGAGAC
L.vulgare_iso-4_CH_Biel          AGCCTTAAAGTGGTGGGATAAGCAGTCAACTACTGCCCGTGCTCAACTTGCAGCAGAGAC
L.vulgare_iso-1_CH_Nyon_Gla      AGCCTTAAAATGGTGGGATAAGCAGTCAACTACTGCCCGTGCTCAACTTGCARCTGAGAC
LigMV_gamma_CH_sRNA              AGCCTTAAAATGGTGGGATAAGCAGTCAACTACTGCCCGTGCTCAACTTGCAGCTGAGAC
L.vulgare_iso-2_CH_Nyon_Gen      AGCCTTAAAATGGTGGGATAAGCAGTCAACTACTGCCCGTGCTCAACTTGCAGCAGAGAC
L.vulgare_iso-8_CH_Nyon_Pra      AGCCTTAAAATGGTGGGATAAGCAGTCAACTACTGCCCGTGCTCAACTTGCAGCAGAGAC
LigMV_FR                         AGCCTTAAAATGGTGGGATAAGCAGTCAACTACTGCCCGTGCTCAACTTGCAGCAGAGAC
L.vulgare_iso-6_AUT_Inns         AGCCTTAAAATGGTGGGATAAGCAGTCAACTACTGCCCGTGCTCAACTTGCAGCAGAGAC
LigMV_AUT                        AGCCTTAAAATGGTGGGATAAGCAGTCAACTACTGCCCGTGCTCAACTTGCAGCAGAGAC
L.vulgare_iso-5_CH_Veve          AGCCTTAAAATGGTGGGATAAGCAGTCAACTACTGCCCGTGCTCAACTTGCAGCAGAGAC
L.vulgare_iso-7_CH_Nyon_Gen      AGCCTTAAAATGGTGGGATAAGCAGTCAACTACTGCCCGTGCTCAACTTGCAGCAGAGAC
C.quinoa_iso-1288_CH_2007        AGCCTTAAAATGGTGGGATAAGCAGTCAACTACTGCCCGTGCTCAACTTGCAGCAGAGAC
LigMV_CH_dsRNA                   AGCCTTAAAATGGTGGGATAAGCAGTCAACTACTGCCCGTGCTCAACTTGCAGCAGAGAC
C.quinoa_iso-1340_CH_2007        AGCCTTAAAATGGTGGGATAAGCAGTCAACTACTGCCCGTGCTCAACTTGCAGCAGAGAC
                                 ********* ****************************************** * *****

L.vulgare_iso-3_CH_Biel          GAGACGACTTTGTGACATCGACTTCTGTACTTACAATTTTATGATCAAAAATGATGTAAA
L.vulgare_iso-4_CH_Biel          GAGACGACTTTGTGACATCGACTTCTGTACTTACAATTTTATGATCAAAAATGATGTAAA
L.vulgare_iso-1_CH_Nyon_Gla      GAGACGACTTTGTGACATCGACTTCTGTACTTACAATTTTATGATTAAAAATGATGTAAA
LigMV_gamma_CH_sRNA              GAGACGACTTTGTGACATCGACTTCTGTACTTACAATTTTATGATTAAAAATGATGTAAA
L.vulgare_iso-2_CH_Nyon_Gen      GAGACGACTTTGTGACATCGACTTCTGTACTTACAATTTTATGATCAAAAATGATGTAAA
L.vulgare_iso-8_CH_Nyon_Pra      GAGACGACTTTGTGACATCGACTTCTGTACTTACAATTTTATGATCAAAAATGATGTAAA
LigMV_FR                         GAGACGACTTTGTGACATCGACTTCTGTACTTACAATTTTATGATCAAAAATGATGTAAA
L.vulgare_iso-6_AUT_Inns         GAGACGACTTTGTGACATCGACTTCTGTACTTACAATTTTATGATCAAAAATGATGTAAA
LigMV_AUT                        GAGACGACTTTGTGACATCGACTTCTGTACTTACAATTTTATGATCAAAAATGATGTAAA
L.vulgare_iso-5_CH_Veve          GAGACGACTTTGTGACATCGACTTCTGTACTTACAATTTTATGATCAAAAATGATGTAAA
L.vulgare_iso-7_CH_Nyon_Gen      GAGACGACTTTGTGACATCGACTTCTGTACTTACAATTTTATGATCAAAAATGATGTAAA
C.quinoa_iso-1288_CH_2007        GAGACGACTTTGTGACATCGACTTCTGTACTTACAATTTTATGATCAAAAATGATGTAAA
LigMV_CH_dsRNA                   GAGACGACTTTGTGACATCGACTTCTGTACTTACAATTTTATGATCAAAAATGATGTAAA
C.quinoa_iso-1340_CH_2007        GAGACGACTTTGTGACATCGACTTCTGTACTTACAATTTTATGATCAAAAATGATGTAAA
                                 ********************************************* **************

L.vulgare_iso-3_CH_Biel          ACCGAAGTTGGATCTGACACCCCAAGTTGAGTATGCAGCTCTGCAAACGGTAGTATTTCC
L.vulgare_iso-4_CH_Biel          ACCGAAGTTGGATCTGACACCCCAAGTTGAGTATGCAGCTTTGCAAACGGTAGTATTTCC
L.vulgare_iso-1_CH_Nyon_Gla      ACCGAAGTTGGATCTGACACCCCAAGTTGAGTATGCAGCTTTGCAAACGGTAGTATTTCC
LigMV_gamma_CH_sRNA              ACCGAAGTTGGATCTGACACCCCAAGTTGAGTATGCAGCTTTGCAAACGGTAGTATTTCC
L.vulgare_iso-2_CH_Nyon_Gen      ACCGAAGTTGGATCTGACACCCCAAGTTGAGTATGCAGCTTTGCAAACCGTAGTATTTCC
L.vulgare_iso-8_CH_Nyon_Pra      ACCGAAGTTGGATCTGACACCCCAAGTTGAGTATGCAGCTTTGCAAACGGTAGTATTTCC
LigMV_FR                         ACCGAAGTTGGATCTGACACCCCAAGTTGAGTATGCAGCTTTGCAAACGGTAGTATTTCC
L.vulgare_iso-6_AUT_Inns         ACCGAAGTTGGATCTGACACCCCAAGTTGAGTATGCAGCTTTGCAAACGGTAGTATTTCC
LigMV_AUT                        ACCGAAGTTGGATCTGACACCCCAAGTTGAGTATGCAGCTTTGCAAACGGTAGTATTTCC
L.vulgare_iso-5_CH_Veve          ACCGAAGTTGGATCTGACACCCCAAGTTGAGTATGCAGCTTTGCAAACGGTAGTATTTCC
L.vulgare_iso-7_CH_Nyon_Gen      ACCGAAGTTGGATCTGACACCCCAAGTTGAGTATGCAGCTTTGCAAACGGTAGTATTTCC
C.quinoa_iso-1288_CH_2007        ACCGAAGTTGGATCTGACACCCCAAGTTGAGTATGCAGCTTTGCAAACGGTAGTATTTCC
LigMV_CH_dsRNA                   ACCGAAGTTGGATCTGACACCCCAAGTTGAGTATGCAGCTTTGCAAACGGTAGTATTTCC
C.quinoa_iso-1340_CH_2007        ACCGAAGTTGGATCTGACACCCCAAGTTGAGTATGCAGCTTTGCAAACGGTAGTATTTCC
                                 **************************************** ******* ***********

L.vulgare_iso-3_CH_Biel          TGATAAAATCGTGAATGCTTTTTTCGGCCCTGTTATAAAGGAAATTAATGAGAGGATCAT
L.vulgare_iso-4_CH_Biel          TGATAAAATCGTGAATGCTTTTTTCGGCCCTGTTATAAAGGAAATTAATGAGAGGATCAT
L.vulgare_iso-1_CH_Nyon_Gla      TGATAAAATCGTGAATGCTTTTTTCGGCCCTGTTATAAAGGAAATTAATGAGAGGATCAT
LigMV_gamma_CH_sRNA              TGATAAAATCGTGAATGCTTTTTTCGGCCCTGTTATAAAGGAAATTAATGAGAGGATCAT
L.vulgare_iso-2_CH_Nyon_Gen      TGATAAAATCGTGAATGCTTTTTTCGGCCCTGTTATAAAGGAAATTAATGAGAGGATCAT
L.vulgare_iso-8_CH_Nyon_Pra      TGATAAAATCGTGAATGCTTTTTTCGGCCCTGTTATAAAGGAAATTAATGAGAGGATCAT
LigMV_FR                         TGATAAAATCGTGAATGCTTTTTTCGGCCCTGTTATAAAGGAAATTAATGAGAGGATCAT
L.vulgare_iso-6_AUT_Inns         TGATAAAATCGTGAATGCTTTTTTCGGCCCTGTTATAAAGGAAATTAATGAGAGGATCAT
LigMV_AUT                        TGATAAAATCGTGAATGCTTTTTTCGGCCCTGTTATAAAGGAAATTAATGAGAGGATCAT
L.vulgare_iso-5_CH_Veve          TGATAAAATCGTGAATGCTTTTTTCGGCCCTGTTATAAAGGAAATTAATGAGAGGATCAT
L.vulgare_iso-7_CH_Nyon_Gen      TGATAAAATCGTGAATGCTTTTTTCGGCCCTGTTATAAAGGAAATTAATGAGAGGATCAT
C.quinoa_iso-1288_CH_2007        TGATAAAATCGTGAATGCTTTTTTCGGCCCTGTTATAAAGGAAATTAATGAGAGGATCAT
LigMV_CH_dsRNA                   TGATAAAATCGTGAATGCTTTTTTCGGCCCTGTTATAAAGGAAATTAATGAGAGGATCAT
C.quinoa_iso-1340_CH_2007        TGATAAAATCGTGAATGCTTTTTTCGGCCCTGTTATAAAGGAAATTAATGAGAGGATCAT
                                 ************************************************************

L.vulgare_iso-3_CH_Biel          TAGAGCTTTGAAACCTCATATTGTGTTTAATACACGTATGACGGCCGAAGGTTTAAATGA
L.vulgare_iso-4_CH_Biel          TAGAGCTTTGAAACCTCATATTGTGTTTAATACACGTATGACGGCCGAAGGTTTAAATGA
L.vulgare_iso-1_CH_Nyon_Gla      TAGAGCCTTGAAACCTCATATTGTGTTTAATACACGTATGACGGCCGAAGGTTTAAATGA
LigMV_gamma_CH_sRNA              TAGAGCCTTGAAACCTCATATTGTGTTTAATACACGTATGACGGCCGAAGGTTTAAATGA
L.vulgare_iso-2_CH_Nyon_Gen      TAGAGCCTTGAAACCTCATATTGTGTTTAATACACGTATGACGGYCGAAGGTTTAAATGA
L.vulgare_iso-8_CH_Nyon_Pra      TAGAGCCTTGAAACCTCATATTGTGTTTAATACACGTATGACGGCCGAAGGTTTAAATGA
LigMV_FR                         TAGAGCCTTGAAACCTCATATTGTGTTTAATACACGTATGACGGCCGAAGGTTTAAATGA
L.vulgare_iso-6_AUT_Inns         TAGAGCCTTGAAACCTCATATTGTGTTTAATACACGTATGACGGCCGAAGGTTTAAATGA
LigMV_AUT                        TAGAGCCTTGAAACCTCATATTGTGTTTAATACACGTATGACGGCCGAAGGTTTAAATGA
L.vulgare_iso-5_CH_Veve          TAGAGCCTTGAAACCTCATATTGTGTTTAATACACGTATGACGGCCGAAGGTTTAAATGA
L.vulgare_iso-7_CH_Nyon_Gen      TAGAGCCTTGAAACCTCATATTGTGTTTAATACACGTATGACGGCCGAAGGTTTAAATGA
C.quinoa_iso-1288_CH_2007        TAGAGCCTTGAAACCTCATATTGTGTTTAATACACGTATGACGGCCGAAGGTTTAAATGA
LigMV_CH_dsRNA                   TAGAGCCTTGAAACCTCATATTGTGTTTAATACACGTATGACGGCCGAAGGTTTAAATGA
C.quinoa_iso-1340_CH_2007        TAGAGCCTTGAAACCTCATATTGTGTTTAATACACGTATGACGGCCGAAGGTTTAAATGA
                                 ****** ************************************* ***************

L.vulgare_iso-3_CH_Biel          TACTATTTCATTTCTGCCCCCCAAGTCTTACAGGTCACTTGAAATCGATTTCTCAAAATT
L.vulgare_iso-4_CH_Biel          TACTATTTCATTTCTGCCCCCCAAGTCTTACAGGTCACTTGAAATCGATTTCTCAAAATT
L.vulgare_iso-1_CH_Nyon_Gla      TACTATTTCATTTCTGCCCCCCAAGTCTTACAGGTCACTTGAAATCGATTTCTCAAAATT
LigMV_gamma_CH_sRNA              TACTATTTCATTTCTGCCCCCCAAGTCTTACAGGTCACTTGAAATCGATTTCTCAAAATT
L.vulgare_iso-2_CH_Nyon_Gen      TACTATTTCATTTCTGCCCCCCAAGTCTTACAGGTCACTTGAAATCGATTTCTCAAAATT
L.vulgare_iso-8_CH_Nyon_Pra      TACTATTTCATTTCTGCCCCCCAAGTCTTACAGGTCACTTGAAATCGATTTCTCAAAATT
LigMV_FR                         TACTATTTCATTTCTGCCCCCCAAGTCTTACAGGTCACTTGAAATCGATTTCTCAAAATT
L.vulgare_iso-6_AUT_Inns         TACTATTTCATTTCTGCCCCCCAAGTCTTACAGGTCACTTGAAATCGATTTCTCAAAATT
LigMV_AUT                        TACTATTTCATTTCTGCCCCCCAAGTCTTACAGGTCACTTGAAATCGATTTCTCAAAATT
L.vulgare_iso-5_CH_Veve          TACTATTTCATTTCTGCCCCCCAAGTCTTACAGGTCACTTGAAATCGATTTCTCAAAATT
L.vulgare_iso-7_CH_Nyon_Gen      TACTATTTCATTTCTGCCCCCCAAGTCTTACAGGTCACTTGAAATCGATTTCTCAAAATT
C.quinoa_iso-1288_CH_2007        TACTATTTCATTTCTGCCCCCCAAGTCTTACAGGTCACTTGAAATCGATTTCTCAAAATT
LigMV_CH_dsRNA                   TACTATTTCATTTCTGCCCCCCAAGTCTTACAGGTCACTTGAAATCGATTTCTCAAAATT
C.quinoa_iso-1340_CH_2007        TACTATTTCATTTCTGCCCCCCAAGTCTTACAGGTCACTTGAAATCGATTTCTCAAAATT
```

```
                                    ************************************************************

L.vulgare_iso-3_CH_Biel             TGACAAGTCAAAGACTGGTCTGCATATTAAATGTGTAATAGCTTTATACAAATTGTTTGG
L.vulgare_iso-4_CH_Biel             TGACAAGTCAAAGACTGGTCTGCATATTAAATGTGTAATAGCTTTATACAAATTGTTTGG
L.vulgare_iso-1_CH_Nyon_Gla         TGACAAGTCAAAGACTGGTCTGCATATTAAATGTGTAATAGCTTTATACAAATTGTTTGG
LigMV_gamma_CH_sRNA                 TGACAAGTCAAAGACTGGTCTGCATATTAAATGTGTAATAGCTTTATACAAATTGTTTGG
L.vulgare_iso-2_CH_Nyon_Gen         TGACAAGTCAAAGACTGGTCTGCATATTAAATGTGTAATAGCTTTATACAAATTGTTTGG
L.vulgare_iso-8_CH_Nyon_Pra         TGACAAGTCAAAGACTGGTCTGCATATTAAATGTGTAATAGCTTTATACAAATTGTTTGG
LigMV_FR                            TGACAAGTCAAAGACTGGTCTGCATATTAAATGTGTAATAGCTTTATACAAATTGTTTGG
L.vulgare_iso-6_AUT_Inns            TGACAAGTCAAAGACTGGTCTGCATATTAAATGTGTAATAGCTTTATACAAACTGTTTGG
LigMV_AUT                           TGACAAGTCAAAGACTGGTCTGCATATTAAATGTGTAATAGCTTTATACAAATTGTTTGG
L.vulgare_iso-5_CH_Veve             TGACAAGTCAAAGACTGGTCTGCATATTAAATGTGTAATAGCTTTATACAAATTGTTTGG
L.vulgare_iso-7_CH_Nyon_Gen         TGACAAGTCAAAGACTGGTCTGCATATTAAATGTGTAATAGCTTTATACAAATTGTTTGG
C.quinoa_iso-1288_CH_2007           TGACAAGTCAAAGACTGGTCTGCATATTAAATGTGTAATAGCTTTATACAAATTGTTTGG
LigMV_CH_dsRNA                      TGACAAGTCAAAGACTGGTCTGCATATTAAATGTGTAATAGCTTTATACAAATTGTTTGG
C.quinoa_iso-1340_CH_2007           TGACAAGTCAAAGACTGGTCTGCATATTAAATGTGTAATAGCTTTATACAAATTGTTTGG
                                    ***************************************************** ******

L.vulgare_iso-3_CH_Biel             ACTGGATGGAATCCTAGAGGTGCTCTGGGAGAAATCTCAAATACAAACATACGTAAAGGA
L.vulgare_iso-4_CH_Biel             ACTGGATGGAATCTTAGAGGTGCTCTGGGAGAAATCTCAAGTACAAACATACGTAAAGGA
L.vulgare_iso-1_CH_Nyon_Gla         ACTGGATGGAATCCTAGAGGTGCTCTGGGAGAAATCTCAAGTACAAACATACGTAAAGGA
LigMV_gamma_CH_sRNA                 ACTGGATGGAATCCTAGAGGTGCTCTGGGAGAAATCTCAAGTACAAACATACGTAAAGGA
L.vulgare_iso-2_CH_Nyon_Gen         ACTGGATGGAATCCTAGAGGTGCTCTGGGAGAAATCTCAAGTACAAACATACGTAAAGGA
L.vulgare_iso-8_CH_Nyon_Pra         ACTGGATGGAATCCTAGAGGTGCTCTGGGAGAAATCTCAAGTACAAACATACGTAAAGGA
LigMV_FR                            ACTGGATGGAATCCTAGAGGTGCTCTGGGAGAAATCTCAAGTACAAACATACGTAAAGGA
L.vulgare_iso-6_AUT_Inns            ACTGGATGGAATCCTAGAGGTGCTCTGGGAGAAATCTCAAGTACAAACATACGTAAAGGA
LigMV_AUT                           ACTGGATGGAATCCTAGAGGTGCTCTGGGAGAAATCTCAAGTACAAACATACGTAAAGGA
L.vulgare_iso-5_CH_Veve             ACTGGATGGAATCCTAGAGGTGCTCTGGGAGAAATCTCAAGTACAAACATACGTAAAGGA
L.vulgare_iso-7_CH_Nyon_Gen         ACTGGATGGAATCCTAGAGGTGCTCTGGGAGAAATCTCAAGTACAAACATACGTAAAGGA
C.quinoa_iso-1288_CH_2007           ACTGGATGGAATCCTAGAGGTGCTCTGGGAGAAATCTCAAGTACAAACATACGTAAAGGA
LigMV_CH_dsRNA                      ACTGGATGGAATCCTAGAGGTGCTCTGGGAGAAATCTCAAGTACAAACATACGTAAAGGA
C.quinoa_iso-1340_CH_2007           ACTGGATGGAATCCTAGAGGTGCTCTGGGAGAAATCTCAAGTACAAACATACGTAAAGGA
                                    ************* ************************** *******************

L.vulgare_iso-3_CH_Biel             CCGAAATTTTGGTCTTGAAGCTTACCTTTTATTTCAGCAGAAATCAGGAAATTGTGATAC
L.vulgare_iso-4_CH_Biel             CCGAAATTTTGGTCTTGAAGCTTACCTTTTATTTCAGCAGAAATCAGGAAATTGTGATAC
L.vulgare_iso-1_CH_Nyon_Gla         CCGAAATTTTGGTCTTGAAGCTTACCTTTTATTTCAGCAGAAATCAGGAAATTGTGACAC
LigMV_gamma_CH_sRNA                 CCGAAATTTTGGTCTTGAAGCTTACCTTTTATTTCAGCAGAAATCAGGAAATTGTGACAC
L.vulgare_iso-2_CH_Nyon_Gen         CCGAAATTTTGGTCTTGAAGCTTACCTTTTATTTCAGCAGAAATCAGGAAATTGTGACAC
L.vulgare_iso-8_CH_Nyon_Pra         CCGAAATTTTGGTCTTGAAGCTTACCTTTTATTTCAGCAGAAATCAGGAAATTGTGACAC
LigMV_FR                            CCGAAATTTTGGTCTTGAAGCTTACCTTTTATTTCAGCAGAAATCAGGAAATTGTGACAC
L.vulgare_iso-6_AUT_Inns            CCGAAATTTTGGTCTTGAAGCTTACCTTTTATTTCAGCAGAAATCAGGAAATTGTGACAC
LigMV_AUT                           CCGAAATTTTGGTCTTGAAGCTTACCTTTTATTTCAGCAGAAATCAGGAAATTGTGACAC
L.vulgare_iso-5_CH_Veve             CCGAAATTTTGGTCTTGAAGCTTACCTTTTATTTCAGCAGAAATCAGGAAATTGTGACAC
L.vulgare_iso-7_CH_Nyon_Gen         CCGAAATTTTGGTCTTGAAGCTTACCTTTTATTTCAGCAGAAATCAGGAAATTGTGACAC
C.quinoa_iso-1288_CH_2007           CCGAAATTTTGGTCTTGAAGCTTACCTTTTATTTCAGCAGAAATCAGGAAATTGTGACAC
LigMV_CH_dsRNA                      CCGAAATTTTGGTCTTGAAGCTTACCTTTTATTTCAGCAGAAATCAGGAAATTGTGACAC
C.quinoa_iso-1340_CH_2007           CCGAAATTTTGGTCTTGAAGCTTACCTTTTATTTCAGCAGAAATCAGGAAATTGTGACAC
                                    ********************************************************* **

L.vulgare_iso-3_CH_Biel             TTACGGTTCTAATACTTGGTCTGCAGCATTAGCGTTGCTAGATTGTTTGCCTTTGGAGCA
L.vulgare_iso-4_CH_Biel             TTACGGTTCTAATACTTGGTCTGCAGCATTAGCGTTGCTAGATTGTTTGCCTTTGGAGCA
L.vulgare_iso-1_CH_Nyon_Gla         TTACGGTTCTAATACTTGGTCTGCAGCATTAGCGTTGCTAGATTGTTTGCCTTTGGAGCA
LigMV_gamma_CH_sRNA                 TTACGGTTCTAATACTTGGTCTGCAGCATTAGCGTTGCTAGATTGTTTGCCTTTGGAGCA
L.vulgare_iso-2_CH_Nyon_Gen         TTACGGTTCTAATACTTGGTCTGCAGCATTAGCGTTGCTAGATTGTTTGCCTTTGGAGCA
L.vulgare_iso-8_CH_Nyon_Pra         TTACGGTTCTAATACTTGGTCTGCAGCATTAGCGTTGCTAGATTGTTTGCCTTTGGAGCA
LigMV_FR                            TTACGGTTCTAATACTTGGTCTGCAGCATTAGCGTTGCTAGATTGTTTGCCTTTGGAGCA
L.vulgare_iso-6_AUT_Inns            TTACGGTTCTAATACTTGGTCTGCAGCATTAGCGTTGCTAGATTGTTTGCCTTTGGAGCA
LigMV_AUT                           TTACGGTTCTAATACTTGGTCTGCAGCATTAGCGTTGCTAGATTGTTTGCCTTTGGAGCA
L.vulgare_iso-5_CH_Veve             TTACGGTTCTAATACTTGGTCTGCAGCATTAGCGTTGCTAGATTGTTTGCCTTTGGAGCA
L.vulgare_iso-7_CH_Nyon_Gen         TTACGGTTCTAATACTTGGTCTGCAGCATTAGCGTTGCTAGATTGTTTGCCTTTGGAGCA
C.quinoa_iso-1288_CH_2007           TTACGGTTCTAATACTTGGTCTGCAGCATTAGCGTTGCTAGATTGTTTGCCTTTGGAGCA
LigMV_CH_dsRNA                      TTACGGTTCTAATACTTGGTCTGCAGCATTAGCGTTGCTAGATTGTTTGCCTTTGGAGCA
C.quinoa_iso-1340_CH_2007           TTACGGTTCTAATACTTGGTCTGCAGCATTAGCGTTGCTAGATTGTTTGCCTTTGGAGCA
                                    ************************************************************

L.vulgare_iso-3_CH_Biel             TTCAGTCTATGCGGTTTTTGGTGGAGACGATTCTTTGATACTATTTGAGGAGGGGTATCT
L.vulgare_iso-4_CH_Biel             TTCAGTCTATGCGGTTTTTGGTGGAGACGATTCTTTGATACTATTTGAGGAGGGGTATCT
L.vulgare_iso-1_CH_Nyon_Gla         TTCAGTCTATGCGGTTTTTGGTGGAGACGATTCTTTGATACTATTTGAGGAGGGGTATCT
LigMV_gamma_CH_sRNA                 TTCAGTCTATGCGGTTTTTGGTGGAGACGATTCTTTGATACTATTTGAGGAGGGGTATCT
L.vulgare_iso-2_CH_Nyon_Gen         TTCAGTCTATGCGGTTTTTGGTGGAGACGATTCTTTGATACTATTTGAGGAGGGGTATCT
L.vulgare_iso-8_CH_Nyon_Pra         TTCAGTCTATGCGGTTTTTGGTGGAGACGATTCTTTGATACTATTTGAGGAGGGGTATCT
LigMV_FR                            TTCAGTCTATGCGGTTTTTGGTGGAGACGATTCTTTGATACTATTTGAGGAGGGGTATCT
L.vulgare_iso-6_AUT_Inns            TTCAGTCTATGCGGTTTTTGGTGGAGACGATTCTTTGATACTATTTGAGGAGGGGTATCT
LigMV_AUT                           TTCAGTCTATGCGGTTTTTGGTGGAGACGATTCTTTGATACTATTTGAGGAGGGGTATCT
L.vulgare_iso-5_CH_Veve             TTCAGTCTATGCGGTTTTTGGTGGAGACGATTCTTTGATACTATTTGAGGAGGGGTATCT
L.vulgare_iso-7_CH_Nyon_Gen         TTCAGTCTATGCGGTTTTTGGTGGAGACGATTCTTTGATACTATTTGAGGAGGGGTATCT
C.quinoa_iso-1288_CH_2007           TTCAGTCTATGCGGTTTTTGGTGGAGACGATTCTTTGATACTATTTGAGGAGGGGTATCT
LigMV_CH_dsRNA                      TTCAGTCTATGCGGTTTTTGGTGGAGACGATTCTTTGATACTATTTGAGGAGGGGTATCT
C.quinoa_iso-1340_CH_2007           TTCAGTCTATGCGGTTTTTGGTGGAGACGATTCTTTGATACTATTTGAGGAGGGGTATCT
                                    ************************************************************

L.vulgare_iso-3_CH_Biel             TGTTAATGACCCATGCAAGCGTTTGGCTGGAACGTGGAATTTTGAATGCAAAATTTTTGC
L.vulgare_iso-4_CH_Biel             TGTTAATGACCCATGCAAGCGTTTGGCTGGAACGTGGAATTTTGAATGCAAAATTTTTGC
L.vulgare_iso-1_CH_Nyon_Gla         TGTTAATGACCCATGCAAGCGTTTGGCTGGAACGTGGAATTTTGAATGCAAAATTTTTGC
LigMV_gamma_CH_sRNA                 TGTTAATGACCCATGCAAGCGTTTGGCTGGAACGTGGAATTTTGAATGCAAAATTTTTGC
L.vulgare_iso-2_CH_Nyon_Gen         TGTTAATGACCCATGCAAGCGTTTGGCTGGAACGTGGAATTTTGAATGCAAAATTTTTGC
L.vulgare_iso-8_CH_Nyon_Pra         TGTTAATGACCCATGCAAGCGTTTGGCTGGAACGTGGAATTTTGAATGCAAAATTTTTGC
LigMV_FR                            TGTTAATGACCCATGCAAGCGTTTGGCTGGAACGTGGAATTTTGAATGCAAAATTTTTGC
L.vulgare_iso-6_AUT_Inns            TGTTAATGACCCATGCAAGCGTTTGGCTGGAACGTGGAATTTTGAATGCAAAATTTTTGC
LigMV_AUT                           TGTTAATGACCCATGCAAGCGTTTGGCTGGAACGTGGAATTTTGAATGCAAAATTTTTGC
L.vulgare_iso-5_CH_Veve             TGTTAATGACCCATGCAAGCGTTTGGCTGGAACGTGGAATTTTGAATGCAAAATTTTTGC
L.vulgare_iso-7_CH_Nyon_Gen         TGTTAATGACCCATGCAAGCGTTTGGCTGGAACGTGGAATTTTGAATGCAAAATTTTTGC
C.quinoa_iso-1288_CH_2007           TGTTAATGACCCATGCAAGCGTTTGGCTGGAACGTGGAATTTTGAATGCAAAATTTTTGC
LigMV_CH_dsRNA                      TGTTAATGACCCATGCAAGCGTTTGGCTGGAACGTGGAATTTTGAATGCAAAATTTTTGC
C.quinoa_iso-1340_CH_2007           TGTTAATGACCCATGCAAGCGTTTGGCTGGAACGTGGAATTTTGAATGCAAAATTTTTGC
                                    ************************************************************

L.vulgare_iso-3_CH_Biel             TTTCAATTTTCCTTCCTTTTGCGGGAAGTTTGTTGTCAGTGTTGATGACAAATATGTTTT
L.vulgare_iso-4_CH_Biel             TTTCAATTTTCCTTCCTTTTGCGGGAAGTTTGTTGTCAGTGTTGATGACAAATATGTTTT
L.vulgare_iso-1_CH_Nyon_Gla         TTTCAATTTTCCTTCCTTTTGCGGGAAGTTTGTTGTCAGTGTTGACGACAAATATGTTTT
LigMV_gamma_CH_sRNA                 TTTCAATTTTCCTTCCTTTTGCGGGAAGTTTGTTGTCAGTGTTGACGACAAATATGTTTT
L.vulgare_iso-2_CH_Nyon_Gen         TTTCAATTTTCCTTCCTTTTGCGGGAAGTTTGTTGTCAGTGTTGACGACAAATATGTTTT
L.vulgare_iso-8_CH_Nyon_Pra         TTTCAATTTTCCTTCCTTTTGCGGGAAGTTTGTTGTCAGTGTTGACGACAAATATGTTTT
LigMV_FR                            TTTCAATTTTCCTTCCTTTTGCGGGAAGTTTGTTGTCAGTGTTGACGACAAATATGTTTT
L.vulgare_iso-6_AUT_Inns            TTTCAATTTTCCTTCCTTTTGCGGGAAGTTTGTTGTCAGTGTTGACGACAAATATGTTTT
LigMV_AUT                           TTTCAATTTTCCTTCCTTTTGCGGGAAGTTTGTTGTCAGTGTTGACGACAAATATGTTTT
L.vulgare_iso-5_CH_Veve             TTTCAATTTTCCTTCCTTTTGCGGGAAGTTTGTTGTCAGTGTTGACGACAAATATGTTTT
L.vulgare_iso-7_CH_Nyon_Gen         TTTCAATTTTCCTTCCTTTTGCGGGAAGTTTGTTGTCAGTGTTGACGACAAATATGTTTT
C.quinoa_iso-1288_CH_2007           TTTCAATTTTCCTTCCTTTTGCGGGAAGTTTGTTGTCAGTGTTGACGACAAATATGTTTT
LigMV_CH_dsRNA                      TTTCAATTTTCCTTCCTTTTGCGGGAAGTTTGTTGTCAGTGTTGACGACAAATATGTTTT
C.quinoa_iso-1340_CH_2007           TTTCAATTTTCCTTCCTTTTGCGGGAAGTTTGTTGTCAGTGTTGACGACAAATATGTTTT
                                    ********************************************* **************

L.vulgare_iso-3_CH_Biel             TGTACCAGATGCTGCTAAGCTAATCACAAAACTGGGGAGAACCGACGTAAAGGATGTAGA
L.vulgare_iso-4_CH_Biel             TGTACCAGATGCTGCTAAGCTAATCACAAAACTGGGGAGAACCGACGTAAAGGATGTAGA
L.vulgare_iso-1_CH_Nyon_Gla         TGTACCAGATGCTGCTAAGCTAATCACAAAACTGGGGAGAACCGACGTAAAGGATGTAGA
LigMV_gamma_CH_sRNA                 TGTACCAGATGCTGCTAAGCTAATCACAAAACTGGGGAGAACCGACGTAAAGGATGTAGA
L.vulgare_iso-2_CH_Nyon_Gen         TGTACCAGATGCTGCTAAGCTAATCACAAAACTGGGGAGAACCGACGTAAAGGATGTAGA
L.vulgare_iso-8_CH_Nyon_Pra         TGTACCAGATGCTGCTAAGCTAATCACAAAACTGGGGAGAACCGACGTAAAGGATGTAGA
LigMV_FR                            TGTACCAGATGCTGCTAAGCTAATCACAAAACTGGGGAGAACCGATGTAAAGGATGTAGA
L.vulgare_iso-6_AUT_Inns            TGTACCAGATGCTGCTAAGCTAATCACAAAACTGGGGAGAACCGACGTAAAGGATGTAGA
LigMV_AUT                           TGTACCAGATGCTGCTAAGCTAATCACAAAACTGGGGAGAACCGACGTAAAGGATGTAGA
L.vulgare_iso-5_CH_Veve             TGTACCAGATGCTGCTAAGCTAATCACAAAACTGGGGAGAACCGACGTAAAGGATGTAGA
L.vulgare_iso-7_CH_Nyon_Gen         TGTACCAGATGCTGCTAAGCTAATCACAAAACTGGGGAGAACCGACGTAAAGGATGTAGA
C.quinoa_iso-1288_CH_2007           TGTACCAGATGCTGCTAAGCTAATCACAAAACTGGGGAGAACCGACGTAAAGGATGTAGA
LigMV_CH_dsRNA                      TGTACCAGATGCTGCTAAGCTAATCACAAAACTGGGGAGAACCGACGTAAAGGATGTAGA
C.quinoa_iso-1340_CH_2007           TGTACCAGATGCTGCTAAGCTAATCACAAAACTGGGGAGAACCGACGTAAAGGATGTAGA
```

```
                                  ********************************************* **************

L.vulgare_iso-3_CH_Biel           AGCTTTAAGTGAAATTTACATTTCAATTAATGACAATTACAGATCCTTTAAGGATTTCCG
L.vulgare_iso-4_CH_Biel           AGCTTTAAGTGAAATTTACATTTCAATTAATGACAATTACAGATCCTTTAAGGATTTCCG
L.vulgare_iso-1_CH_Nyon_Gla       AGCTTTAAGTGAAATTTACATTTCAATTAATGACAATTACAGATCCTTTAAGGATTTCCG
LigMV_gamma_CH_sRNA               AGCTTTAAGTGAAATTTACATTTCAATTAATGACAATTACAGATCCTTTAAGGATTTCCG
L.vulgare_iso-2_CH_Nyon_Gen       AGCTTTAAGTGAAATTTACATTTCAATTAATGACAAYTACAGATCCTTTAAGGATTTCCG
L.vulgare_iso-8_CH_Nyon_Pra       AGCTTTAAGTGAAATTTACATTTCAATTAATGACAATTACAGATCCTTTAAGGATTTCCG
LigMV_FR                          AGCTTTAAGTGAAATTTACATTTCAATTAATGACAATTACAGATCCTTTAAGGATTTCCG
L.vulgare_iso-6_AUT_Inns          AGCTTTAAGTGAAATTTACATTTCAATTAATGACAATTACAGATCCTTTAAGGATTTCCG
LigMV_AUT                         AGCTTTAAGTGAAATTTACATTTCAATTAATGACAATTACAGATCCTTTAAGGATTTCCG
L.vulgare_iso-5_CH_Veve           AGCTTTAAGTGAAATTTACATTTCAATTAATGACAATTACAGATCCTTTAAGGATTTCCG
L.vulgare_iso-7_CH_Nyon_Gen       AGCTTTAAGTGAAATTTACATTTCAATTAATGACAATTACAGATCCTTTAAGGATTTCCG
C.quinoa_iso-1288_CH_2007         AGCTTTAAGTGAAATTTACATTTCAATTAATGACAATTACAGATCCTTTAAGGATTTCCG
LigMV_CH_dsRNA                    AGCTTTAAGTGAAATTTACATTTCAATTAATGACAATTACAGATCCTTTAAGGATTTCCG
C.quinoa_iso-1340_CH_2007         AGCTTTAAGTGAAATTTACATTTCAATTAATGACAATTACAGATCCTTTAAGGATTTCCG
                                  ************************************ ***********************

L.vulgare_iso-3_CH_Biel           TGTTTTGGATGAGTTGAGCGATGCTTTGGTTGATAGATATCATGCCCCTTACGACGCTTT
L.vulgare_iso-4_CH_Biel           TGTTTTGGATGAGTTGAGCGATGCTTTGGTTGATAGATATCATGCCCCTTACGACGCTTT
L.vulgare_iso-1_CH_Nyon_Gla       TGTTTTGGATGAGTTGAGCGATGCTTTGGTTGATAGATATCATGCTCCTTACGACGCTTT
LigMV_gamma_CH_sRNA               TGTTTTGGATGAGTTGAGCGATGCTTTGGTTGATAGATATCATGCTCCTTACGACGCTTT
L.vulgare_iso-2_CH_Nyon_Gen       TGTTTTGGATGAGTTGAGCGATGCTTTGGTTGATAGATATCATGCYCCTTACGACGCTTT
L.vulgare_iso-8_CH_Nyon_Pra       TGTTTTGGATGAGTTGAGCGATGCTTTGGTTGATAGATATCATGCCCCTTACGATGCTTT
LigMV_FR                          TGTTTTGGATGAGTTGAGCGATGCTTTGGTTGATAGATATCATGCCCCTTACGACGCTTT
L.vulgare_iso-6_AUT_Inns          TGTTTTGGATGAGTTGAGCGATGCTTTGGTTGATAGATATCATGCCCCTTACGACGCTTT
LigMV_AUT                         TGTTTTGGATGAGTTGAGCGATGCTTTGGTTGATAGATATCATGCCCCTTACGACGCTTT
L.vulgare_iso-5_CH_Veve           TGTTTTGGATGAGTTAAGCGATGCTTTGGTTGATAGATATCATGCCCCTTACGACGCTTT
L.vulgare_iso-7_CH_Nyon_Gen       TGTTTTGGATGAGTTGAGCGATGCTTTGGTTGATAGATATCATGCCCCTTACGACGCTTT
C.quinoa_iso-1288_CH_2007         TGTTTTGGATGAGTTGAGCGATGCTTTGGTTGATAGATATCATGCCCCTTACGACGCTTT
LigMV_CH_dsRNA                    TGTTTTGGATGAGTTGAGCGATGCTTTGGTTGATAGATATCATGCCCCTTACGACGCTTT
C.quinoa_iso-1340_CH_2007         TGTTTTGGATGAGTTGAGCGATGCTTTGGTTGATAGATATCATGCCCCTTACGACGCTTT
                                  *************** ***************************** ******** *****

L.vulgare_iso-3_CH_Biel           ATCTGCTTTGGTGTCTCTGTGTTACTATATTTTCGATTTCGACAACTTTAAGCAGCTGTT
L.vulgare_iso-4_CH_Biel           ATCTGCTTTGGTGTCTCTGTGTTACTATATTTTCGATTTCGACAACTTTAAGCAGCTGTT
L.vulgare_iso-1_CH_Nyon_Gla       ATCTGCTTTGGTGTCTCTGTGTTACTATATATTCGATTTCGACAACTTTAAGCAGCTGTT
LigMV_gamma_CH_sRNA               ATCTGCTTTGGTGTCTCTGTGTTACTATATATTCGATTTCGACAACTTTAAGCAGCTGTT
L.vulgare_iso-2_CH_Nyon_Gen       ATCTGCTTTGGTGTCTCTGTGTTACTATATATTCGATTTCGACAACTTTAAGCAGCTGTT
L.vulgare_iso-8_CH_Nyon_Pra       ATCTGCTTTGGTGTCTCTGTGTTACTATATATTCGATTTCGACAACTTTAAGCAGCTGTT
LigMV_FR                          ATCTGCTTTGGTGTCTCTGTGTTACTATATATTCGATTTCGACAACTTTAAGCAGCTGTT
L.vulgare_iso-6_AUT_Inns          ATCTGCTTTGGTGTCTCTGTGTTACTATATATTCGATTTCGACAACTTTAAGCAGCTGTT
LigMV_AUT                         ATCTGCTTTGGTGTCTCTGTGTTACTATATATTCGATTTCGACAACTTTAAGCAGCTGTT
L.vulgare_iso-5_CH_Veve           ATCTGCTTTGGTGTCTCTGTGTTACTATATATTCGATTTCGACAACTTTAAGCAGCTGTT
L.vulgare_iso-7_CH_Nyon_Gen       ATCTGCTTTGGTGTCTCTGTGTTACTATATTTTCGATTTCGACAACTTTAAGCAGCTGTT
C.quinoa_iso-1288_CH_2007         ATCTGCTTTGGTGTCTCTGTGTTACTATATTTTCGATTTCGACAACTTTAAGCAGCTGTT
LigMV_CH_dsRNA                    ATCTGCTTTGGTGTCTCTGTGTTACTATATTTTCGATTTCGACAACTTTAAGCAGCTGTT
C.quinoa_iso-1340_CH_2007         ATCTGCTTTGGTGTCTCTGTGTTACTATATTTTCGATTTCGACAACTTTAAGCAGCTGTT
                                  ****************************** *****************************

L.vulgare_iso-3_CH_Biel           TGGTTGTGATGGCAATTTTGTGGATAAAATAATTGGAAAGGATTTCGATTGGTGAATCCT
L.vulgare_iso-4_CH_Biel           TGGTTGTGATGGCAATTTTGTGGATAAAATAATTGGAAAGGATTTCGATTGGTGAATCCT
L.vulgare_iso-1_CH_Nyon_Gla       TGGTTGTGATGGCAATTTTGTGGATAAAATAATTGGAAAGGATTTCGATTGGTGAATCCT
LigMV_gamma_CH_sRNA               TGGTTGTGATGGCAATTTTGTGGATAAAATAATTGGAAAGGATTTCGATTGGTGAATCCT
L.vulgare_iso-2_CH_Nyon_Gen       TGGTTGTGATGGCAATTTTGTGGATAAAATAATTGGAAAGGATTTCGATTGGTGAATCCT
L.vulgare_iso-8_CH_Nyon_Pra       TGGTTGTGATGGCAATTTTGTGGATAAAATAATTGGAAAGGAT-----------------
LigMV_FR                          TGGTTGTGATGGCAATTTTGTGGATAAAATAATTGGAAAGGATTTCGATTGGTGAATCCT
L.vulgare_iso-6_AUT_Inns          TGGTTGTGATGGCAATTTTGTGGATAAAATAATTGGAAAGGATTTCGATTGGTGAATCCT
LigMV_AUT                         TGGTTGTGATGGCAATTTTGTGGATAAAATAATTGGAAAGGATTTCGATTGGTGAATCCT
L.vulgare_iso-5_CH_Veve           TGGTTGTGATGGCAATTTTGTGGATAAAATAATTGGAAAGGATTTCGATTGGTGAATCCT
L.vulgare_iso-7_CH_Nyon_Gen       TGGTTGTGATGGCAATTTTGTGGATAAAATAATTGGAAAGGATTTCGATTGN-NAATCCT
C.quinoa_iso-1288_CH_2007         TGGTTGTGATGGCAATTTTGTGGATAAAATAATTGGAAAGGATTTCGATTGGTGAATCCT
LigMV_CH_dsRNA                    TGGTTGTGATGGCAATTTTGTGGATAAAATAATTGGAAAGGATTTCGATTGGTGAATCCT
C.quinoa_iso-1340_CH_2007         TGGTTGTGATGGCAATTTTGTGGATAAAATAATTGGAAAGGATTTCGATTGGTGAATCCT
                                  *******************************************

L.vulgare_iso-3_CH_Biel           TGATGTATTTTTCCTAACATTNNNGC-TTTTTACCAAATGAGCANCCAACCNNAGAGTTT
L.vulgare_iso-4_CH_Biel           TGATGTATTTTT-CC---------------------------------------------
L.vulgare_iso-1_CH_Nyon_Gla       TGATGTATTTTT-CTAACATTTTTGC-TTTTTACCAAATG-GCAGCNNCCNNAAANTTT-
LigMV_gamma_CH_sRNA               TGATGTATTTTT-CTAACATTTTTGC-TTTTTACCAAATG-GCAGCCAACCCAAGAGTTT
L.vulgare_iso-2_CH_Nyon_Gen       TGATGTATTTTT-CTAACATTTTTGC-TTTTTACCAAATG-GCAGCNNCCNNAAANTTT-
L.vulgare_iso-8_CH_Nyon_Pra       ------------------------------------------------------------
LigMV_FR                          TGATGTATTTTT-CTAACACTTTTGCTTTTTTACCAAATG-GCAGCCAACCCGAGAGTTT
L.vulgare_iso-6_AUT_Inns          TGATGTATTTTT-CTAACATTTTTGC-TTTTTACCAAATG-GCAGCCAAC----------
LigMV_AUT                         TGATGTATTTTT-CTAACATTTTTGC-TTTTTACCAAATG-GCAGCCAACCCAAGAGTTT
L.vulgare_iso-5_CH_Veve           TGATGTA-----------------------------------------------------
L.vulgare_iso-7_CH_Nyon_Gen       TGATGTATT---------------------------------------------------
C.quinoa_iso-1288_CH_2007         TGATGTATTTTT-CTAACATTTTTGC-TTTTTACCAAATG-GCAGCCAACCC--------
LigMV_CH_dsRNA                    TGATGTATTTTT-CTAACATTTTTGC-TTTTTACCAAATG-GCAGCCAACCCAAGAGTTT
C.quinoa_iso-1340_CH_2007         TGATGTATTTTT-CTAACA-----------------------------------------
```
